# Microhydration of Methylated Pyrazoles and Imidazoles: Robust Vibrational Resonances in Red-Shifted Water

**DOI:** 10.1021/acs.jpca.6c04895

**Published:** 2026-10-27

**Authors:** Finn Knüppe, Eaindra Lwin, Martin A. Suhm

**Affiliations:** Institute of Physical Chemistry, 9375 University of Göttingen, Tammannstr. 6, Göttingen 37077, Germany

## Abstract

Mono- and dihydrates of 11 *N*-methylated pyrazoles and imidazoles are studied as vacuum-isolated complexes at low temperature by linear infrared spectroscopy to reveal the interplay between the hydrogen-bonded water OH stretching fundamentals and anharmonic resonance partners. The water bending overtones, with and without additional stretching motion of a water unit against the diazole, are identified as the dominant resonance partners. Methyl substitution at the ring allows to tune the water OH stretch wavenumber through these resonances. A simple nested 2 × 2 model Hamiltonian, in combination with harmonic DFT predictions, provides an effective joint description of the anharmonic spectra of all 22 investigated hydrogen-bonded complexes, including resonance-induced intensity redistribution patterns. Future multidimensional anharmonic quantum calculations can test the extent to which the proposed empirical model is successful for the right reasons and the extent to which it may just mimic other vibrational effects. Model-based blind and thus unbiased predictions for 6 experimentally uncharacterized *N*-alkylated pyrazoles and imidazoles may allow quantification of the predictive power of the DFT-supported anharmonic model Hamiltonian in a future investigation.

## Graphical abstract

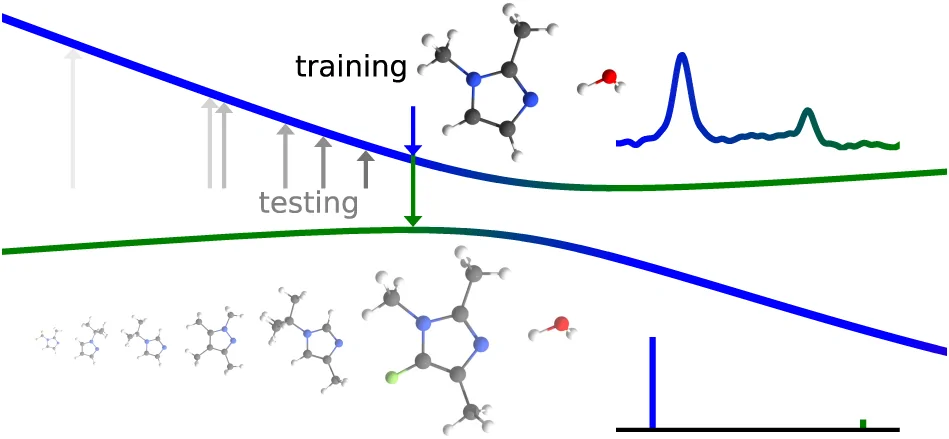

## Introduction

When a single water molecule is attached to organic bases of increasing strength, its hydrogen-bonded OH stretching mode (OHb) is progressively downshifted in energy.^1^ The vibrational dynamics becomes particularly interesting when the downshifted OHb stretch approaches the range of the bending overtone of water (b2), some 500 cm^–1^ or roughly 14% lower in energy than the symmetric OH stretch in the free water monomer. In this situation, anharmonic Fermi resonance^2^ sets in and mixes the character of the two coupled vibrations. The last years have seen a lot of progress toward the understanding of this OH stretch/bend Fermi resonance in water.^3–5^ For the isolated water molecule, it is just a little anharmonic detail, with much less impact on the vibrational dynamics than the kinematic coupling of the two OH bond oscillators or Darling-Dennison resonances for their overtones.^6^ In liquid water, however, the Fermi resonance is fully developed, because cooperative hydrogen bonding brings the stretching fundamentals into close vicinity of the bending overtone,^3^ similar to the historical case of monomeric CO_2_ which was first discussed 95 years ago.^2^ While stretch/bend Fermi resonance in CO_2_ has important consequences for the atmospheric radiation balance of the earth,^7^ the analogous OHb/b2 resonance in liquid water contributes importantly to ultrafast vibrational relaxation and heat transport after local OH stretch excitation.^8^

A natural bridge between the isolated and condensed water extremes is provided by cluster science. Clusters generated in adiabatic supersonic jet expansions^9^ feature spectral simplification and resolution through the removal of internal energy of the complexes.^10^ An increase in hydrogen-bonded cluster size can switch on or off a resonance situation through increased cooperativity.^11^ For plain water clusters, it was shown by a combination of infrared and Raman spectroscopy that the ring-like water pentamer is close to the crossover point for stretch/bend Fermi resonance,^12^ where the spectral sequence of concerted hydrogen-bonded stretching fundamental and the concerted bending overtone swaps. This was recently worked out in more quantitative detail by a gas recycling variant of Raman jet spectroscopy,^4^ which facilitates extended data acquisition and signal deconvolution. Given the importance of water as a solvent for organic molecules, it is of interest to see how the same Fermi resonance is activated in a solvating water molecule as a function of hydrogen bonding to neutral organic solutes and perhaps to other solvating water molecules. This is the topic of the present contribution.

Neutral monohydrates have been explored intensely by vibrational spectroscopy in supersonic jets.^10,13–15^ In most cases, there is little to no evidence for the water stretch/bend Fermi resonance, because a single hydrogen bond interaction is often too weak to tune the OH stretch close to the bending overtone and thus to transfer enough spectral intensity by wave function mixing. When the hydrogen bond becomes strong enough due to cooperativity, such as in aminoalcohol monohydration,^10^ Fermi resonance would be expected to be strong, but surprisingly the b2 bending overtone signal sometimes appears to be weak or at least broad. Partial explanations may be that aminoalcohol monohydrates shift the OH stretch too far beyond the b2 resonance partner or that the coupling strength decreases with further increasing hydrogen bond strength. This unclear situation called for further empirical exploration.

A well-characterized noncooperative class of compounds where monohydrates exhibit strong Fermi resonance in the infrared spectrum with substantial b2 intensity is that of aliphatic (tertiary) amines.^16,17^ The focus on tertiary amines in this context has two reasons: strengthening of the hydrogen bond through inductive/polarization^18^ effects and absence of complicating signatures of NH stretching fundamentals in the same spectral region, but the resonance is present in less substituted amines as well.^19–21^ By varying the aliphatic nitrogen substituents, it was shown that one can tune the hydrogen-bonded water stretching fundamental OHb almost continuously in the region above b2. It turns out that on top of the expected stretch/bend Fermi resonance between the OHb and b2 states, there are characteristic spectral signatures involving ON motion, i.e., stretching of the entire water unit (O) against the amine (N).

Two limiting mechanisms M_i_ in addition to the well established OHb/b2 (stretch/bend) Fermi resonance mechanism (M_F_) are conceivable for this additional ON-related degree of freedom:

- M_FC_ Vibrational Franck–Condon-mediated intensity transfer from the OHb fundamental to OHb + ON combination bands (OHbON, OHbON2, etc.), i.e., spectral intensity redistribution based on wave function overlap along the ON coordinate between the OHb ground and excited state. This is a kind of transition moment modulation in a vibrationally adiabatic picture,^22,23^ which can already occur for two vibrational degrees of freedom. In the adiabatic picture, it does not affect the position of the OHb fundamental but transfers some of its intrinsic intensity to the combination bands.
- M_RP_ Resonance polyads built from OHb, b2, and b2 + ON (b2ON, b2ON2, etc.), i.e., a mix of strong Fermi resonance between OHb and b2 and presumably weaker three- or higher-quantum resonances between OHb and b2ON*n*. The resulting resonance polyad built on b2 may be selectively activated depending on the position of OHb.^11,24^ In contrast to M_FC_, M_RP_ necessarily involves wave function mixing in the OHb excited state. It is thus the character of the excited quantum states rather than of the transitions to them which is mixed. Furthermore, this mechanism involves at least three vibrational degrees of freedom and it directly affects the position of the dominant OHb transition.

In reality, any combination of M_FC_ and M_RP_ mechanisms might be active in a given monohydrate vibrational spectrum. Furthermore, b2ON as the key player of mechanism M_RP_ and OHbON as the key player of mechanism M_FC_ will themselves undergo a Fermi resonance (M_F_) in analogy to bare b2 and OHb, when they come close to each other in wavenumber. This calls for detailed theoretical modeling including Fermi and higher order resonances together with Franck–Condon factors for transition moments, which are far from routine for these hydrogen-bonded prototype systems.^25^ However, an effective M_RP_-only model was quite successful in phenomenologically describing the spectral intensity features of a large number of tertiary amine monohydrates^16,17^ and may thus form a suitable starting point for theoretical modeling. In contrast to monohydrates, amine dihydrates remain incompletely characterized^17^ and understood, in particular the dynamics of the water molecule directly binding to the nitrogen atom. This is also due to spectral overlap with the CH stretching region.

The objective of the present work is to fill important gaps in the characterization of the resonance onset in water molecules bound to nitrogen, by investigating the microhydration of pyrazoles and imidazoles. Pyrazoles are less basic than imidazoles, because the direct contact of the two nitrogen atoms in the ring reduces the electron density at the lone electron pair. Together, pyrazoles and imidazoles cover the spectral water OHb range of successive alkylation of ammonia, with added aromaticity and planarity. The parent compounds have interesting hydrogen bond properties,^11,14,26–30^ but here we remove spectral complications due to NH modes by *N*-methylation.^31^ A welcome side effect of the NH removal is the increased volatility,^32^ which is advantageous for room temperature nozzle expansions. In the tradition of our work on tertiary amines,^16,17^ chemical substitution is used as a tool to estimate generic anharmonic coupling constants for the solvation site. The wave function mixing effects are sufficiently subtle and theoretically unexplored that chemical tuning appears indispensable at this stage, in particular if one aims at an experimental approach with as little bias as possible from theoretical anharmonic models. Figure 1 shows four representative mono- and dihydrate structures out of the 22 species which were experimentally investigated in this work. This serves to introduce the heterocycle and OHb nomenclature, hydration structure and employed substitution patterns. Substitution at all three heterocyclic C–H groups by either methyl groups or halogen atoms is explored (see Table S1 in the Supporting Information). Halogen substitution tends to weaken the hydrogen bond acceptor strength of the diazole, whereas methyl substitution increases it. Structurally, some of these hydrates are already well characterized,^33,34^ allowing us to focus on the OHb dynamics in these species.

**Figure 1:**
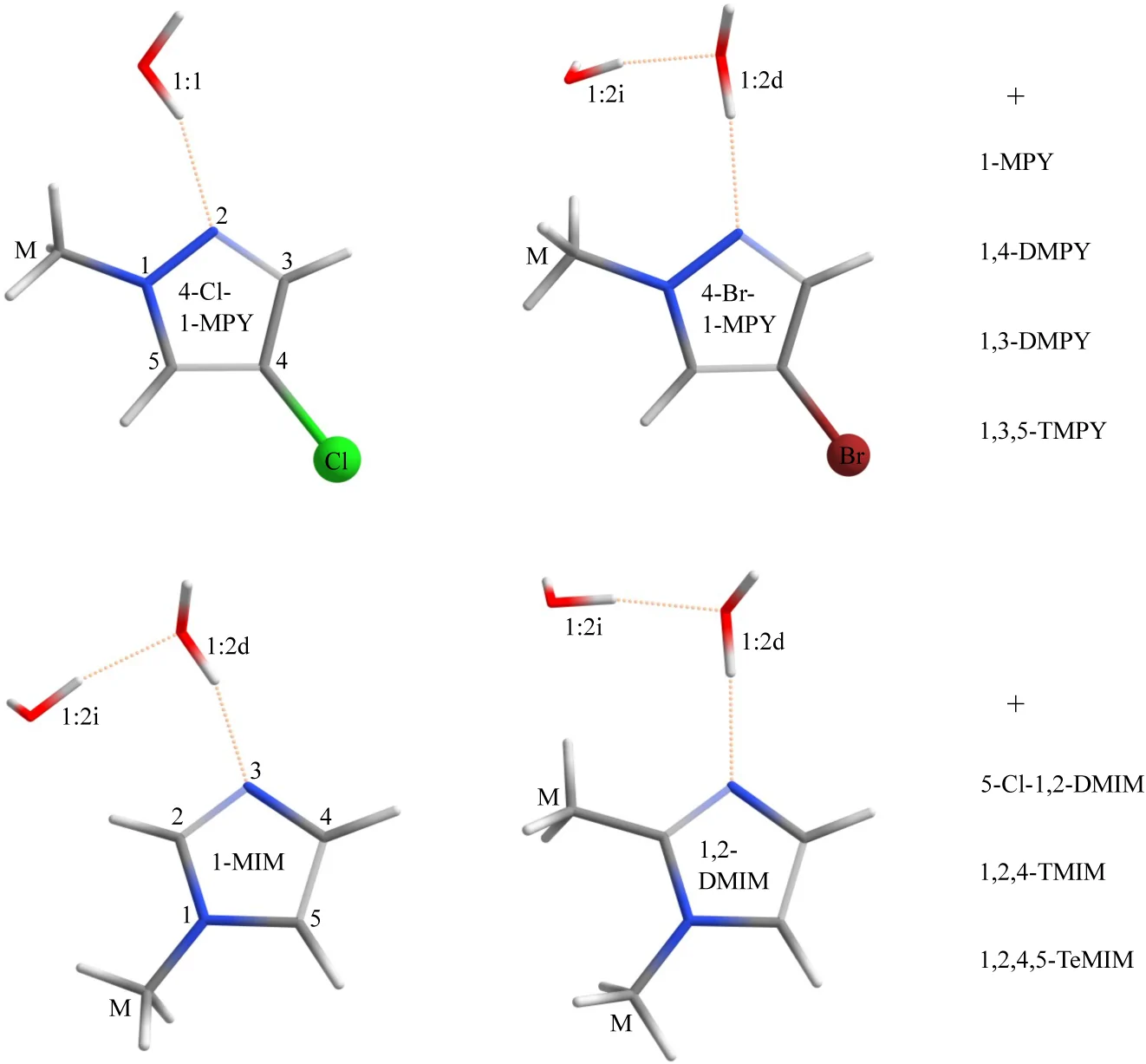
11 pyrazoles (PY) and imidazoles (IM) which are mono- and dihydrated in this work lack an NH group but include halogen substitution (Cl, Br) and up to 4-fold (M, DM, TM, TeM) methylation. The absence of an adjacent (2- or 4-) methyl group in 1-MIM has a strong effect on the directly (d) and indirectly (i) coordinating water units in the (1:1 and) 1:2 complexes (Figures S30–S33, Supporting Information). For the compound acronyms, see also Table S1 in the Supporting Information.

The present work also contributes to the series of HyDRA blind challenges, by supporting the assignment of the particularly challenging test member 1-methyl-4-iodo-1H-pyrazole in the recent HyDRA II competition,^35^ in terms of mono- and dihydration. We demonstrate very similar spectra for the lighter halogens Cl and Br, thus confirming the experimental mono- and dihydrate assignments. The other 9 N-heterocycles further support these assignments and extend the database.

Beyond presenting the experimental raw data which can be used for the training of models for hydrogen bond dynamics, we propose our own phenomenological resonance descriptions, which are remarkably uniform and consistent across the chemical modifications and may serve as a reference point for multidimensional quantum dynamics treatments. They build on simple harmonic density functional theory (DFT) computations, but should also be applicable to perturbative anharmonic models in a similar semiemprical spirit, if the resonances of interest are removed from the perturbative treatment^36^ before reintroducing them empirically.

Finally, we provide forecasts on 6 experimentally still uncharacterized mono- and dihydrates of selected pyrazoles and imidazoles with chemical substitutions which go considerably beyond those investigated in the present work. Some of these compounds were provided to us by another group after the present work had been submitted and prepublished, such that the present predictions are completely blind with respect to the future experimental spectra. The latter are challenging due to the limited vapor pressure of the substituted N-heterocycles. It is not likely that we will succeed in measuring microhydrate spectra for the entire subset made available to us.

## Experimental Section

The gas recycling jet FTIR spectrometer employed in this work (Figure S1 in the Supporting Information) has been described before^37^ and the compound handling is analogous to that for hydrate complexes of tertiary amines.^16^ The compounds and their suppliers are described in the SI (Table S1). For relative band intensities, a stochastic integration program was used.^38^ It is described in Chapter 6 of the SI and provides uncertainties based on noise characteristics, which are important to assess the robustness of the derived intensity centers of resonance polyads. Due to the low volatility of some of the investigated N-heterocycles (Table S2 in the Supporting Information), small temperature variations during measurement can modulate the gas phase partial pressure and water concentration can further vary due to wall desorption and adsorption. An advantage of the FTIR technique is its linearity, which allows to relate signal intensities to concentrations. The disadvantage of not being explicitly cluster size selective can be alleviated by concentration variation, which allows to distinguish 1:1 from 1:2 and 2:1 as well as higher clusters and monomers if the signal-to-noise ratio is sufficient. Difference spectra which eliminate one cluster size can also be helpful (Figures S45 and S47 in the Supporting Information). Switching from the standard carrier gas neon to helium reduces the aggregation tendency and maximizes the 1:1 complex concentration. Overview and detailed spectra for each compound in combination with water are provided in the SI (Figures S2–S29), as are experimental details (Tables S3 and S4 in the Supporting Information).

## Results and Discussion

### Infrared Spectra

The 6 investigated pyrazoles and 5 investigated imidazoles (Figure 1) were mixed with water and a carrier gas (helium or neon) in different mixing ratios and expanded through a pulsed slit nozzle, as in the work on tertiary amines.^16^ The pulsed gas expansion was probed by synchronized FTIR spectroscopy and the gas mixture recompressed to be reused for further pulses.^37^ By varying the relative concentration of the diazole and of the water as well as the stagnation pressure and carrier gas, monohydrate complexes (1:1) can be unambiguously distinguished from dihydrates (1:2) and hemihydrates (2:1) or larger clusters, as long as there is no spectral overlap. Spectral overlap is minimized by the slit jet cooling effect, which removes vibrationally and rotationally hot transitions to a large extent. The remaining spectral width is largely limited by vibrational lifetime effects and (for monomers and perhaps also 1:1 complexes) by the chosen spectral resolution (2 cm^–1^). The recorded spectra are documented in the SI (Figures S2–S29).

A comparison with one representative spectrum of each pyrazole is given in Figure 2 together with a structure formula of the expanded heterocycle. For the present work, up to 4 spectral transitions are relevant. The OHb stretching fundamentals of the single water in the 1:1 complex (to which all spectra in Figure 2 are intensity-normalized) and of the two waters in the 1:2 complex (1:2d directly bound to the pyrazole, 1:2i indirectly bound via the directly bound water, see Figure 1 and Table S7 in the Supporting Information) are clearly visible in all cases. A weak composite signal in the region of the water bending overtone (b2) may be noted for stronger hydrogen bonds. It has potential contributions from all hydrate clusters (even including water homoclusters at higher concentration), largely derived from Fermi resonance with the OHb stretching fundamentals. The spectral sequence 1:2i > 1:1 > 1:2d > b2 is experimentally robust and a consequence of the lower basicity of water and the cooperative nature of the OH···OH···N hydrogen bond chain. The spectral trends observed for 1:1 are straightforward, with electron-withdrawing ring substituents (Cl, Br) increasing the OHb fundamental wavenumber and each additional methyl group decreasing it. In an attenuated form, this is also valid for the indirectly attached water in the dihydrate and in an amplified form for the directly attached water. The b2 signal is hardly discernible at low methylation, but when both α positions of the N acceptor are methylated (1,3-di- and 1,3,5-trimethylpyrazole) its lower wavenumber component intensifies. It is largely due to b2 in dihydrates and the fact that it becomes as intense as the 1:2d OHb signal for the trimethylpyrazole dihydrate is strong evidence for a nearly perfect water stretch/bend Fermi resonance. Thus, we can tune 1:2d OHb all the way from negligible to full Fermi resonance and this provides access to the effective anharmonic coupling constant, if the b2 contributions can be deconvoluted sufficiently well. That is attempted in the SI (Section 4, Tables S20–S24) and the resulting Fermi coupling constant *W*_b_ is about 40 cm^–1^. The magnitude already follows straightforwardly from the spectral separation of the similarly intense peaks in 1,3,5-TMPY (2*W*_b_ = 80(5) cm^–1^). The effective pyrazole-induced coupling constant of about 40 cm^–1^ in dihydrates is also in satisfactory agreement with 46 ± 5 cm^–1^ empirically derived for tertiary amines attached to a single water molecule.^16^ If one applies this coupling constant derived from the multiply methylated pyrazoles to the less downshifted halogenated 1-methylpyrazoles, one can show that a significant b2 intensity contribution would indeed not be expected in their mono- and dihydrates. A remarkably simple dynamical conclusion emerges for the pyrazoles: The monohydrates show essentially unperturbed pure OHb stretching states which scale with substitution effects as expected. The indirectly solvating water in dihydrates behaves similarly, whereas the directly solvated water signal is tuned into Fermi resonance by electron donating substitution. The investigated substitution patterns do not lead beyond this Fermi resonance, though. The latter is reminiscent of the tertiary amine monohydrate case.^16^

**Figure 2:**
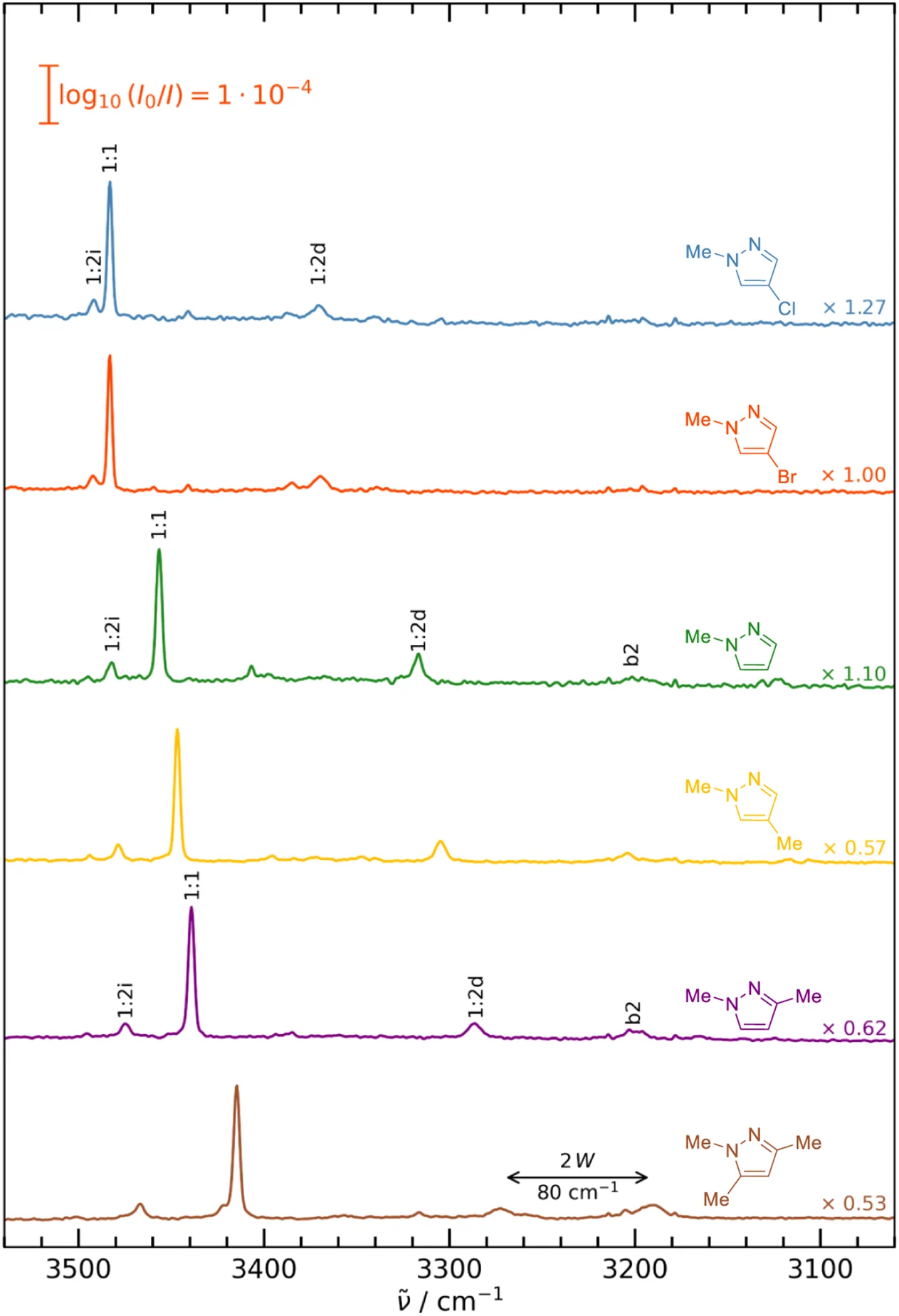
Overview of different substituted pyrazole-water coexpansion FTIR spectra (Table S3 in the Supporting Information) in the water OHb stretching region, scaled (×) to similar monohydrate (1:1) peak intensity. The progressive downshift of the dominant 1:1 feature, the somewhat blueshifted second solvation shell (1:2i) signal and the evolution of the OHb/b2 Fermi resonance for the directly solvated water molecule (1:2d) can be followed from top to bottom. The Fermi coupling constant *W*_b_ is seen to be on the order of 40 cm^–1^. See text for further explanations and Table S8 in the Supporting Information for peak wavenumbers.

This is where the more basic imidazoles can provide further insight. Their 1:2d signals are expected to tune beyond the Fermi resonance with b2, whereas their 1:1 and 1:2i signals should predominantly be downshifted relative to the pyrazoles, tuning slowly into Fermi resonance with b2 from above. As shown in Figure 3, the reality is somewhat more complicated. The 1-MIM spectrum looks very simple, but this is due to spectral overlap of 1:1 and 1:2i. The overlap is promoted by the absence of a methyl group next to the nitrogen docking site. Any b2 contribution from 1:1 and 1:2d is at best very weak. The 5-Cl-1,2-DMIM spectrum resolves the 1:1/1:2i overlap through its methyl group in 2-position and 1:2d is now more visible, but its b2 contribution is either very weak or very broad. This remains true when the Cl substituent is removed and the hydrogen bonds are strengthened. However, the 1:1 complex now has three contributions instead of one. Its b2 contribution can be tentatively identified near 3200 cm^–1^ as a result of the reduction of the energy mismatch between OHb and b2 and the increased extent of Fermi resonance. In between, a new sharp peak can be assigned to the b2ON combination band. This transition is familiar from tertiary amines,^16^ where it invariably was observed higher in wavenumber than OHb. Now, due to the weaker hydrogen bond between the water molecule and the imidazole, it appears to lower wavenumber than OHb itself. Like OHb, it has a somewhat asymmetric band profile, but the intensity pattern is consistent with an anharmonic coupling constant close to 15 cm^–1^. When moving to 1,2,4-TMIM, the 1:1 OHb/b2ON resonance intensifies and there is a small signal of unclear origin (see discussion in Figure S26 in the Supporting Information) in between the resonance doublet which has little influence on the resonance analysis. Very little b2 evidence is found, even for the 1:1 complex. Fully methylated imidazole has no unexplained additional peak near its resonance doublet, a nearly perfect OHb/b2ON resonance, and again no substantial b2 coupling partner for 1:2d. This may be partially explained by the strong downshift of 1:2d, far beyond the expected position of b2, but clearly the evidence for OHb/b2 Fermi resonance is much reduced for the stronger base imidazole than for pyrazole. The details of the resonance analysis are given in Tables S25–S29 in the Supporting Information. The absence of significant b2 intensity in the imidazole dihydrates is also supported by the regular behavior of the 1:2i/1:2d intensity ratio (Figure S44 in the Supporting Information), roughly paralleling harmonic predictions. This is somewhat in agreement with the findings for aminoalcohol monohydrates^10^ which also involve strong cooperative hydrogen bonds.

**Figure 3:**
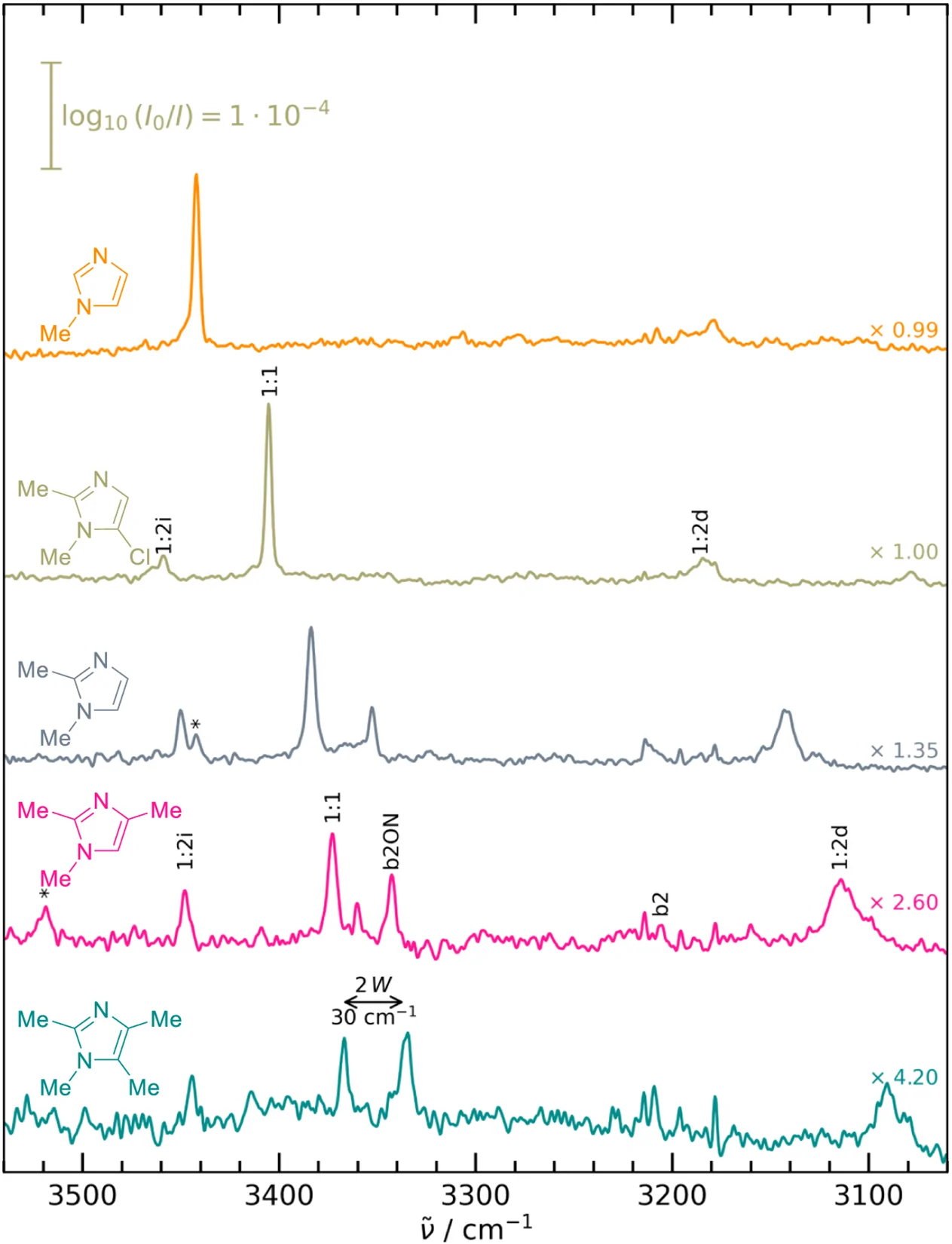
Overview of different substituted imidazole-water coexpansion FTIR spectra (Table S4 in the Supporting Information) in the water OHb stretching region, scaled (×) to similar monohydrate (1:1) peak intensity. The progressive downshift of the dominant 1:1 feature with ring methylation, the evolution of a local OHb/b2ON resonance with a coupling constant *W*_N_ on the order of 15 cm^–1^, the significantly blueshifted second solvation shell (1:2i) signal whenever there is a methyl group in 2-position and an intensifying strongly red-shifted 1:2d signal without substantial b2 Fermi resonance counterpart can be followed from top to bottom. See text for further explanations and Table S8 in the Supporting Information for the peak wavenumbers.

In summary, imidazole monohydrate OHb signals reach the spectral window where a b2ON resonance can be activated, whereas pyrazole monohydrate OHb fundamentals are too high in wavenumber to light up this resonance. Pyrazole dihydrates can fully activate the b2 Fermi resonance by appropriate chemical substitution. Imidazole dihydrates go beyond b2, without showing evidence for this strong resonance.

### Basic Anharmonic Modeling

At this stage, one could turn to anharmonic theory and ask for a detailed explanation of these dynamical differences between pyrazole and imidazole hydrate complexes. However, this is a nontrivial task and while first-principles methods are hopefully being pushed toward this goal, also along the HyDRA blind challenge series,^35^ we will try to offer an interim solution, a simple unified model for the apparently rather different spectral signatures of pyrazole and imidazole hydrates.

We start with a reasonably accurate harmonic DFT prediction. Harmonic force constants are typically somewhat too soft, barriers too low, subtle substitution trends can be unreliable,^39^ but basically the hydrogen-bonded complexes are correctly described, if some kind of London dispersion contribution is added. Resonances are excluded in such a harmonic treatment. One could include them by vibrational perturbation theory,^36^ but they are sensitive to the relative energy of the involved vibrational modes and therefore often need a higher level of electronic structure treatment than DFT. The interim solution consists in extracting information about the size of the underlying effective 2 × 2 coupling constants from experimental spectra and in improving insufficient DFT mode coincidences by empirical wavenumber offsets.

The 1:2i state is simple to treat, because the direct hydrogen bond partner (water) remains the same for all 11 systems, the substitution effects are small, and there are no detected resonances. Therefore, a uniform scaling or shifting model of the type

1 $$\mathrm{\nu ̃}\left(OHb\right)=\omega \left(OHb\right)-c_{2i}$$

might be sufficient, in which a DFT harmonic wavenumber ω is uniformly corrected to obtain the observable anharmonic wavenumber *ν̃*. The shift *c*_2i_ in eq 1 accounts for DFT deficiencies and (mostly diagonal) anharmonicity.

The 1:2d state is more complicated, because it couples to b2 (proven for pyrazoles, not so clear for imidazoles) and one has to make sure that the 2 × 2 diagonalization is carried out with the proper mismatch δ between the zero-order bright state OHb° ≈ ω­(OHb) and the dark perturber b2° ≈ ω­(b2) = 2ω­(b). *ω̅* is the average wavenumber of the bright state and the perturbing dark state, in this specific case (ω­(OHb) + ω­(b2))/2. If *F*[*ω̅*; δ; *W*_b_] stands for the eigenvalue with dominant OHb character in the 2 × 2 (Fermi) resonance problem with off-diagonal effective coupling matrix element *W*_b_ , one can try

$$\mathrm{\nu ̃}\left(OHb\right)=F\left[\left(\omega \left(OHb\right)+\left(\omega \left(b2\right)+s_{2d}\right)\right)/2;,\omega \left(OHb\right)-\left(\omega \left(b2\right)+s_{2d}\right);{,W}_{b}={40\;cm}^{-1}\right]-c_{2d}$$ 2

as a model. Here, *s*_2d_ shifts the harmonic bending overtone such that it matches OHb where it does so in experiment, based on the intensity pattern. *c*_2d_ compensates for this, for DFT errors and for (mostly diagonal) anharmonicity in the coupling modes. The hope is that these two parameters *s*_2d_, *c*_2d_ as well as *W*_b_ are more or less conserved in all 11 pyrazoles and imidazoles and thus transferable.

Because the chromophore of 1:2d is involved in a very strong hydrogen bond with a lot of variation between the least and most electron rich heterocycles, it may be too coarse to assume that the diagonal anharmonicity in OHb remains system-independent, when it is tuned through the more or less stationary b2 dark state. Therefore, an ad hoc refined model 1:2d′

$$\mathrm{\nu ̃}{\left(OHb\right)}^{\prime }=F\left[\mathrm{\omega ̅};\;\left(\omega \left(OHb\right)-{\left(\omega_{M}(OH)-\omega (OHb)\right)}^{2}/\omega_{M}\left(OH\right)\right)-\left(\omega \left(b2\right)+s_{2d}^{\prime }\right);W_{b}^{\prime }={40cm}^{-1}\right]-c_{2d}^{\prime }$$ 3

could be used instead of eq 2. Here, ω_M_ is the harmonic symmetric stretch prediction for isolated water. The more the harmonic prediction is downshifted from ω_M_ due to strong hydrogen bonding, the more anharmonicity is added in eq 3. A 10% (20%) harmonic downshift induces a 1% (4%) anharmonic downshift. In this case, *ω̅* stands for

$$\left(\left(\omega \left(\mathrm{OHb}\right)-{\left(\omega_{M}(\mathrm{OH})-\omega (\mathrm{OHb})\right)}^{2}/\omega_{M}\left(\mathrm{OH}\right)\right)+\left(\omega \left(b2\right)+s_{2d}^{\prime }\right)\right)/2$$

We do not know whether this purely empirical adjustment obtained by trial and error can be justified by anharmonicity, by DFT deficiencies or not at all.

The most elaborate model is required for the OHb wavenumber in the 1:1 complex, because it undergoes anharmonic resonance with b2ON (for imidazoles) and potentially also with b2 (with weak evidence at least for pyrazoles). Therefore, two nested 2 × 2 problems have to be solved (as a shortcut to a 3 × 3 diagonalization):

$$\mathrm{\nu ̃}\left(OHb\right)=F\left[{\mathrm{\omega ̅}}_{N};\left(F\left[{\mathrm{\omega ̅}}_{b};\omega \left(OHb\right)-\left(\omega \left(b2\right)+s_{1b}\right);W_{b}={40\;cm}^{-1}\right]-c_{1b}\right)-\left(\omega \left(b2ON\right)+s_{1N}\right);W_{N}={15\;cm}^{-1}\right]$$ 4

Again, *F*[*ω̅*; δ; *W*] (in cm^–1^) stands for the eigenvalue with dominant OHb character in the 2 × 2 resonance problem with off-diagonal effective coupling matrix element *W*, average wavenumber *ω̅* of the two interacting states, and their wavenumber mismatch δ. In eq 4, *s*_1b_ can be used to adjust the harmonic DFT predictions for OHb and b2 so that their gap corresponds to the (diagonal) anharmonic one, but the two states are too distant to fix *s*_1b_ and we typically set it to 0. However, the OHb state deperturbed for Fermi-resonance needs to be adjusted such that it matches the OHb° centroid of the OHb/b2ON doublet, which is achieved by *c*_1b_. Thus, *c*_1b_ accounts for DFT errors and (diagonal) anharmonicity of the OHb stretching state. Then one can apply the weaker resonance with b2ON, after shifting the b2ON harmonic DFT prediction by *s*_1N_ such that it generates the experimental doublet, where this is observed. *c*_1b_ and *s*_1N_ (which accounts for anharmonicity and DFT error in b2ON) are linearly dependent, but we introduce both to have a realistic interim OHb° value at hand. See also Section 8 in the SI for more details.

### Harmonic Predictions as Input

So far, this work has not made use of quantitative DFT results. The assignments are based on experimental parameter variations, assisted by the robustly predicted hydrogen bond topology. To make use of the anharmonic models proposed in the previous section, we have applied four electronic structure computational levels including harmonic analysis to all investigated mono- and dihydrates, using the ORCA^40^ program suite (version 5.0.3, see Table S9 in the Supporting Information for a sample input). Three are based on the D3-corrected B3LYP hybrid functional^41^ with the basis sets def2-TZVP (TZ), ma-def2-TZVP (maTZ) and def2-QZVP (QZ), the fourth is a double hybrid prediction using D3-corrected B2PLYP^42^ with the def2-QZVP basis (B2QZ). The harmonic wavenumbers relevant for the anharmonic models are compiled in the SI (Tables S11–S18), together with details of the computations. Experimentally, there is no indication for structural isomers. Some of their theoretical predictions are presented in Table S10 and Figure S34 in the Supporting Information and for most of them it appears plausible that they relax during the supersonic jet expansion. The limited signal-to-noise ratio does not allow to rule out a small population of such metastable isomers in the presented spectra, but the regular behavior across the 11 diazoles renders interference with the major resonance band assignments unlikely.

The harmonic predictions themselves are of limited usefulness in the direct comparison to experiment. This is illustrated by Figure 4, which compares the predicted harmonic wavenumber splitting between the 1:2i transition and the 1:1 transition (blue) with the experimental (anharmonic) splitting as a function of (locally deperturbed) experimental 1:1 wavenumber for the B2QZ approach (red).

**Figure 4:**
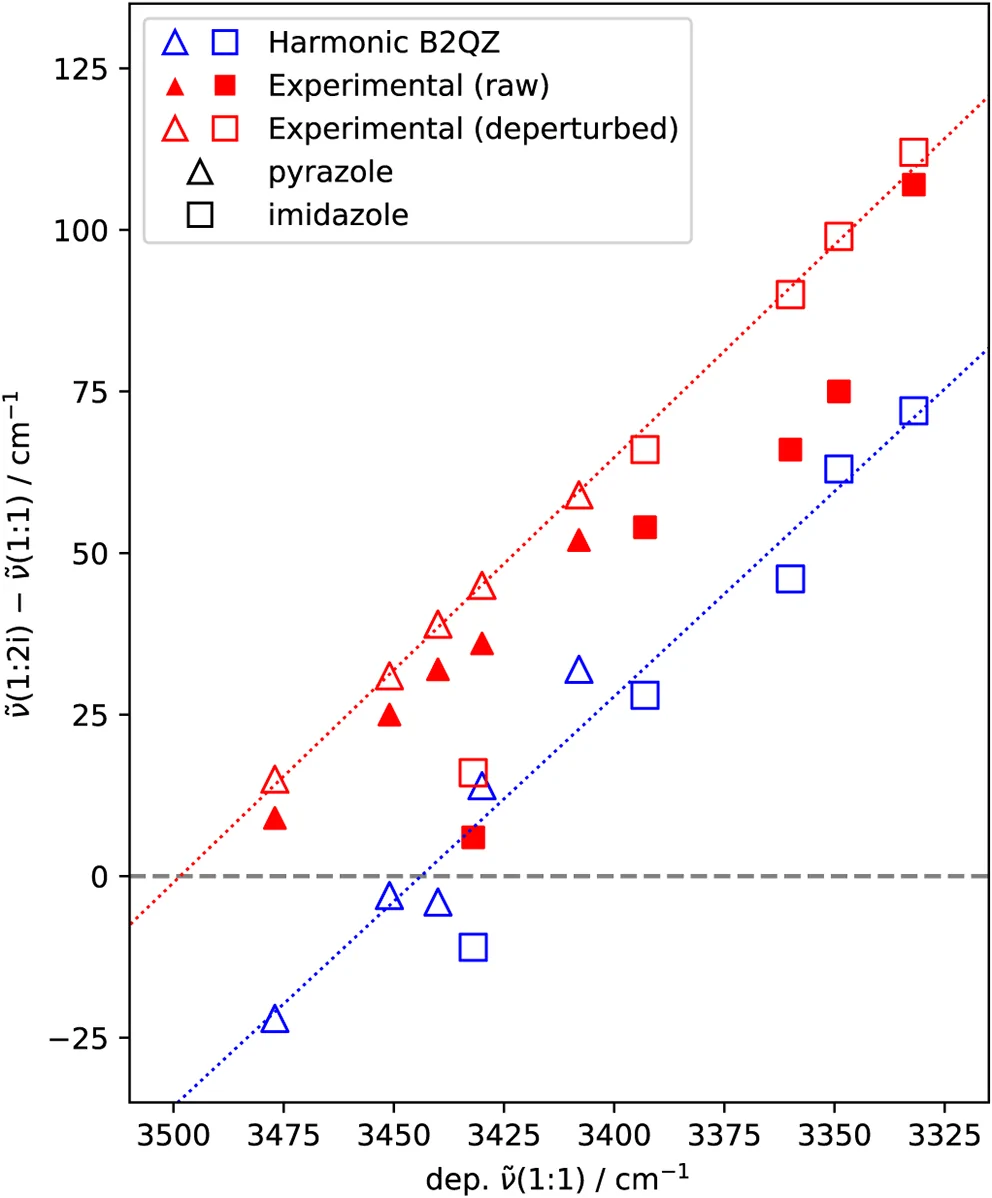
Correlation of the wavenumber gap between the 1:2i transition and the 1:1 transition (OHb, deperturbed for the b2ON resonance) with the deperturbed experimental 1:1 transition wavenumber.

Experimentally, all pyrazoles and imidazoles except for 1-MIM (which lacks a supporting CH_3_ group for the second water molecule, see Figure 1) show a reasonably linear correlation (red unfilled symbols and regression line with a slope of −0.66). The experimental 1:1 and 1:2i values are taken from Tables S24 and S29 (global deperturbation). Without resonance deperturbation (filled red symbols), the correlation would be less linear and less uniform between pyrazoles and imidazoles.

For the harmonic B2QZ predictions, the correlation is somewhat less strong than for experiment (blue unfilled symbols and regression with similar slope of −0.63). Nevertheless, a systematically offset gap is predicted, i.e., 1:2i is substantially downshifted relative to 1:1. As a consequence, most pyrazole 1:2i transitions are harmonically predicted to fall below the 1:1 transitions (below the horizontal black dashed line), which is never the case in experiment. Clearly this is a qualitative disagreement between theory and experiment which could be due to deficiencies in the functional or in the harmonic approximation. The other functionals explored in this work show no improvement at the harmonic level. Likely, the mono- and the dihydrate involve different anharmonicities, besides any DFT and basis set size deficiencies. On the other hand, the harmonic and experimental correlations are reasonably parallel, suggesting that trends are captured and that B2QZ provides a good starting point for the simple anharmonic model proposed above. This is also the case for the experimentally observed level coincidence between OHb and b2ON in the monohydrates (Figure S43 in the Supporting Information). Harmonically, one has to shift b2ON relative to OHb to create the monohydrate level coincidence for the compounds which show it experimentally. This shift is smallest for the B2QZ level (Figure S43 in the Supporting Information). For dihydrates, the corresponding shift for the OHb/b2 level coincidence is largest at B2QZ level (Figure S46 in the Supporting Information). All this suggests that the harmonic predictions can be very useful after experimentally inspired shifting and adequate resonance correction.

Based on the TZ and B2QZ harmonic predictions, the parameters *c*_*i*_, *s*_*i*_ were adjusted in steps of 5 cm^–1^ to find a satisfactory match between the observed OHb transitions and the model predictions. The idea is not to obtain a perfect fit, but rather a semiquantitative uniform treatment of pyrazoles and imidazoles. For TZ, the model parameters are (*s*_1b_, *c*_1b_, *s*_1N_)/cm^–1^ = (0, 85, −125), *c*_2i_ = 35 cm^–1^ and (*s*_2d_, *c*_2d_)/cm^–1^ = (0, 50). For B2QZ, they are (*s*_1b_, *c*_1b_, *s*_1N_)/cm^–1^ = (0, 145, −165), *c*_2i_ = 110 cm^–1^ and (*s*_2d_, *c*_2d_)/cm^–1^ = (25, 130), alternatively $\left(s_{2d}^{\prime },c_{2d}^{\prime }\right)/{\mathrm{cm}}^{-1}=\left(-30,85\right)$ . The performance of the models is analyzed in Tables S31–S33 in the Supporting Information. The resulting RMSD (root-mean-square-deviation) values for the investigated diazoles are summarized in Table 1.

**Table 1:** RMSD Values in cm^–1^ for Different Transitions from Experiment, Different Unifying Models and Different Subsets of Compounds^a^

Transition	Compounds	TZ	B2QZ	B2QZ′ (eq 3)
1:1 (eq 4)	6 PY + 5 IM	3.5	3.2	
	6 PY	1.7	1.4	
	5 IM	4.8	4.4	
1:2i (eq 1)	6 PY + 5 IM	2.4	2.4	
1:2d(′) (eqs 2, 3)	6 PY + 5 IM	14.4	19.0	4.1
	6 PY	8.7	9.0	3.4
	5 IM	19.1	26.5	4.7

a The best model B2QZ′ shows average deviations of the OHb stretching wavenumbers by less than 5 cm^–1^. This is about as good as such an empirical model, the wavenumber parameters of which were varied in steps of 5 cm^–1^, can be.

Figure 5 illustrates the performance of the most refined B2QZ′ model. The dominant outlier is 1-MIM, the only system where the water molecule can have no secondary interaction with an adjacent methyl group. This either indicates a weakness in the underlying DFT model to describe such a substitution trend with weak cooperative CH···O interactions^39^ or else a change in anharmonicity due to such weak cooperative interactions. Analogous binding situations e.g., to pyrimidine^43^ may help to further elucidate this aspect. Otherwise, the model performance is very satisfactory. It describes the substitution trends in band positions, it predicts dominant and shared intensities among the 6 depicted transitions of the model (four narrow 1:1, 1:2i, b2ON(1:1) and b2(1:1) transitions, two broader 1:2d, b2­(1:2d) transitions) and perhaps most importantly it explains where transitions are too weak to be observable at the available signal-to-noise ratio.

**Figure 5:**
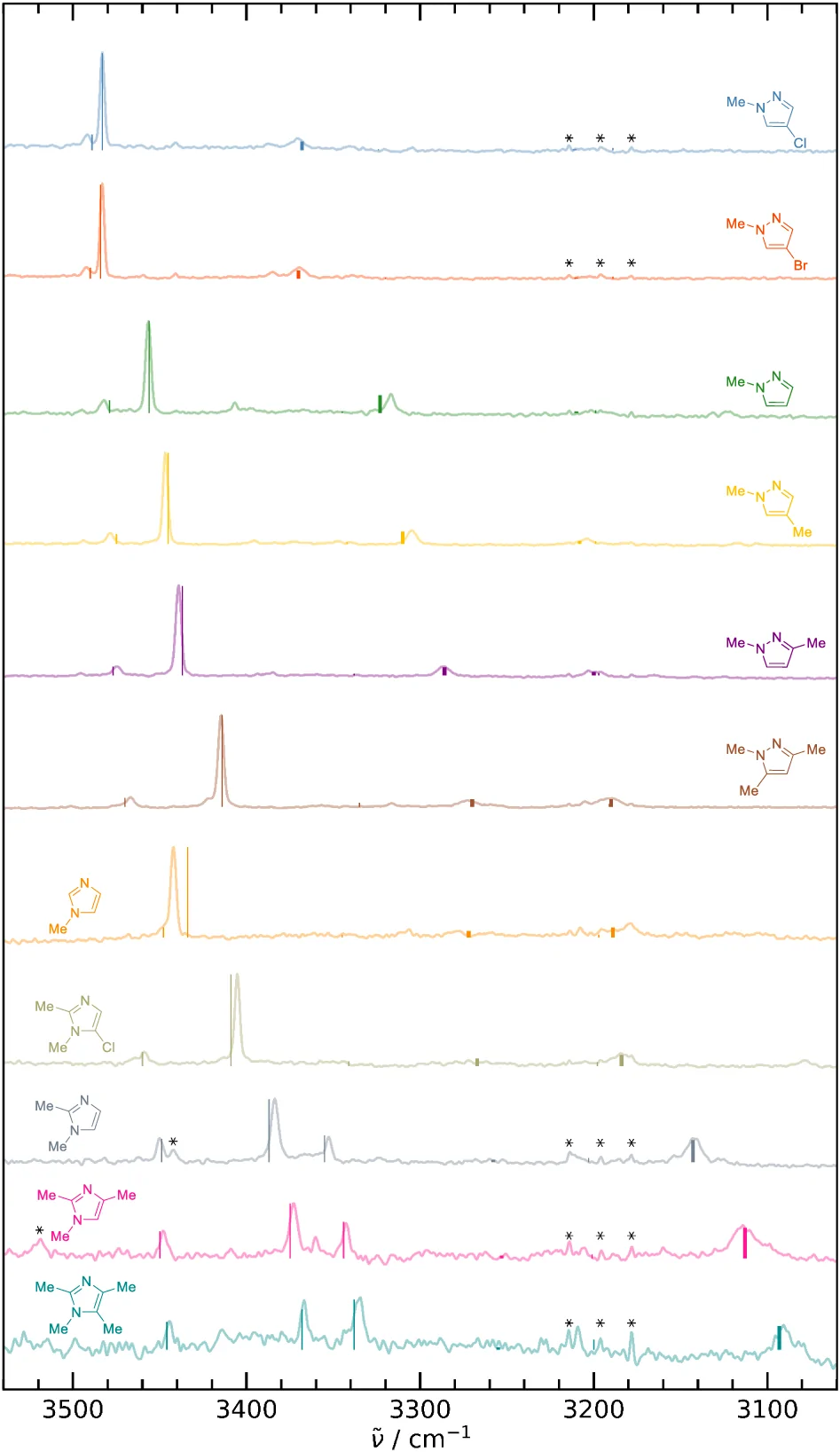
Wavenumber and relative intensity predictions (sticks) based on the refined B2QZ′ model superimposed on experimental spectra of 11 pyrazole/imidazole coexpansions with water. For the analogous TZ model performance, see Figure S50 in the Supporting Information. The total intensity attributed to 1:1 is kept constant across the systems. 1:2d signals and their resonance partners (with their intensity ratio derived from the model) semiquantitatively encode the larger spectral width of the directly bound oscillator in a 3× larger thickness of the sticks. The 1:2i intensity is independently matched to experiment, but the ratio to the combined 1:2d and resonance intensities is in reasonable agreement with harmonic predictions (Figure S44 in the Supporting Information). Impurities and weak water monomer b2 transitions are marked with stars (*).

By exploiting approximate linear relationships between harmonic OHb and b2 as well as b2ON predictions (Figures S35–S42 in the Supporting Information), one can transform the nested 1:1 anharmonic model into an approximate univariate model. Any calculated B2QZ harmonic monohydrate OHb stretching wavenumber will then yield an approximate triplet of anharmonically coupled OHb/b2/b2ON wavenumbers. The evolution of this resonance triad with harmonic B2QZ OHb wavenumber is depicted in Figure 6. One can see how the anharmonic OHb wavenumber (blue, negative slope) avoids crossing two perturbers (green and red, with slightly positive slope) and there is no chance to describe these avoided crossings accurately by a uniform scaling factor of the harmonic input value. The raw experimental positions are predicted quite well by the anharmonic B2QZ model. The main outlier is 1-methylimidazole, the only tested compound which has no methyl group in α-position available to support the solvating water molecule. The avoided crossing between OHb (blue) and the local resonance partner b2ON (green) is probed by three imidazoles. If the water were bound more strongly, OHb would further downshift and intensify the Fermi resonance with b2 (red), so far only observed as trace signals. The small kinks in the curves at low wavenumber are a consequence of the simplified nested 2 × 2 treatment instead of a consistent 3 × 3 diagonalization, with effects far smaller than remaining extrapolation uncertainties. Specifically, even for a strongly coupled OHb/b2ON case such as 1,2,4-TMIM, the error is still less than 1 cm^–1^. The reason is that b2ON and b2 are separated by the dimer stretching mode ON, which has a relatively large wavenumber due to the lightness of the solvating water molecule. This invites the approximate nested procedure, where OHb is first coupled to b2, and the relevant coupled state with highest OHb character is subsequently mixed with b2ON.

**Figure 6:**
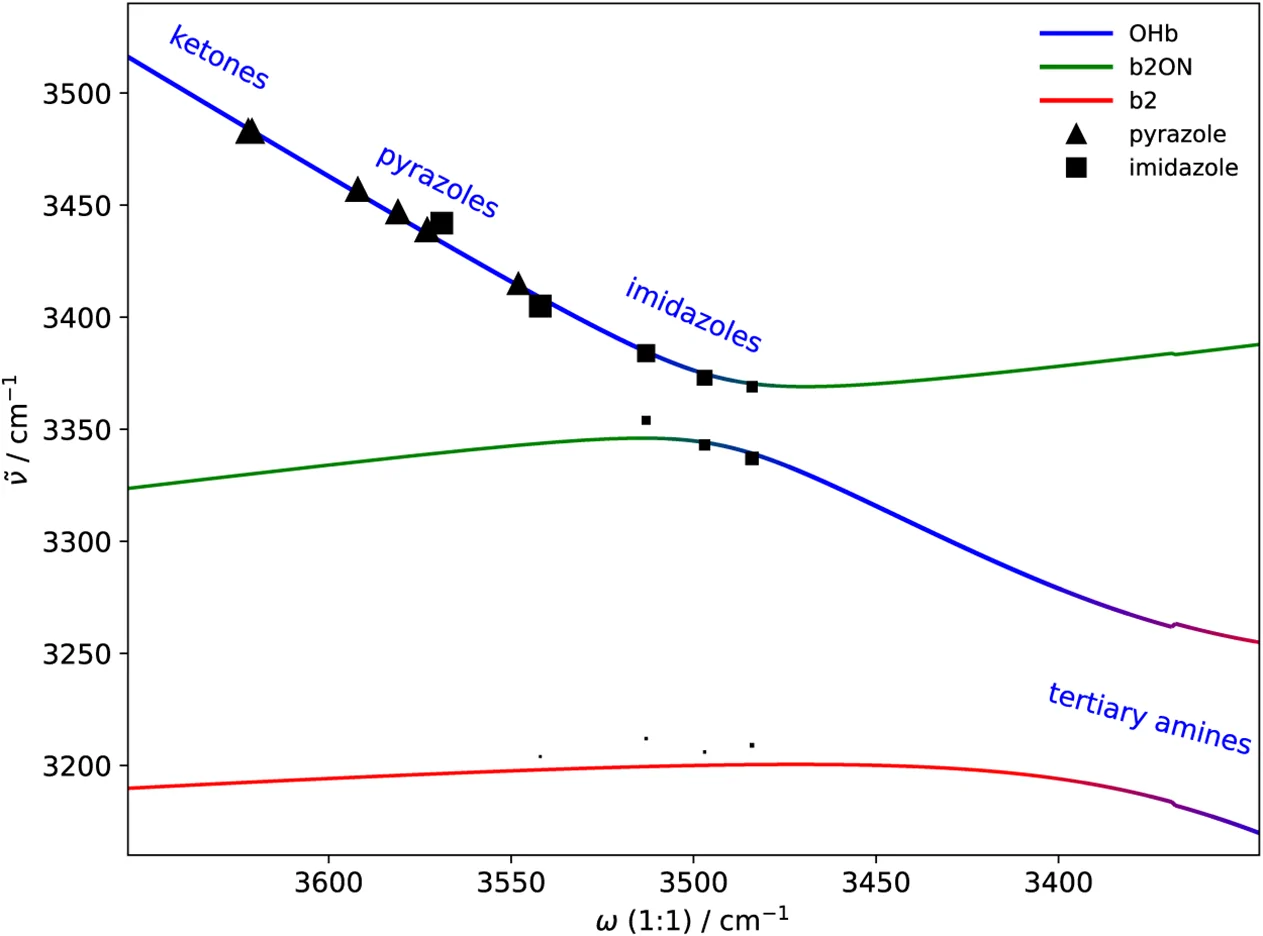
Anharmonic B2QZ model for the OHb/b2ON/b2 triad *ν̃* in diazole monohydrates as a function of harmonic B2QZ OHb wavenumber ω with linear harmonic correlations for b2ON (green) and b2 (red). One can see how from left to right the dropping OHb wavenumber (blue) is interrupted by two resonances. This avoided crossing effect cannot be captured by a simple scaled harmonic approach but instead requires previous anharmonic deperturbation on the experimental side. Also shown are experimentally observed sufficiently intense raw 1:1 transitions (black, predicted intensity encoded into symbol size), to which the model has been coarsely adjusted. The extrapolation into the OHb/b2 Fermi resonance on the right uses the same coupling matrix element as derived for diazole dihydrates.

Despite this success in unifying the apparently different spectral fingerprints of the 22 microhydrates of *N*-methylated pyrazoles and imidazoles, one should always consider the possibility that the success is fortuitous, given the simple coupling assumptions and the empirically adjusted parameters. Pending rigorous multidimensional ab initio calculations, the best way to find out is to organize a blind challenge on further systems, which deviate from the narrow substitution patterns explored in the present work. In contrast to the overarching multiparticipant HyDRA double-blind challenge, we explore a faster local variant, where spectral predictions are made in this manuscript, including commercially unavailable pyrazole and imidazole derivatives. Experiments on these test species (which will in part be synthesized by another group in the research training group BENCh) only start once the present manuscript is prepublished. The results of this single-blind test will be published separately, to the extent that the synthesis is successful and the vapor pressure of the synthesized compounds is sufficiently high for our instruments.

### Blind Spectral Predictions

We identified four imidazoles and two pyrazoles which have not been investigated spectroscopically before and which are hoped to be sufficiently volatile for a jet FTIR investigation in combination with water to reveal their mono- and dihydrate OHb stretching vibrations. They are tetramethylpyrazole, 1-*tert*-butylpyrazole, 1-*tert*-butylimidazole, 1-*tert*-butyl-4-methylimidazole, 2-methyl­(-1-trifluoromethyl)-1H-imidazole and 5-fluoro-1,2,4-trimethylimidazole (see Table S34 in the Supporting Information). Based on the anharmonic models and harmonic DFT results for their hydrate complexes (Tables S35–S44 in the Supporting Information) and prior to experimental investigation, we can make unbiased predictions to demonstrate the accuracy and limitations of the anharmonic models which were trained on 11 pyrazoles and imidazoles, none of them involving a *tert*-butyl group or fluorine atoms. Therefore, only one out of the six test candidates can be regarded as a closely trained system.

Figure 7 shows the structural formulas of the six test systems, together with the best predictions for the three OH stretching transitions of their mono- and dihydrates (including the purity *P* of the two transitions which are potentially involved in resonances). These predictions build on the three anharmonic models and on deviations of these models from two individually selected training systems (one directly above and one directly below the predicted harmonic 1:1 transition, Tables S43–S44 in the Supporting Information). They are given as multiples of 5 cm^–1^ due to the remaining uncertainties and the approximate nature of the models as well as the lack of explicit training for 5 out of the 6 systems. Estimated uncertainties are given in parentheses in steps of 5 cm^–1^. Particularly interesting is the dihydrate of 1-TFM-2-MIM, where the directly attached water OHb is predicted to spectrally switch from below b2 to above b2 due to the fluorination of the methyl group, when comparing with the dihydrate of 1,2-DMIM. Partial fluorination may also be of interest in this context.^44^

**Figure 7:**
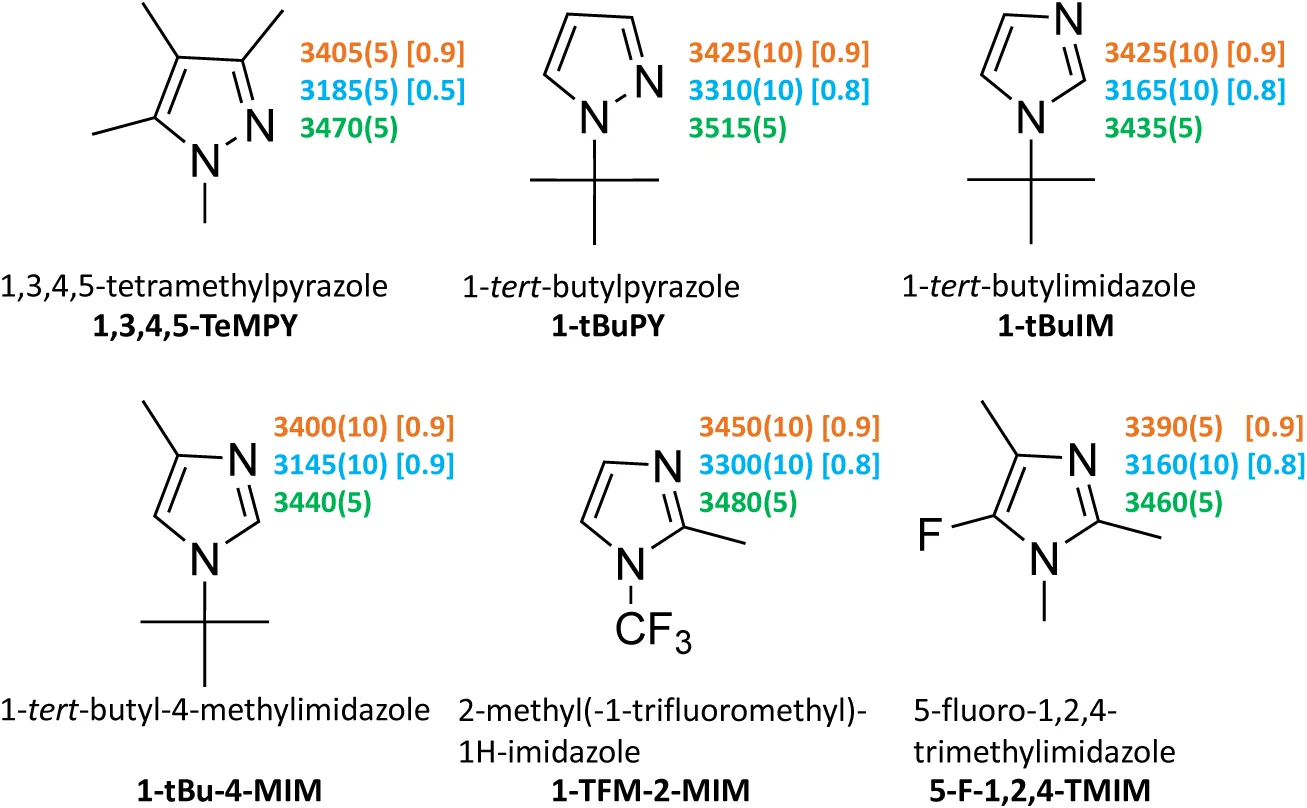
Two *N*-alkylated pyrazoles and four *N*-alkylated imidazoles which we propose for a spectroscopic blind test of the anharmonic coupling models for mono- and dihydrates developed in this work. Most of them are relatively far from the 11 training members, by newly introducing *tert*-butyl groups, −F and −CF_3_ substituents, while the first one is a direct extension from 1,3,5-TMPY. The predicted 1:1 (orange, top), 1:2d (blue, middle) and 1:2i (green, bottom) OHb wavenumbers (in cm^–1^, with estimated uncertainty in parentheses) are provided next to the structure formula together with the predicted OHb purity [*P*] of the dominant transition in the former two categories. For computed structures of the mono- and dihydrates, see Figures S48–S49 in the Supporting Information.

In the SI, we also provide purely harmonic BEsT guesses^35^ for the six test systems (Tables S43–S44), based on interpolation between the closest training system spectrally above and below for the 1:1 complex. These harmonic predictions are in good agreement with the anharmonic model forecasts whenever there is no resonance passage involved. We also show that the model reproduces the literature-known transitions of the parent imidazole^14^ and the HyDRA II test member 1-methyl-4-iodo-pyrazole^35^ within 5 cm^–1^.

## Conclusions

This work presents 33 additional entries for the HyDRA database^45^ (Table S30 in the Supporting Information) of currently 70 mono- and dihydrate OHb stretching vibrations. Nine of the entries involve at least one experimentally explicit anharmonic resonance, in 8 cases at least one resonance can be implied by analogy, for the other 16 OHb transitions there is only a single experimental peak. This turns the underlying pyrazoles and imidazoles into suitable targets for microhydration research. Their mix of cooperative and isolated hydrogen bonding and the systematic tuning through anharmonic resonances provide further aspects and training for future blind challenges with the purpose of quantum-chemical benchmarking. They fill a gap between ketones (left end of Figure 6) and tertiary amines (right end of Figure 6) as acceptors toward the first water unit. Resonances are an excellent tool to disentangle electronic structure errors^46^ from nuclear-dynamics errors,^47^ because they prevent systematic error cancellation. Although the spectral fingerprints for pyrazole and imidazole as hydrogen bond acceptors appear to differ widely, simple effective^48^ Hamiltonian modeling suggests common resonance patterns for both diazoles as hydrogen bonding partners for one and two water molecules. While harmonic DFT predictions fail in describing the spectral evolution with chemical substitution, they can be augmented by low-dimensional anharmonic couplings to achieve near-quantitative performance for the training systems based on 11 different pyrazoles and imidazoles.

The spectra of six other pyrazole and imidazole hydrate complexes are predicted by the resonance model for the purpose of future experimental model testing, if synthesis is successful and the vapor pressure is high enough. Stretch/bend Fermi resonance with a coupling strength of ≈40 cm^–1^ provides a major coupling pathway^49^ for OH-stretching excitation, which is further modulated by donor–acceptor stretching motion, if the OHb stretch falls close to 3350 cm^–1^. This local resonance with a coupling strength of ≈15 cm^–1^ occurs about midway between the well-known pure bending resonance (≈3200 cm^–1^) and the recently discovered bend-librational resonance^15^ (≈3500 cm^–1^). It helps to ensure that water OHb stretch energy in all but the weakest hydrogen bonds efficiently finds its way out of the local hydride oscillator.

It is remarkable that a basic resonance model coupling OHb and b2 + ON (intensity redistribution mechanism M_RP_) can explain the spectra very well, without having to invoke additional vibrational Franck–Condon transitions, i.e., combination transitions involving OHb and ON (intensity redistribution mechanism M_FC_). Such adiabatically explainable transitions should be favored by the typical modulation of hydrogen bond strength with OHb excitation and if present, they would also be expected to undergo strong Fermi resonance with the corresponding observed b2 + ON combination transitions. Rigorous calculations will have to show why the resonance polyad model alone is so successful, without any visible contributions from Franck–Condon activity. The latter may be hidden within the empirically derived effective anharmonic coupling constants. In this sense, microhydration of pyrazoles and imidazoles also invites theory on a conceptual level, rather than only as a practical benchmark for the prediction of observed water OHb stretching spectra.

## Supplementary Material



## Notes

It contains information on the investigated compounds, measurement conditions and FTIR jet spectra, harmonic DFT calculations, anharmonic deperturbation approaches, and resonance models. The original spectra (Tables S5–S6 in the SI) are available in a GRO.data repository under 10.25625/FZIVF4 (PDF)
