# Supplementary material for: Microhydration of Methylated Pyrazoles and Imidazoles: Robust Vibrational Resonances in Red-Shifted Water
Source: J Phys Chem A. 2026 Oct 27;130(44):8794–805. doi: 10.1021/acs.jpca.6c04895 (PMC13746806; doi:10.1021/acs.jpca.6c04895)
Supplement: Supplementary file 1 [file jp6c04895_si_001.pdf]

# Microhydration of Methylated Pyrazoles and Imidazoles: Robust Vibrational Resonances in Red-shifted Water

Finn Knüppe, Eaindra Lwin, Martin A. Suhm*

*Institute of Physical Chemistry, University of Göttingen, Tammannstr. 6, 37077 Göttingen (Germany). E-mail: msuhm@gwdg.de*

## Contents

**1 Experiment** **S4**
1.1 Investigated compounds . . . S4
1.2 Experimental details . . . S5

**2 Spectral assignments** **S9**
2.1 Nomenclature . . . S9
2.2 4-Chloro-1-methylpyrazole (4-Cl-1-MPY) . . . S11
2.3 4-Bromo-1-methylpyrazole (4-Br-1-MPY) . . . S14
2.4 1-Methylpyrazole (1-MPY) . . . S17
2.5 1,4-Dimethylpyrazole (1,4-DMPY) . . . S19
2.6 1,3-Dimethylpyrazole (1,3-DMPY) . . . S22
2.7 1,3,5-Trimethylpyrazole (1,3,5-TMPY) . . . S25
2.8 1-Methylimidazole (1-MIM) . . . S27
2.9 5-Chloro-1,2-dimethylimidazole (5-Cl-1,2-DMIM) . . . S30
2.10 1,2-Dimethylimidazole (1,2-DMIM) . . . S32
2.11 1,2,4-Trimethylimidazole (1,2,4-TMIM) . . . S35
2.12 1,2,4,5-Tetramethylimidazole (1,2,4,5-TeMIM) . . . S37
2.13 Summary . . . S39

**3 Harmonic modeling** **S39**
3.1 Details on harmonic calculations . . . S39
3.2 Example input . . . S39
3.3 Minimum structures . . . S41
3.3.1 1:1 complexes . . . S41
3.3.2 1:2 complexes . . . S43
3.4 Harmonic predictions . . . S46
3.4.1 1:1 complexes . . . S46
3.4.2 Harmonic prediction performance for the b2ON resonance . . . S46
3.4.3 1:2 complexes . . . S52

**4 Size decomposition and estimates of resonance contributions in methylated pyrazole mono- and dihydrate complexes** **S55**
4.1 Treatment of the b2 resonance in pyrazole hydrates . . . S55

**5 Size decomposition and estimates of resonance contributions in methylated imidazole mono- and dihydrate complexes** **S59**
5.1 Treatment of the b2ON resonance in imidazole monohydrates . . . S59
5.2 Treatment of the b2 resonance in imidazole monohydrates . . . S60

**6 Integration methods** **S61**

**7 Summary of deperturbation strategies** **S62**

**8 A simple unified pyrazole/imidazole anharmonic model based on harmonic predictions** **S64**
8.1 TZ model variant . . . S64
8.2 B2QZ model variant . . . S66
8.3 Improving the 1:2d performance (B2QZ') . . . S66
8.4 Testing the models with 6 new N-heterocycles . . . S69

References S77

# List of Tables

S1 Investigated compounds S4
S2 Volatility of the N-heterocycles S4
S3 Experimental details for the FTIR spectra of pyrazole hydrate complexes S6
S4 Experimental details for the FTIR spectra of imidazole hydrate complexes S7
S5 Pyrazole hydrate spectra sources S8
S6 Imidazole hydrate spectra sources S9
S7 Nomenclature S10
S8 Experimentally observed wavenumbers S39
S9 Example input S40
S10 Lowest predicted isomers of the dihydrate global minimum structures S44
S11 Harmonic predictions for 1:1 OHb S46
S12 Harmonic predictions for 1:1 b2 S47
S13 Harmonic predictions for 1:1 b2ON S50
S14 Harmonic predictions for 1:2d OHb S52
S15 Harmonic predictions for 1:2i OHb S52
S16 Harmonic predictions for 1:2 b2 (in-phase) S53
S17 Harmonic predictions for 1:2 b2 (out-of-phase) S53
S18 Harmonic predictions for 1:2 b2 (average) S54
S19 Pyrazole 1:2d deperturbation from b2 S56
S20 Extrapolated pyrazole 1:2d deperturbation from b2 S56
S21 Pyrazole 1:1 deperturbation from b2 based on He expansions S58
S22 Alternative pyrazole 1:1 deperturbation from b2 based on subtracted 1:2d contributions S58
S23 Extrapolated pyrazole 1:1 deperturbation from b2 S58
S24 Summary of pyrazole mono- and dihydrate complex deperturbations from b2 S59
S25 Imidazole 1:1 complex deperturbation from b2ON S60
S26 Extrapolated imidazole 1:1 complex deperturbation from b2ON S61
S27 Imidazole 1:1 complex deperturbation from b2 S61
S28 Extrapolated imidazole 1:1 complex deperturbation from b2 S61
S29 Summary of imidazole mono- and dihydrate complex deperturbations from b2 S62
S30 Suggested HyDRA database entries for pyrazole and imidazole hydrate complexes S63
S31 TZ-based Fermi model predictions S65
S32 B2QZ-based Fermi model predictions S67
S33 Improved 1:2d' Fermi model predictions S68
S34 Proposed test set S69
S35 Harmonic predictions for 1:1 OHb of the six new compounds S69
S36 Harmonic predictions for b2 (1:1) of the six new compounds S69
S37 Harmonic predictions for b2ON (1:1) of the six new compounds S70
S38 Harmonic predictions for 1:2d OHb of the six new compounds S71
S39 Harmonic predictions for 1:2i OHb of the six new compounds S71
S40 Harmonic predictions for 1:2 b2 (in-phase) of the six new compounds S72
S41 Harmonic predictions for 1:2 b2 (out-of-phase) of the six new compounds S72
S42 Harmonic predictions for 1:2 b2 (average) of the six new compounds S72
S43 Fermi model predictions B2QZ S73
S44 Fermi model predictions TZ S74

# List of Figures

S1 Figure of the FTIR set up S5
S2 4-Cl-1-MPY jet FTIR spectra with different concentrations of water S11
S3 4-Cl-1-MPY jet FTIR spectra with water comparing He and Ne carrier gas S12
S4 4-Cl-1-MPY comparison including monomer transitions S13
S5 4-Br-1-MPY jet FTIR spectra with different concentrations of water S14
S6 4-Br-1-MPY jet FTIR spectra with water comparing He and Ne carrier gas S15
S7 4-Br-1-MPY comparison including monomer transitions S16
S8 1-MPY jet FTIR spectra with different concentrations of water S17
S9 1-MPY comparison including monomer transitions S18

S10 1,4-DMPY jet FTIR spectra with different concentrations of water . . . . . S19
S11 1,4-DMPY jet FTIR spectra with water comparing He and Ne carrier gas . . . . . S20
S12 1,4-DMPY comparison including monomer transitions . . . . . S21
S13 1,3-DMPY jet FTIR spectra with different concentrations of water . . . . . S22
S14 1,3-DMPY jet FTIR spectra with water comparing He and Ne carrier gas . . . . . S23
S15 1,3-DMPY comparison including monomer transitions . . . . . S24
S16 1,3,5-TMPY jet FTIR spectra with different concentrations of water . . . . . S25
S17 1,3,5-TMPY comparison including monomer transitions . . . . . S26
S18 1-MIM jet FTIR spectra with different concentrations of water . . . . . S27
S19 1-MIM jet FTIR spectra with water comparing He and Ne carrier gas . . . . . S28
S20 1-MIM comparison including monomer transitions . . . . . S29
S21 5-Cl-1,2-DMIM jet FTIR spectra with different concentrations of water . . . . . S30
S22 5-Cl-1,2-DMIM comparison including monomer transitions . . . . . S31
S23 1,2-DMIM jet FTIR spectra with different concentrations of water . . . . . S32
S24 1,2-DMIM jet FTIR spectra with water comparing He and Ne carrier gas . . . . . S33
S25 1,2-DMIM comparison including monomer transitions . . . . . S34
S26 1,2,4-TMIM jet FTIR spectra with different concentrations of water . . . . . S35
S27 1,2,4-TMIM comparison including monomer transitions . . . . . S36
S28 1,2,4,5-TeMIM jet FTIR spectra with different concentrations of water . . . . . S37
S29 1,2,4,5-TeMIM comparison including monomer transitions . . . . . S38
S30 Optimized B2PLYP-D3BJ/def2-QZVP (B2QZ) structures of pyrazole monohydrates . . . . . S41
S31 Optimized B2PLYP-D3BJ/def2-QZVP (B2QZ) structures of imidazole monohydrates . . . . . S42
S32 Optimized B2PLYP-D3BJ/def2-QZVP (B2QZ) structures of pyrazole dihydrates . . . . . S43
S33 Optimized B2PLYP-D3BJ/def2-QZVP (B2QZ) structures of imidazole dihydrates . . . . . S44
S34 Optimized structures of low-lying isomers of halogenated N-heterocycles . . . . . S45
S35 TZ b2 dependence on OHb . . . . . S47
S36 maTZ b2 dependence on OHb . . . . . S47
S37 QZ b2 dependence on OHb . . . . . S48
S38 B2QZ b2 dependence on OHb . . . . . S48
S39 TZ b2ON dependence on OHb . . . . . S48
S40 maTZ b2ON dependence on OHb . . . . . S49
S41 QZ b2ON dependence on OHb . . . . . S49
S42 B2QZ b2ON dependence on OHb . . . . . S49
S43 DFT predictive power for the OHb/b2ON level coincidence in imidazole and pyrazole monohydrates . S51
S44 Intensity trends for dihydrates . . . . . S54
S45 Dihydrate difference spectra for pyrazoles . . . . . S55
S46 DFT predictive power for the OHb/b2 level coincidence in pyrazole dihydrates . . . . . S57
S47 Dihydrate difference spectra for imidazoles . . . . . S60
S48 Optimized B2PLYP-D3BJ/def2-QZVP (B2QZ) structures of 6 new monohydrates . . . . . S70
S49 Optimized B2PLYP-D3BJ/def2-QZVP (B2QZ) structures of 6 new dihydrates . . . . . S71
S50 TZ model performance . . . . . S75

# 1 Experiment

## 1.1 Investigated compounds

Table S1 lists all experimentally investigated chemicals with their respective introduced code names used in the supplementary information and main document as well as the CAS number, the supplier, purity and Lot number.

**Table S1:** Table of investigated chemicals, the introduced code names (supplement and main document), their CAS number, the supplier, purity and Lot#.

| Name | Code | CAS Number | Supplier | Purity | Lot# |
|---|---|---|---|---|---|
| 4-Chloro-1-methylpyrazole | 4-Cl-1-MPY | 35852-81-4 | Fluorochem | 95% | Lg04h0119 |
| 4-Bromo-1-methylpyrazole | 4-Br-1-MPY | 15803-02-8 | TCI chemicals | 98% | ER48O-UH |
| 1-Methylpyrazole | 1-MPY | 930-36-9 | TCI chemicals | 98% | OAUAJ-CS |
| 1,4-Dimethylpyrazole | 1,4-DMPY | 1072-68-0 | Chemat | 95% | AGN25-714094-1 |
| 1,3-Dimethylpyrazole | 1,3-DMPY | 694-48-4 | Chemat | 99.7% | A105054-006 |
| 1,3,5-Trimethylpyrazole | 1,3,5-TMPY | 1072-91-9 | TCI chemicals | 98% | 7MHXH-HH |
| 1-Methylimidazole | 1-MIM | 616-47-7 | TCI chemicals | 99% | Q2EVC-UI |
| 5-Chloro-1,2-dimethylimidazole | 5-Cl-1,2-DMIM | 937818-18-3 | EnamineStore | 95% | 2020-0423555 |
| 1,2-Dimethylimidazole | 1,2-DMIM | 1739-84-0 | TCI chemicals | 98% | MKJ5L-EW |
| 1,2,4-Trimethylimidazole | 1,2,4-TMIM | 1842-63-3 | BLDpharm | 95% | GLX561 |
| 1,2,4,5-Tetramethylimidazole | 1,2,4,5-TeMIM | 1739-83-9 | AmBeed | 99.6% | A515568-AQ5 |
| Demineralised water | $H_2O$ | 7732-18-5 | - | - | - |
| Helium | He | 7440-59-7 | Nippon | 99.9990% | - |
| Neon | Ne | 7440-01-9 | Linde | 99.9990% | - |

Table S2 lists known boiling points and vapor pressures to assess the individual challenge of room temperature detection in the gas phase. For both pyrazole and imidazole, the 1-methyl derivative has a standard boiling point which is lower by about 60 K.[1] From pyrazole to imidazole, the standard boiling point increases by about 70 K. These trends are also reflected in the standard sublimation enthalpies at room temperature, which decrease by about 15 kJ mol$^{-1}$ upon methylation and increase by about 8 kJ mol$^{-1}$ from pyrazole to imidazole.[1] The effects of CH methylation are typically smaller and may differ in direction.

**Table S2:** Table of standard boiling points and vapor pressures near room temperature of pyrazoles and imidazoles at different pressures (1 hPa = 0.001 bar), where sources could be found (important for room temperature nozzle expansions).

| Name | CAS Number | Boiling point / K | Pressure / bar | Source |
|---|---|---|---|---|
| 4-Chloro-1-methylpyrazole | 35852-81-4 | | | |
| 4-Bromo-1-methylpyrazole | 15803-02-8 | | | |
| 1-Methylpyrazole | 930-36-9 | 273.5 | 0.0031 | [2] |
| | | 293.3 | 0.0115 | [2] |
| 1,4-Dimethylpyrazole | 1072-68-0 | | | |
| 1,3-Dimethylpyrazole | 694-48-4 | | | |
| 1,3,5-Trimethylpyrazole | 1072-91-9 | | | |
| 1-Methylimidazole | 616-47-7 | 291.0 | 0.00036 | [3] |
| | | 470-471 | 1.013 | [4] |
| 5-Chloro-1,2-dimethylimidazole | 937818-18-3 | | | |
| 1,2-Dimethylimidazole | 1739-84-0 | 479 | 1.013 | [4] |
| 1,2,4-Trimethylimidazole | 1842-63-3 | | | |
| 1,2,4,5-Tetramethylimidazole | 1739-83-9 | | | |
| 1,3,4,5-tetramethylpyrazole | 1073-20-7 | | | |
| 1-*tert*-Butylpyrazole | 15754-60-6 | | | |
| 1-*tert*-Butylimidazole | 45676-04-8 | 296.4 | 0.00012 | [5] |
| 1-*tert*-Butyl-4-methylimidazole | 67768-62-1 | | | |
| 2-Methyl(-1-trifluoromethyl)-1H-imidazole | 2222049-66-1 | | | |
| 5-Fluoro-1,2,4-trimethylimidazole | | | | |
| Pyrazole | | 282.3 | 0.00004 | [6] |
| Imidazole | | 297.1 | 0.000003 | [6] |
| | | 304.0 | 0.000006 | [3] |

## 1.2 Experimental details

All FTIR measurements were performed using a *g*as *r*ecycling *at*om economic *in*frared (*gratin*) jet spectrometer (for further information related to the setup see reference [7]). To perform soft expansions, the setup was modified by using a Leybold Ecodry 65+ pump instead of the Busch Cobra NS 0070 C screw pump, which cannot handle exhaust pressures below 300 hPa. Additionally, the Pfeiffer ACP 40G pump shown in Fig. S1 was replaced with another Leybold Ecodry 65+ pump. Spectroscopic parameters and details chosen for the different supersonic jet

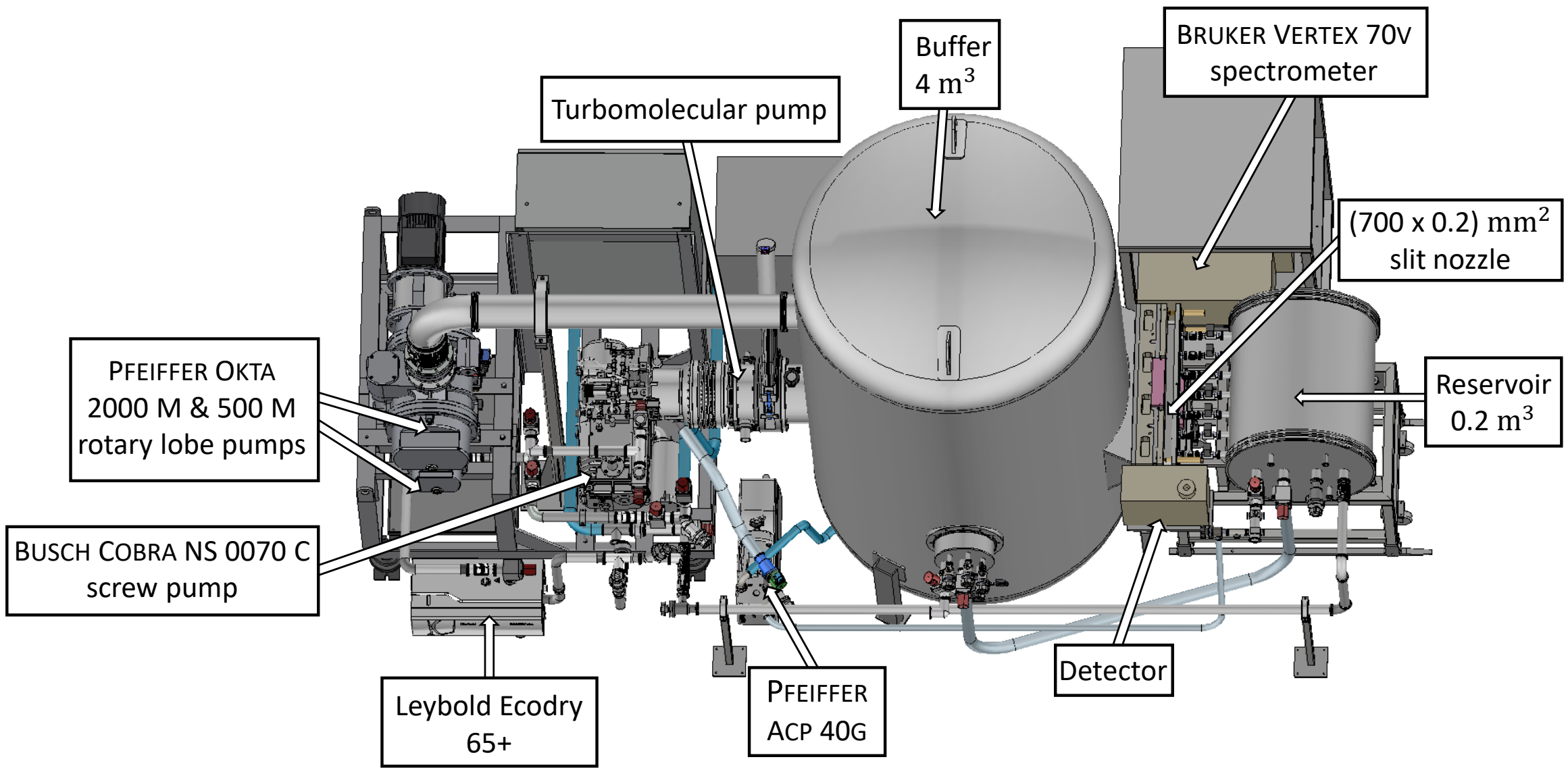


**Figure S1:** Schematic figure of the *gratin*-jet spectrometer[8], created by Reinhard Hildebrandt from the institute's workshop. All evacuations were done using a second Leybold Ecodry 65+ pump instead of the Pfeiffer Acp 40G pump.[8]

expansions are listed in Tables S3 and S4. The codes introduced there are connected with the laboratory journal entries and file names in Tables S5 and S6.

**Table S3:** Spectroscopic details of FTIR spectra shown in Figs. 2 and 5 from the main text and Figs. S2, S3, S4, S5, S6, S7, S8, S9, S10, S11, S12, S16, S17, S45, and S50 from the SI. The partial pressures of the pyrazole ($p_\mathrm{P}$), the solvent ($p_\mathrm{S}$) such as water $H_2O$, as well as the carrier gases helium ($p_\mathrm{He}$) or neon ($p_\mathrm{Ne}$) and the total stagnation pressure of the expansion ($p_\mathrm{s}$) are given. The background pressure in the buffer chamber oscillates between about 0.01 and 0.1 hPa for both He and Ne. The spectra were obtained by averaging # FTIR scans, each synchronized to a 133 ms gas pulse through a 700 mm×0.2 mm slit nozzle with a Bruker VERTEX 70v FTIR spectrometer in double-sided mode at 140 kHz scanning speed. A 20 W tungsten light source, an InSb/HgCdTe sandwich detector and an optical filter (wavenumber range <4000 cm$^{-1}$) were used. The recording date of the spectra is given in a dd/mm/yyyy format and the last column is indicating in which figures and tables in the main text and the SI, the corresponding spectrum is shown.

| code | $p_\mathrm{P}$ / hPa | $p_\mathrm{S}$ / hPa | $p_\mathrm{He}$ / hPa | $p_\mathrm{Ne}$ / hPa | $p_\mathrm{s}$ / hPa | # | dd/mm/yyyy | Figure | Table |
|---|---|---|---|---|---|---|---|---|---|
| | 4-Cl-1-MPY | $H_2O$ | | | | | | | |
| PC1 | 0.1 | 0.2 | 0 | 400 | 400 | 800 | 23/06/2025 | S2, S3, S4 | |
| PC2 | 0.1 | 0.2 | 400 | 0 | 400 | 800 | 03/11/2025 | S3, S4 | |
| PC3 | 0.1 | 0.4 | 0 | 400 | 400 | 900 | 23/06/2025 | 2, 5, S2, S4, S50 | |
| PC4 | 0.1 | 0.8 | 0 | 400 | 400 | 800 | 25/06/2025 | S2, S4 | |
| PC5 | 0.2 | 0.2 | 0 | 400 | 400 | 768 | 24/06/2025 | S2, S4 | |
| | 4-Br-1-MPY | $H_2O$ | | | | | | | |
| PB1 | 0.1 | 0.2 | 0 | 400 | 400 | 830 | 15/05/2025 | S5, S7 | |
| PB2 | 0.1 | 0.4 | 0 | 400 | 400 | 900 | 15/05/2025 | 2, 5, S5, S6, S7, S50 | |
| PB3 | 0.1 | 0.4 | 400 | 0 | 400 | 600 | 28/05/2025 | S6, S7 | |
| PB4 | 0.1 | 0.8 | 0 | 400 | 400 | 900 | 26/06/2025 | S5, S7 | |
| PB5 | 0.2 | 0.2 | 0 | 400 | 400 | 700 | 16/05/2025 | S5, S7 | |
| | 1-MPY | $H_2O$ | | | | | | | |
| PM1 | 0.1 | 0.2 | 0 | 400 | 400 | 850 | 12/05/2025 | S8, S9 | |
| PM2 | 0.1 | 0.2 | 400 | 0 | 400 | 650 | 29/10/2025 | S8, S9 | |
| PM3 | 0.1 | 0.5 | 0 | 400 | 400 | 600 | 28/03/2025 | 2, 5, S8, S9, S50 | |
| PM4 | 0.2 | 0.2 | 0 | 400 | 400 | 800 | 26/05/2025 | S8, S9 | |
| | 1,4-DMPY | $H_2O$ | | | | | | | |
| PMM1 | 0.1 | 0.2 | 0 | 400 | 400 | 800 | 17/06/2025 | S10, S12, S45 | S22 |
| PMM2 | 0.1 | 0.4 | 0 | 400 | 400 | 800 | 18/06/2025 | 2, 5, S10, S11, S12, S45, S50 | |
| PMM3 | 0.1 | 0.4 | 400 | 0 | 400 | 850 | 19/06/2025 | S11, S12 | S21 |
| PMM4 | 0.1 | 0.8 | 0 | 400 | 400 | 900 | 07/07/2025 | S10, S12 | |
| PMM5 | 0.2 | 0.2 | 0 | 400 | 400 | 900 | 17/06/2025 | S10, S12 | |
| | 1,3-DMPY | $H_2O$ | | | | | | | |
| PMM'1 | 0.1 | 0.2 | 0 | 400 | 400 | 700 | 06/10/2025 | S13, S14, S15, S45 | S22 |
| PMM'2 | 0.1 | 0.2 | 400 | 0 | 400 | 800 | 26/11/2025 | S14, S15 | S21 |
| PMM'3 | 0.1 | 0.4 | 0 | 420 | 420 | 850 | 09/09/2025 | 2, 5, S13, S15, S45, S50 | |
| PMM'4 | 0.1 | 0.4 | 400 | 0 | 400 | 700 | 07/10/2025 | S13, S14, S15 | |
| PMM'5 | 0.2 | 0.2 | 0 | 400 | 400 | 800 | 05/09/2025 | S13, S15 | |
| | 1,3,5-TMPY | $H_2O$ | | | | | | | |
| PMMM1 | 0.1 | 0.2 | 0 | 400 | 400 | 800 | 09/10/2025 | S16, S17, S45 | S22 |
| PMMM2 | 0.1 | 0.2 | 400 | 0 | 400 | 800 | 16/10/2025 | S16, S17 | S21 |
| PMMM3 | 0.1 | 0.4 | 0 | 400 | 400 | 800 | 16/10/2025 | 2, 5, S16, S17, S45, S50 | |
| PMMM4 | 0.2 | 0.2 | 0 | 400 | 400 | 800 | 17/10/2025 | S16, S17 | |

**Table S4:** Spectroscopic details of FTIR spectra shown in Figs. 3 and 5 from the main text and Figs. S18, S19, S20, S21, S22, S23, S25, S26, S27, S28 S29, S47, and S50 from the SI. The partial pressures of the imidazole ($p_\mathrm{I}$), the solvent ($p_\mathrm{S}$) such as water $H_2O$, as well as the carrier gases helium ($p_\mathrm{He}$) or neon ($p_\mathrm{Ne}$) and the total stagnation pressure of the expansion ($p_\mathrm{s}$) are given (all in hPa). The background pressure in the buffer chamber oscillates between about 0.01 and 0.1 hPa for both He and Ne. The spectra were obtained by averaging # FTIR scans, each synchronized to a 133 ms gas pulse through a 700 mm×0.2 mm slit nozzle with a Bruker VERTEX 70v FTIR spectrometer in double-sided mode at 140 kHz scanning speed. A 20 W tungsten light source, an InSb/HgCdTe sandwich detector and an optical filter (wavenumber range <4000 cm$^{-1}$) were used. The recording date of the spectra is given in a dd/mm/yyyy format and the last column is indicating in which figures and tables in the main text and the SI, the corresponding spectrum is shown.

| code | $p_\mathrm{I}$ / hPa | $p_\mathrm{S}$ / hPa | $p_\mathrm{He}$ | $p_\mathrm{Ne}$ | $p_\mathrm{s}$ | # | dd/mm/yyyy | Figure | Table |
|---|---|---|---|---|---|---|---|---|---|
| | 1-MIM | $H_2O$ | | | | | | | |
| IM1 | 0.1 | 0.2 | 0 | 400 | 400 | 700 | 24/03/2025 | S18, S19 S20, S47 | |
| IM2 | 0.1 | 0.2 | 440 | 0 | 440 | 800 | 28/10/2025 | S19, S20 | |
| IM3 | 0.1 | 0.4 | 0 | 400 | 400 | 400 | 27/03/2025 | 3, 5, S18, S20, S50 | |
| IM4 | 0.1 | 0.6 | 0 | 400 | 400 | 900 | 30/05/2025 | S18, S20, S47 | |
| IM5 | 0.2 | 0.2 | 0 | 400 | 400 | 700 | 16/06/2025 | S18, S20 | |
| | 5-Cl-1,2-DMIM | $H_2O$ | | | | | | | |
| IC1 | 0.065 | 0.4 | 0 | 400 | 400 | 800 | 10/11/2025 | 3, 5, S21, S22, S47, S50 | |
| IC2 | 0.065 | 0.8 | 0 | 400 | 400 | 700 | 11/11/2025 | S21, S22, S47 | |
| | 1,2-DMIM | $H_2O$ | | | | | | | |
| IMM1 | 0.05 | 0.2 | 0 | 400 | 400 | 700 | 12/01/2026 | S24, S25 | |
| IMM2 | 0.1 | 0.2 | 0 | 400 | 400 | 900 | 04/06/2025 | S23, S25, S47 | |
| IMM3 | 0.1 | 0.4 | 0 | 400 | 400 | 800 | 04/06/2025 | 3, 5, S23, S24, S25, S47, S50 | |
| IMM4 | 0.1 | 0.4 | 400 | 0 | 400 | 600 | 10/06/2025 | S23, S24, S25 | S25, S27 |
| IMM5 | 0.2 | 0.2 | 0 | 400 | 400 | 800 | 05/06/2025 | S23, S25 | |
| | 1,2,4-TMIM | $H_2O$ | | | | | | | |
| IMMM1 | 0.05 | 0.2 | 0 | 400 | 400 | 800 | 13/08/2025 | S26, S27, S47 | |
| IMMM2 | 0.05 | 0.4 | 0 | 400 | 400 | 800 | 12/08/2025 | 3, 5, S26, S27, S47, S50 | |
| IMMM3 | 0.05 | 0.4 | 400 | 0 | 400 | 750 | 14/08/2025 | S26, S27 | S25, S27 |
| | 1,2,4,5-TeMIM | $H_2O$ | | | | | | | |
| IMMMM1 | 0.05 | 0.2 | 0 | 500 | 500 | 800 | 10/10/2025 | S28, S29 | |
| IMMMM2 | 0.03 | 0.2 | 400 | 0 | 400 | 1400 | 23/10/2025 | S28, S29, S47 | S25, S27 |
| IMMMM3 | 0.02 | 0.4 | 0 | 500 | 500 | 800 | 22/10/2025 | 3, 5, S28, S29, S47, S50 | |

**Table S5:** For reference to data repository, the pyrazole hydrate spectra file names are connected to the codes used in Table S3. These corresponding spectra are provided here: dataset.

| code | filename |
|---|---|
| PC1 | 20250623-abc-4-Chloro-1-methylpyrazole-0.1mbar-H2O-0.2mbar-Ne-400mbar-p_s-0,4barpm.800 |
| PC2 | 20251103-ab-4-Chloro-1-methylpyrazole-0.1mbar-H2O-0.2mbar-He-400mbar-p_s-0,4barpm.800 |
| PC3 | 20250623-a-4-Chloro-1-methylpyrazole-0.1mbar-H2O-0.4mbar-Ne-400mbar-p_s-0,4barpm.900 |
| PC4 | 20250625-abc-4-Chloro-1-methylpyrazole-0.1mbar-H2O-0.8mbar-Ne-400mbar-p_s-0,4barpm.800 |
| PC5 | 20250624-abc-4-Chloro-1-methylpyrazole-0.2mbar-H2O-0.2mbar-Ne-400mbar-p_s-0,4barpm.768 |
| PB1 | 20250515-ab-NBE-1-0.1mbar-H2O-0.2mbar-Ne-400mbar-p_s-0,4barpm.830 |
| PB2 | 20250515-a-NBE-1-0.1mbar-H2O-0.4mbar-Ne-400mbar-p_s-0,4barpm.900 |
| PB3 | 20250528-abc-4-Bromo-1-methylpyrazole-0.1mbar-H2O-0.4mbar-He-400mbar-p_s-0,4barpm.600 |
| PB4 | 20250626-abc-NBE-1-0.1mbar-H2O-0.8mbar-Ne-400mbar-p_s-0,4barpm.900 |
| PB5 | 20250516-abcde-NBE-1-0.2mbar-H2O-0.2mbar-Ne-400mbar-p_s-0,4barpm.700 |
| PM1 | 20250512-abc-1-Methylpyrazole-0.1mbar-H2O-0.2mbar-Ne-400mbar-p_s-0,4barpm.850 |
| PM2 | 20251029-abc-1-Methylpyrazole-0.1mbar-H2O-0.2mbar-He-400mbar-p_s-0,4barpm.650 |
| PM3 | 20250328-ab-1-Methylpyrazole-0.1mbar-H2O-0.5mbar-Ne-400mbar-p_s-0,4barpm.600 |
| PM4 | 20250526-abc-1-Methylpyrazole-0.2mbar-H2O-0.2mbar-Ne-400mbar-p_s-0,4barpm.800 |
| PMM1 | 20250617-abcd-1_4-Dimethylpyrazole-0.1mbar-H2O-0.2mbar-Ne-400mbar-p_s-0,4barpm.800 |
| PMM2 | 20250618-abc-1_4-Dimethylpyrazole-0.1mbar-H2O-0.4mbar-Ne-400mbar-p_s-0,4barpm.800 |
| PMM3 | 20250619-abcd-1_4-Dimethylpyrazole-0.1mbar-H2O-0.4mbar-He-400mbar-p_s-0,4barpm.850 |
| PMM4 | 20250707-abc-1_4-Dimethylpyrazole-0.1mbar-H2O-0.8mbar-Ne-400mbar-p_s-0,4barpm.900 |
| PMM5 | 20250617-a-1_4-Dimethylpyrazole-0.2mbar-H2O-0.2mbar-Ne-400mbar-p_s-0,4barpm.900 |
| PMM'1 | 20251006-abc-1_3-Dimethylpyrazole-0.1mbar-H2O-0.2mbar-Ne-400mbar-p_s-0,4barpm.700 |
| PMM'2 | 20251126-a-1_3-Dimethylpyrazole-0.1mbar-H2O-0.2mbar-He-400mbar-p_s-0,4barpm.800 |
| PMM'3 | 20250909-abcd-1_3-Dimethylpyrazole-0.1mbar-H2O-0.4mbar-Ne-420mbar-p_s-0,4barpm.850 |
| PMM'4 | 20251007-ab-1_3-Dimethylpyrazole-0.1mbar-H2O-0.4mbar-He-400mbar-p_s-0,4barpm.700 |
| PMM'5 | 20250905-abc-1_3-Dimethylpyrazole-0.2mbar-H2O-0.2mbar-Ne-400mbar-p_s-0,4barpm.800 |
| PMMM1 | 20251009-ab-1_3_5-Trimethylpyrazole-0.1mbar-H2O-0.2mbar-Ne-400mbar-p_s-0,4barpm.800 |
| PMMM2 | 20251016-a-1_3_5-Trimethylpyrazole-0.1mbar-H2O-0.2mbar-He-400mbar-p_s-0,4barpm.800 |
| PMMM3 | 20251016-abc-1_3_5-Trimethylpyrazole-0.1mbar-H2O-0.4mbar-Ne-400mbar-p_s-0,4barpm.800 |
| PMMM4 | 20251017-ab-1_3_5-Trimethylpyrazole-0.2mbar-H2O-0.2mbar-Ne-400mbar-p_s-0,4barpm.800 |

**Table S6:** For reference to the lab journal and data repository, the pyrazole hydrate spectra file names are connected to the codes used in Table S4. These corresponding spectra are provided here: dataset.

| code | filename |
|---|---|
| IM1 | 20250324-abc-1-Methylimidazole-0.1mbar-H2O-0.2mbar-Ne-400mbar-p_s-0,4barpm.700 |
| IM2 | 20251028-ab-1-Methylimidazole-0.1mbar-H2O-0.2mbar-He-440mbar-p_s-0,4barpm.800 |
| IM3 | 20250327-ab-1-Methylimidazole-0.1mbar-H2O-0.4mbar-Ne-400mbar-p_s-0,4barpm.400 |
| IM4 | 20250530-abcd-1-Methylimidazole-0.1mbar-H2O-0.6mbar-Ne-400mbar-p_s-0,4barpm.900 |
| IM5 | 20250616-abcde-1-Methylimidazole-0.2mbar-H2O-0.2mbar-Ne-400mbar-p_s-0,4barpm.800 |
| IC1 | 20251110-a-5-Chloro-1,2-dimethylimidazole-0.065mbar-H2O-0.4mbar-Ne-400mbar-p_s-0,4barpm.800 |
| IC2 | 20251111-b-5-Chloro-1,2-dimethylimidazole-0.065mbar-H2O-0.8mbar-Ne-400mbar-p_s-0,4barpm.700 |
| IMM1 | 20260112-a-1_2-Dimethylimidazole-0.05mbar-H2O-0.2mbar-Ne-400mbar-p_s-0,4barpm.700 |
| IMM2 | 20250604-a-1_2-Dimethylimidazole-0.1mbar-H2O-0.2mbar-Ne-400mbar-p_s-0,4barpm.900 |
| IMM3 | 20250604-abcd-1_2-Dimethylimidazole-0.1mbar-H2O-0.4mbar-Ne-400mbar-p_s-0,4barpm.800 |
| IMM4 | 20250610-abc-1_2-Dimethylimidazole-0.1mbar-H2O-0.4mbar-He-400mbar-p_s-0,4barpm.600 |
| IMM5 | 20250605-abc-1_2-Dimethylimidazole-0.2mbar-H2O-0.2mbar-Ne-400mbar-p_s-0,4barpm.800 |
| IMMM1 | 20250813-abc-1,2,4-Trimethyl-1H-imidazole-0.05mbar-H2O-0.2mbar-Ne-400mbar-p_s-0,4barpm.800 |
| IMMM2 | 20250812-abc-1,2,4-Trimethyl-1H-imidazole-0.05mbar-H2O-0.4mbar-Ne-400mbar-p_s-0,4barpm.800 |
| IMMM3 | 20250814-ab-1,2,4-Trimethyl-1H-imidazole-0.05mbar-H2O-0.4mbar-He-400mbar-p_s-0,4barpm.750 |
| IMMMM1 | 20251010-ab-1_2_4_5-Tetramethylimidazole-0.05mbar-H2O-0.2mbar-Ne-500mbar-p_s-0,5barpm.800 |
| IMMMM2 | 20251023-abc-1_2_4_5-Tetramethylimidazole-0.03mbar-H2O-0.2mbar-He-400mbar-p_s-0,4barpm.1400 |
| IMMMM3 | 20251022-abc-1_2_4_5-Tetramethylimidazole-0.05_0.02_mbar-H2O-0.4mbar-Ne-500mbar-p_s-0,5barpm.800 |

# 2 Spectral assignments

## 2.1 Nomenclature

A compact nomenclature for each vibration and hydrate complex is introduced. The stoichiometry of the complexes is described as solvate:water. The monohydrate is denoted as 1:1, the dihydrate as 1:2, while 2:1 denotes a hemihydrate (which will be discussed in more detail in a separate study). Our target vibrations are hydrogen-bonded OH stretching vibrations "OHb" of the water donors. This vibrational label is often used explicitly, but for brevity sometimes only implied, whenever a complex composition is listed. In a dihydrate (1:2), there are two OH stretching vibrations: "i" for the indirectly solvating hydrogen bond between two water molecules and "d" for the direct hydrogen bond from the water donor to the solvate acceptor. Bending overtones in water units are denoted "b2" and the three quantum combination state of a bending overtone b2 with the O-N intermolecular stretching fundamental is marked as "b2ON". A summary of the abbreviations is provided in Table S7.

Not all spectra show unambiguous evidence for a bending overtone signal, which has very little intensity on its own but profits from anharmonic resonance with nearby bright OHb states. Whenever 1:1, 1:2 (and traces of 2:1) clusters are simultaneously present in the expansion, it is not always straightforward to disentangle potential b2 contributions in 1:1 clusters from those of 1:2i and of 1:2d water units (2:1 contributions are expected to be negligible under the evaluated expansion conditions). Generally, 1:2d contributions are expected to be stronger than those of 1:2i due to the closer proximity of OHb and b2, although there are cases where OHb falls slightly below b2 in wavenumber but no evidence for a b2 partner is seen. An additional complication are three weak and sharper rovibrational transitions of the bending overtone in water monomer (at 3215, 3196 and 3178 $\mathrm{cm}^{-1}$) in the same region. The specific situation in complex overlap situations is mentioned in the figure captions.

In terms of spectral quantities, we distinguish between raw and different types of deperturbed wavenumbers. Raw wavenumbers can be targeted by fully anharmonic computational methods, whereas deperturbed wavenumbers try to remove the effects of resonances with dark states corresponding to multi-quantum excitations, assuming that they carry no intrinsic intensity. This can be useful for the benchmarking of harmonic or low order perturbation theory treatments. In the present work, the deperturbation (dep) can involve b2ON and/or b2 interaction. Based on the analogy among the investigated pyrazoles and imidazoles, there are also cases where the resonances remain hidden in the noise level, but their intensity can still be estimated either by analogy or as a worst case assumption ($<60\,\mathrm{cm}^{-1}$ coupling matrix element). If they are guessed by analogy, the resonances are included into the deperturbation (dep). If a worst case assumption is made, they are only included as an additional unsymmetric uncertainty in a global deperturbation analysis (gdp), see Table S30.

Another useful experimental quantity for benchmarking purposes is $P$, the fraction of the total OHb intensity contained in the main signal for a given complex. In the dark state perturbation approximation, it corresponds to the OHb

**Table S7:** Nomenclature used for the spectral features observed in this work, which are assumed to derive most of their spectral intensity from a hydrogen-bonded OH group (OHb).

| complex | stretching mode | abbreviation |
|---|---|---|
| monohydrate | bonded OHb | 1:1 |
| | bending overtone | b2 1:1 |
| | bending overtone combined with ON stretch | b2ON |
| dihydrate | directly bonded OHb | 1:2d |
| | in-phase/out-of-phase 1:2d bending overtone | b2 1:2d |
| | indirectly bonded OHb | 1:2i |
| hemihydrate | bonded OHb | 2:1 |
| trihydrate | bonded OHb | 1:3 |
| unspecified | bending overtone | b2 |

probability (hence $P$, can also be read as purity) in this most dominant eigenstate. For a single perturber, $0.5 \leq P \leq 1.0$. For two perturbers, $0.3 \leq P \leq 1.0$. We specify $P$ in steps of 0.05 due to the difficulty to measure reliable intensities and the limited signal-to-noise ratio.

## 2.2 4-Chloro-1-methylpyrazole (4-Cl-1-MPY)

The spectra for 4-Cl-1-MPY are presented in Figs. S2, S3 and S4.

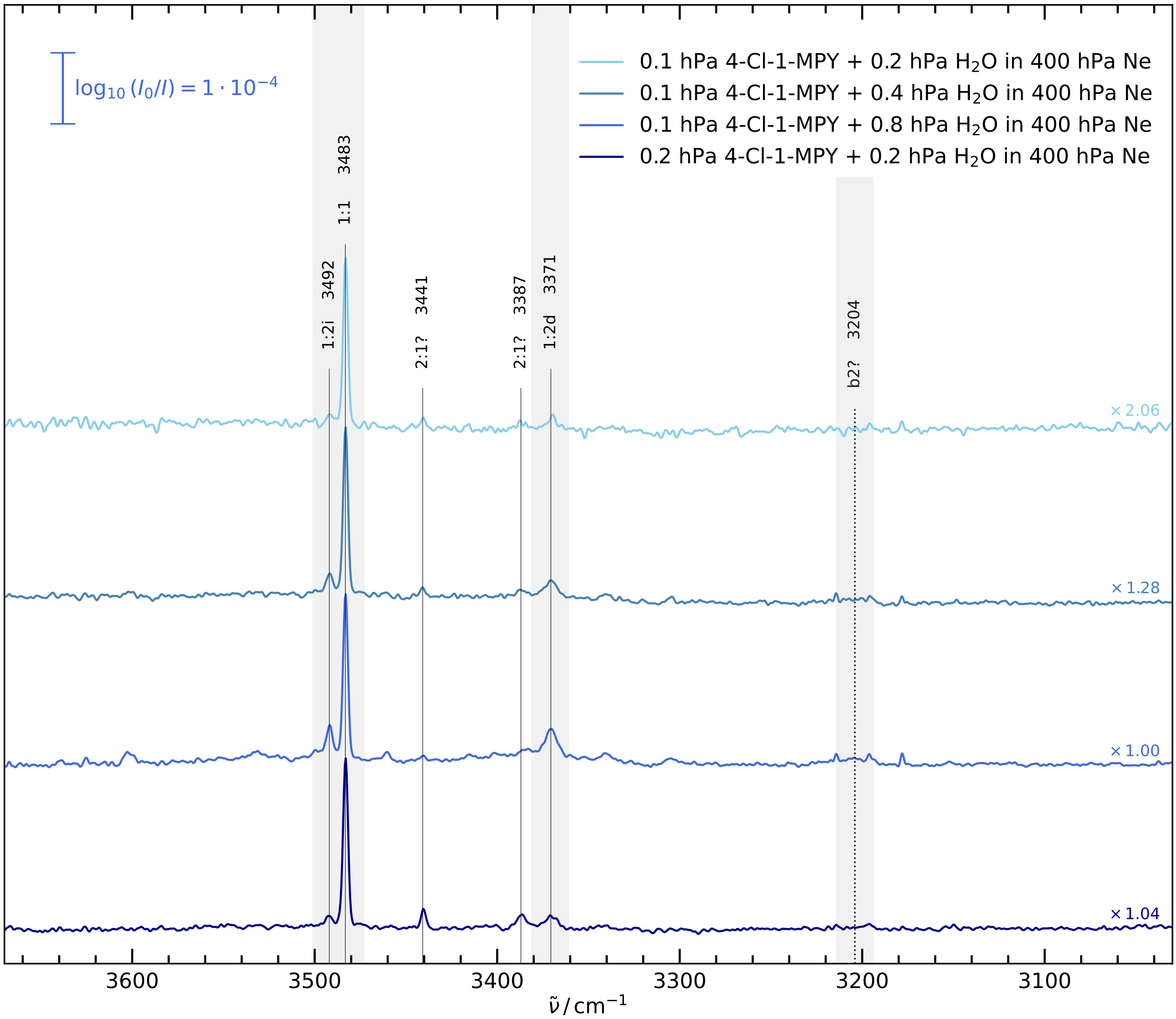


**Figure S2:** Experimental FTIR spectra of 4-chloro-1-methylpyrazole (4-Cl-1-MPY) with different concentrations of water. The monohydrate of 4-Cl-1-MPY is denoted as 1:1 and the dihydrate as 1:2. In the dihydrate, 1:2i is the indirectly solvating water signal and the broad band 1:2d is for the directly contacting water. Possible hemihydrates are labeled as 2:1?. A potential water bending overtone position with weak contributions from 1:1 and/or 1:2d is indicated as b2?. The experimental wavenumbers are provided after each label. The spectra are scaled on the monohydrate, and the scaling factors are marked horizontally.

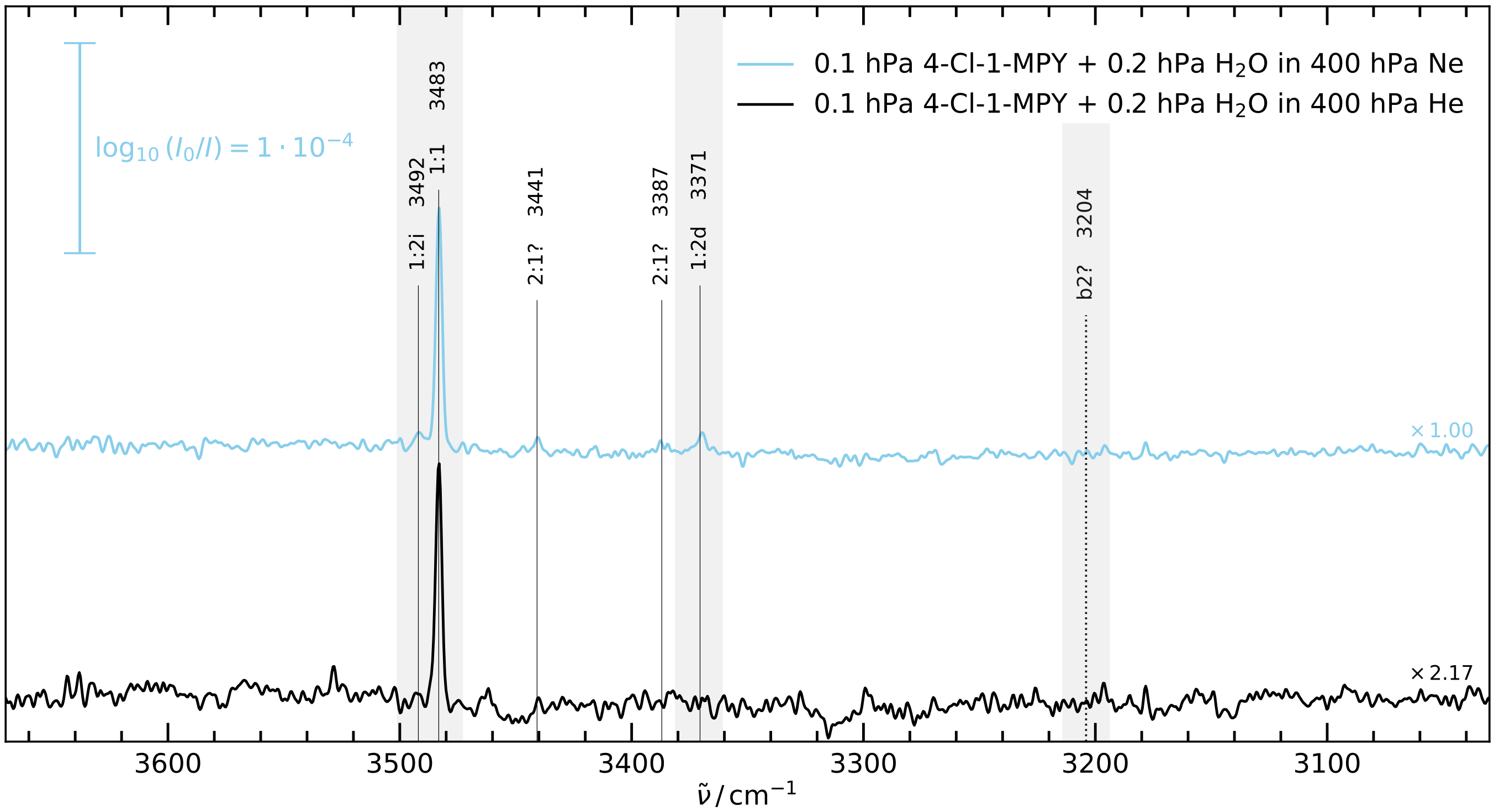


**Figure S3:** Experimental FTIR spectra of 4-chloro-1-methylpyrazole (4-Cl-1-MPY) with water for different carrier gas: helium (He) and neon (Ne). The monohydrate of 4-Cl-1-MPY is denoted as 1:1 and the dihydrate as 1:2. In the dihydrate, 1:2i is the indirectly solvating water signal and the broad band 1:2d is for the directly contacting water. Possible hemihydrates are labeled as 2:1?. The potential position of the water bending overtone is dashed, because it is absent in the displayed spectra. The experimental wavenumbers are provided after each label. The spectra are scaled on the monohydrate, and the scaling factors are marked horizontally. The spectrum (black) with helium as carrier gas has fewer clusters compared to other.

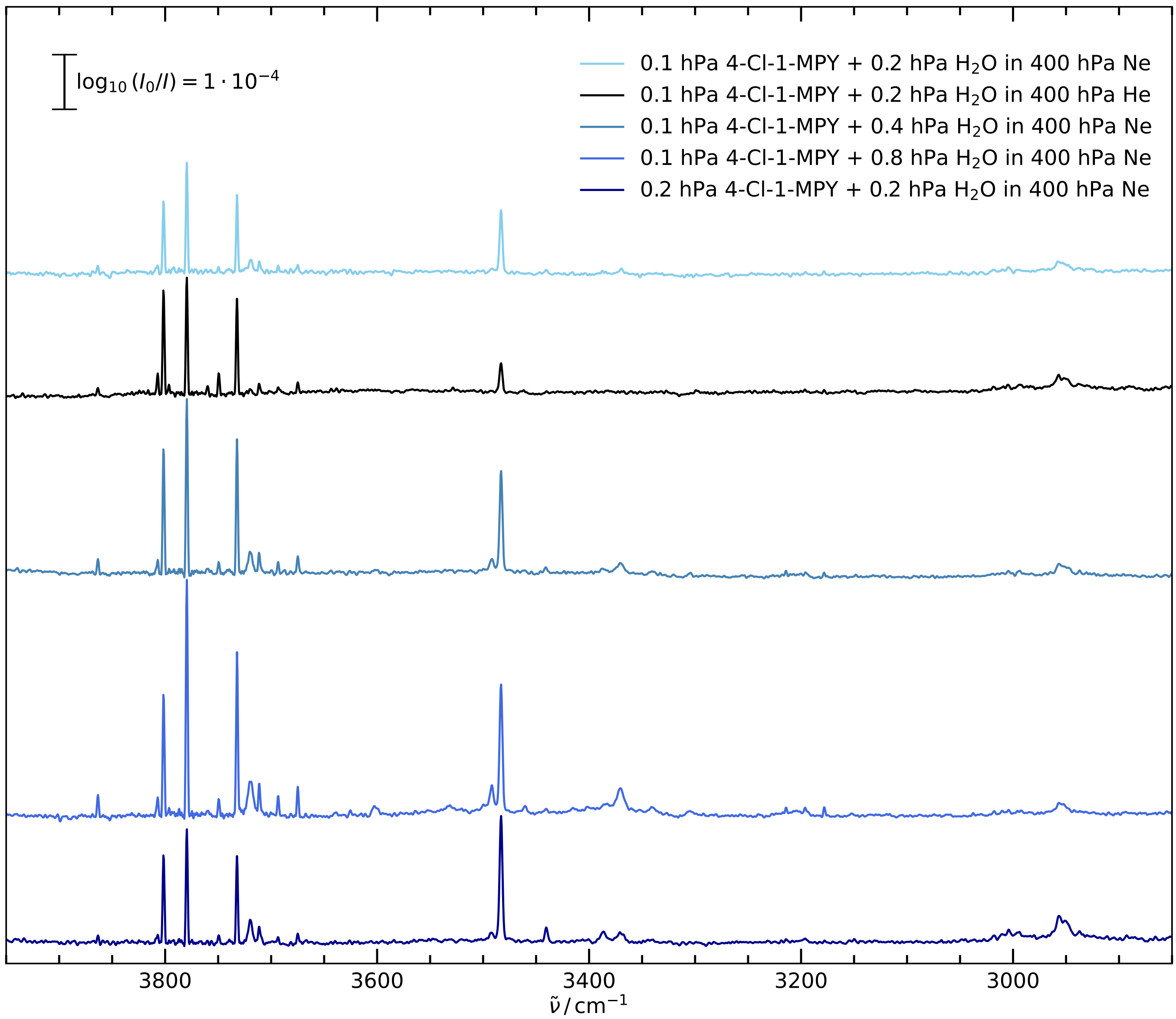


**Figure S4:** Overview FTIR spectra of 4-chloro-1-methylpyrazole (4-Cl-1-MPY) with water, which are shown in Fig. S2 and S3. The CH signals near and below 3000 cm$^{-1}$ reflect the indicated heterocycle partial pressures reasonably well.

## 2.3 4-Bromo-1-methylpyrazole (4-Br-1-MPY)

The spectra for 4-Br-1-MPY are presented in Figs. S5, S6 and S7.

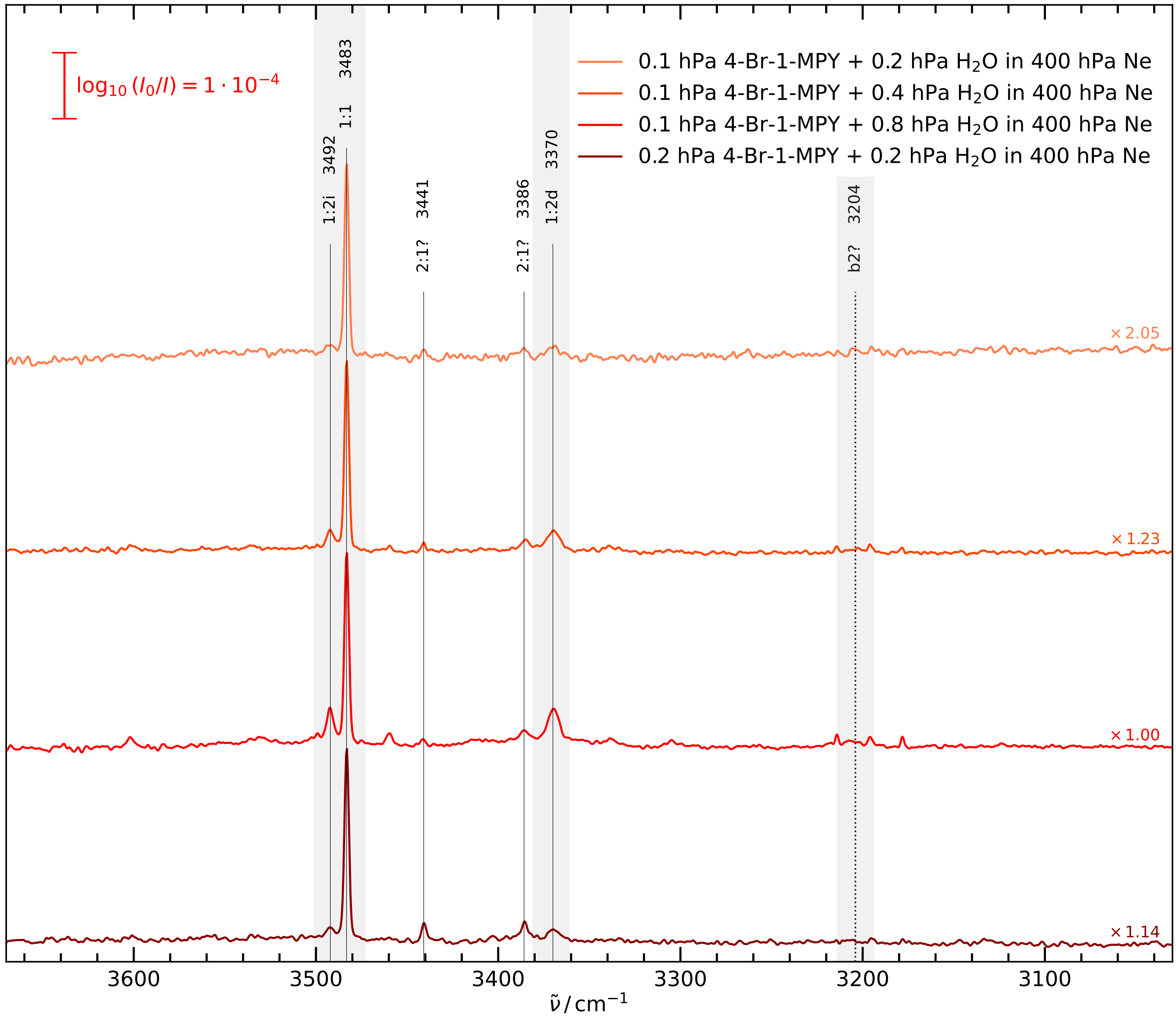


**Figure S5:** Experimental FTIR spectra of 4-bromo-1-methylpyrazole (4-Br-1-MPY) with different concentrations of water. The monohydrate of 4-Br-1-MPY is denoted as 1:1 and the dihydrate as 1:2. In the dihydrate, 1:2i is the indirectly solvating water signal and the broad band 1:2d is for the directly contacting water. Possible hemihydrates are labeled as 2:1?. A potential water bending overtone position with weak contributions from 1:1 and/or 1:2d is indicated as b2?. The three monomer overtone rovibrational transitions at 3215, 3196 and 3178 $cm^{-1}$[9] which complicate band integration in the b2 range are well visible in the second-lowest spectrum. The experimental wavenumbers are provided after each label. The spectra are scaled on the monohydrate, and the scaling factors are marked horizontally.

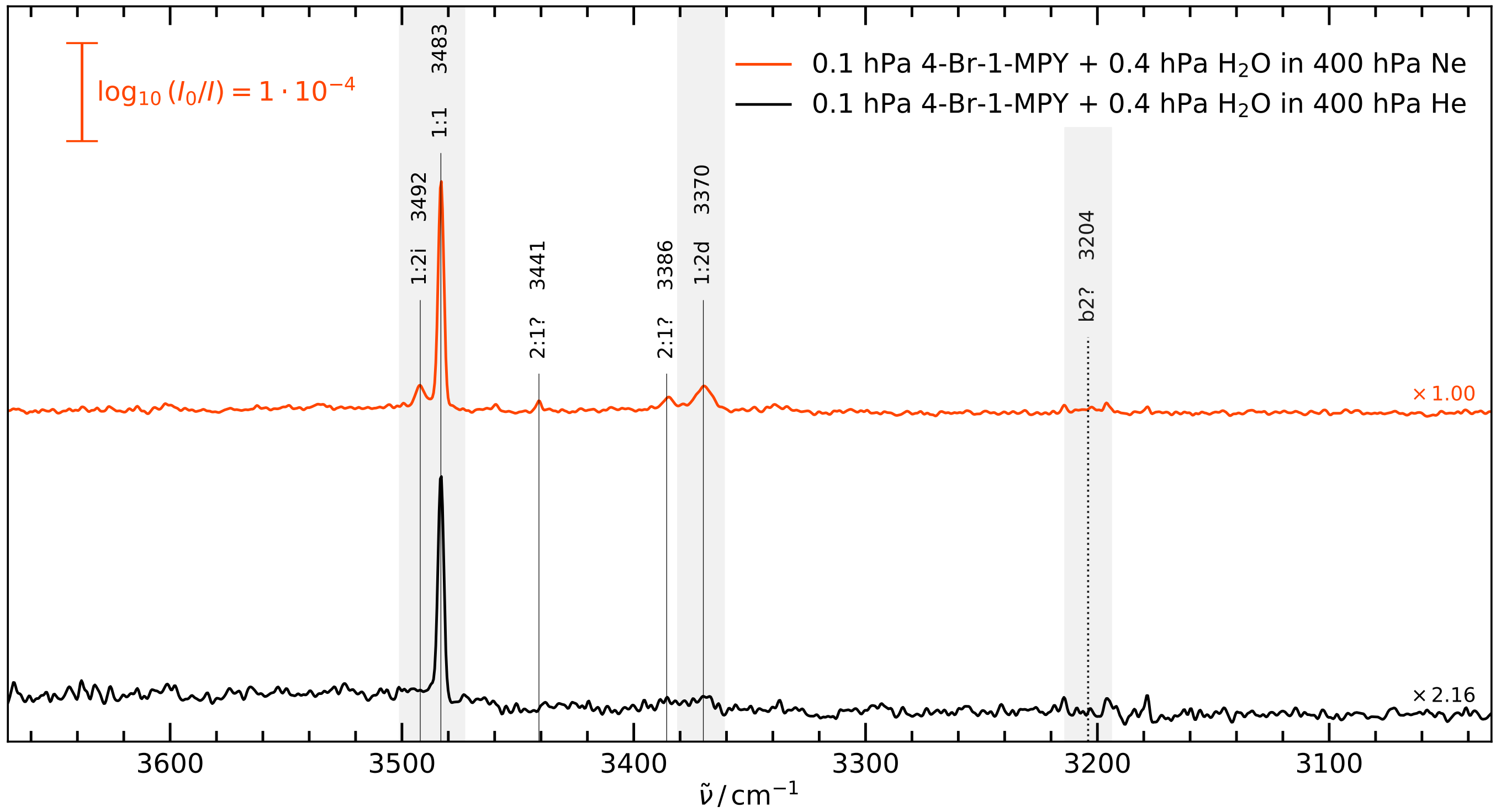


**Figure S6:** Experimental FTIR spectra of 4-bromo-1-methylpyrazole (4-Br-1-MPY) with water for different carrier gas: helium (He) and neon (Ne). The monohydrate of 4-Br-1-MPY is denoted as 1:1 and the dihydrate as 1:2. In the dihydrate, 1:2i is the indirectly solvating water signal and the broad band 1:2d is for the directly contacting water. Possible hemihydrates are labeled as 2:1?. The potential position of the water bending overtone is dashed, because it is absent in the displayed spectra. The experimental wavenumbers are provided after each label. The spectra are scaled on the monohydrate, and the scaling factors are marked horizontally. The spectrum (black) with helium as carrier gas has fewer clusters compared to other.

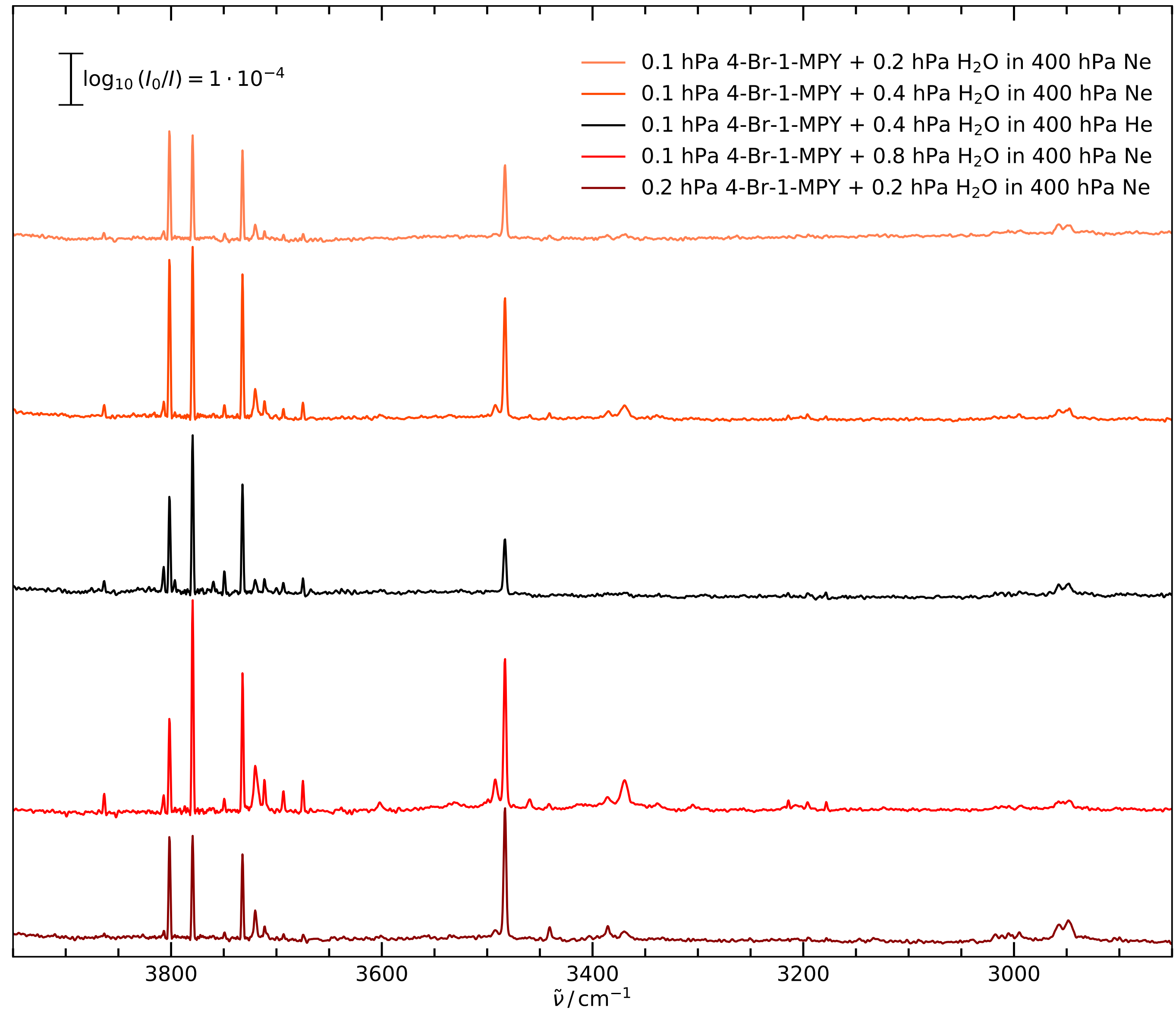


**Figure S7:** Overview FTIR spectra of 4-bromo-1-methylpyrazole (4-Br-1-MPY) with water, which are shown in Fig. S5 and S6. The CH signals near and below 3000 cm$^{-1}$ reflect the indicated heterocycle partial pressures reasonably well.

## 2.4 1-Methylpyrazole (1-MPY)

The spectra for 1-MPY are presented in Figs. S8 and S9.

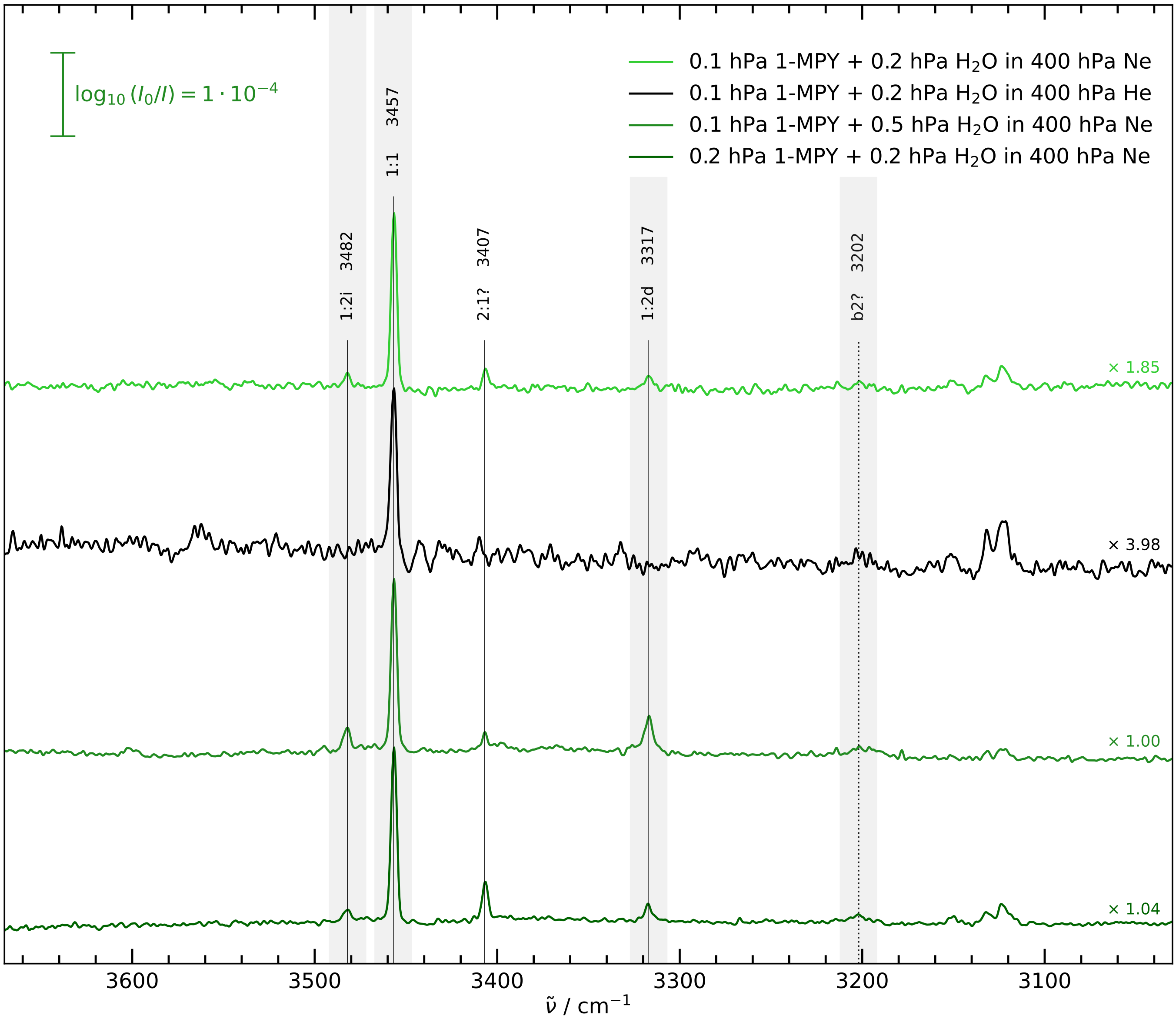


**Figure S8:** Experimental FTIR spectra of 1-methylpyrazole (1-MPY) with different concentrations of water. The monohydrate of 1-MPY is denoted as 1:1 and the dihydrate as 1:2. In the dihydrate, 1:2i is the indirectly solvating water signal and the broad band 1:2d is for the directly contacting water. A possible hemihydrate is labeled as 2:1?. The bending overtone of water is labeled as b2?. The most likely contributors to it are the 1:1 and the 2:1 complexes, although a small 1:2d contribution cannot be ruled out. The experimental wavenumbers are provided after each label. The spectra are scaled on the monohydrate, and the scaling factors are marked horizontally. The spectrum (black) with helium as carrier gas has fewer clusters compared to others.

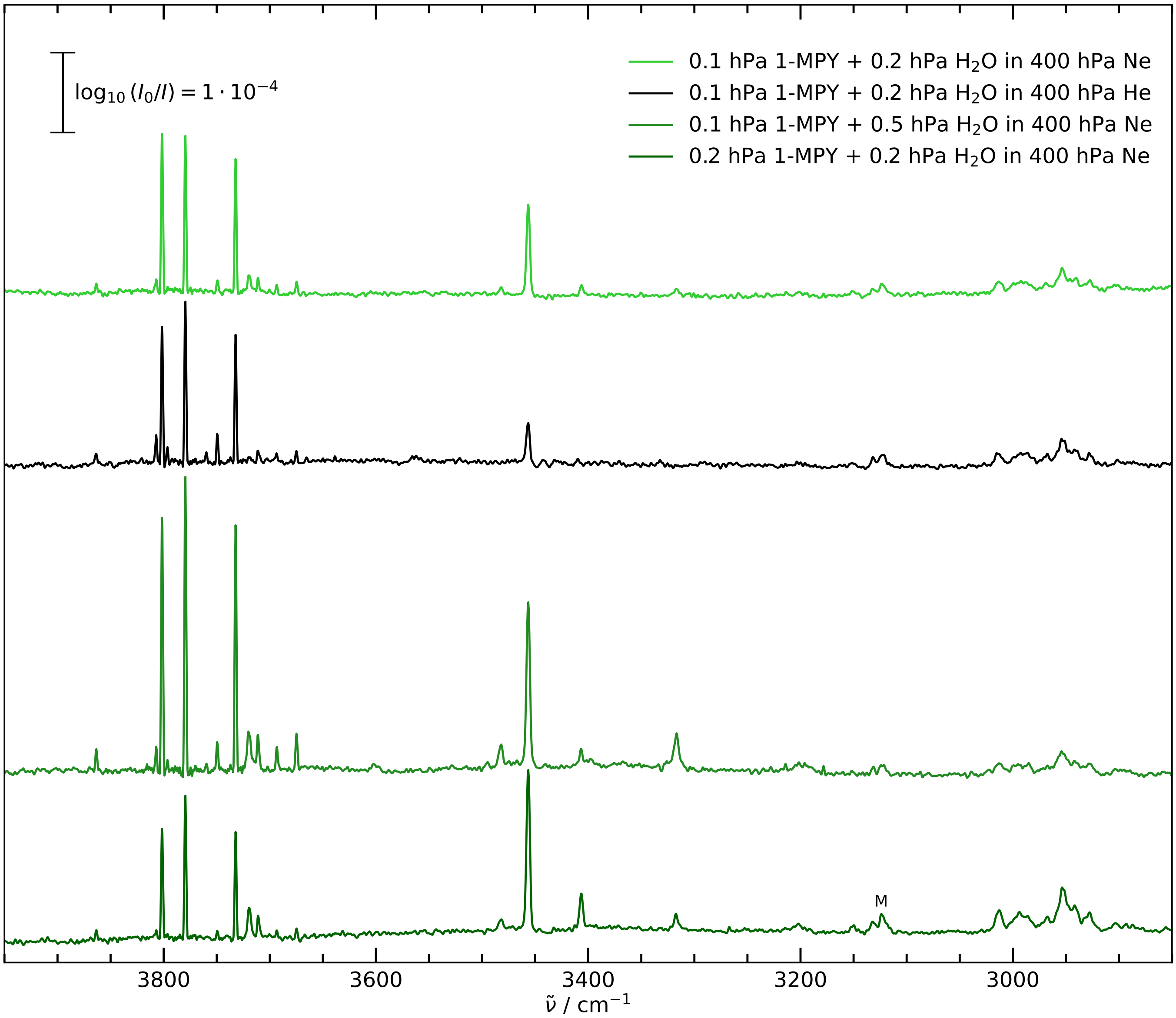


**Figure S9:** Overview FTIR spectra of 1-methylpyrazole (1-MPY) with water, which are shown in Fig. S8. The CH signals near 3000 cm$^{-1}$ reflect the indicated heterocycle partial pressures reasonably well. In the bottom spectrum, a particularly high-frequency CH stretching fundamental of 1-MPY near 3120 cm$^{-1}$ is marked M, because it comes close to the b2 region of the clusters.

## 2.5 1,4-Dimethylpyrazole (1,4-DMPY)

The spectra for 1,4-DMPY are presented in Figs. S10, S11 and S12.

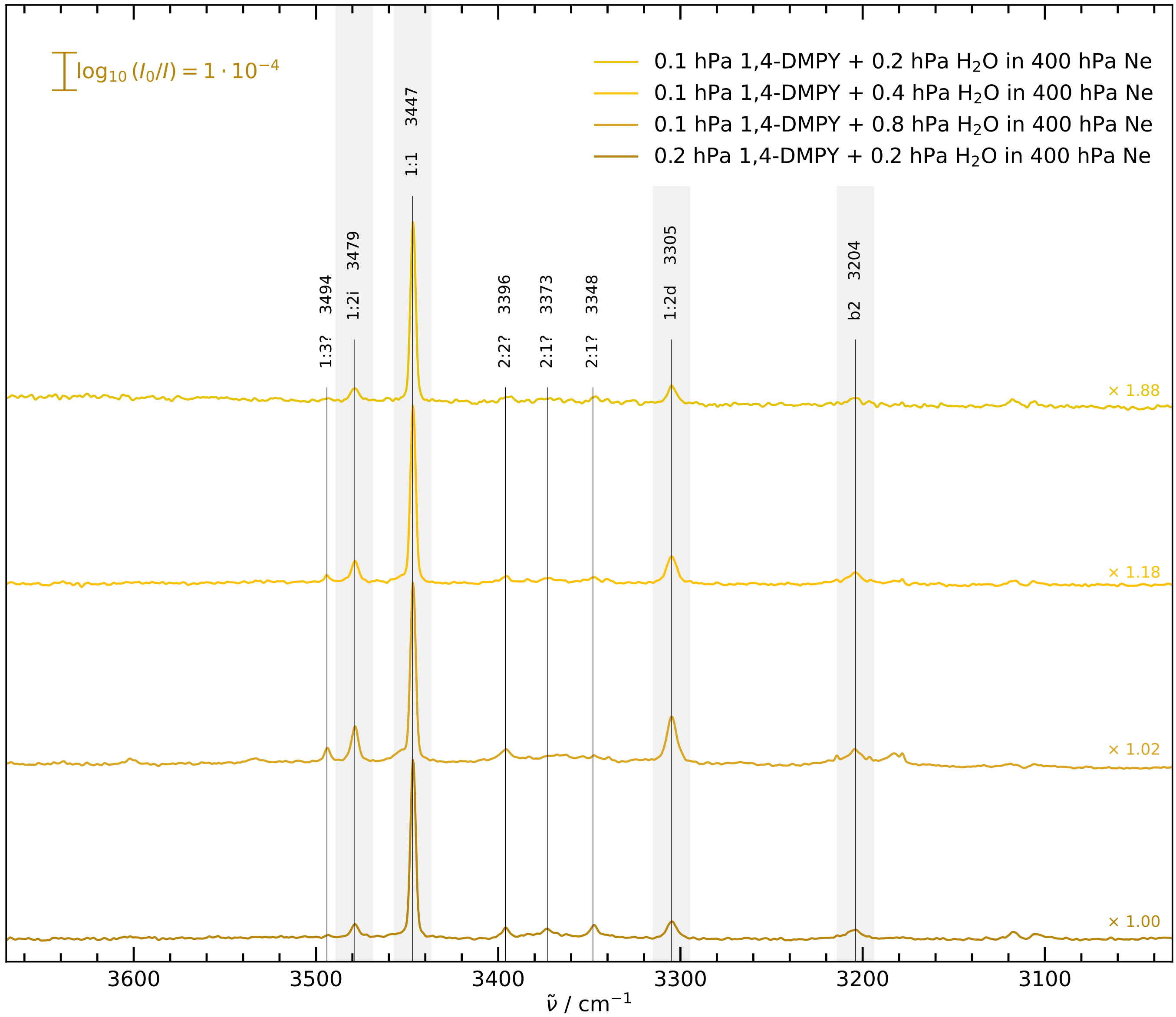


**Figure S10:** Experimental FTIR spectra of 1,4-dimethylpyrazole (1,4-DMPY) with different concentrations of water. The monohydrate of 1,4-DMPY is denoted as 1:1 and the dihydrate as 1:2. In the dihydrate, 1:2i is the indirectly solvating water signal and the broad band 1:2d is for the directly contacting water. Possible hemihydrates are labeled as 2:1?. The bending overtone of water is labeled as b2 and has likely resonance contributions from 1:1 and 1:2d. The experimental wavenumbers are provided after each label. The spectra are scaled on the monohydrate, and the scaling factors are marked horizontally. The 2:2?-assignment is particularly tentative and would profit from a supporting Raman jet measurement. This is also the case for the signal near 3180 $cm^{-1}$ which may be due to a trihydrate, among other possibilities.

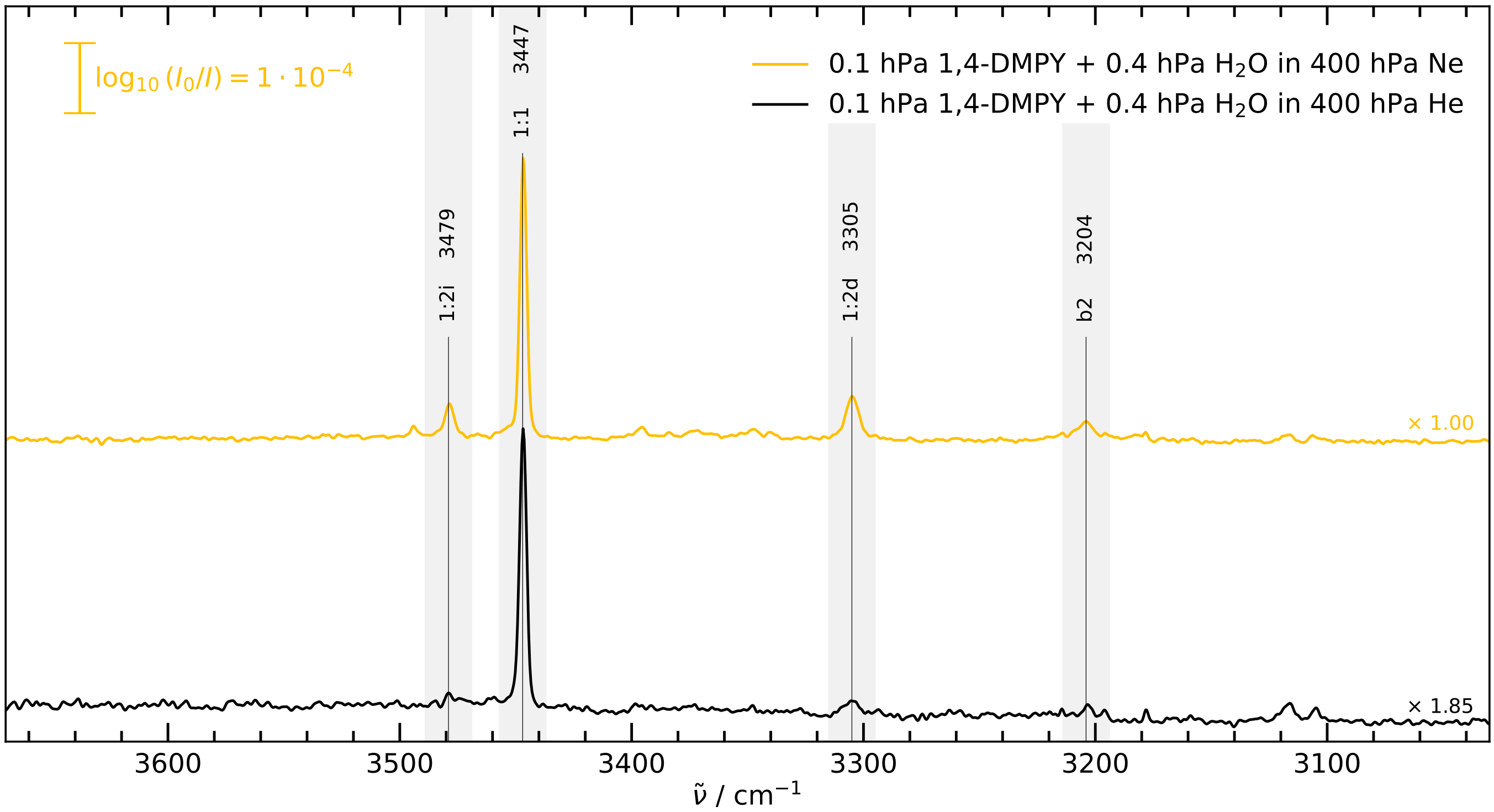


**Figure S11:** Experimental FTIR spectra of 1,4-dimethylpyrazole (1,4-DMPY) with water for different carrier gas: helium (He) and neon (Ne). The monohydrate of 1,4-DMPY is denoted as 1:1 and the dihydrate as 1:2. In the dihydrate, 1:2i is the indirectly solvating water signal and the broad band 1:2d is for the directly contacting water. Possible hemihydrates are labeled as 2:1?. The bending overtone of water is labeled as b2. The most likely contributors to b2 are the 1:1 and 1:2d water molecules. The experimental wavenumbers are provided after each label. The spectra are scaled on the monohydrate, and the scaling factors are marked horizontally. The spectrum (black) with helium as carrier gas has fewer clusters compared to other.

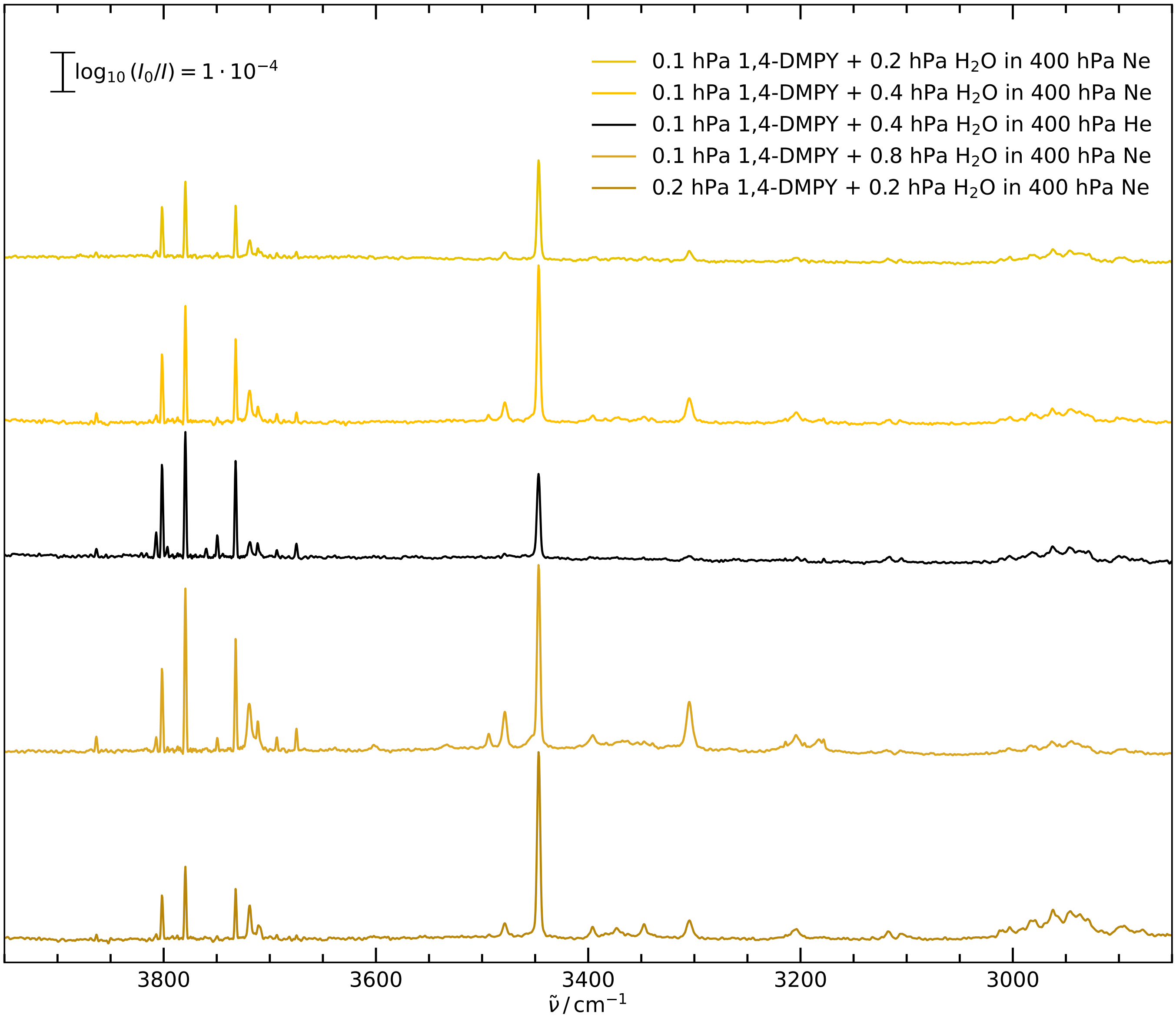


**Figure S12:** Overview FTIR spectra of 1,4-dimethylpyrazole (1,4-DMPY) with water, which are shown in Fig. S10 and S11. The CH signals below 3000 cm$^{-1}$ reflect the indicated heterocycle partial pressures reasonably well.

## 2.6 1,3-Dimethylpyrazole (1,3-DMPY)

The spectra for 1,3-DMPY are presented in Figs. S13, S14 and S15.

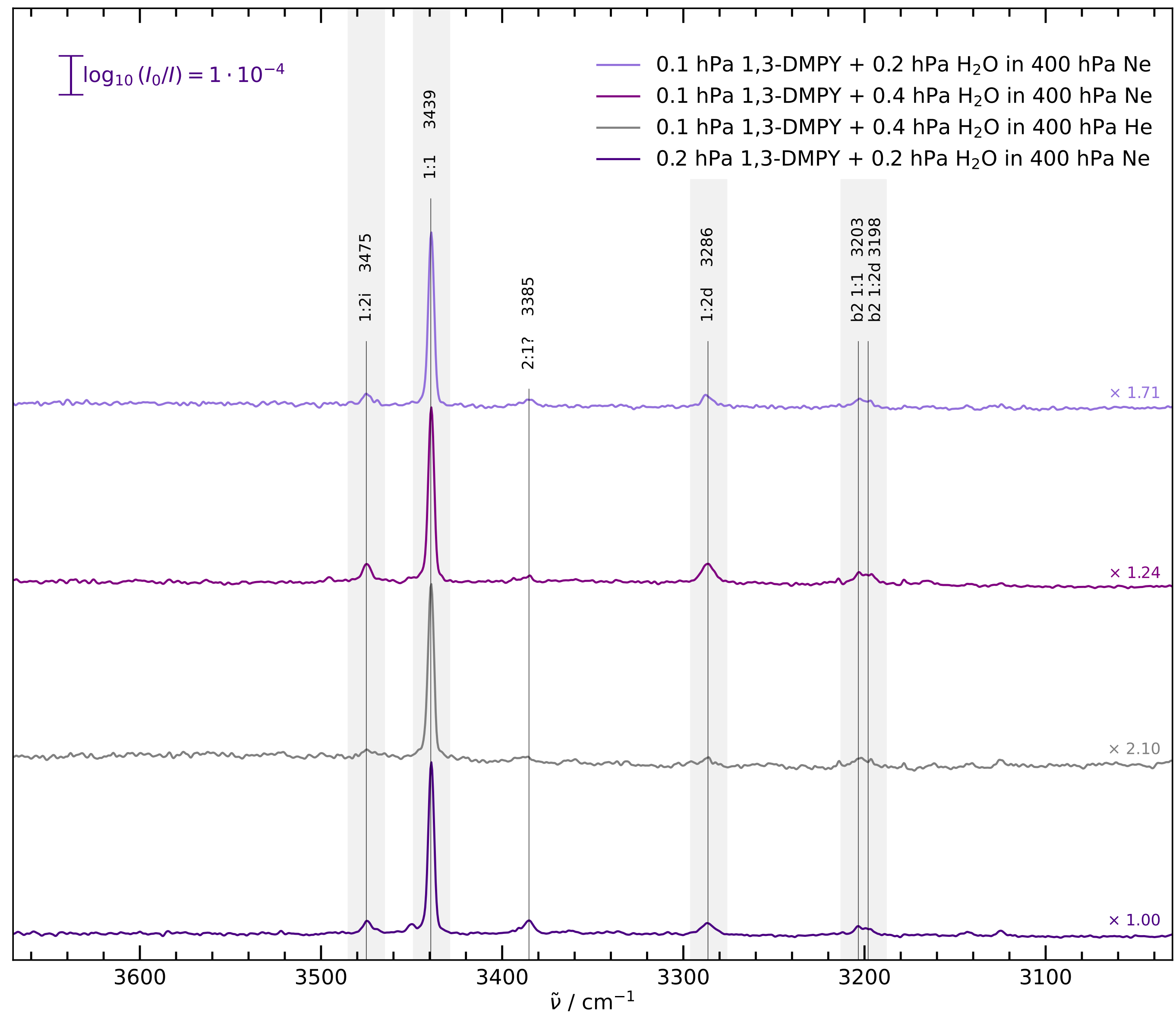


**Figure S13:** Experimental FTIR spectra of 1,3-dimethylpyrazole (1,3-DMPY) with different concentrations of water. The monohydrate of 1,3-DMPY is denoted as 1:1 and the dihydrate as 1:2. In the dihydrate, 1:2i is the indirectly solvating water signal and the broad band 1:2d is for the directly contacting water. A possible hemihydrate is labeled as 2:1?. The bending overtone of water is labeled as b2. The most likely contributions to b2 are the 1:1 and 1:2d water complexes, with the latter contributing at somewhat lower wavenumber than the former. The experimental wavenumbers are provided after each label. The spectra are scaled on the monohydrate, and the scaling factors are also marked. The spectrum (grey) with helium as carrier gas has fewer clusters compared to others.

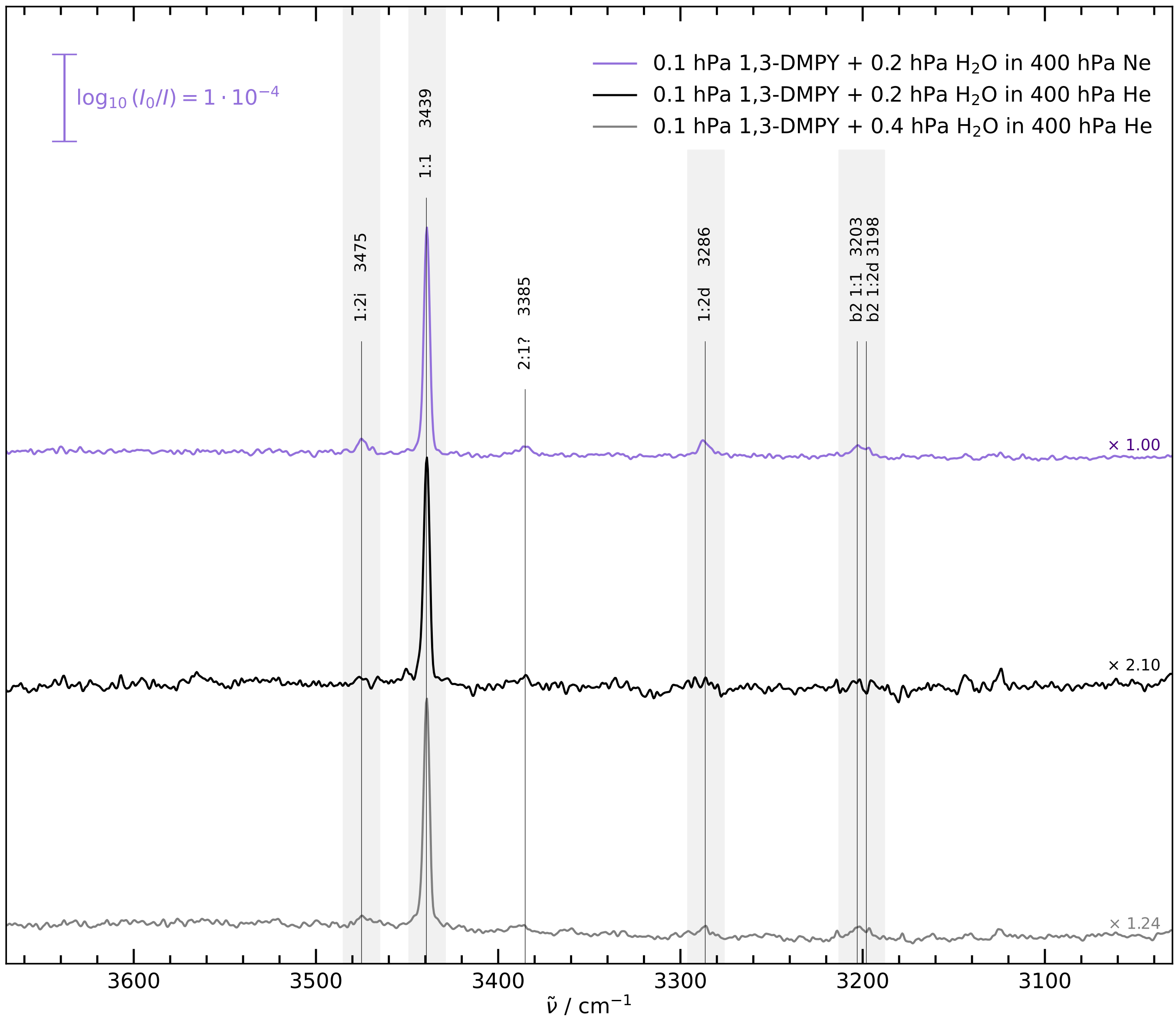


**Figure S14:** Experimental FTIR spectra of 1,3-dimethylpyrazole (1,3-DMPY) with water for different carrier gas: helium (He) and neon (Ne). The monohydrate of 1,3-DMPY is denoted as 1:1 and the dihydrate as 1:2. In the dihydrate, 1:2i is the indirectly solvating water signal and the broad band 1:2d is for the directly contacting water. Possible hemihydrates are labeled as 2:1?. The bending overtone of water is labeled as b2. The experimental wavenumbers are provided after each label. The spectra are scaled on the monohydrate, and the scaling factors are marked horizontally. The spectrum (black/grey) with helium as carrier gas has fewer clusters compared to other.

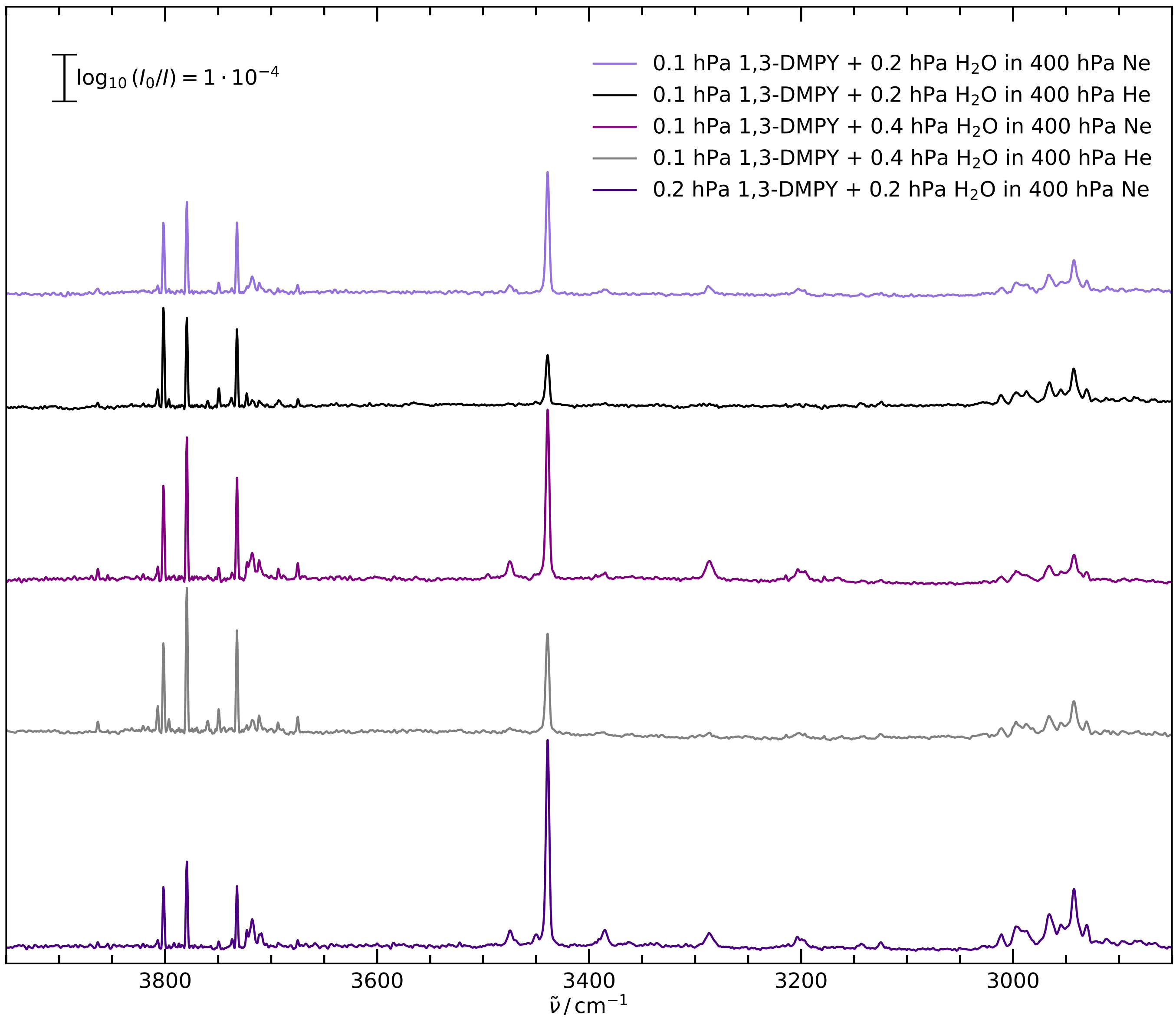


**Figure S15:** Overview FTIR spectra of 1,3-dimethylpyrazole (1,3-DMPY) with water, which are shown in Fig. S13. The CH signals below 3000 cm$^{-1}$ reflect the indicated heterocycle partial pressures reasonably well.

## 2.7 1,3,5-Trimethylpyrazole (1,3,5-TMPY)

The spectra for 1,3,5-TMPY are presented in Figs. S16 and S17.

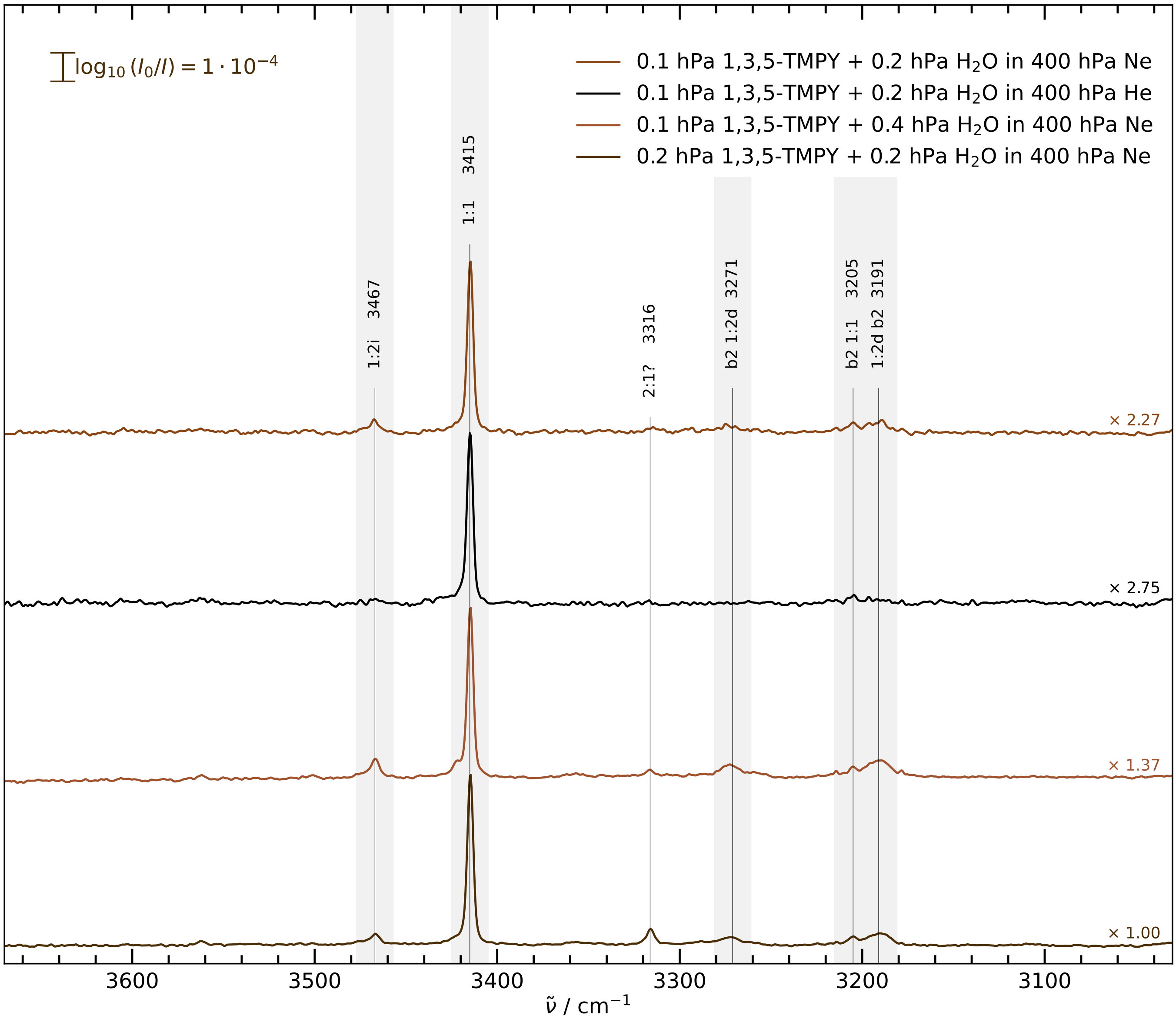


**Figure S16:** Experimental FTIR spectra of 1,3,5-trimethylpyrazole (1,3,5-TMPY) with different concentrations of water. The monohydrate of 1,3,5-TMPY is denoted as 1:1 and the dihydrate as 1:2. In the dihydrate, 1:2i is the indirectly solvating water signal and the broad band 1:2d is for the directly contacting water. Possible hemihydrates are labeled as 2:1?. The bending overtones of water are labeled as b2. In this case, there are at least two separate contributions, which could in principle originate in 1:1, 2:1, 1:2i and 1:2d water molecules. Due to the smaller distance from OHb, 1:2d should dominate over 1:2i. The most likely assignment is given in the spectrum. The experimental wavenumbers are provided after each label. The spectra are scaled on the monohydrate, and the scaling factors are marked horizontally. The spectrum (black) with helium as carrier gas has fewer clusters compared to others.

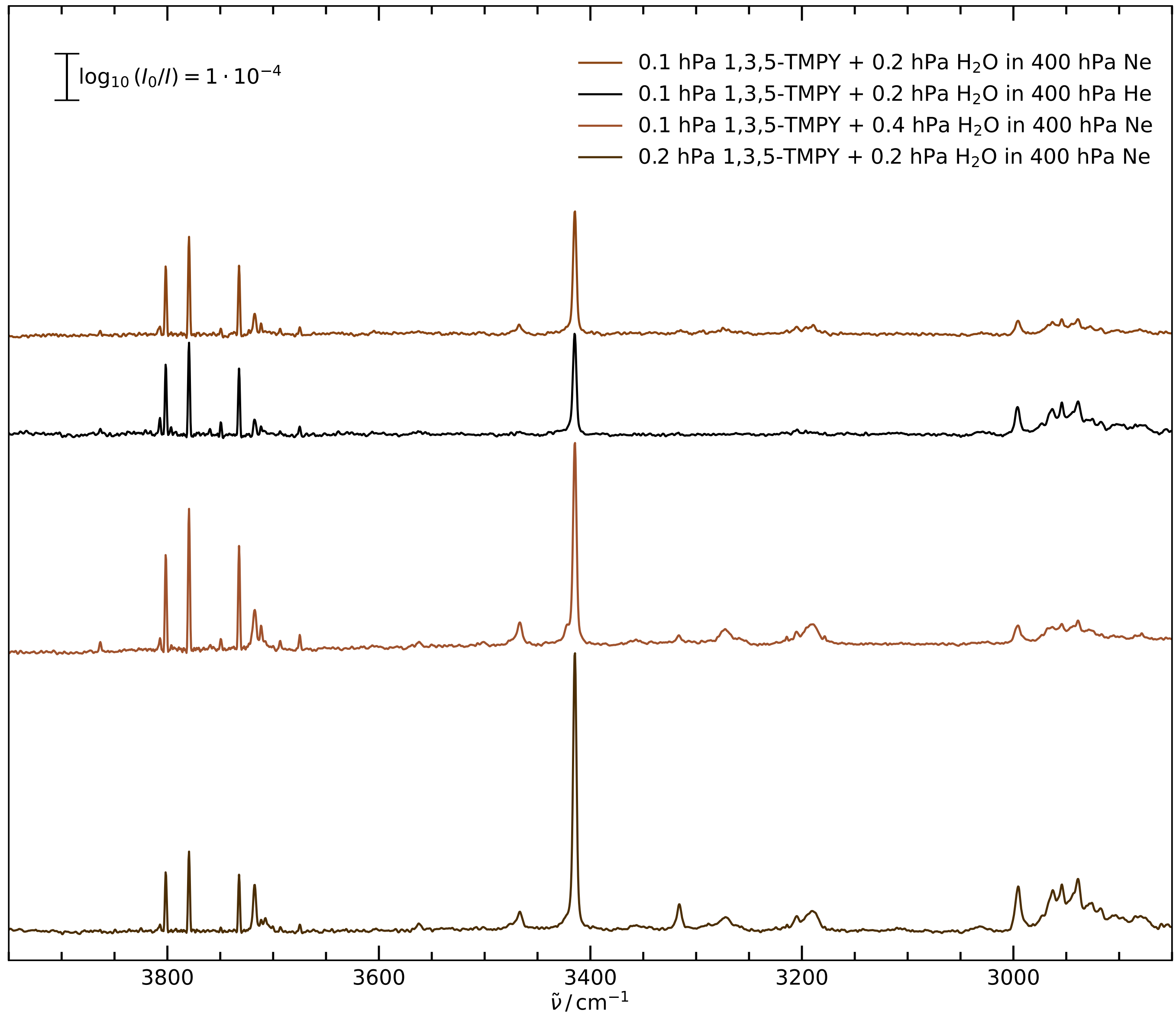


**Figure S17:** Overview FTIR spectra of 1,3,5-trimethylpyrazole (1,3,5-TMPY) with water, which are shown in Fig. S16. The CH signals below 3000 cm$^{-1}$ reflect the indicated heterocycle partial pressures reasonably well, but the He expansion probably has a higher pyrazole concentration than indicated.

## 2.8 1-Methylimidazole (1-MIM)

The spectra for 1-MIM are presented in Figs. S18, S19 and S20.

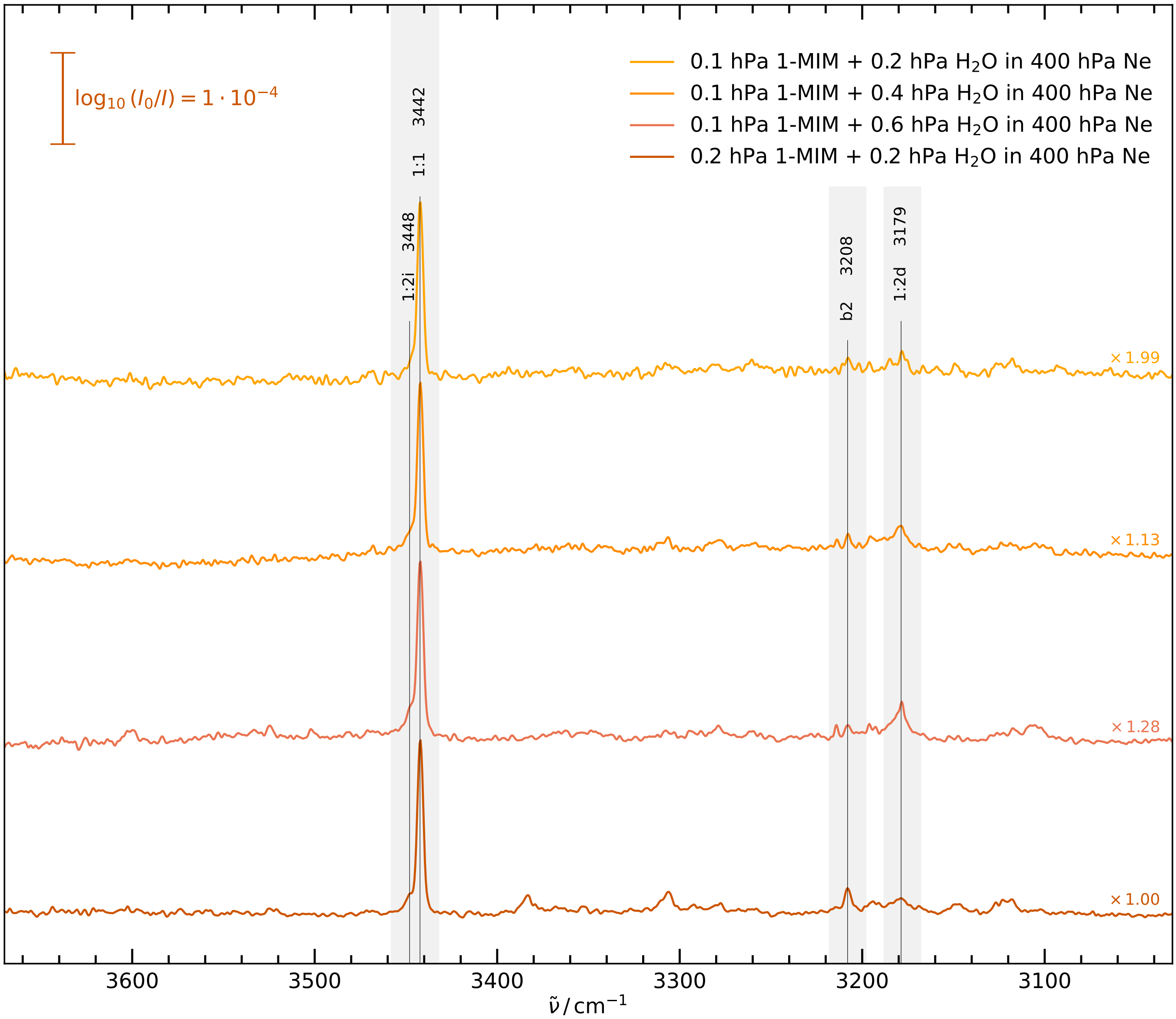


**Figure S18:** Experimental FTIR spectra of 1-methylimidazole (1-MIM) with different concentrations of water. The monohydrate of 1-MIM is denoted as 1:1 and the dihydrate as 1:2. In the dihydrate, 1:2i is the indirectly solvating water signal and the broad band 1:2d is for the directly contacting water. The bending overtone of water in the 1:1 complex is labeled as b2. The experimental wavenumbers are provided after each label. The spectra are scaled on the monohydrate, and the scaling factors are marked horizontally.

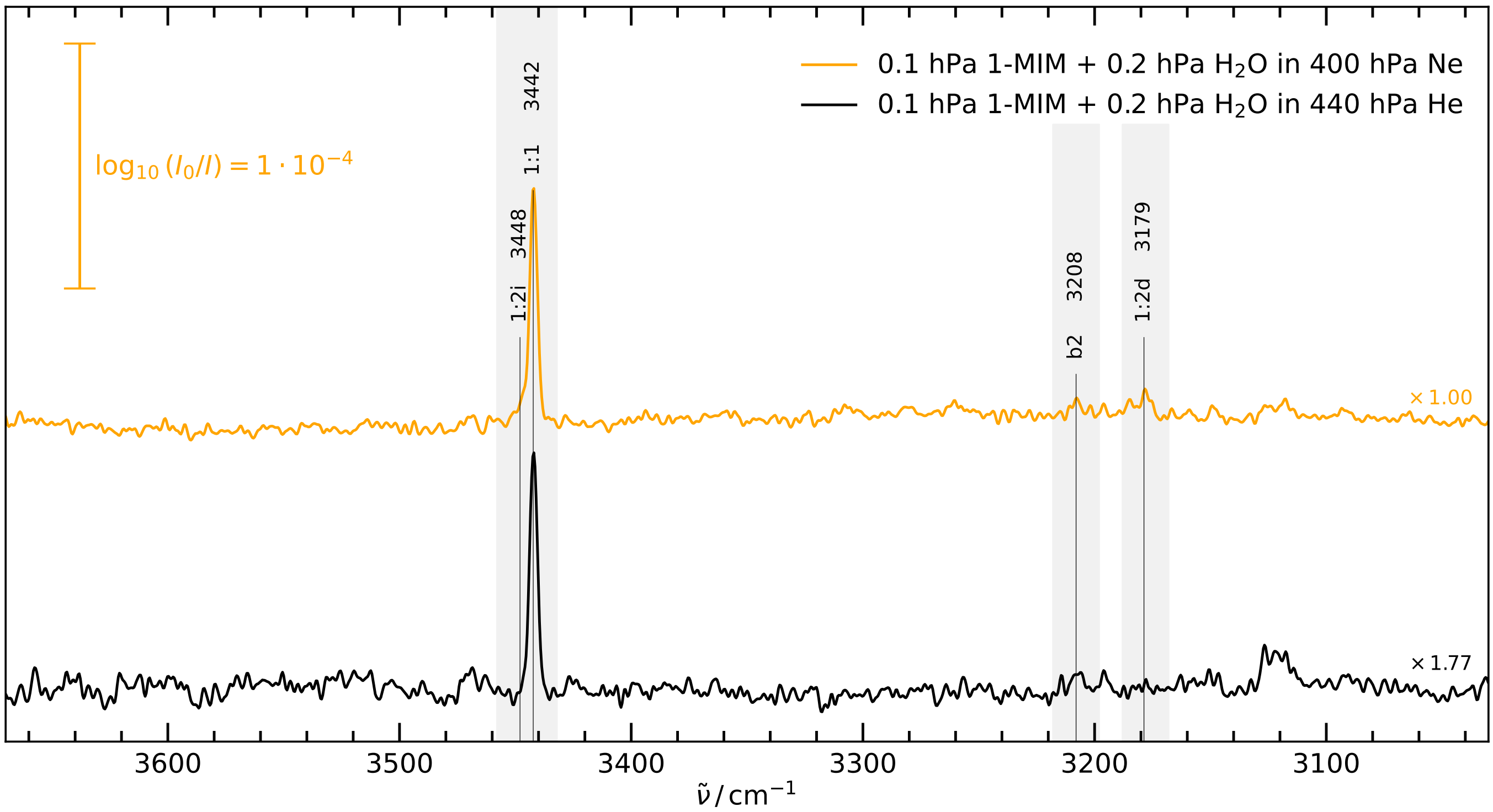


**Figure S19:** Experimental FTIR spectra of 1-methylimidazole (1-MIM) with water for different carrier gas: helium (He) and neon (Ne). The monohydrate of 1-MIM is denoted as 1:1 and the dihydrate as 1:2. In the dihydrate, 1:2i is the indirectly solvating water signal and the broad band 1:2d is for the directly contacting water. The bending overtone of water in the 1:1 complex is labeled as b2. The experimental wavenumbers are provided after each label. The spectra are scaled on the monohydrate, and the scaling factors are marked horizontally. The spectrum (black) with helium as carrier gas has fewer clusters compared to other.

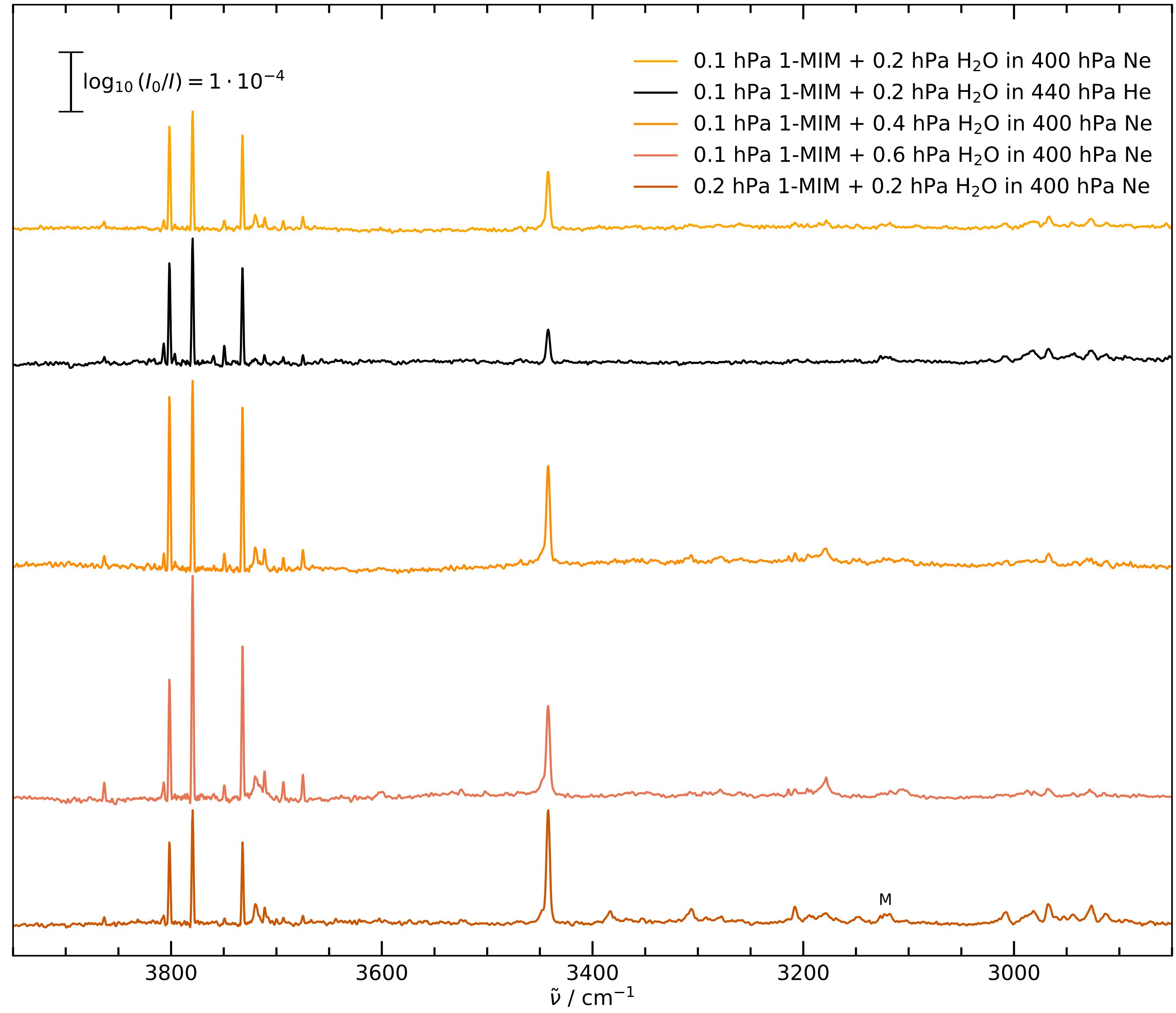


**Figure S20:** Overview FTIR spectra of 1-methylimidazole (1-MIM) with water, which are shown in Fig. S18 and S19. In the bottom spectrum, a particularly high-frequency CH stretching fundamental of 1-MIM near 3120 $cm^{-1}$ is marked M, because it comes close to the b2 region of the clusters.

## 2.9 5-Chloro-1,2-dimethylimidazole (5-Cl-1,2-DMIM)

The spectra for 5-Cl-1,2-DMIM are presented in Figs. S21 and S22.

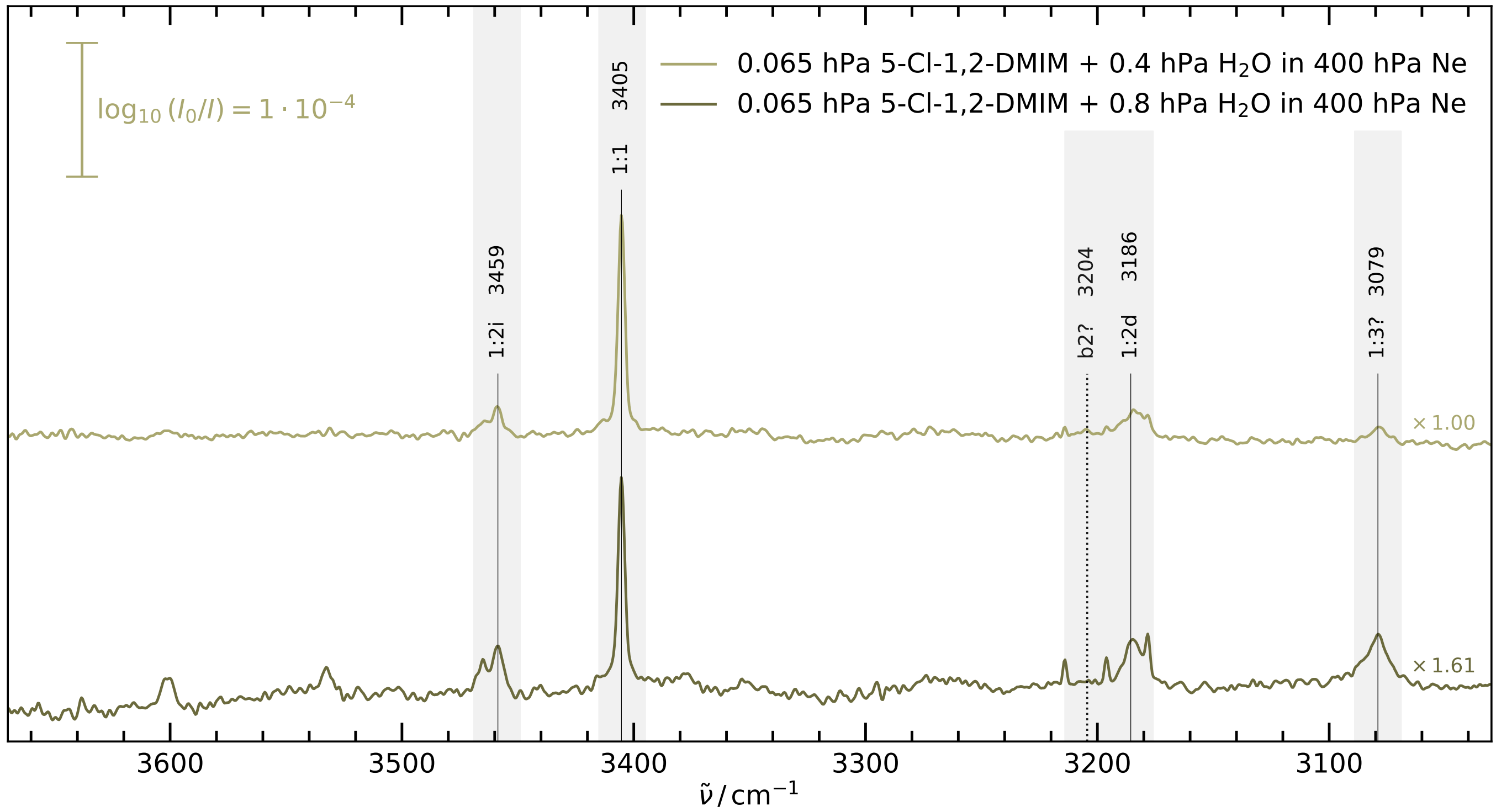


**Figure S21:** Experimental FTIR spectra of 5-chloro-1,2-dimethylimidazole (5-Cl-1,2-DMIM) with different concentrations of water. The monohydrate of 5-Cl-1,2-DMIM is denoted as 1:1 and the dihydrate as 1:2. In the dihydrate, 1:2i is the indirectly solvating water signal and the broad band 1:2d is for the directly contacting water. The position of a potential bending overtone of water in the 1:1 complex is labeled as b2?. The spectra are scaled on the monohydrate, and the scaling factors are marked horizontally. The experimental wavenumbers are provided after each label.

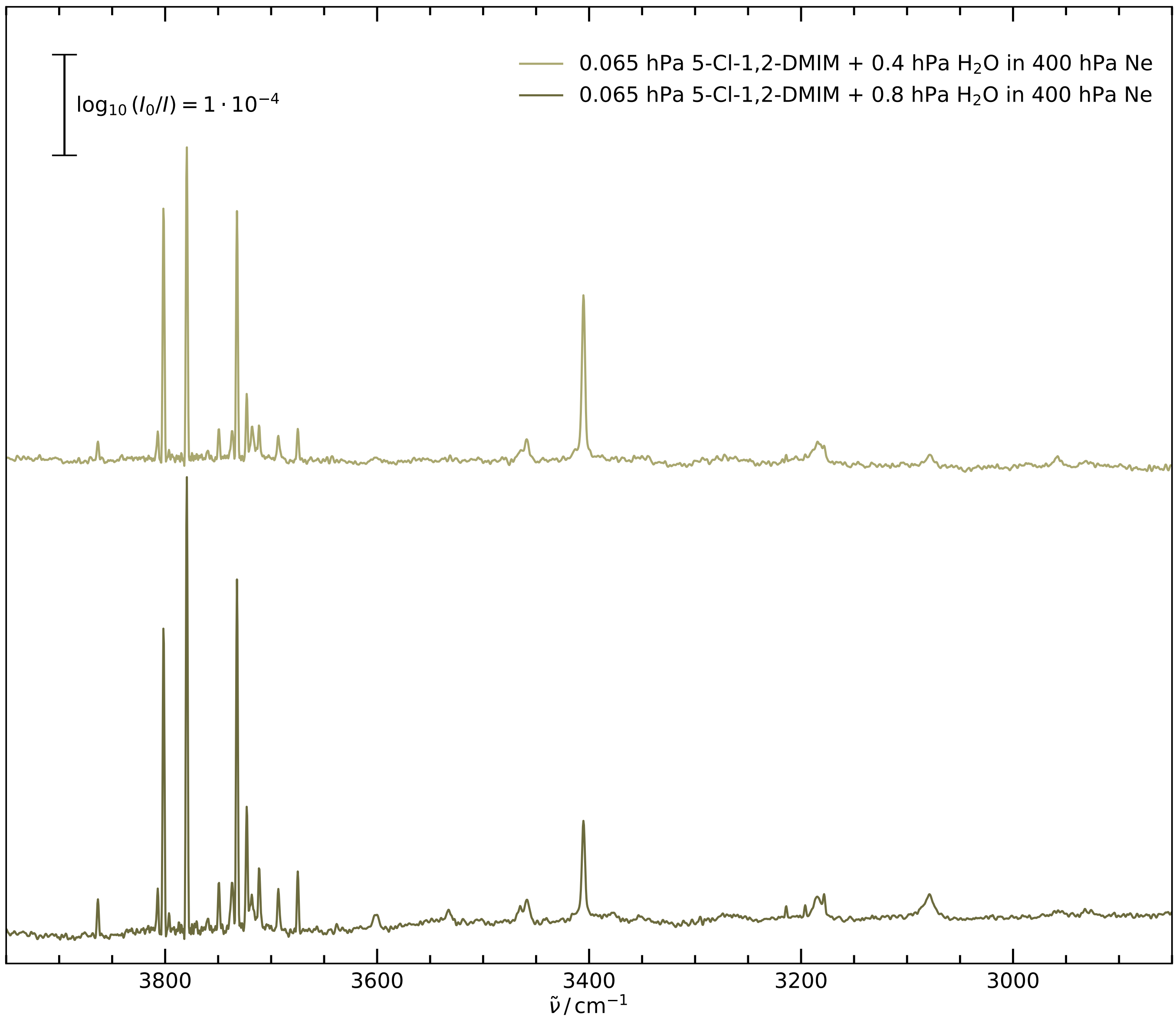


**Figure S22:** Overview FTIR spectra of 5-chloro-1,2-dimethylimidazole (5-Cl-1,2-DMIM) with water, which are shown in Fig. S21.

## 2.10 1,2-Dimethylimidazole (1,2-DMIM)

The spectra for 1,2-DMIM are presented in Figs. S23, S24 and S25.

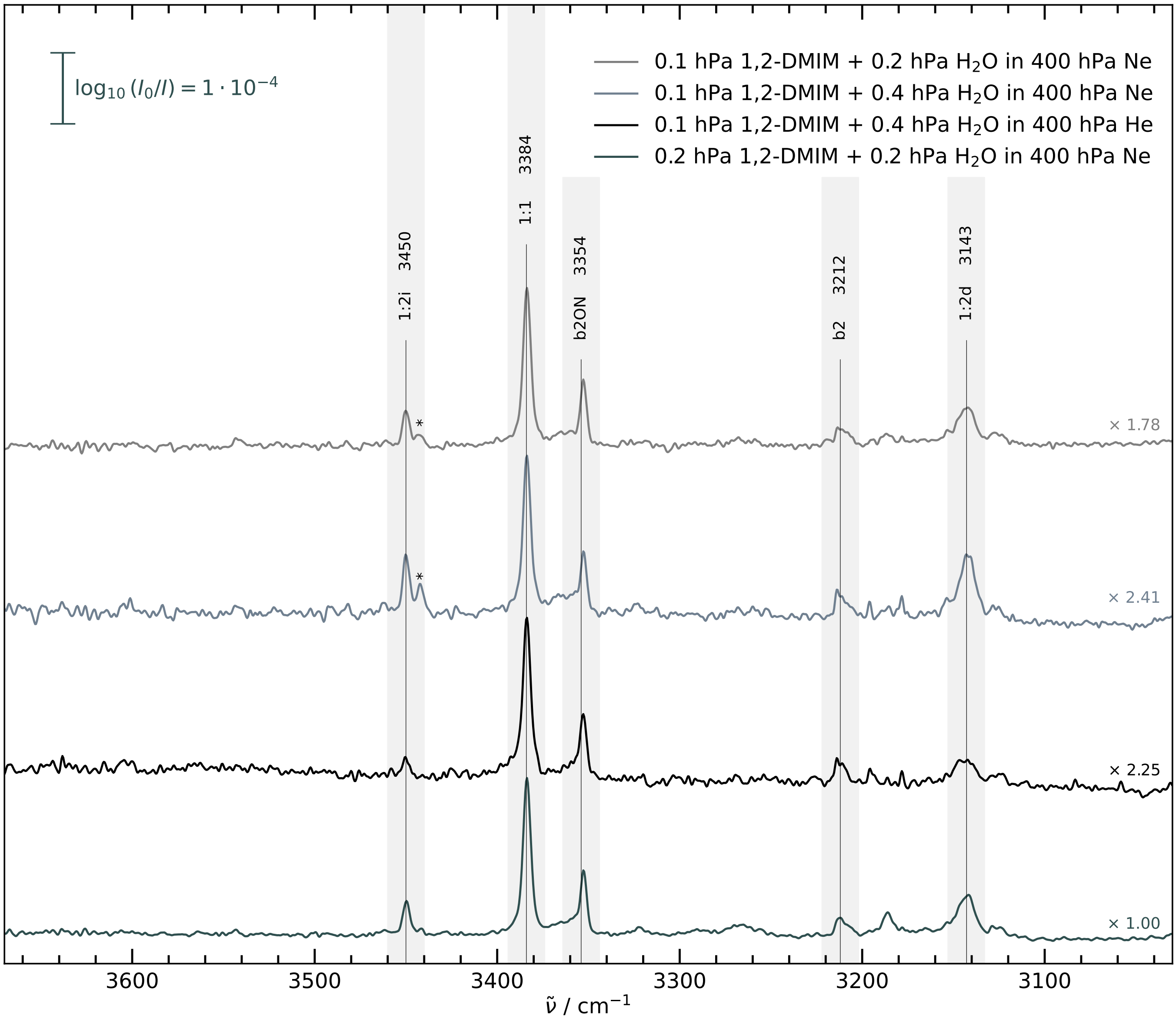


**Figure S23:** Experimental FTIR spectra of 1,2-dimethylimidazole (1,2-DMIM) with different concentrations of water. The monohydrate of 1,2-DMIM is denoted as 1:1 and the dihydrate as 1:2. In the dihydrate, 1:2i is the indirectly solvating water signal and the broad band 1:2d is for the directly contacting water. The bending overtone of water in the 1:1 complex is labeled as b2, while the combination band of bending overtone b2 with ON stretching fundamental is denoted as b2ON. The experimental wavenumbers are provided after each label. The spectra are scaled on the monohydrate, and the scaling factors are marked horizontally. The spectrum (black) with helium as carrier gas has fewer clusters compared to others. The signal at 3442 cm$^{-1}$ marked * in the two topmost spectra is a contamination from the previous measurement of 1-MIM.

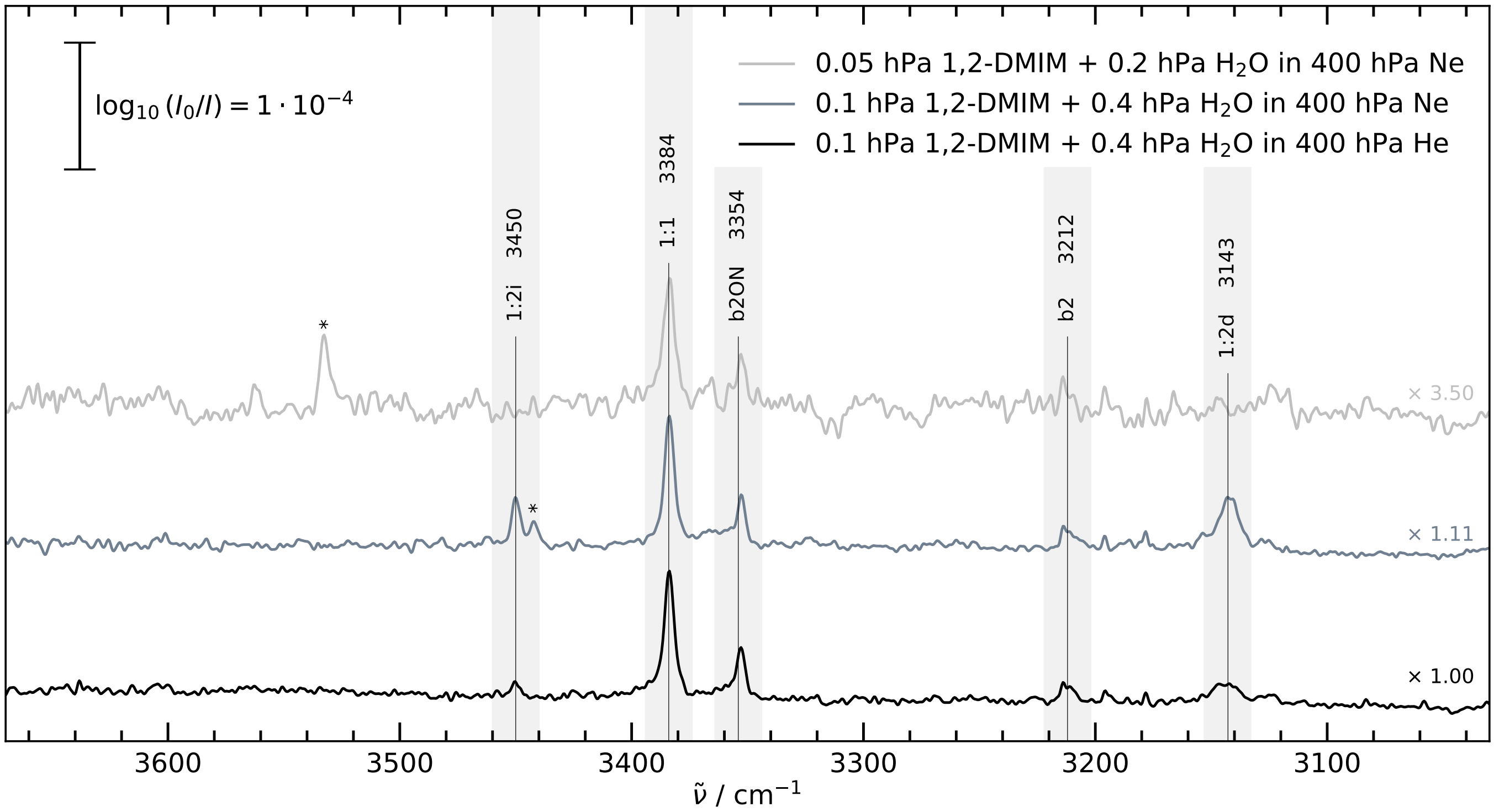


**Figure S24:** Experimental FTIR spectra of 1,2-dimethylimidazole (1,2-DMIM) with water for different carrier gas: helium (He) and neon (Ne). The monohydrate of 1,2-DMIM is denoted as 1:1 and the dihydrate 1:2. In the dihydrate, 1:2i is the indirectly solvating water signal and the broad band 1:2d is for the directly contacting water. The bending overtone of water in the 1:1 complex is labeled as b2, while the combination band of bending overtone b2 with ON stretching fundamental is denoted as b2ON. The experimental wavenumbers are provided after each label. The spectra are scaled on the monohydrate, and the scaling factors are marked horizontally. The spectrum (black) with helium as carrier gas has fewer clusters compared to other. The signal at 3534 cm$^{-1}$ marked * for the low concentration Ne expansion (top trace) is a contamination from the previous measurement of 1-piperidineethanol. The signal at 3442 cm$^{-1}$ marked * in the middle trace is a contamination from the previous measurement of 1-MIM.

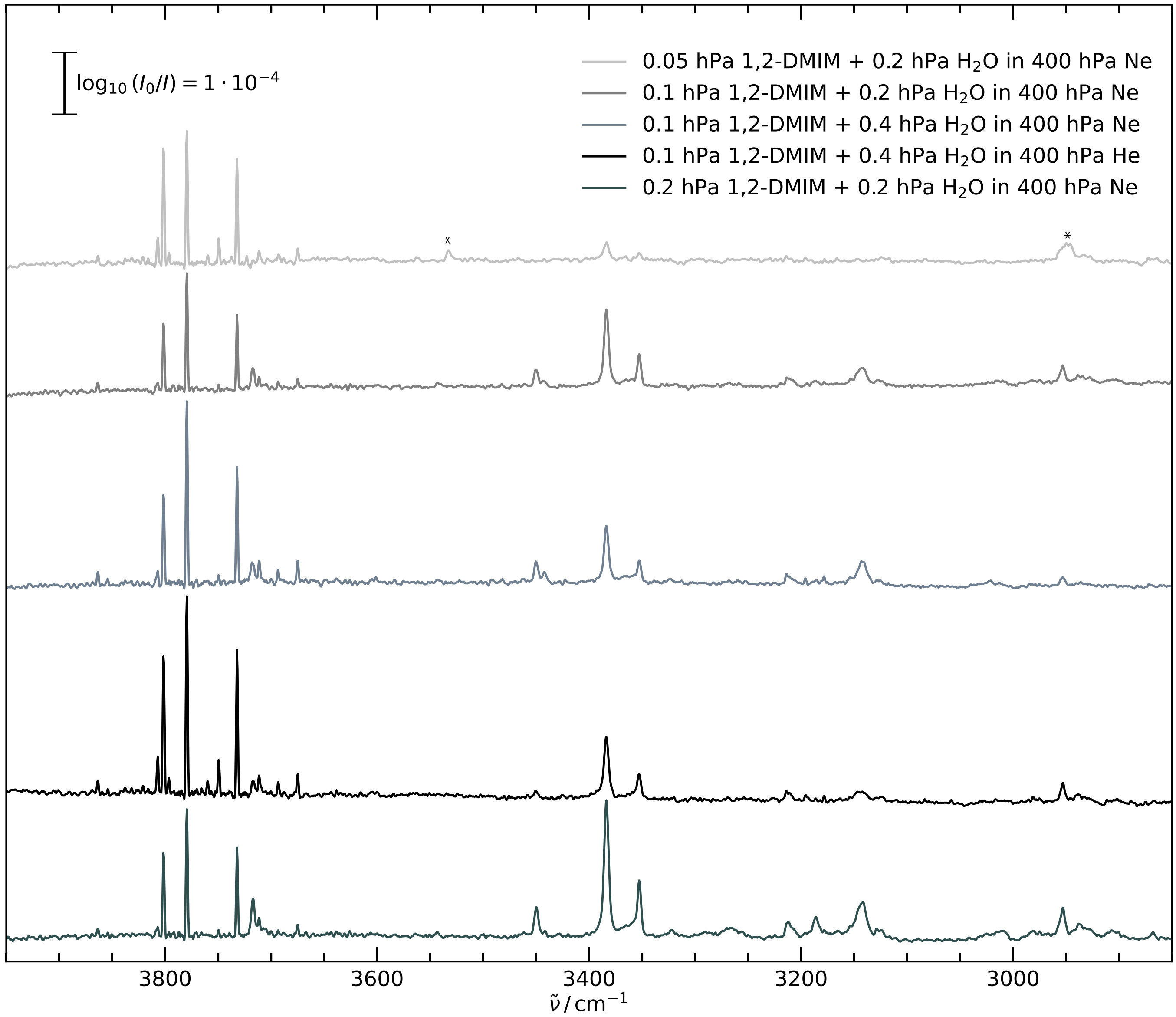


**Figure S25:** Overview FTIR spectra of 1,2-dimethylimidazole (1,2-DMIM) with water, which are shown in Fig. S23 and S24. The two signals marked * for the low concentration Ne expansion (top trace) are contaminations from the previous measurement of 1-piperidineethanol. The central spectrum shows less N-heterocycle signal than expected, perhaps due to stronger complexation or wall condensation at higher water partial pressure.

### 2.11 1,2,4-Trimethylimidazole (1,2,4-TMIM)

The spectra for 1,2,4-TMIM are presented in Figs. S26 and S27.

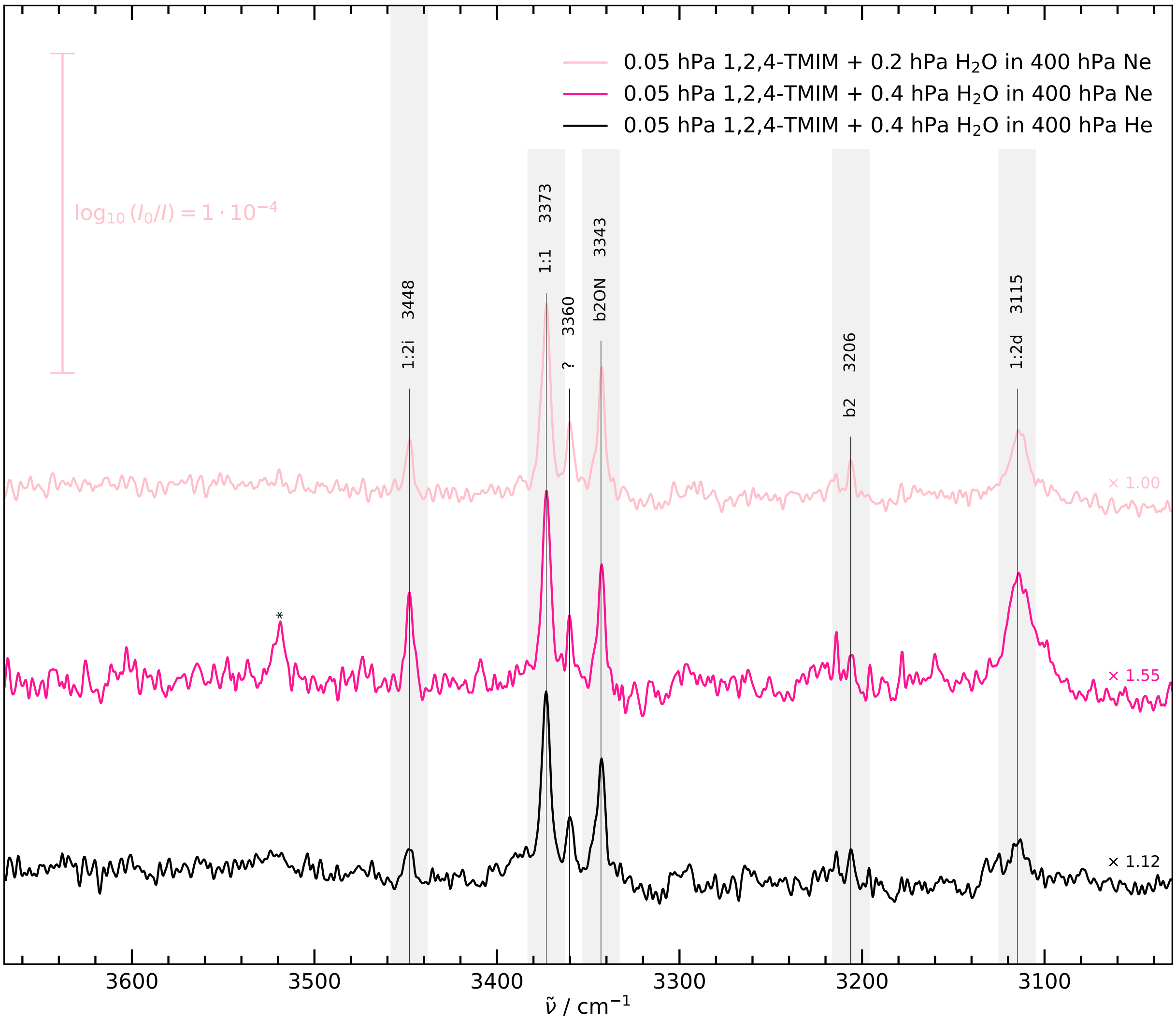


**Figure S26:** Experimental FTIR spectra of 1,2,4-trimethylimidazole (1,2,4-TMIM) with different concentrations of water. The monohydrate of 1,2,4-TMIM is denoted as 1:1 and the dihydrate as 1:2. In the dihydrate, 1:2i is the indirectly solvating water signal and the broad band 1:2d is for the directly contacting water. The bending overtone of water in the 1:1 complex is labeled as b2, while the combination band of bending overtone b2 with ON stretching fundamental is denoted as b2ON. The experimental wavenumbers are provided after each label. The spectra are scaled on the monohydrate, and the scaling factors are marked horizontally. The spectrum (black) with helium as carrier gas has fewer clusters compared to others. Different explanations for the small signal at 3360 cm$^{-1}$ were explored, including a general impurity, harmonic mixing of ON with a scaffold mode, an imidazole regioisomer, and a water positional isomer. No strong evidence was found for either of these explanations. Even if it is a local anharmonic resonance with a symmetry-consistent dark state (such as b2 with a torsional overtone of the free OH group), it is so close to the intensity centroid of 1:1 and b2 that its influence on the deperturbed band position is predicted to be small. The signal marked * near 3520 cm$^{-1}$ in the middle trace is due to the alcoholic OH stretch in the monohydrate of a small 1-pyrrolidineethanol impurity.

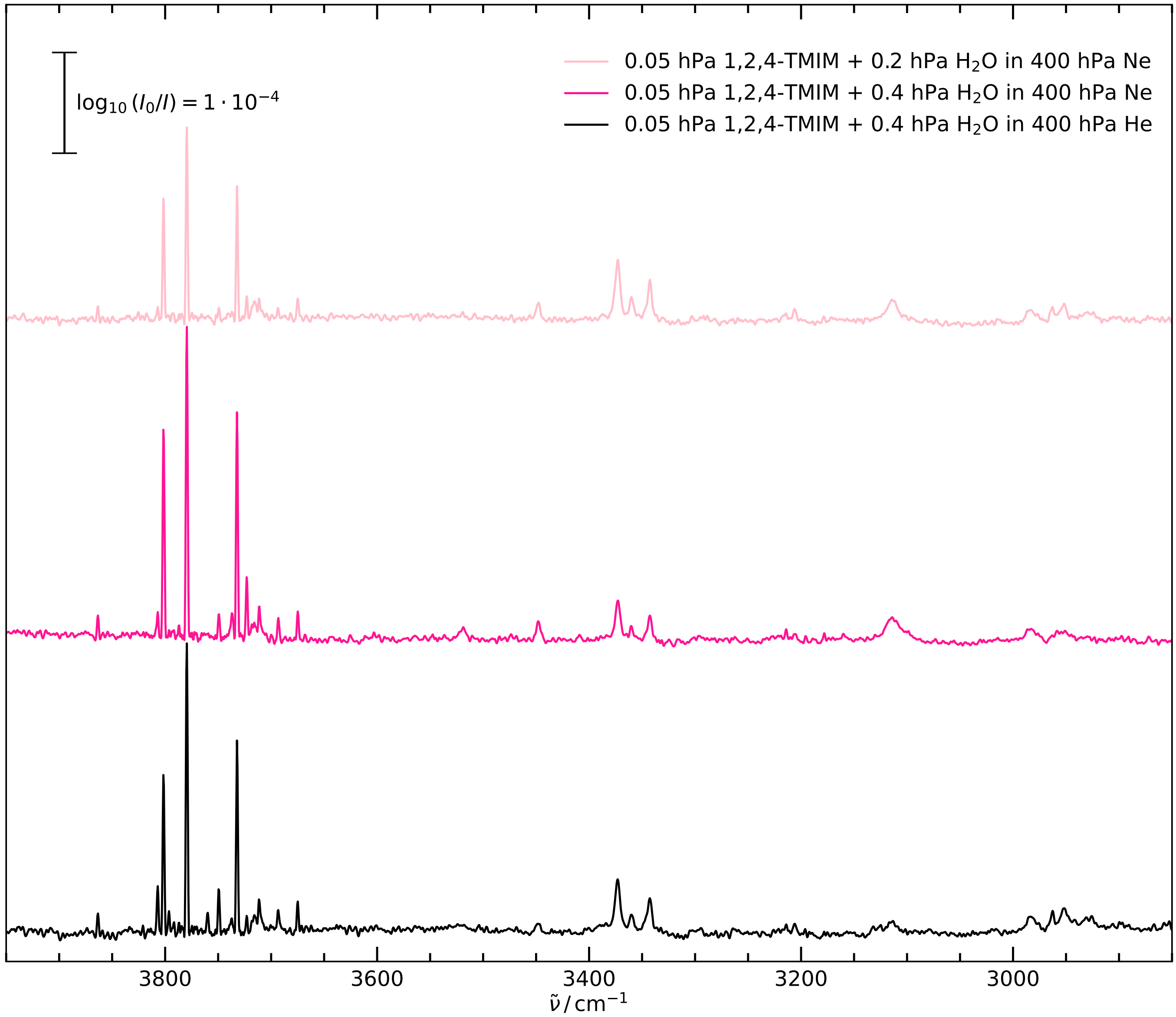


**Figure S27:** Overview FTIR spectra of 1,2,4-trimethylimidazole (1,2,4-TMIM) with water, which are shown in Fig. S26.

## 2.12 1,2,4,5-Tetramethylimidazole (1,2,4,5-TeMIM)

The spectra for 1,2,4,5-TeMIM are presented in Figs. S28 and S29.

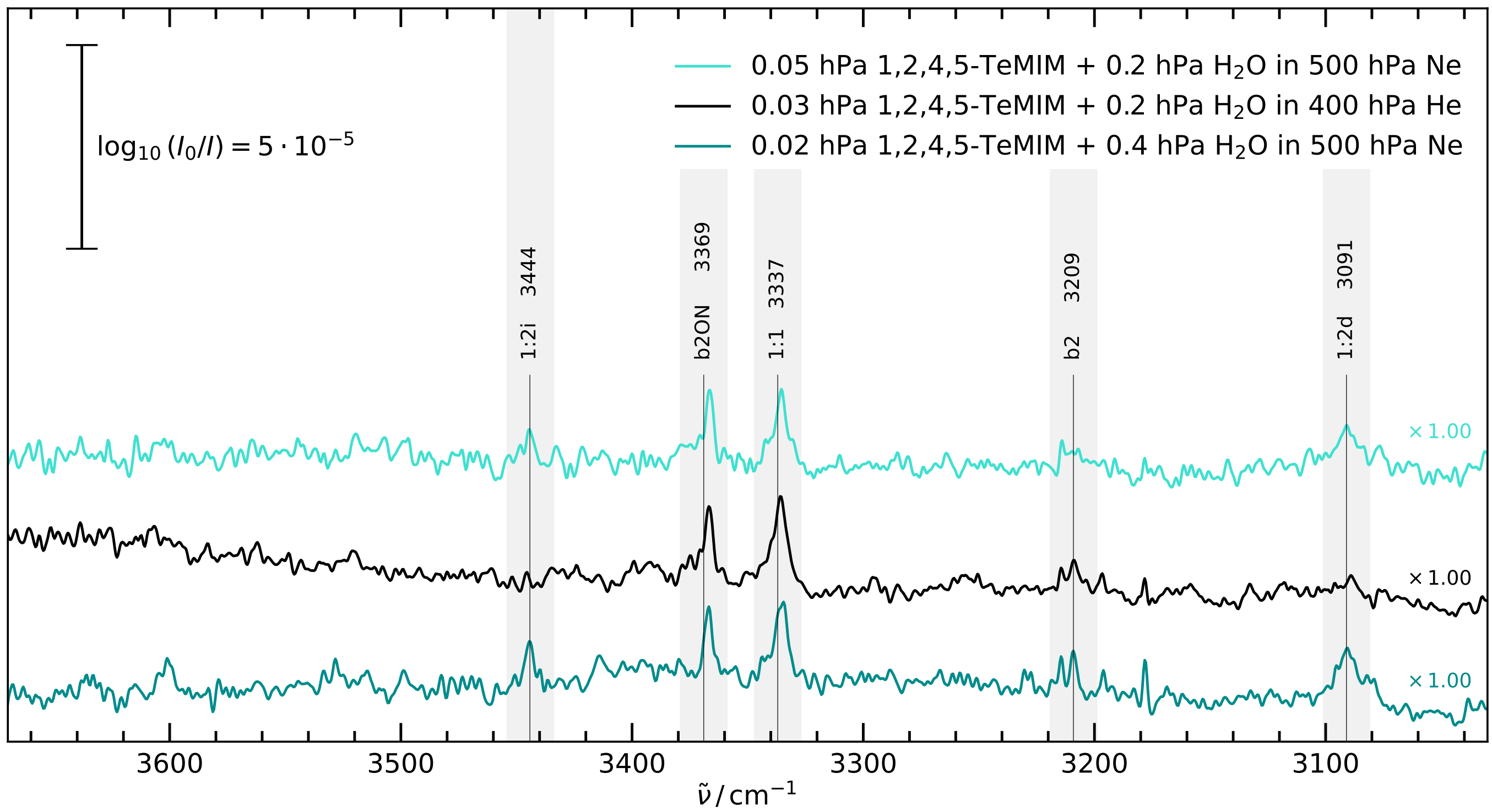


**Figure S28:** Experimental FTIR spectra of 1,2,4,5-tetramethylimidazole (1,2,4,5-TeMIM) with different concentrations of water. The monohydrate of 1,2,4,5-TeMIM is denoted as 1:1 and the dihydrate as 1:2. In the dihydrate, 1:2i is the indirectly solvating water signal and the broad band 1:2d is for the directly contacting water. Overlap with CH signals is unlikely, because no aromatic CH groups are left. The bending overtone of water in the 1:1 complex is labeled as b2, while the combination band of bending overtone b2 with ON stretching fundamental is denoted as b2ON. The experimental wavenumbers are provided after each label. The spectrum (black) with helium as carrier gas has fewer clusters compared to others.

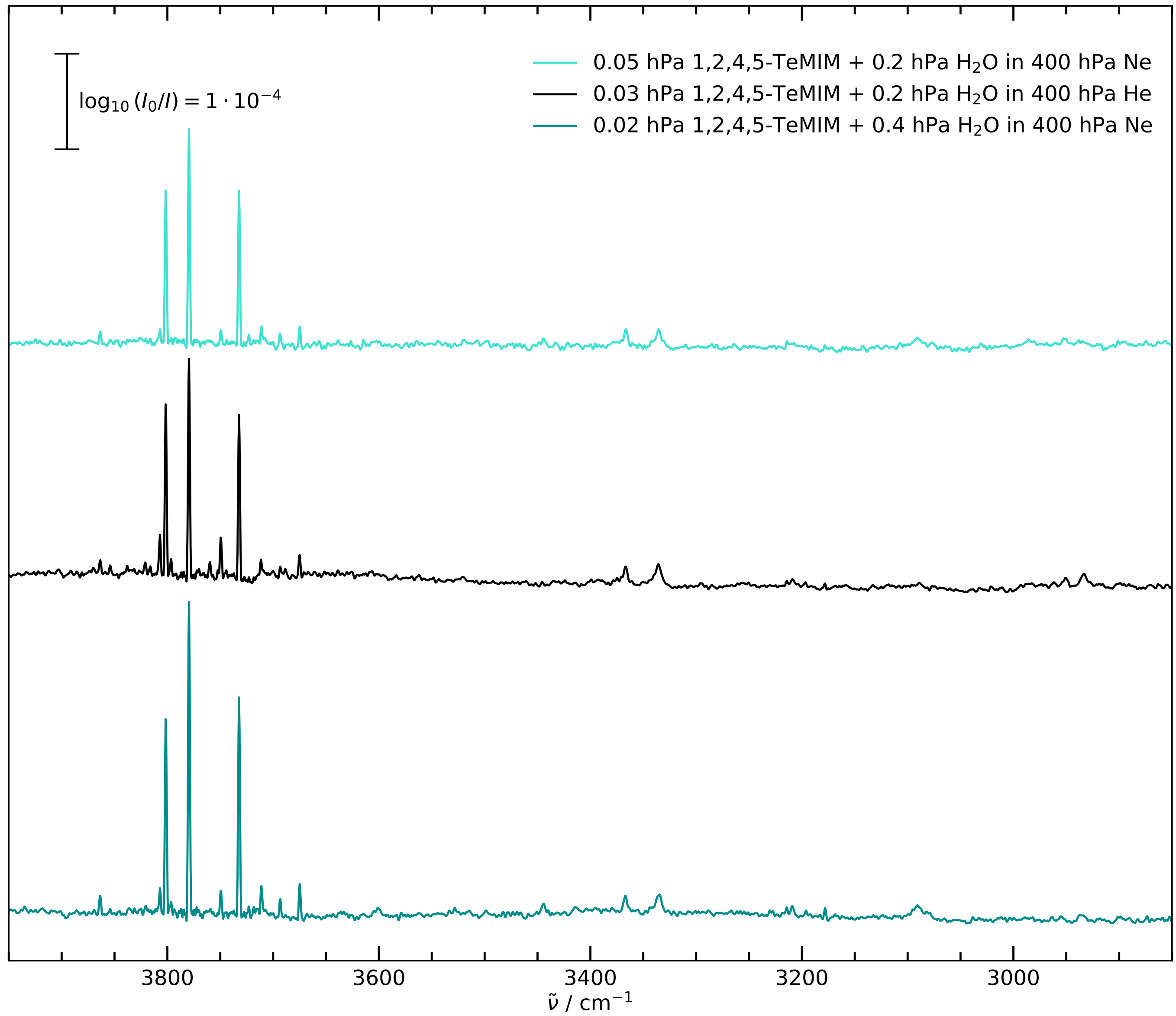


**Figure S29:** Overview FTIR spectra of 1,2,4,5-tetramethylimidazole (1,2,4,5-TeMIM) with water, which are shown in Fig. S28. The CH signals do not correlate well with the nominal heterocycle concentration, probably there is some co-condensation with water on the walls.

### 2.13 Summary

Table S8 summarizes the tentative (?) and robust spectral assignments for the mono- and dihydrates of the N-heterocycles.

**Table S8:** Summary of the experimentally observed wavenumbers (in $cm^{-1}$, centroids in asymmetric cases, estimated uncertainties in parentheses) of the investigated complexes.

| system | 1:1 | 1:2d | 1:2i | b2 (1:1) | b2 (1:2d) | b2ON (1:1) |
|---|---|---|---|---|---|---|
| 4-Cl-1-MPY | 3483(1) | 3371(2) | 3492(2) | 3204(5)? | 3204(5)? | |
| 4-Br-1-MPY | 3483(1) | 3370(2) | 3492(2) | 3204(5)? | 3204(5)? | |
| 1-MPY | 3457(1) | 3317(2) | 3482(2) | 3202(5)? | 3202(5)? | |
| 1,4-DMPY | 3447(1) | 3305(3) | 3479(2) | 3204(5) | 3204(4) | |
| 1,3-DMPY | 3439(1) | 3286(3) | 3475(2) | 3203(5) | 3198(4) | |
| 1,3,5-TMPY | 3415(1) | 3191(3) | 3467(2) | 3205(5) | 3271(4) | |
| | | [3271(3)] | | | [3191(4)] | |
| 1-MIM | 3442(2) | 3179(5) | 3448(3) | 3208(5) | | |
| 5-Cl-1,2-DMIM | 3405(1) | 3186(5) | 3459(3) | 3204(5) | | |
| 1,2-DMIM | 3384(1) | 3143(5) | 3450(2) | 3212(5) | | 3354(3) |
| 1,2,4-TMIM | 3373(1) | 3115(5) | 3448(2) | 3206(5) | | 3343(3) |
| 1,2,4,5-TeMIM | 3337(3) | 3091(5) | 3444(2) | 3209(5) | | 3369(3) |

For the further processing of the spectral information, it is useful to have standardized estimates of uncertainties. For strong and sharp transitions (typically OHb in 1:1 complexes), we assume that the uncertainty is comparable to the instrumental resolution, $\pm 1\,cm^{-1}$. For weaker satellite peaks, overlap situations, shoulders, broader signals and generally for dihydrates, we assume an uncertainty of at least $\pm 2\,cm^{-1}$, sometimes also $\pm 3\,cm^{-1}$. For b2 bands which are often broad, weak, and believed to consist of several overlapping cluster compositions, we assume an uncertainty of $\pm 5\,cm^{-1}$ but when a question mark is given, it is uncertain whether there is a signal at all. In cases of anharmonic mixing between OHb and b2, the uncertainties are mixed as well, see section 4. For OHb bands below b2, which are typically broad, we also assume an uncertainty of $\pm 5\,cm^{-1}$.

## 3 Harmonic modeling

### 3.1 Details on harmonic calculations

The molecular structures of all investigated compounds (1-MPY, 1,3-DMPY, 1,4-DMPY 1,3,5-TMPY, 4-Cl-1-MPY, 4-Br-1-MPY, 1-MIM, 1,2-DMIM, 1,2,4-TMIM, 1,2,4,5-TeMIM) were optimized by using the three-body-inclusive D3-dispersion correction[10–14] with B3LYP[15–17] and B2PLYP[18] using ORCA version 5.0.3[19–21] with the keywords D3BJ, ABC, SlowConv, DEFGRID3, VERYTIGHTSCF, VERYTIGHTOPT, FREQ, mass2016. For B3LYP-D3BJ the basis sets def2-TZVP[22] (TZ), ma-def2-TZVP[22,23] (maTZ) and def2-QZVP[22] (QZ) were used, while optimizations using B2PLYP-D3BJ[18] were done with the basis set def2-QZVP[22] (B2QZ).
The initial structures of the clusters were drawn using Chemcraft version 1.8 and then sampled using the GFN2-xTB method[24,25] in CREST[26,27]. The gained structures were preoptimized at B3LYP-D3BJ / def2-TZVP level. The lowest energy conformers were then reoptimized using the mentioned higher levels of computation maTZ, QZ and B2QZ.

### 3.2 Example input

For an example input, see Table S9.

**Table S9:** Example input for geometry optimizations and harmonic frequency calculations using B3LYP-D3BJ def2-TZVP (TZ).

| type of calculation | input |
|---|---|
| Geometry optimizations &<br>Harmonic frequency calculations | !B3LYP D3BJ ABC def2-TZVP SlowConv defgrid3<br>VeryTightSCF VeryTightOpt FREQ Mass2016<br>%geom<br>MaxIter 500<br>ENFORCESTRICTCONVERGENCE true<br>end<br>%scf<br>MaxIter 1500<br>end<br>%pal nprocs 6 end<br>%Maxcore 4000<br>xyzfile 0 1 axstart.xyz |

## 3.3 Minimum structures

### 3.3.1 1:1 complexes

A survey over the pyrazole monohydrate structures is given in Fig. S30, the imidazole monohydrates are shown in Fig. S31. The hydrogen bond length correlates qualitatively with the predicted harmonic OHb wavenumber. The shorter the hydrogen bond, the stronger its redshift. The out-of-plane tilt of the free OH group of the water molecule increases with ring CH methylation in pyrazoles, but not in imidazoles.

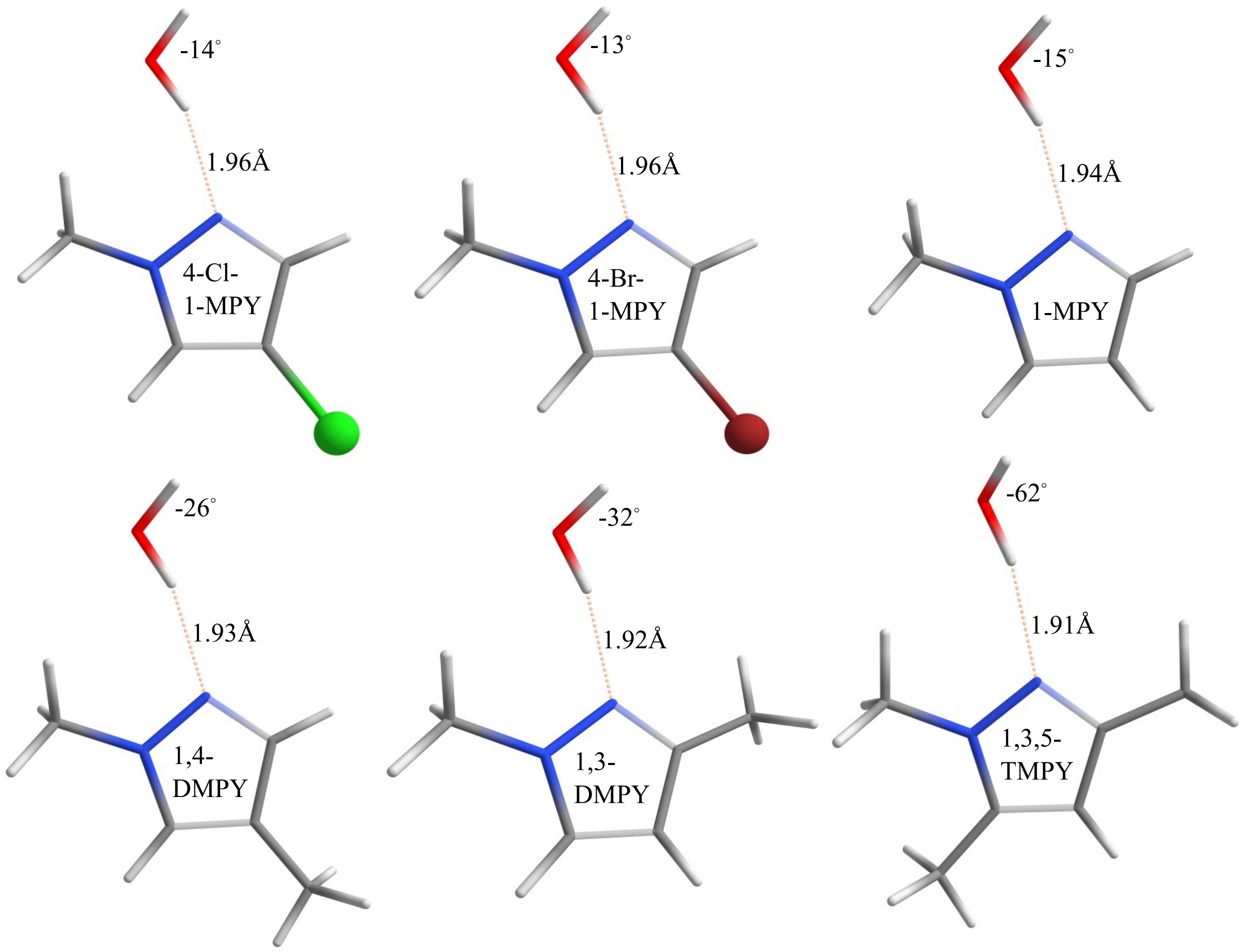


**Figure S30:** Optimized structures of six pyrazole monohydrates: 4-Cl-1-MPY, 4-Br-1-MPY, 1-MPY, 1,4-DMPY, 1,3-DMPY and 1,3,5-TMPY at B2PLYP-D3BJ/def2-QZVP (B2QZ) level. Also shown are hydrogen bond lengths (in Å) as well as torsional angles (in degrees) of the free OH group relative to plane spanned by O, N and the adjacent ring C-atom. To illustrate the basis set dependence of the torsional angles, we also provide the other computed values for 1-MPY: TZ: -36 °, maTZ: -17 °, QZ: -26 °.

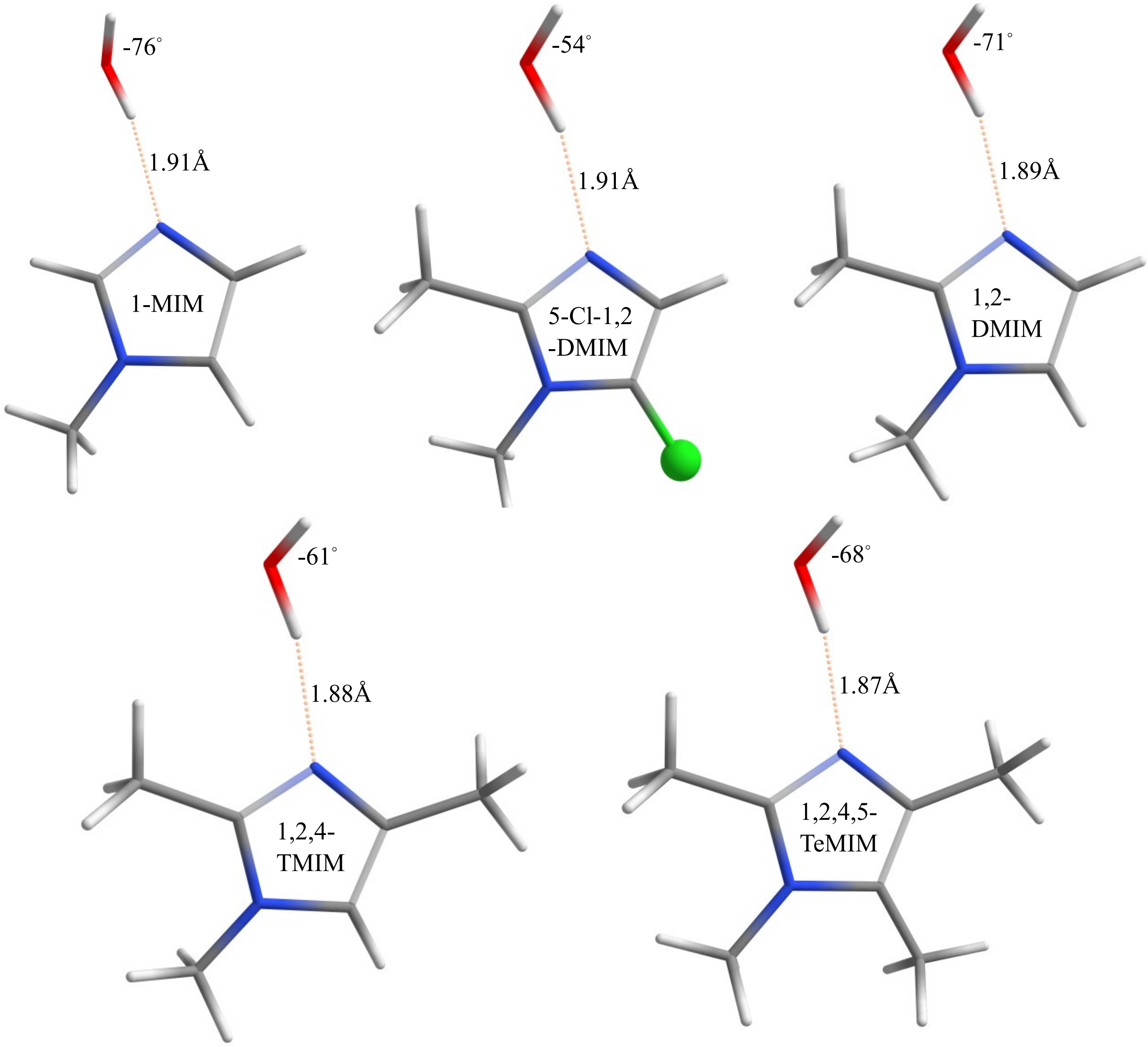


**Figure S31:** Optimized structure of five imidazole monohydrates: 1-MIM, 5-Cl-1,2-DMIM, 1,2-DMIM, 1,2,4-TMIM and 1,2,4,5-TeMIM at B2PLYP-D3BJ/def2-QZVP (B2QZ) level. Also shown are hydrogen bond lengths (in Å) as well as torsional angles (in degrees) of the free OH group relative to plane spanned by O, N and the adjacent ring C-atom. To illustrate the basis set dependence of the torsional angles, we also provide the other computed values for 1-MIM: TZ: -78°, maTZ: -67°, QZ: -76°.

### 3.3.2 1:2 complexes

A survey over the most stable pyrazole dihydrate structures is given in Fig. S32, the imidazole dihydrates are shown in Fig. S33.

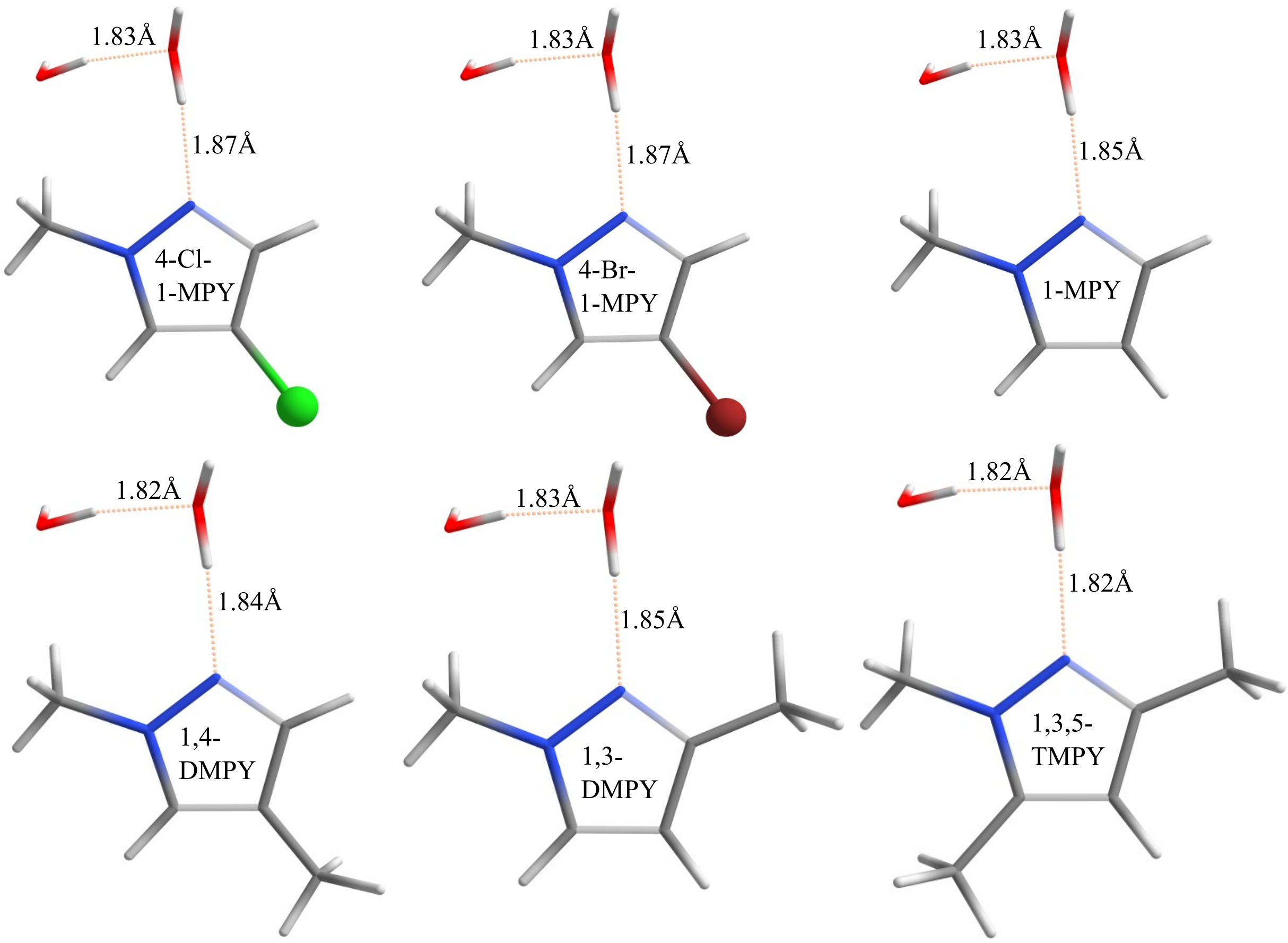


**Figure S32:** Optimized structures of six pyrazole dihydrates: 4-Cl-1-MPY, 4-Br-1-MPY, 1-MPY, 1,4-DMPY, 1,3-DMPY and 1,3,5-TMPY at B2PLYP-D3BJ/def2-QZVP (B2QZ) level. Also shown are hydrogen bond lengths (in Å) for both OH bonds (1:2i and 1:2d). For 1:2i, the hydrogen bond length is taken as OH. . . O, and it is taken as OH. . . N for 1:2d. Note that the 1:2d hydrogen bond lengthens with ring halogenation and shortens with ring methylation.

The relative energy of the second-lowest identified dihydrate for the investigated pyrazoles and imidazoles is listed in Table S10. In several cases, it is below 2 kJ mol$^{-1}$, indicating a possible survival in the supersonic jet expansion given a sufficiently large interconversion barrier. As there is no strong evidence for competing isomers in the observed spectra, it is plausible that they are separated from the global minimum structures by shallow barriers. Indeed, in most cases the spectral shifts are large enough to be spectrally distinct, but relatively small. A prominent exception is 5-Cl-1,2-DMIM, where the Cl substituent is more directly involved in the hydrogen bond interaction (Fig. S34).

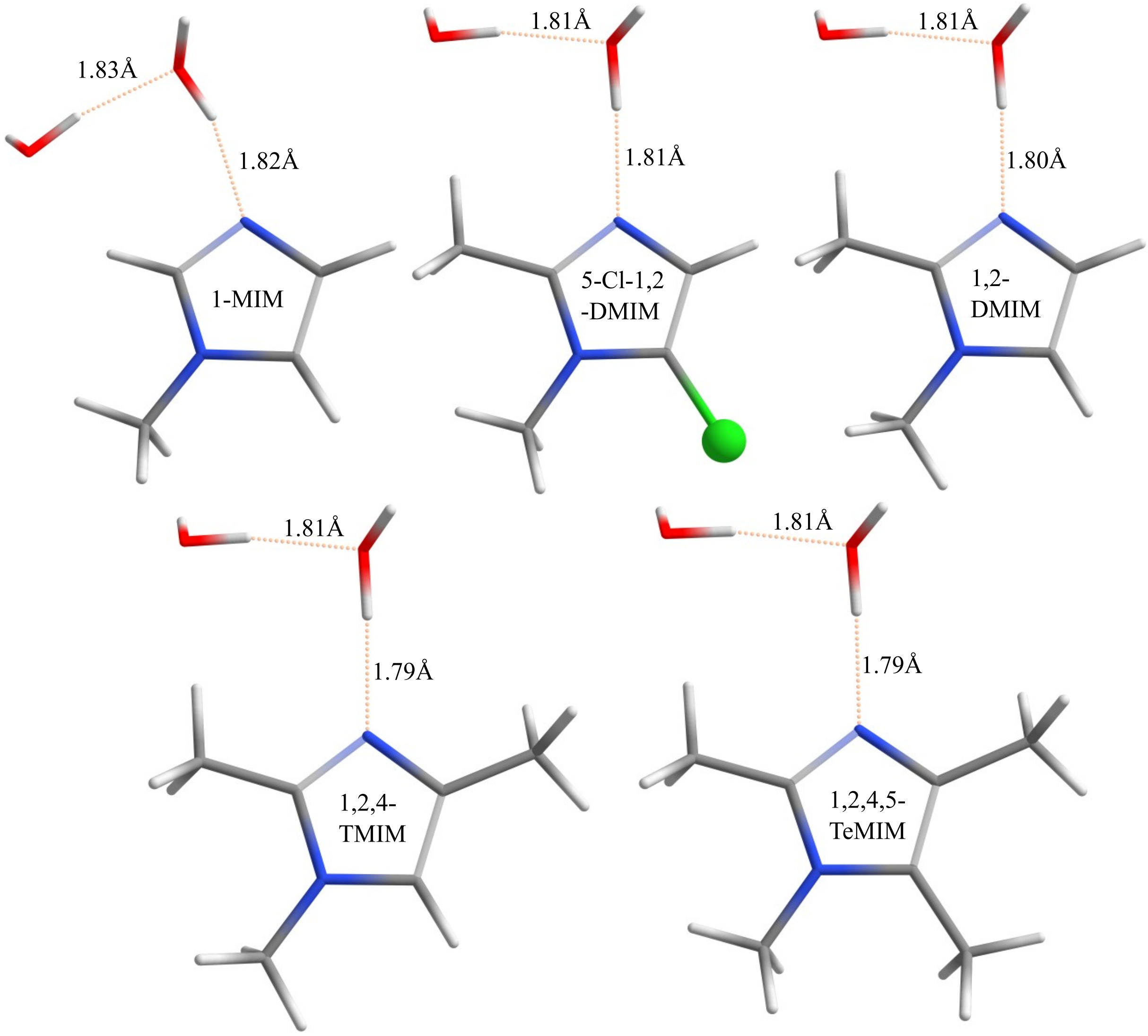


**Figure S33:** Optimized structures of five imidazole dihydrates: 1-MIM, 5-Cl-1,2-DMIM, 1,2-DMIM, 1,2,4-TMIM and 1,2,4,5-TeMIM at B2PLYP-D3BJ/def2-QZVP (B2QZ) level. Also shown are hydrogen bond lengths (in Å) for both OH bonds (1:2i and 1:2d). For 1:2i, the hydrogen bond length is taken as OH. . . O, and it is taken as OH. . . N for 1:2d. Note that the 1:2d hydrogen bond contracts with increasing ring methylation, whereas the 1:2i hydrogen bond contracts in the presence of a methyl group in $\alpha$ position, which allows for a cooperative CH-OH-OH-N hydrogen bond chain.

**Table S10:** Calculated energy $\Delta E_0$ of the lowest metastable isomer relative to the global minimum structure of the investigated dihydrates at TZ level. Spectral shifts $\Delta\omega$ in OHb wavenumber are also provided. A positive $\Delta\omega$ means that the metastable isomer absorbs at higher wavenumber.

| system | $\Delta E_0$ / kJ mol$^{-1}$ | $\Delta\omega$(1:2i) / cm$^{-1}$ | $\Delta\omega$(1:2d) / cm$^{-1}$ |
|---|---|---|---|
| 4-Cl-1-MPY | 1.4 | −6 | 9 |
| 4-Br-1-MPY | 1.4 | −5 | 10 |
| 1-MPY | 2.2 | 35 | 14 |
| 1,4-DMPY | 1.3 | −5 | 10 |
| 1,3-DMPY | 1.8 | −4 | 20 |
| 1,3,5-TMPY | 5.3 | 36 | −10 |
| 1-MIM | 6.6 | 32 | 24 |
| 5-Cl-1,2-DMIM | 1.8 | 54 | 150 |
| 1,2-DMIM | 2.0 | 43 | 28 |
| 1,2,4-TMIM | 1.9 | 39 | 27 |
| 1,2,4,5-TeMIM | 2.0 | 45 | 26 |

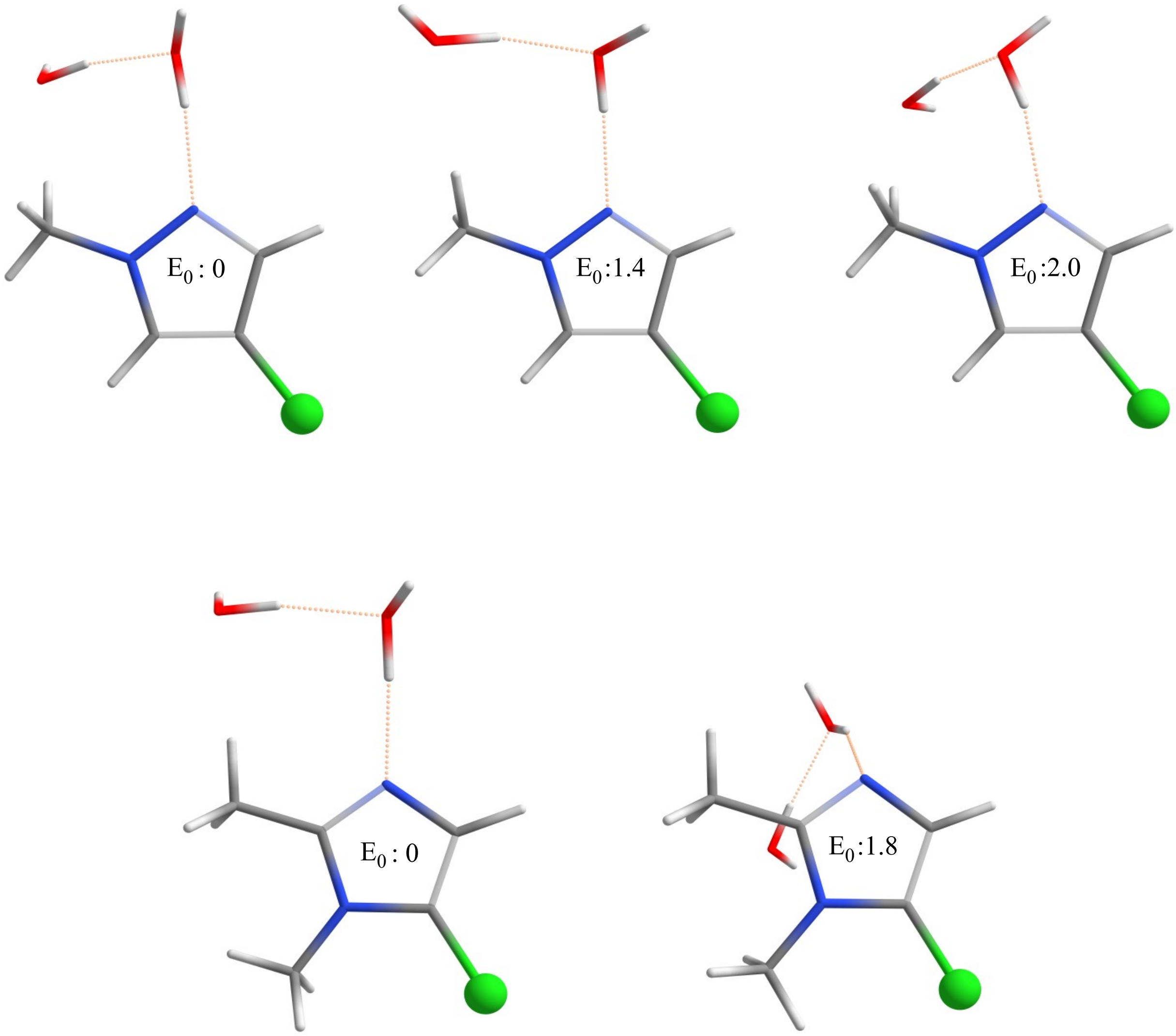


**Figure S34:** Optimized structures of low-lying isomers of 4-Cl-1-MPY dihydrate and 5-Cl-1,2-DMIM dihydrate at B3LYP-D3BJ/def2-TZVP (TZ) level. The structure of the (unobserved) metastable 5-Cl-1,2-DMIM dihydrate explains why its spectrum is predicted to differ substantially from the observed global minimum structure.

## 3.4 Harmonic predictions

An advantage of four different harmonic computational levels for the mono- and dihydrates of the 11 pyrazoles and imidazoles is a better judgement of the robustness of these predictions. Therefore, the Tables S11, S14, and S15 summarize the predictions for OHb, whereas Tables S12, S13, S16, S17, and S18 provide the corresponding information for resonance partners.

### 3.4.1 1:1 complexes

For 1:1 complexes, the results in Tables S11 and S12 can be correlated with each other to see how the harmonic b2 wavenumber changes with the OHb wavenumber. As a very rough approximation, it has been proposed [28] that $5\,\mathrm{cm}^{-1}$ downshift in OHb result in about $1\,\mathrm{cm}^{-1}$ upshift in b2, based on TZ calculations. Fig. S35 shows that this is approximately the case for pyrazole and imidazole monohydrates as well. Such a weak trend will underly any Fermi resonance interaction between the two states. The correlation between the hydrogen bond-induced water OHb wavenumber lowering and the stiffening of the water bend overtone for the other DFT approaches used in this work is illustrated in Figs. S36, S37 and S38. Here, the slope is reduced to about $10\,\mathrm{cm}^{-1}$ downshift in OHb for $1\,\mathrm{cm}^{-1}$ upshift in b2. For the analogous correlation of the harmonic b2ON prediction with OHb, the slopes are systematically steeper and can be approximated by $4\,\mathrm{cm}^{-1}$ downshift in OHb for $1\,\mathrm{cm}^{-1}$ upshift in b2 (TZ, Fig. S39) and $5\,\mathrm{cm}^{-1}$ downshift in OHb for $1\,\mathrm{cm}^{-1}$ upshift in b2 (maTZ in Fig. S40, QZ in Fig. S41, B2QZ in Fig. S42).

**Table S11:** Harmonically predicted wavenumbers for the monohydrate (1:1) OHb stretching vibration at different levels of computation. The values given in brackets reflect the predicted shift relative to 1-MPY. Note that the dependence of the substitution effects on the basis set or method is significantly more pronounced for imidazoles ($\leq 15\,\mathrm{cm}^{-1}$) than for pyrazoles ($\leq 5\,\mathrm{cm}^{-1}$). In part, this is due to 1-MIM being the only binding partner (see Fig. S31) where the solvating water has no secondary contact to a neighboring methyl group. The basis set/method sensitivity of imidazole substitution effects excluding 1-MIM is more in line with those predicted for pyrazoles. The first line gives the harmonically predicted value of the symmetric stretching vibration of water without binding partner.

| system | TZ / $\mathrm{cm}^{-1}$ | maTZ / $\mathrm{cm}^{-1}$ | QZ / $\mathrm{cm}^{-1}$ | B2QZ / $\mathrm{cm}^{-1}$ |
|---|---|---|---|---|
| - | 3782 | 3779 | 3807 | 3839 |
| 4-Cl-1-MPY | 3561 [29] | 3544 [33] | 3573 [31] | 3621 [29] |
| 4-Br-1-MPY | 3563 [31] | 3545 [34] | 3574 [32] | 3622 [30] |
| 1-MPY | 3532 [0] | 3511 [0] | 3542 [0] | 3592 [0] |
| 1,4-DMPY | 3522 [−10] | 3499 [−12] | 3531 [−11] | 3581 [−11] |
| 1,3-DMPY | 3513 [−19] | 3490 [−21] | 3522 [−20] | 3573 [−19] |
| 1,3,5-TMPY | 3486 [−46] | 3462 [−49] | 3494 [−48] | 3548 [−44] |
| 1-MIM | 3524 [−8] | 3483 [−28] | 3516 [−26] | 3569 [−23] |
| 5-Cl-1,2-DMIM | 3483 [−49] | 3456 [−55] | 3490 [−52] | 3542 [−50] |
| 1,2-DMIM | 3455 [−77] | 3423 [−88] | 3460 [−82] | 3513 [−79] |
| 1,2,4-TMIM | 3438 [−94] | 3405 [−106] | 3442 [−100] | 3497 [−95] |
| 1,2,4,5-TeMIM | 3425 [−107] | 3390 [−121] | 3427 [−115] | 3484 [−108] |

### 3.4.2 Harmonic prediction performance for the b2ON resonance

The four explored computational models can be tested with respect to their ability to systematically predict the OHb/b2ON resonance condition across the investigated systems (Fig. S43). Because of the harmonic approximation, one has to allow for a systematic offset between the harmonic resonance prediction and the experimental one, even for a hypothetically perfect density functional. This offset is determined by plotting the harmonic predictions for OHb vs. those for b2ON with symbol size proportional to the resonance extent (the symbol size is chosen proportional to the fraction of intensity transfer from OHb, with an intensity offset of 10% of the maximum possible intensity transfer to make non-resonant cases visible). A perfect computational model would show large symbols close to the diagonal. The required wavenumber offset for the individual computational models is obtained by finding the off-diagonal ribbon of slope 1 and width $\pm W$ which best covers the three systems which experimentally show significant resonance, including a switch in OHb/b2ON wavenumber sequence. This offset ranges from 30 (B2QZ) to $69\,\mathrm{cm}^{-1}$ (QZ). For water monomer, the anharmonic offset is about $175-146\,\mathrm{cm}^{-1}$ or $29\,\mathrm{cm}^{-1}$ (https://cccbdb.nist.gov/exp2x.asp?casno=7732185&charge=0, http://dx.doi.org/10.1098/rsta.2017.0149) based on the symmetric stretch and the bending overtone alone, and it is plausible that ON in the 1:1 complex further reduces this value due to its anharmonicity. Therefore, B2QZ is closest to expectations, with the smallest underlying DFT wavenumber offset among the four levels.

**Table S12:** Harmonically predicted wavenumbers for the water bending overtone b2 of the monohydrate (1:1) at different levels of computation. The values given in brackets reflect the predicted shift relative to 1-MPY. The dependence of the substitution effects on the basis set is comparable to the substitution effects themselves. The first line gives the harmonically predicted value of the bending overtone of water without binding partner. Data pairs in parentheses provide original harmonic wavenumbers where a demixing of the water bend fundamental b was necessary.

| system | TZ / $cm^{-1}$ | maTZ / $cm^{-1}$ | QZ / $cm^{-1}$ | B2QZ / $cm^{-1}$ |
|---|---|---|---|---|
| - | 3233 | 3209 | 3260 | 3279 |
| 4-Cl-1-MPY | 3292 [−10] | 3278 [−11] | 3321 [−9] | 3340 [−10] |
| 4-Br-1-MPY | 3292 [−10] | 3278 [−11] | 3321 [−9] | 3340 [−10] |
| 1-MPY | 3302 [0] | 3289 [0] | 3330 [0] | 3350 [0] |
| 1,4-DMPY | 3303 [1] | 3289 [0] | 3330 [0] | 3351 [1] |
| 1,3-DMPY | 3300 [−2] | 3289 [0] | 3328 [−2] | 3349 [−1] |
| 1,3,5-TMPY | 3299 [−3] | 3279 [−10] | 3324 [−6] | 3343 [−7] |
| 1-MIM | 3310 [8] | 3277 [−12] | 3330 [0] | 3349 [−1] |
| 5-Cl-1,2-DMIM | 3308 [6] | 3285 [−4] | 3331 [1] | 3351 [1] |
| 1,2-DMIM | 3316 [14] | 3292 [3] | 3336 [6] | 3358 [8] |
| 1,2,4-TMIM | 3315 [13] | 3292 [3] | 3336 [6] | 3357 [7] |
| 1,2,4,5-TeMIM | 3319 [17] | 3293 [4] | 3338 [8] | 3356 [6] |
| | | (3285 / 3300) | | |

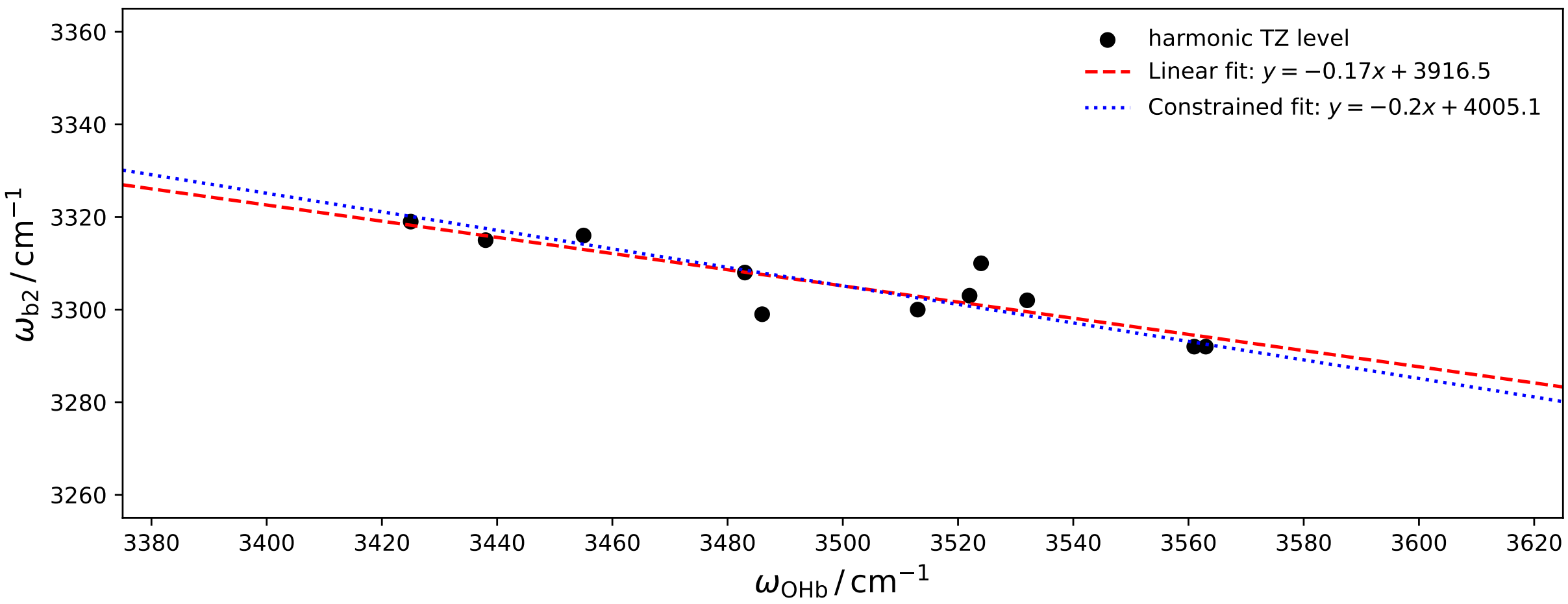


**Figure S35:** Dependence of the 1:1 harmonic b2 prediction on the harmonic OHb prediction for the TZ approach, with an unconstrained linear fit and a fit constrained to a slope of −0.2, as assumed in modeling approaches in HyDRA II.

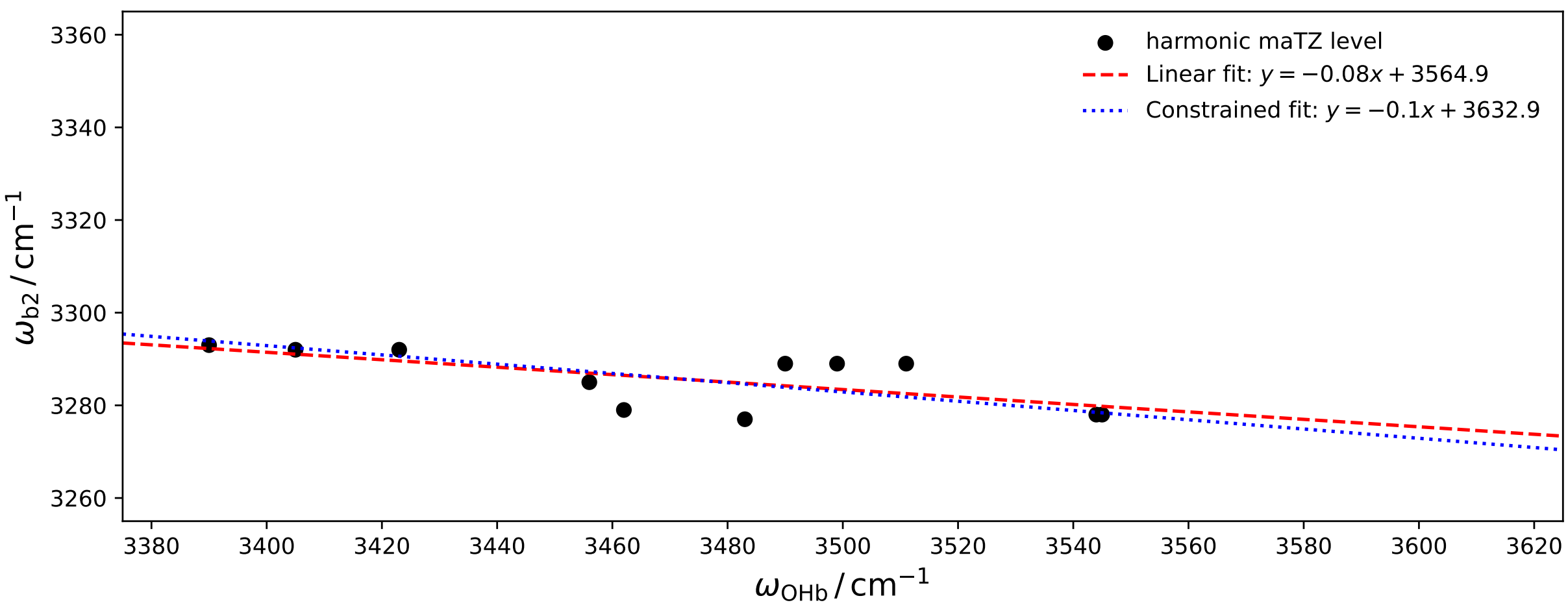


**Figure S36:** Dependence of the 1:1 harmonic b2 prediction on the harmonic OHb prediction for the maTZ computational levels, with an unconstrained linear fit and a fit constrained to a slope of −0.1.

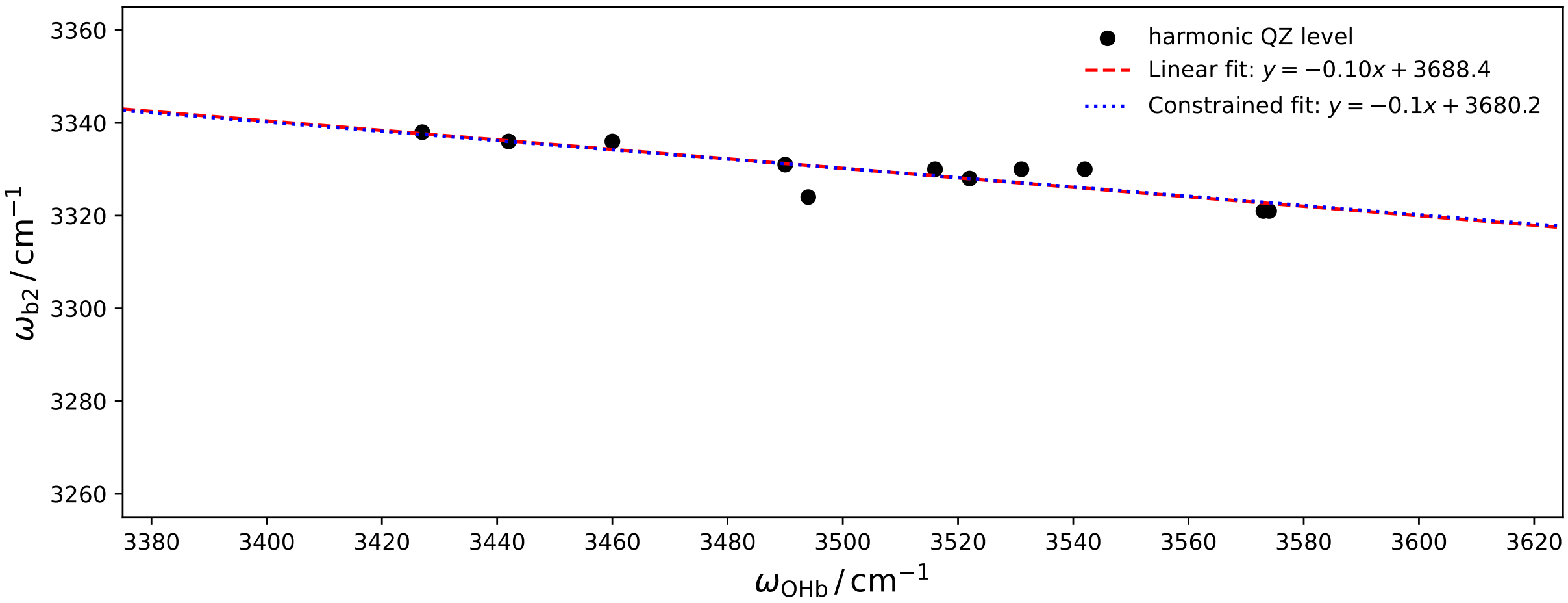


**Figure S37:** Dependence of the 1:1 harmonic b2 prediction on the harmonic OHb prediction for the QZ computational level, with an unconstrained linear fit and a fit constrained to a slope of −0.1.

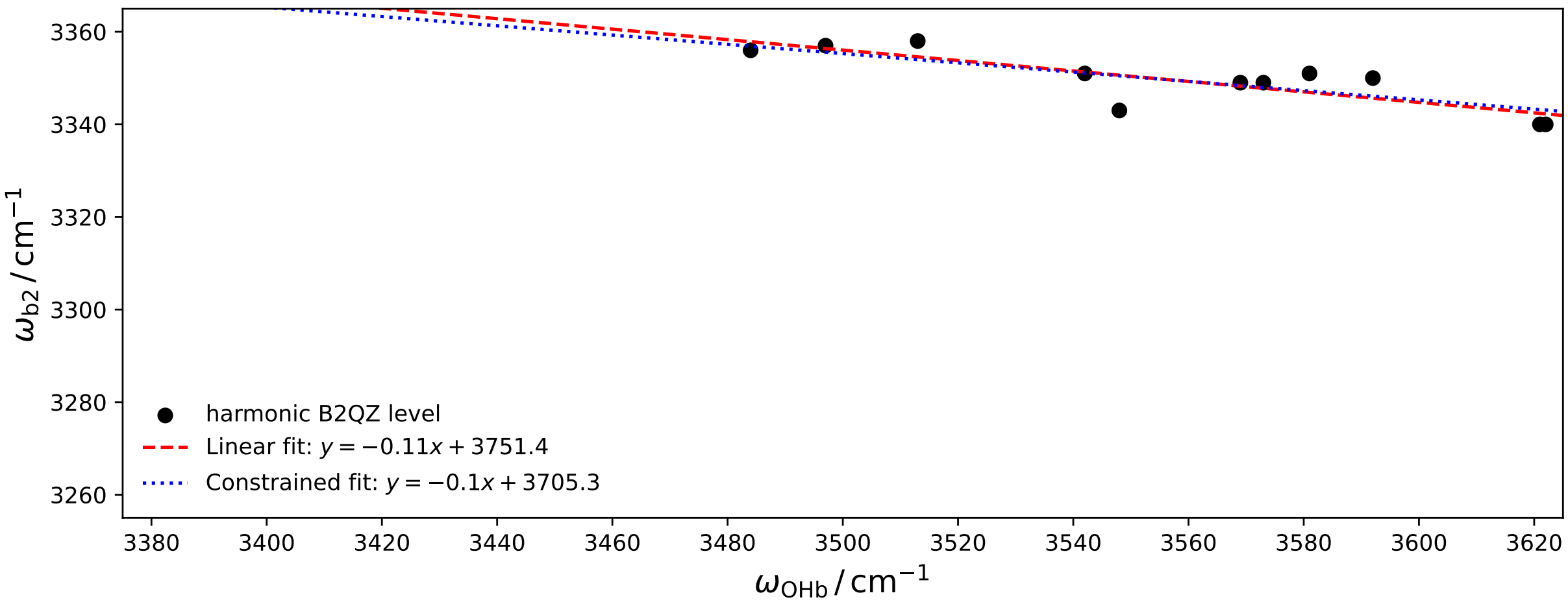


**Figure S38:** Dependence of the 1:1 harmonic b2 prediction on the harmonic OHb prediction for the B2QZ level, with an unconstrained linear fit and a fit constrained to a slope of −0.1.

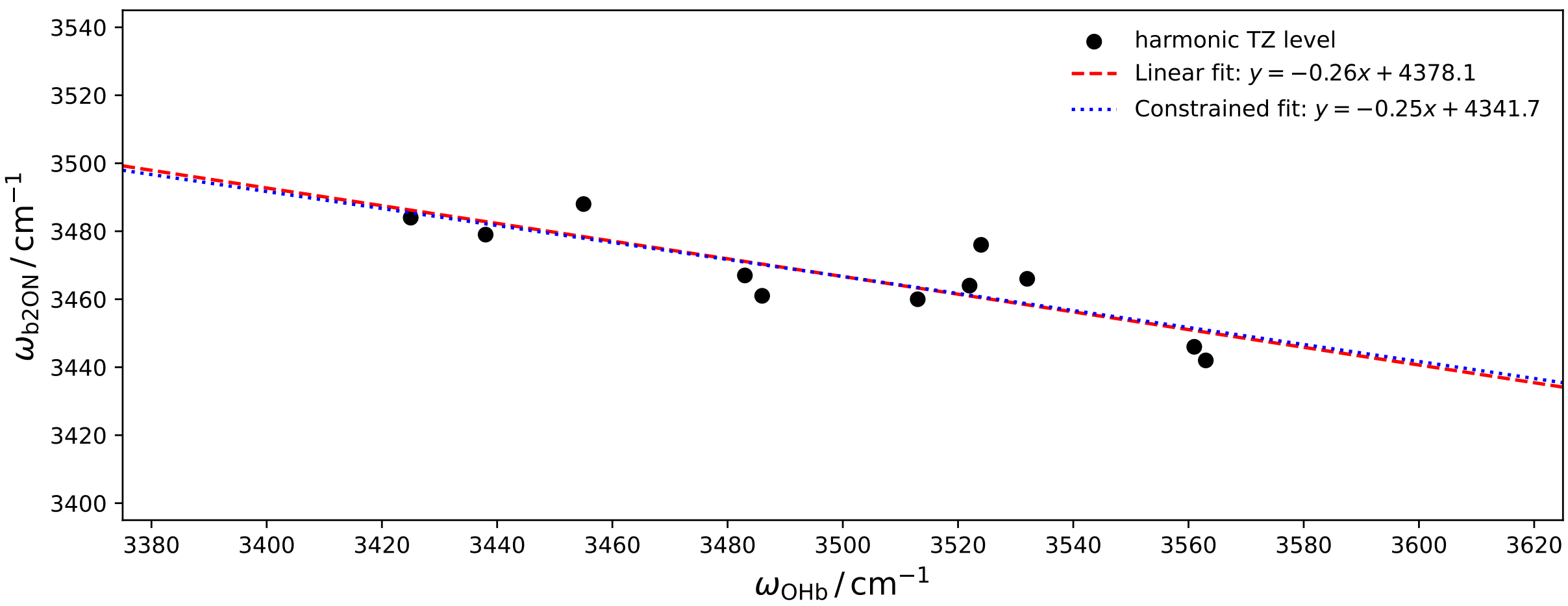


**Figure S39:** Dependence of the 1:1 harmonic b2ON prediction on the harmonic OHb prediction for the TZ level, with an unconstrained linear fit and a fit constrained to a slope of −0.25.

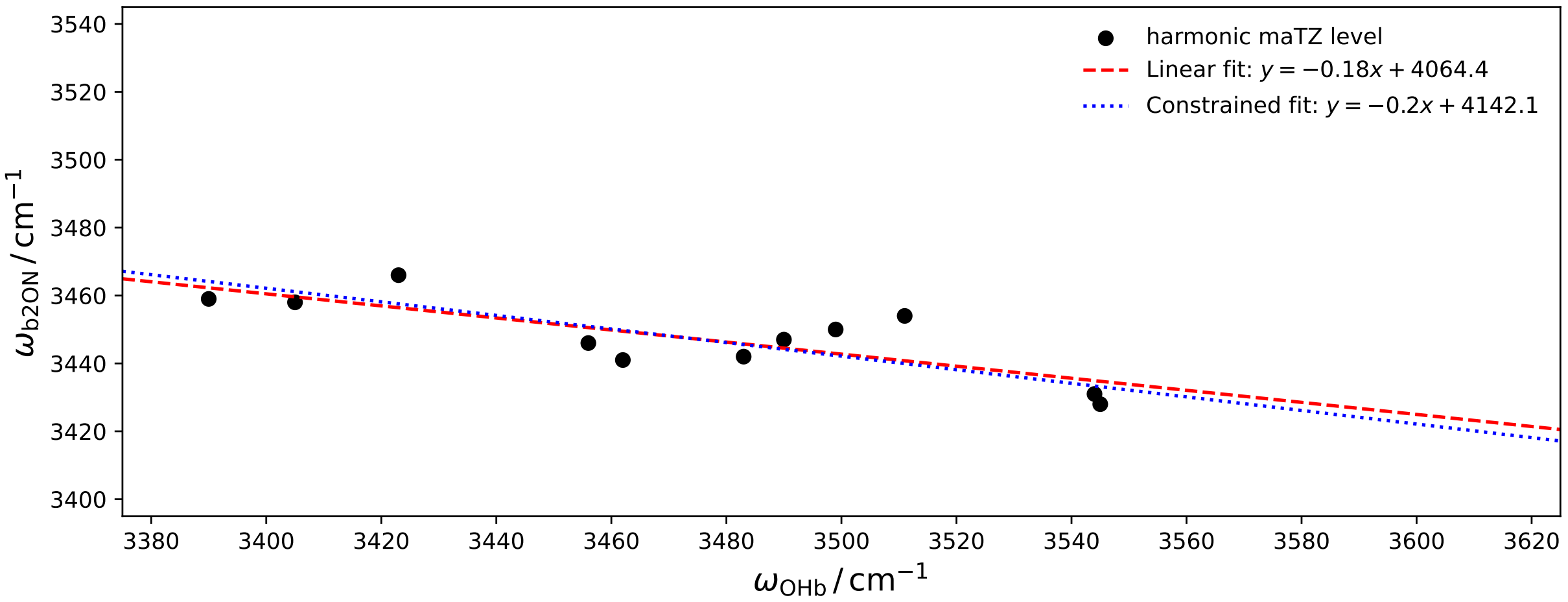


**Figure S40:** Dependence of the 1:1 harmonic b2ON prediction on the harmonic OHb prediction for the maTZ level, with an unconstrained linear fit and a fit constrained to a slope of −0.2.

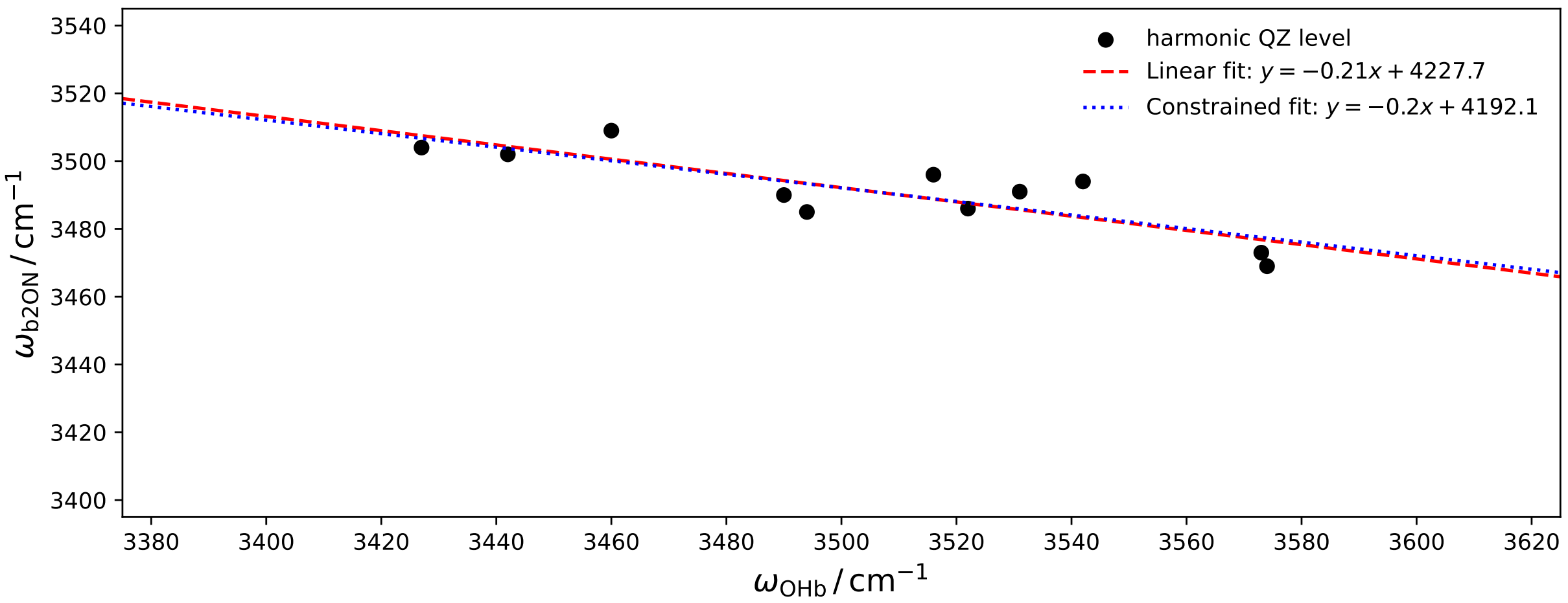


**Figure S41:** Dependence of the 1:1 harmonic b2ON prediction on the harmonic OHb prediction for the QZ level, with an unconstrained linear fit and a fit constrained to a slope of −0.2.

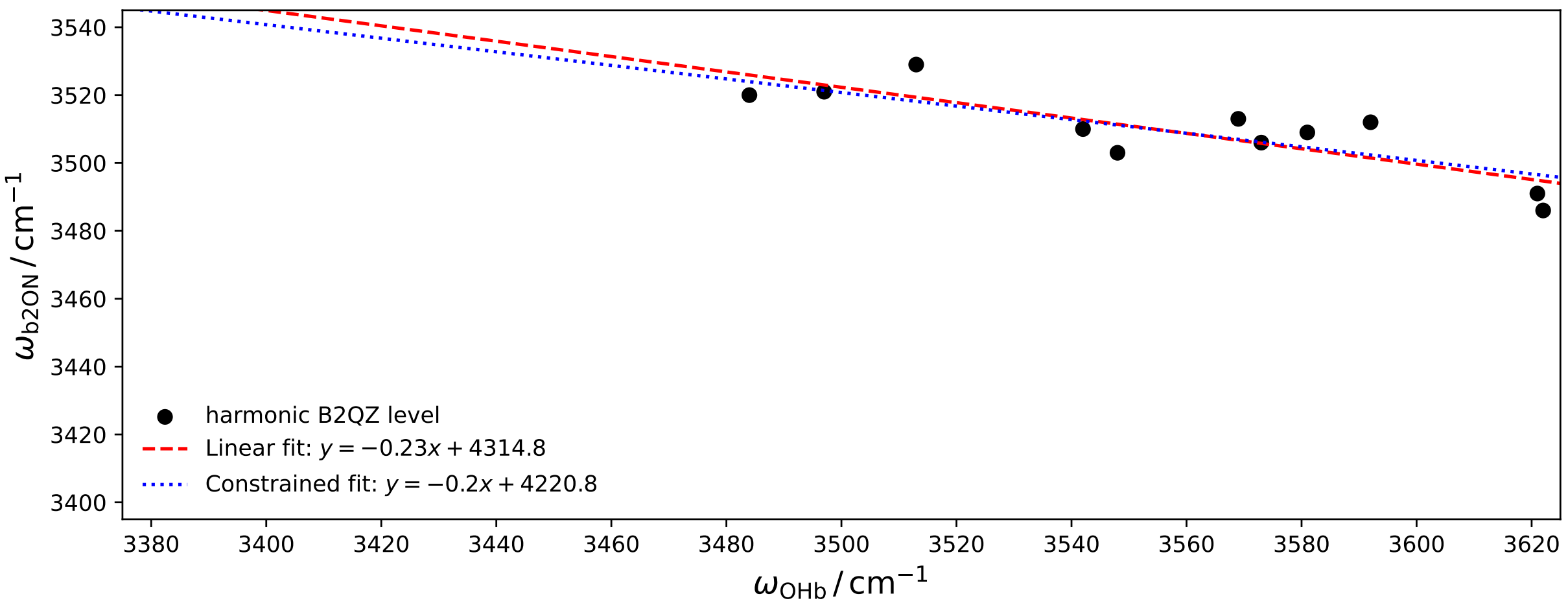


**Figure S42:** Dependence of the 1:1 harmonic b2ON prediction on the harmonic OHb prediction for the B2QZ level, with an unconstrained linear fit and a fit constrained to a slope of −0.2.

**Table S13:** Harmonically predicted wavenumbers for the combination band of the bending overtone and ON stretching vibration b2ON of the monohydrate (1:1) at different levels of computation. The values given in brackets reflect the predicted shift relative to 1-MPY. Data pairs in parentheses provide original harmonic wavenumbers where a demixing of the ON stretching or water bending fundamental b was necessary.

| system | TZ / $cm^{-1}$ | maTZ / $cm^{-1}$ | QZ / $cm^{-1}$ | B2QZ / $cm^{-1}$ |
|---|---|---|---|---|
| 4-Cl-1-MPY | 3446 [−20] | 3431 [−23] | 3473 [−21] | 3491 [−21] |
| 4-Br-1-MPY | 3442 [−24] | 3428 [−26] | 3469 [−25] | 3486 [−26] |
| | (3439 / 3445) | (3426 / 3430) | (3468 / 3471) | |
| 1-MPY | 3466 [0] | 3454 [0] | 3494 [0] | 3512 [0] |
| 1,4-DMPY | 3464 [−2] | 3450 [−4] | 3491 [−3] | 3509 [−3] |
| 1,3-DMPY | 3460 [−6] | 3447 [−7] | 3486 [−8] | 3506 [−6] |
| 1,3,5-TMPY | 3461 [−5] | 3441 [−13] | 3485 [−9] | 3503 [−9] |
| 1-MIM | 3476 [10] | 3442 [−12] | 3496 [2] | 3513 [1] |
| 5-Cl-1,2-DMIM | 3467 [1] | 3446 [−8] | 3490 [−3] | 3510 [−2] |
| 1,2-DMIM | 3488 [22] | 3466 [12] | 3509 [15] | 3529 [17] |
| 1,2,4-TMIM | 3479 [13] | 3458 [4] | 3502 [8] | 3521 [9] |
| | | (3457 / 3459) | | (3520 / 3522) |
| 1,2,4,5-TeMIM | 3484 [18] | 3459 [5] | 3504 [10] | 3520 [8] |

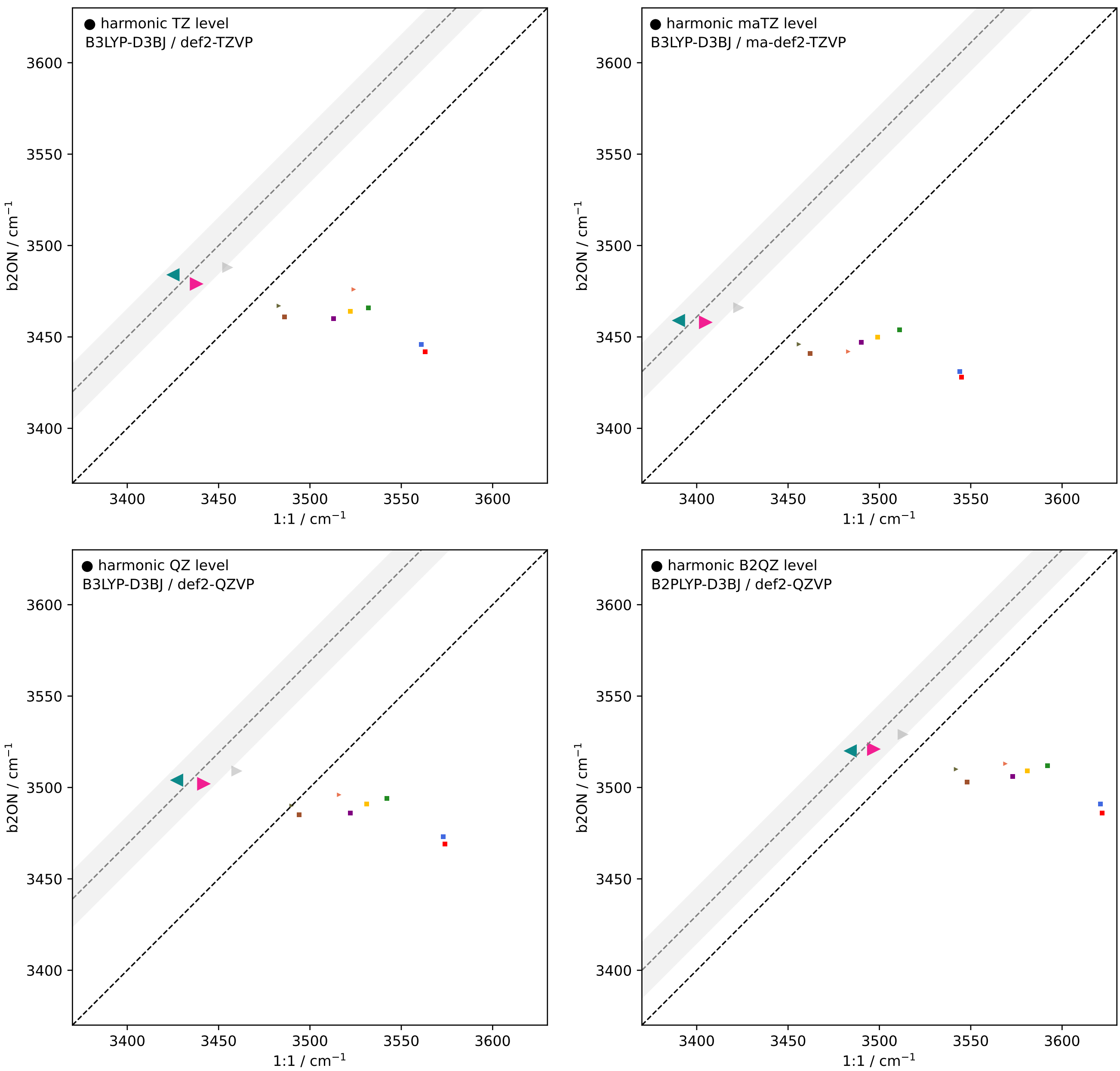


**Figure S43:** Chemical tuning of the proximity of the OHb fundamental transition and the b2ON combination transition for the four different harmonic levels of computation (panels) and the 11 monohydrates (colors corresponding to the spectral colors). Squares (□) are used for pyrazoles, triangles (▷, ◁) for imidazoles. The symbol size encodes the intensity of the observed resonance, with 5% of the maximum possible size added as a base size to show non-resonant cases. If OHb is experimentally found above b2ON (stronger signal at higher wavenumber), the triangle points to the right (▷), if it is below b2ON (stronger signal at lower wavenumber), the triangle points to the left (◁). Perfect cancellation of the harmonic approximation and the DFT approximation errors would lead to large symbols near the dashed diagonal. Their actual position is more or less upshifted (grey ribbons centered midway between the two strongly coupled cases, extending over $\pm 15\,\mathrm{cm}^{-1}$ to account for the approximate range of the resonance), which means that the anharmonic downshift is smaller for b2ON than for OHb, modulated by the DFT variations. The illustrated upshifts are 50, 61, 69 and 30 $\mathrm{cm}^{-1}$ for TZ, maTZ QZ and B2QZ respectively. All probed DFT functionals correctly predict that the three systems with an observed resonance (large symbols) have similar harmonic approximation errors.

### 3.4.3 1:2 complexes

For dihydrates, it is less likely that the harmonic approximation yields robust results, because they involve strong and medium strength hydrogen bonds, with potential coupling of the involved OH oscillators. As shown in Tables S14, and S15, the two hydrogen-bonded OH groups differ substantially in their wavenumber, thus reducing kinematic coupling.

Harmonic mixing due to kinematic coupling can be an issue for strongly bound water OHb modes coming close in wavenumber to CH stretching modes of the acceptor (intra-intra mixing) and for intermolecular ON stretching modes coming close to scaffold vibrations of the acceptor (inter-intra mixing). Less frequent is harmonic mixing between b and other intramolecular modes, which affects the purity of the harmonic overtone (b2). If the two mixed-character normal modes are separated by less than 10 $cm^{-1}$ and it is difficult to assign dominant water motion to one of them, we use their average wavenumber for both. For intra-intra mixing, a more accurate separation is achieved in some cases (1-MIM 1:2d (TZ, maTZ), 5-Cl-1,2-DMIM 1:2d (TZ), 1,2-DMIM 1:2d (QZ), 1,2,4,5-TeMIM 1:2d (maTZ)) by shifting away one mode at a time by suitable fictitious mass scaling and by thus obtaining a decoupled estimate for the other. To correct in first order for the arbitrariness of mass scaling, the sum of the decoupled estimates is scaled to the sum of the original mixed modes involved.

**Table S14:** Harmonically predicted wavenumbers for the directly bonded dihydrate (1:2d) OHb stretching vibration at different levels of computation. The values given in brackets reflect the predicted shift relative to 1-MPY. The dependence of the substitution effects on the basis set is still more pronounced for imidazoles ($\leq$ 13 $cm^{-1}$) than for pyrazoles ($\leq$ 7 $cm^{-1}$). Note that for some of the most downshifted OHb wavenumbers, harmonic mixing with CH stretching motion is predicted to set in. In such cases, data sets in parenthesis provide the original harmonic wavenumbers with partial (1:2d) OHb character.

| system | TZ / $cm^{-1}$ | maTZ / $cm^{-1}$ | QZ / $cm^{-1}$ | B2QZ / $cm^{-1}$ |
|---|---|---|---|---|
| 4-Cl-1-MPY | 3394 [45] | 3382 [48] | 3416 [45] | 3476 [42] |
| 4-Br-1-MPY | 3396 [47] | 3383 [49] | 3417 [46] | 3478 [44] |
| 1-MPY | 3349 [0] | 3334 [0] | 3371 [0] | 3434 [0] |
| 1,4-DMPY | 3335 [−14] | 3318 [−16] | 3357 [−14] | 3420 [−14] |
| 1,3-DMPY | 3306 [−43] | 3293 [−41] | 3332 [−39] | 3396 [−38] |
| 1,3,5-TMPY | 3283 [−66] | 3267 [−67] | 3308 [−63] | 3374 [−60] |
| 1-MIM | 3278 [−71] | 3253 [−81] | 3295 [−76] | 3363 [−71] |
| | (3271 / 3279) | (3246 / 3255) | | |
| 5-Cl-1,2-DMIM | 3269 [−80] | 3247 [−87] | 3292 [−79] | 3359 [−75] |
| | (3262 / 3271) | | | |
| 1,2-DMIM | 3222 [−127] | 3197 [−137] | 3245 [−126] | 3316 [−118] |
| | | | (3241 / 3248) | |
| 1,2,4-TMIM | 3194 [−155] | 3171 [−163] | 3219 [−152] | 3289 [−145] |
| 1,2,4,5-TeMIM | 3175 [−174] | 3149 [−185] | 3199 [−172] | 3272 [−162] |
| | | (3129 / 3151 / 3152) | | |

**Table S15:** Harmonically predicted wavenumbers for the indirectly bonded dihydrate (1:2i) OHb stretching vibration at different levels of computation. The values given in brackets reflect the predicted shift relative to 1-MPY. The dependence of the substitution effects on the basis set is comparable for imidazoles ($\leq$ 7 $cm^{-1}$) and for pyrazoles ($\leq$ 5 $cm^{-1}$).

| system | TZ / $cm^{-1}$ | maTZ / $cm^{-1}$ | QZ / $cm^{-1}$ | B2QZ / $cm^{-1}$ |
|---|---|---|---|---|
| 4-Cl-1-MPY | 3526 [11] | 3521 [11] | 3545 [11] | 3599 [10] |
| 4-Br-1-MPY | 3526 [11] | 3522 [12] | 3545 [11] | 3600 [11] |
| 1-MPY | 3515 [0] | 3510 [0] | 3534 [0] | 3589 [0] |
| 1,4-DMPY | 3512 [−3] | 3506 [−4] | 3530 [−4] | 3585 [−4] |
| 1,3-DMPY | 3512 [−3] | 3507 [−3] | 3533 [−1] | 3587 [−2] |
| 1,3,5-TMPY | 3508 [−7] | 3498 [−12] | 3524 [−10] | 3580 [−9] |
| 1-MIM | 3485 [−30] | 3479 [−31] | 3503 [−31] | 3558 [−31] |
| 5-Cl-1,2-DMIM | 3493 [−22] | 3487 [−23] | 3513 [−21] | 3570 [−19] |
| 1,2-DMIM | 3482 [−33] | 3475 [−35] | 3502 [−32] | 3559 [−30] |
| 1,2,4-TMIM | 3483 [−32] | 3474 [−36] | 3502 [−32] | 3560 [−29] |
| 1,2,4,5-TeMIM | 3480 [−35] | 3470 [−40] | 3499 [−35] | 3556 [−33] |

In contrast to the two OHb transitions in 1:2 dihydrates (1:2i, 1:2d), which are widely separated in harmonic wavenumber due to their different diagonal force constants, the two local water bending vibrations and correspondingly also their overtones (b2) are close in wavenumber and therefore split into delocalized in-phase (ip) and out-of-phase (oop) eigenstates. The bending overtone localized in the 1:2d water unit, which is expected to couple most strongly with the 1:2d component of OHb, will usually be slightly higher in wavenumber than the 1:2i component, but the kinematic coupling presumably dominates. Because of this and the two neighboring combination bands which complete the bending overtone tetrad, we roughly approximate the harmonic position of the b2 state which can couple to OHb (1:2d) as the average of the harmonic b2 predictions for the ip (Table S16) and oop (Table S17) states. This average is listed in Table S18.

**Table S16:** Harmonically predicted wavenumbers for the in-phase water bending overtone b2 of the dihydrate (1:2) at different levels of computation.

| system | TZ / $cm^{-1}$ | maTZ / $cm^{-1}$ | QZ / $cm^{-1}$ | B2QZ / $cm^{-1}$ |
|---|---|---|---|---|
| 4-Cl-1-MPY | 3314 | 3300 | 3333 | 3354 |
| 4-Br-1-MPY | 3314 | 3300 | 3333 | 3353 |
| 1-MPY | 3319 | 3304 | 3337 | 3358 |
| 1,4-DMPY | 3320 | 3306 | 3339 | 3359 |
| 1,3-DMPY | 3315 | 3303 | 3336 | 3357 |
| 1,3,5-TMPY | 3317 | 3302 | 3337 | 3357 |
| 1-MIM | 3337 | 3328 | 3353 | 3375 |
| 5-Cl-1,2-DMIM | 3329 | 3318 | 3349 | 3368 |
| 1,2-DMIM | 3334 | 3324 | 3355 | 3374 |
| 1,2,4-TMIM | 3330 | 3322 | 3354 | 3372 |
| 1,2,4,5-TeMIM | 3333 | 3328 | 3357 | 3377 |

**Table S17:** Harmonically predicted wavenumbers for the out-of-phase water bending overtone b2 of the dihydrate (1:2) at different levels of computation. Data pairs in parentheses provide original harmonic wavenumbers where a demixing of the water bend fundamental b was necessary.

| system | TZ / $cm^{-1}$ | maTZ / $cm^{-1}$ | QZ / $cm^{-1}$ | B2QZ / $cm^{-1}$ |
|---|---|---|---|---|
| 4-Cl-1-MPY | 3277 | 3255 | 3300 | 3320 |
| 4-Br-1-MPY | 3277 | 3255 | 3299 | 3319 |
| 1-MPY | 3284 | 3260 | 3304 | 3324 |
| 1,4-DMPY | 3286 | 3262 | 3307 | 3326 |
| 1,3-DMPY | 3277 | 3261 | 3306 | 3325 |
| 1,3,5-TMPY | 3282 | 3263 | 3309 | 3327 |
| 1-MIM | 3307 | 3285 | 3320 | 3339 |
| 5-Cl-1,2-DMIM | 3302 | 3276 | 3317 | 3335 |
| 1,2-DMIM | 3307 | 3280 | 3321 | 3339 |
| 1,2,4-TMIM | 3305 | 3280 | 3321 | 3340 |
| 1,2,4,5-TeMIM | 3307 | 3300 | 3322 | 3341 |
| | (3298 / 3308) | | | |

Harmonic IR intensities can be useful in assigning two different transitions to the same cluster species, because the intensity ratios tend to be more reliable than absolute intensities in the double-harmonic approximation. Figure S44 plots the computed 1:2i/1:2d intensity ratio as a function of experimental 1:2d wavenumber for the different computational methods and compares it to the estimated experimental ratio. There are some clear outliers in the computed intensity ratios (filled symbols) due to harmonic mode mixing of 1:2d with nearby CH stretching fundamentals. By taking the sum of the two mixed intensities one obtains a more reasonable trend despite a slight overcorrection due to the added intrinsic intensity of the mixing CH mode.

**Table S18:** Average over harmonic ip and oop wavenumbers for the water bending overtones b2 of the dihydrate (1:2) at different levels of computation.

| system | TZ / $cm^{-1}$ | maTZ / $cm^{-1}$ | QZ / $cm^{-1}$ | B2QZ / $cm^{-1}$ |
|---|---|---|---|---|
| 4-Cl-1-MPY | 3296 | 3278 | 3317 | 3337 |
| 4-Br-1-MPY | 3295 | 3278 | 3316 | 3336 |
| 1-MPY | 3302 | 3282 | 3321 | 3341 |
| 1,4-DMPY | 3303 | 3284 | 3323 | 3343 |
| 1,3-DMPY | 3296 | 3282 | 3321 | 3341 |
| 1,3,5-TMPY | 3299 | 3383 | 3323 | 3342 |
| 1-MIM | 3322 | 3306 | 3337 | 3357 |
| 5-Cl-1,2-DMIM | 3315 | 3297 | 3333 | 3351 |
| 1,2-DMIM | 3321 | 3302 | 3338 | 3357 |
| 1,2,4-TMIM | 3318 | 3301 | 3337 | 3356 |
| 1,2,4,5-TeMIM | 3320 | 3314 | 3340 | 3359 |

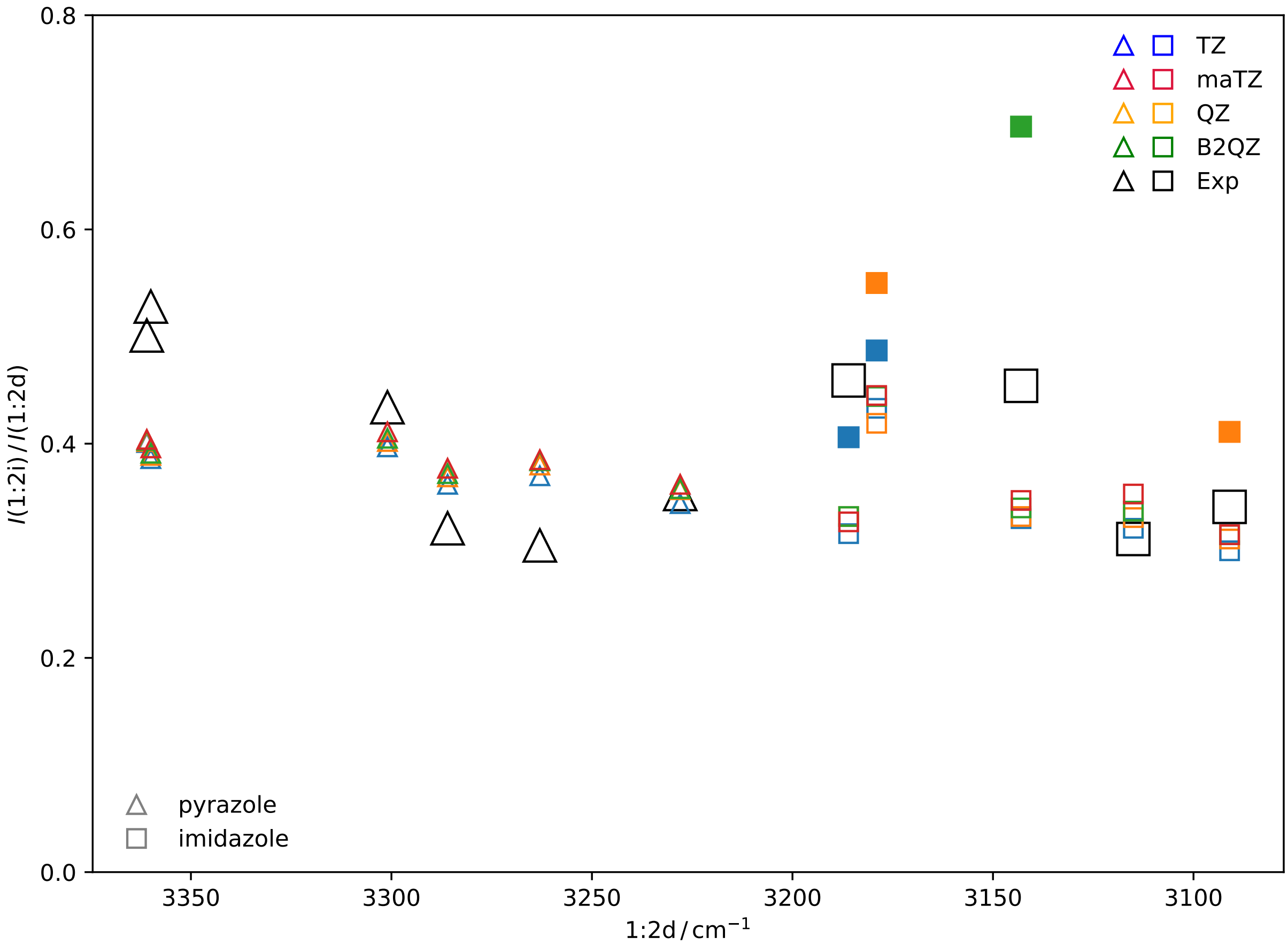


**Figure S44:** Computed harmonic intensity ratios 1:2i / 1:2d plotted versus the experimental 1:2d wavenumber for four computational levels. The computed ratio is approximately independent of the 1:2d wavenumber, showing a slight decrease with increasing hydrogen bond strength. Outliers (filled colored symbols) are due to harmonic mixing with CH stretching modes and are approximately corrected by summing the intensities of the mixed fundamentals. B2QZ is least susceptible to this mixing. The corresponding experimental intensity ratios (excluding 1-MIM due to overlap) are plotted with larger symbols (including any significant b2 intensity of the three most downshifted pyrazole dihydrates into 1:2d). Overall, the agreement between harmonic theory and anharmonic experiment is reasonable and supports a robust 1:3 intensity ratio between 1:2i and 1:2d. Slight deviations for the two least downshifted imidazoles may point at missing b2 intensity contributions in these cases.

# 4 Size decomposition and estimates of resonance contributions in methylated pyrazole mono- and dihydrate complexes

The spectra of three methylated pyrazole co-expansions with water exhibit weak b2 signals which are assumed to gain intensity from Fermi resonance with OHb of the 1:1 complex and OHb of the 1:2d transition in the 1:2 complex. For 1:1 complexes, a single weak b2 transition is expected in the absence of Fermi resonance. For the 1:2 complexes, up to four two-quantum bending transitions are expected in the absence of Fermi resonance, two b2 overtones and two combination bands. Not all four 1:2 bending states are expected to couple strongly to the closest 1:2d OHb state and due to the weak dependence of b2 on hydrogen bond strength, they will be split by kinematic coupling. Because there is typically only one observed b2 signal at low concentrations, the working hypothesis is that the 1:1 and dominant 1:2d contributions overlap, at least partially. Due to the ambiguity and weakness of b2, we estimate and assign a generic uncertainty of $\pm 5\,\mathrm{cm}^{-1}$ in its position, in contrast to the generic uncertainty of $\pm 1\,\mathrm{cm}^{-1}$ for OHb. Note that individual contributions to the b2 intensity centroid can deviate more. In situations where OHb and b2 mix (as evidenced by intensity sharing), the (integer) uncertainty $\pm u$ is shared in proportion to the b2 character (intensity), according to $u_{\mathrm{OHb}} = 3I_{\mathrm{b2}}/(I_{\mathrm{OHb}} + I_{\mathrm{b2}}) + 2$ and $u_{\mathrm{b2}} = 3I_{\mathrm{OHb}}/(I_{\mathrm{OHb}} + I_{\mathrm{b2}}) + 2$. For the results, see Table S8.

## 4.1 Treatment of the b2 resonance in pyrazole hydrates

To deconvolute these contributions, the first step is to subtract a suitably scaled water-poor expansion spectrum from a water-rich expansion spectrum, to remove the 1:1 OHb contribution. The corresponding difference spectra are necessarily more noisy than the individual spectra. They are shown in Fig. S45 and the analysis of the 1:2d OHb and b2 intensities is shown in Table S19.

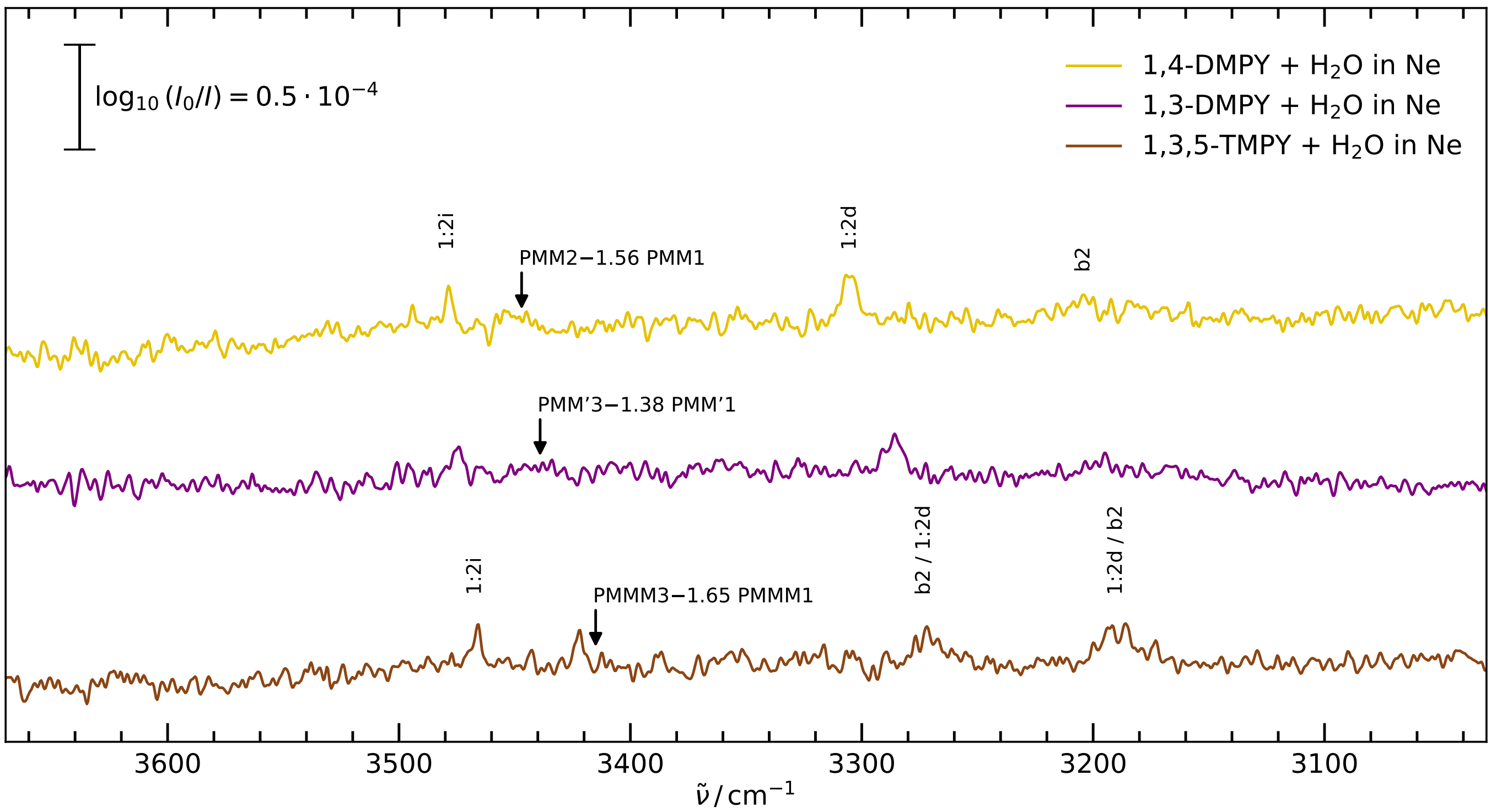


**Figure S45:** Difference spectra between water-rich (0.4 hPa) and water-poor (0.2 hPa) methylated pyrazole-water expansions in neon to remove the 1:1 complex contribution (marked with an arrow, see Table S3). The residual peak intensities in these difference spectra were used to estimate the dihydrate Fermi resonance coupling constant of OHb(2d) with b2 in the dihydrate, see Table S19.

This analysis together with the inspection of the original spectra suggests that the effective $W_2$ coupling constant (denoted $W_{\mathrm{b}}$ in the main text) between 1:2d OHb and the dominant b2 contribution is close to $40\,\mathrm{cm}^{-1}$. Any system dependence appears to be weaker than the statistical uncertainty, which grows with increasing detuning of the resonance. This is due to the decreasing significance of the perturber intensity. If the uncertainty includes zero intensity, the coupling constant vanishes. Therefore, coupling constants obtained by averaging over this uncertainty ($W_{2u}$) are somewhat smaller than those using the fixed average intensities ($W_{2f}$). The best defined coupling constant is that of 1,3,5-TMPY, where the resonance is perfect within experimental uncertainty, thus leading to two transitions of very similar intensity. From an experimental benchmarking perspective, the most important results in Table S19

**Table S19:** Experimental centroids of the perturbed (and deperturbed) 1:2d (1:2d$^0$) stretching vibrations and bending overtones b2 (b2$^0$) of different pyrazoles as well as their intensity fractions $I$ and the resulting coupling constants $W_2$. $W_2$ is calculated in two ways. $W_{2f}$ corresponds to the fixed wavenumbers and intensities and $W_{2u}$ averages over the uncertainties given in parentheses. The spectra used for the analysis are shown in Fig. S45.

| | Raw (perturbed) | | | | Deperturbed | | | |
|---|---|---|---|---|---|---|---|---|
| Compound | 1:2d / cm$^{-1}$ | $I_{1:2d}$ | b2 / cm$^{-1}$ | $I_{b2}$ | 1:2d$^0$ / cm$^{-1}$ | b2$^0$ / cm$^{-1}$ | $W_{2f}$ / cm$^{-1}$ | $W_{2u}$ / cm$^{-1}$ |
| 1,4-DMPY | 3305(3) | 0.81(9) | 3204(4) | 0.19(9) | 3286(9) | 3223(9) | 40(7) | 38(9) |
| 1,3-DMPY | 3286(3) | 0.74(10) | 3198(4) | 0.26(10) | 3263(9) | 3221(9) | 39(5) | 37(6) |
| 1,3,5-TMPY | 3191(3) | 0.53(10) | 3271(4) | 0.47(10) | 3228(9) | 3234(9) | 40(1) | 39(3) |

are the deperturbed OHb positions (1:2d$^0$) of the water-rich trimers. If a computational model does not account for stretch-bend Fermi resonance (such as a scaled harmonic calculation), it is advisable to compare its predictions to this deperturbed value. If the computational model includes Fermi resonance, it is more consistent to compare to the most intense raw signal (1:2d), which contains the largest OHb stretching character. Likely, the computational model then also will predict the fractional OHb character of the state, which corresponds to its fractional intensity $I_{1:2d}$ in the resonance doublet. The correlation between different harmonic calculations and experiment is illustrated in Fig. S46. A perfect anharmonic calculation without explicit Fermi resonance would coincide with the deperturbed experimental OHb value for 1:2d (diagonal line and ribbon $\tilde{\nu} = \omega$ in dark grey). High quality harmonic calculations must deviate systematically by predicting OHb higher than experimentally (lines and ribbons shifted to the right in light grey, approximating black lines connecting the individual pyrazole data pairs, note that even for the case where no b2 intensity can be attributed to the 1:2 complex, a coupling constant $W$=40 cm$^{-1}$ is implied in analogy to those cases where b2 intensity can be attributed to 1:2 complexes, see Table S20).

**Table S20:** Experimental centroids of the perturbed (and deperturbed) 1:2d (1:2d$^0$) stretching vibrations and bending overtones b2 (b2$^0$) of different pyrazoles as well as their intensity fractions $I$ under the assumption that the Fermi resonance coupling constant is fixed at $W_{fix}$=40 cm$^{-1}$. For the halogenated species, a 6% intensity b2 contribution would disappear in the spectral noise, whereas for 1-MPY, a 14% intensity contribution from the 1:2d resonance is at the threshold of being assignable.

| | Raw (perturbed) | | | | Deperturbed | | |
|---|---|---|---|---|---|---|---|
| Compound | 1:2d / cm$^{-1}$ | $I_{1:2d}$ | b2 / cm$^{-1}$ | $I_{b2}$ | 1:2d$^0$ / cm$^{-1}$ | b2$^0$ / cm$^{-1}$ | $W_{fix}$ / cm$^{-1}$ |
| 4-Cl-1-MPY | 3371 | 0.939 | 3204 | 0.061 | 3361 | 3214 | 40 |
| 4-Br-1-MPY | 3370 | 0.938 | 3204 | 0.062 | 3360 | 3214 | 40 |
| 1-MPY | 3317 | 0.859 | 3202 | 0.141 | 3301 | 3218 | 40 |

One can see that harmonic maTZ deviates the least from the dark grey diagonal, but this is a good agreement for the wrong reason, because realistic anharmonic correction would move the computation to the other side of the diagonal. In this sense, harmonic B2QZ shows the most realistic deviation from experiment, because an anharmonic correction by >100 cm$^{-1}$ would bring the black line and grey diagonal ribbon into good agreement. Rewardingly, all explored computational methods yield black lines which are systematically shifted from the diagonal, suggesting that they can be used similarly well for predictive purposes. Note that the actually observed transitions (orange points connected by dashed lines) correlate less well with the shifted diagonal, in particular for the most downshifted case of 1,3,5-TMPY, where OHb stretching intensity is shared almost equally with the b2 Fermi resonance partner, which leads to a bifurcation of the correlation.

To see which harmonic computational model best predicts the perfect Fermi resonance for 1,3,5-TMPY, Fig. S46 also shows the correlation of the harmonic b2 predictions with the estimated experimental b2 position (where visible, otherwise assuming the same $W_2$ coupling constant). Again, the raw b2 positions (yellow symbols with dashed lines) scatter more strongly than the deperturbed b2 positions (grey symbols). The gap between the grey and black 1,3,5-TMPY symbols (illustrated with a red double arrow) shows the size and sign of the mismatch. The only harmonic method which predicts the b2 state below OHb is B2QZ, the others predict it slightly above OHb, whereas experimentally the two coincide within experimental error. This means that harmonic predictions for unknown pyrazoles must be corrected in different directions for B2QZ and the other employed methods to predict the occurrence of a resonance. Actually, the corrections needed for the B3LYP variants are small enough (short red arrow) to be negligible in a simple model. In other words, the Fermi resonance condition for pyrazoles is coincidentally fulfilled for harmonic B3LYP predictions.

Based on this dihydrate analysis, one can try different estimates of the corresponding $W_2$ resonance for the monohydrate, where the spectral distance is considerably larger but the OHb signal is also stronger. One strategy is to

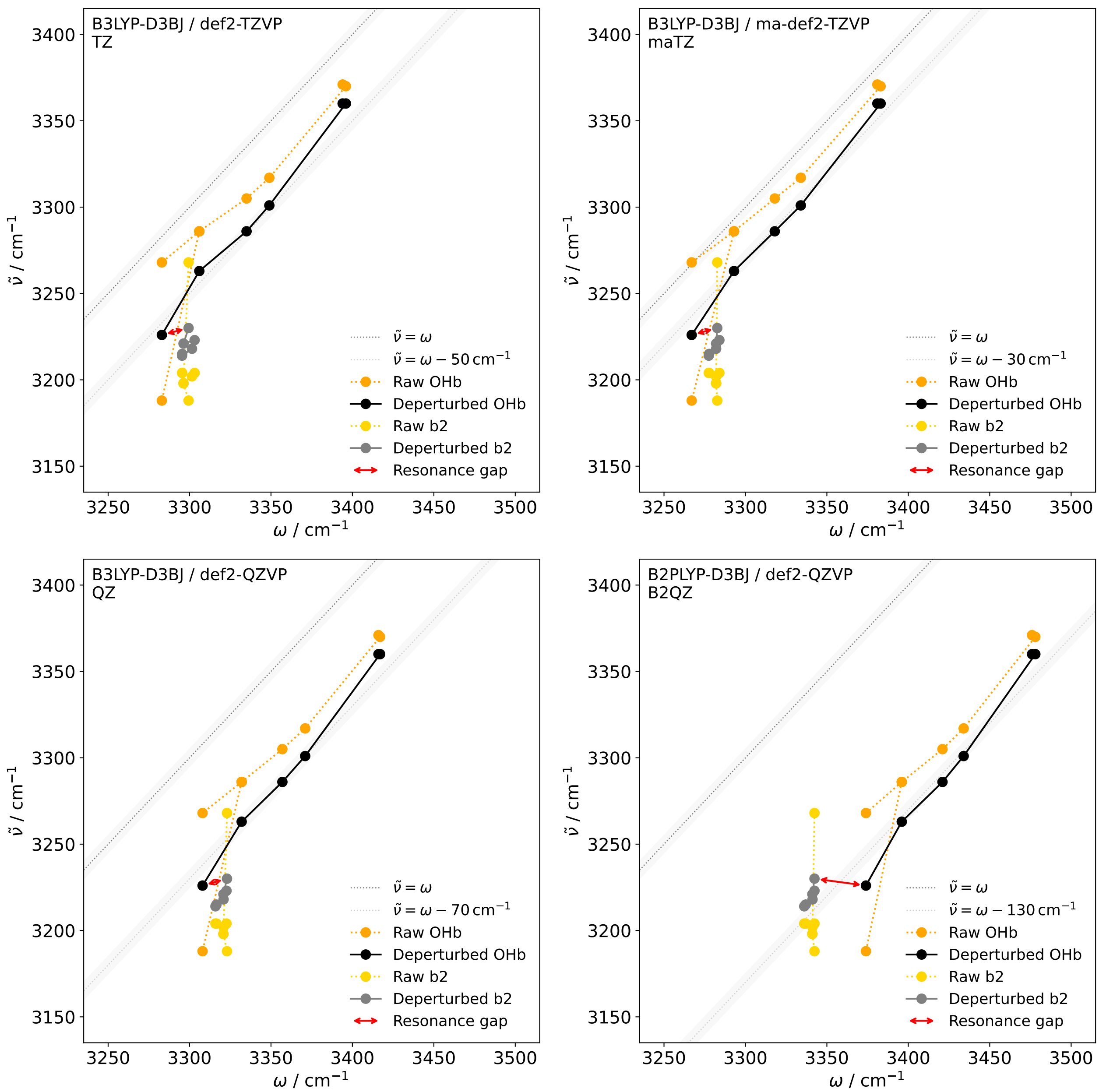


**Figure S46:** Comparison of the resonance condition between OHb and b2 in pyrazole dihydrates (1:2d) for different harmonic computational levels ($\omega$) and experiment ($\tilde{\nu}$). The red arrow indicates the harmonic prediction for the essentially perfect experimental resonance in 1,3,5-TMPY, by vertically (horizontally) connecting the deperturbed experimental positions (harmonic predictions) of OHb and b2 (black and grey data points). A short arrow is not a quality sign, because of neglected differences in anharmonicity for OHb and b2, but it suggests that the specific harmonic calculation predicts a level coincidence where it is also observed. The black and grey data points not connected with a red arrow stand for pyrazole dihydrates with less perfect experimental level coincidence. They are connected by solid lines in the sequence of progressive experimental wavenumber. For OHb (black), the connecting line has an approximate slope of 1, indicating that the hydrogen bond shift variation is predicted reasonably well by the computational approach. The offset relative to $\tilde{\nu} = \omega$ is a combination of DFT deficiency and neglected anharmonicity. For b2 (grey), the system dependence is much more subtle. Yellow and orange dots and dashed lines correspond to raw instead of deperturbed experimental positions. They behave more erratically and ambiguously, because OHb and b2 character is mixed by the perturbation. B2QZ is the most physical prediction for OHb, because it shows sizable anharmonicity effects in the expected direction. The other three levels require smaller corrections in the experiment-computation comparison due to reasonably systematic error cancellation.

use helium expansions, in which the overlapping dihydrate signals are very weak. Table S21 shows the result of this analysis, which avoids difference spectra. The average value is still close to $W_2 = 40\,\mathrm{cm}^{-1}$ and thus similar to the coupling constant for the dihydrate, but now the uncertainty is much larger, approaching 50 %.

**Table S21:** Experimental centroids of the perturbed (and deperturbed) 1:1 (1:1$^0$) complex stretching vibrations and bending overtones b2 (b2$^0$) for different pyrazoles as well as their intensity fractions $I$ and the resulting coupling constants $W_2$. $W_2$ is calculated in two ways. $W_{2f}$ corresponds to the fixed wavenumbers and intensities and $W_{2u}$ averages over the uncertainties given in parentheses. The underlying He expansion spectra used for the analysis are PMM3, PMM'2 and PMMM2 and are also marked in Table S3.

| | Raw (perturbed) | | | | Deperturbed | | | |
|---|---|---|---|---|---|---|---|---|
| Compound | 1:1 / cm$^{-1}$ | $I_{1:1}$ | b2 / cm$^{-1}$ | $I_{b2}$ | 1:1$^0$ / cm$^{-1}$ | b2$^0$ / cm$^{-1}$ | $W_{2f}$ / cm$^{-1}$ | $W_{2u}$ / cm$^{-1}$ |
| 1,4-DMPY | 3447(1) | 0.97(2) | 3204(5) | 0.03(2) | 3440(7) | 3211(7) | 42(14) | 38(16) |
| 1,3-DMPY | 3439(1) | 0.96(3) | 3203(5) | 0.04(3) | 3430(8) | 3212(8) | 45(11) | 42(19) |
| 1,3,5-TMPY | 3415(1) | 0.96(2) | 3205(5) | 0.04(2) | 3408(6) | 3213(6) | 39(10) | 39(11) |

An alternative strategy is to use the experimental dihydrate OHb/b2 ratio from the neon expansions and to subtract the b2 contribution for the dihydrate based on the dihydrate OHb signal in the helium expansions from the b2 integral. This analysis (Table S22) is less robust, underscoring that an uncertainty of at least 50% has to be assigned to the monohydrate $W_2$ value.

**Table S22:** Experimental centroids of the perturbed (and deperturbed) 1:1 (1:1$^0$) stretching vibrations and bending overtones b2 (b2$^0$) of different pyrazoles as well as their intensity fractions $I$ and the resulting coupling constants $W_2$. $W_2$ is calculated in two ways. $W_{2f}$ corresponds to the fixed wavenumbers and intensities and $W_{2u}$ averages over the uncertainties given in parentheses. The monohydrate b2 intensity was calculated by subtracting the intensity fraction of the dihydrate b2 with the following formula: $I_{b2,1:1} = I_{b2} - \left(\frac{I_{b2}}{I_{1:2}}\right)_{1:2} \cdot I_{1:2}$. The spectra used for the analysis are PMM1, PMM'1 and PMMM1 and are also marked in Table S3. Because the spectra differ from those used in Table S21, the b2 positions also vary.

| | Raw (perturbed) | | | | Deperturbed | | | |
|---|---|---|---|---|---|---|---|---|
| Compound | 1:1 / cm$^{-1}$ | $I_{1:1}$ | b2 / cm$^{-1}$ | $I_{b2}$ | 1:1$^0$ / cm$^{-1}$ | b2$^0$ / cm$^{-1}$ | $W_{2f}$ / cm$^{-1}$ | $W_{2u}$ / cm$^{-1}$ |
| 1,4-DMPY | 3447(1) | 0.99(3) | 3205(5) | 0.01(3) | 3444(7) | 3209(7) | 26(22) | 23(21) |
| 1,3-DMPY | 3439(1) | 0.94(4) | 3201(5) | 0.06(4) | 3424(10) | 3215(10) | 58(19) | 52(20) |
| 1,3,5-TMPY | 3415(1) | 0.96(5) | 3196(5) | 0.04(5) | 3406(10) | 3205(10) | 44(19) | 37(24) |

The insights from the monohydrates of di- and trimethylated pyrazoles, where a coupling constant between OHb and b2 of about 40 cm$^{-1}$ and in any case less than 60 cm$^{-1}$ is extracted (Table S21), can be transferred to the monohydrates of the other studied pyrazoles, by fixing the coupling constant at 40 cm$^{-1}$, or 60 cm$^{-1}$ as in water homoclusters [29]. This is done in Table S23.

**Table S23:** Experimental centroids of the perturbed (and deperturbed) 1:1 (1:1$^0$) stretching vibrations and bending overtones b2 (b2$^0$) of different pyrazoles as well as their intensity fractions $I$ under the assumption that the Fermi resonance coupling constant is fixed at $W_{fix} = 40$ cm$^{-1}$ and 60 cm$^{-1}$. Inspection of the associated jet spectra shows that a coupling constant larger than 60 cm$^{-1}$ can be safely excluded based on the experimental b2 intensity, whereas a coupling constant $W_{fix} = 40$ cm$^{-1}$ is consistent with the very weak absorption in the b2 region due to hydrate complexes.

| | Raw (perturbed) | | | | Deperturbed | | |
|---|---|---|---|---|---|---|---|
| Compound | 1:1 / cm$^{-1}$ | $I_{1:1}$ | b2 / cm$^{-1}$ | $I_{b2}$ | 1:1$^0$ / cm$^{-1}$ | b2$^0$ / cm$^{-1}$ | $W_{fix}$ / cm$^{-1}$ |
| 4-Cl-1-MPY | 3483 | 0.979 | 3204 | 0.021 | 3477 | 3210 | 40 |
| 4-Br-1-MPY | 3483 | 0.979 | 3204 | 0.021 | 3477 | 3210 | 40 |
| 1-MPY | 3457 | 0.975 | 3202 | 0.025 | 3451 | 3208 | 40 |
| 4-Cl-1-MPY | 3483 | 0.951 | 3204 | 0.049 | 3469 | 3218 | 60 |
| 4-Br-1-MPY | 3483 | 0.951 | 3204 | 0.049 | 3469 | 3218 | 60 |
| 1-MPY | 3457 | 0.941 | 3202 | 0.059 | 3442 | 3217 | 60 |

In summary, the methylated pyrazole spectra which reveal significant b2 contributions are consistent with a dihydrate Fermi resonance coupling constant $W_2$=40(4) cm$^{-1}$ with up to 50% wave-function mixing and a monohydrate Fermi resonance coupling constant $W_2$=40(20) cm$^{-1}$ with up to 10% wave-function mixing. For benchmarking purposes, the most important conclusion is that the observed OHb monohydrate wavenumber may be upshifted by up to 10 cm$^{-1}$ from the value in the absence of a b2 Fermi resonance. For the directly N-coordinating OHb dihydrate wavenumber,

this upshift may be as large as $40\,\mathrm{cm}^{-1}$ (for 1,3,5-TMPY, where the almost perfect resonance means that it could as well be a downshift of the same size, depending on which raw signal one assigns to OHb) and at least $20\,\mathrm{cm}^{-1}$.

Table S24 summarizes the resulting OHb stretching values which can be used for the benchmarking of anharmonic and harmonic computations of pyrazole mono- and dihydrates.

**Table S24:** OHb stretching wavenumbers in $\mathrm{cm}^{-1}$ for mono- (1:1) and dihydrates (1:2) of N-methylated pyrazoles which can be used for experimental benchmarking purposes. The raw values are obtained directly from the spectra and are useful when comparing to computational methods which fully account for anharmonicity. They are accompanied by estimated intensity fractions $P$ of the dominant raw signals among (potential) resonance polyads. The dep(erturbed) estimates aim to remove the effect of b2 Fermi resonance, either explicitly (last three entries) or implicitly ($^{i}$) by adopting an approximate coupling constant of $40\,\mathrm{cm}^{-1}$ derived from the explicit cases.

| system | 1:1-raw | $P_{max}$ / $P_{min}$ | (1:1-dep) | 1:2i-raw | 1:2d-raw | $P_{max}$ / $P_{min}$ | (1:2d-dep) |
|---|---|---|---|---|---|---|---|
| 4-Cl-1-MPY | 3483(1) | 1.00 / 0.90 | 3477(8)$^{i}$ | 3492(2) | 3371(2) | 1.00 / 0.90 | 3361(9)$^{i}$ |
| 4-Br-1-MPY | 3483(1) | 1.00 / 0.90 | 3477(8)$^{i}$ | 3492(2) | 3370(2) | 1.00 / 0.90 | 3360(9)$^{i}$ |
| 1-MPY | 3457(1) | 1.00 / 0.90 | 3451(8)$^{i}$ | 3482(2) | 3317(2) | 0.95 / 0.80 | 3301(9)$^{i}$ |
| 1,4-DMPY | 3447(1) | 1.00 / 0.90 | 3440(7) | 3479(2) | 3305(3) | 0.90 / 0.70 | 3286(9) |
| 1,3-DMPY | 3439(1) | 1.00 / 0.90 | 3430(8) | 3475(2) | 3286(3) | 0.85 / 0.60 | 3263(9) |
| 1,3,5-TMPY | 3415(1) | 1.00 / 0.90 | 3408(6) | 3467(2) | 3191(3) | 0.65 / 0.40 | 3228(9) |

# 5 Size decomposition and estimates of resonance contributions in methylated imidazole mono- and dihydrate complexes

Spectra of substituted imidazole-water coexpansions differ from those of substituted pyrazole-water coexpansions in various aspects. In the most redshifted monohydrates, there is a local resonance of OHb with b2ON leading to doublets and the Fermi resonance with b2 also becomes visible. For dihydrates, there are again 1:2i and 1:2d features, but the directly bound water OHb signal now falls below the b2 region. Somewhat surprisingly, this is accompanied by a loss of visible intensity transfer to b2. This is confirmed in difference spectra between expansions differing in water content (and sometimes also imidazole content), where the monohydrate contribution is removed by suitable scaling (Fig. S47). Despite the subtraction-amplified noise and some residual signals due to larger clusters, one can see that 1:2d is systematically more intense than 1:2i and that there is no b2 resonance contribution near or above $3200\,\mathrm{cm}^{-1}$ for dihydrates. One can also see that 1:2i is relatively independent of substitution, whereas 1:2d shows a significant redshift with increasing electron density at the N acceptor due to ring substitution.

There are several possible explanations for the elusiveness of b2 in the dihydrate complexes. As a first overtone and thus two-quantum transition, b2 may have sufficient intrinsic IR intensity beyond the resonance-acquired intensity that there are intensity interferences. Such interferences are expected to behave differently dependent on the energy sequence of the resonance partners. Alternatively, the swap of the energy sequence between OHb and b2 might give rise to new intra-complex energy redistribution channels, leading to a short lifetime and thus broad appearance of the b2 state. Given the achievable signal-to-noise ratio in the difference spectra, this could hide the b2 signature, if the resonance mixing ratio remains low. In any case, there remains considerable uncertainty in the deperturbation of the 1:2d dihydrate OHb transition. For the sake of completeness, we add a worst-case upper uncertainty of $60\,\mathrm{cm}^{-1}$ to the deperturbed 1:2d position. This would apply if by chance b2 and 1:2d coincide and at the same time they are anharmonically coupled as strongly as in cooperative water homoclusters.[29] In the following, any b2 intensity in monohydrate-dominated spectra is thus assigned to the monohydrate.

## 5.1 Treatment of the b2ON resonance in imidazole monohydrates

The possible resonances in imidazole monohydrates are analyzed sequentially. First, the local resonance of OHb with b2ON is analyzed, where it is clearly visible (Table S25). The associated effective coupling constant $W_3$ (denoted $W_N$ in the main text) is found to be close to $15\,\mathrm{cm}^{-1}$. This is extrapolated to those (less red-shifted) cases where the resonance is no more visible at the available signal-to-noise ratio, but may still have an effect on the deperturbed position ($1{:}1^0$). For this purpose, $W_{fix} = 15\,\mathrm{cm}^{-1}$ is used (Table S26) together with an estimated position of b2ON. The estimated (deperturbed) position is obtained from plotting the harmonic predictions of b2ON vs. the deperturbed positions for the cases with visible resonance. The deperturbed positions for 1-MIM and 5-Cl-1,2-DMIM are estimated by inserting their harmonic prediction into the linear correlations obtained from the visible resonances at different computational levels. By taking the average of the results for all computational levels, the estimated deperturbed positions of b2ON are $3350\,\mathrm{cm}^{-1}$ and $3347\,\mathrm{cm}^{-1}$ for 1-MIM and 5-Cl-1,2-DMIM, respectively.

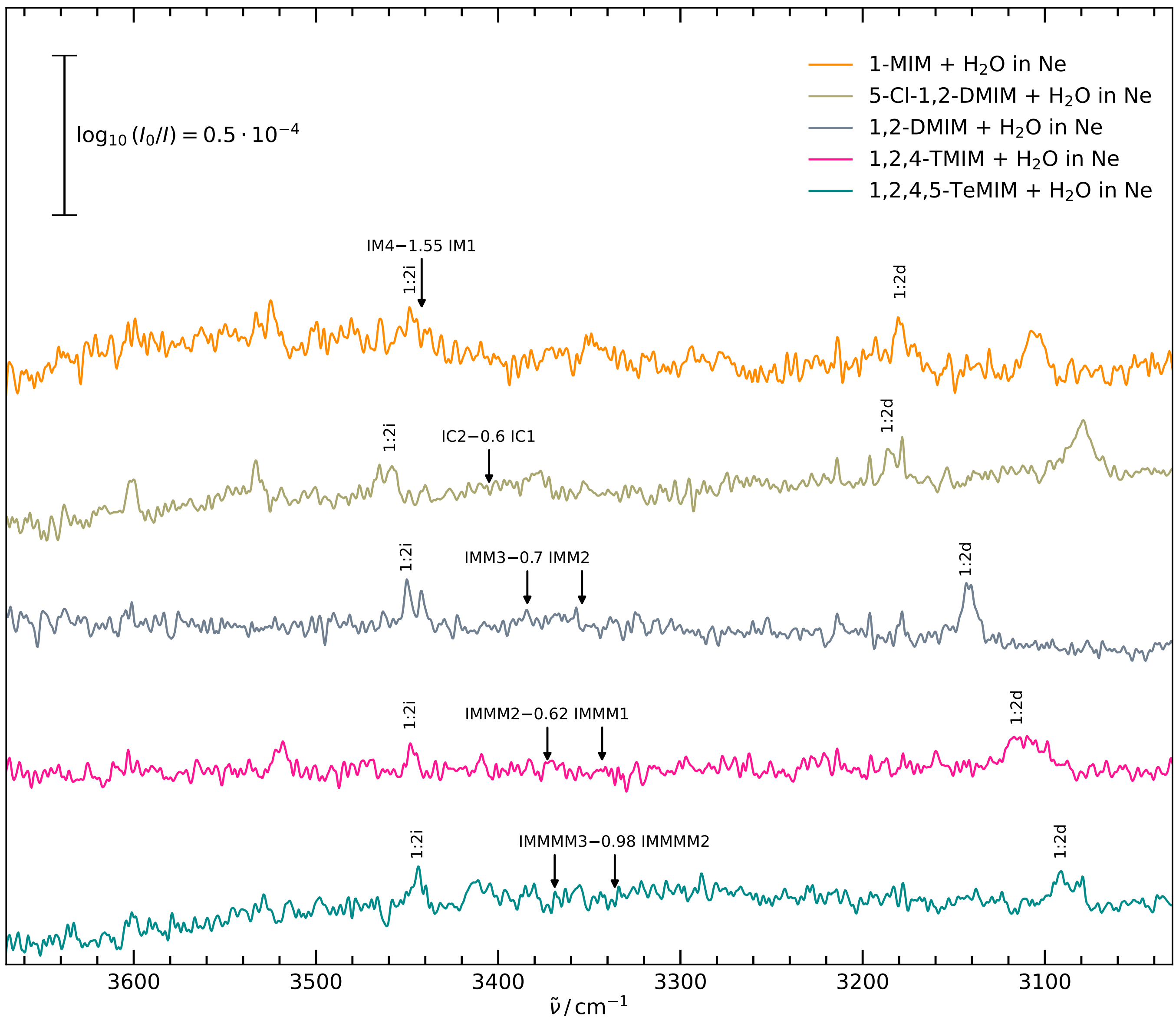


**Figure S47:** Difference spectra for N-methylated imidazole water co-expansions employing different expansion conditions. The components are scaled in such a way that monohydrate contributions (arrows) cancel and only the weak contributions from dihydrates (and larger clusters) remain. Due to slight temperature variations, the scaling factors vary more than usual for these compounds which are measured close to their saturation pressure.

**Table S25:** Experimental centroids of the perturbed (and deperturbed) 1:1 (1:1$^0$) stretching vibrations and combination band of the bending overtone with ON stretching fundamental b2ON (b2ON$^0$) of different imidazoles as well as their intensity fractions $I$ and the resulting coupling constants $W_3$. $W_3$ is calculated in two ways. $W_{3f}$ corresponds to the fixed wavenumbers and intensities and $W_{3u}$ averages over the uncertainties given in parentheses. The spectra used for the analysis are IMM4, IMMM3 and IMMMM2 and are also marked in Table S4.

| | Raw (perturbed) | | | | Deperturbed | | | |
|---|---|---|---|---|---|---|---|---|
| Compound | 1:1 / cm$^{-1}$ | $I_{1:1}$ | b2ON / cm$^{-1}$ | $I_{b2ON}$ | 1:1$^0$ / cm$^{-1}$ | b2ON$^0$ / cm$^{-1}$ | $W_{3f}$ / cm$^{-1}$ | $W_{3u}$ / cm$^{-1}$ |
| 1,2-DMIM | 3384(1) | 0.74(4) | 3354(3) | 0.26(4) | 3376(1) | 3362(1) | 13(1) | 13(2) |
| 1,2,4-TMIM | 3373(1) | 0.59(5) | 3343(3) | 0.41(5) | 3361(2) | 3355(2) | 15(1) | 15(2) |
| 1,2,4,5-TeMIM | 3337(3) | 0.62(9) | 3369(3) | 0.38(9) | 3349(3) | 3357(3) | 16(1) | 15(2) |

## 5.2 Treatment of the b2 resonance in imidazole monohydrates

Now, with rather reliable locally deperturbed 1:1$^0$ positions, one can try to globally deperturb this position by including the effect of b2 Fermi resonance (Table S27). It is rewarding to see that this analysis yields reasonable coupling constants around 40-50 cm$^{-1}$, certainly below the upper limit of 60 cm$^{-1}$, in those cases where the resonance can be

**Table S26:** Experimental centroids of the perturbed (and deperturbed) 1:1 (1:1$^0$) stretching vibrations and combination band of the bending overtone with ON stretching fundamental b2ON (b2ON$^0$) of different imidazoles as well as their intensity fractions $I$ under the assumption that the coupling constant between 1:1 and b2ON is fixed at $W_{fix}$ =15 cm$^{-1}$. The experimental non-observation of b2ON in pyrazole monohydrates is consistent with the non-observation for imidazole monohydrates with OHb above 3400 cm$^{-1}$, because no pyrazole monohydrate with OHb below 3400 cm$^{-1}$ was investigated, assuming that b2ON for pyrazole monohydrates falls in the same range of 3340-3370 cm$^{-1}$ as in imidazole monohydrates.

| | Raw (perturbed) | | | | Deperturbed | | |
|---|---|---|---|---|---|---|---|
| Compound | 1:1 / cm$^{-1}$ | $I_{1:1}$ | b2ON / cm$^{-1}$ | $I_{b2ON}$ | 1:1$^0$ / cm$^{-1}$ | b2ON$^0$ / cm$^{-1}$ | $W_{fix}$ / cm$^{-1}$ |
| 1-MIM | 3442 | 0.972 | 3350 | 0.028 | 3439 | 3353 | 15 |
| 5-Cl-1,2-DMIM | 3405 | 0.932 | 3347 | 0.068 | 3401 | 3351 | 15 |

analyzed (Table S27). We therefore proceed as in the case of the b2lib resonance and include the possible effect of b2 on the globally deperturbed 1:1$^{00}$ position as an enhanced upper uncertainty, based on either the analogy to pyrazoles (Table S28, top, $W_{fix}$ =40 cm$^{-1}$) or the worst case scenario of $W_{fix}$ =60 cm$^{-1}$ (Table S28, bottom)

**Table S27:** Experimental centroids of the locally deperturbed (and globally deperturbed) 1:1$^0$ (1:1$^{00}$) stretching vibrations and bending overtones b2 (b2$^0$) of different imidazoles, as well as their intensity fractions $I$ and the resulting coupling constants $W_2$. $W_2$ is calculated in two ways. $W_{2f}$ corresponds to the fixed wavenumbers and intensities and $W_{2u}$ averages over the uncertainties given in parentheses. The spectra used for the analysis are IMM4, IMMM3 and IMMMM2 and are also marked in Table S4.

| | Raw (locally deperturbed for OHb) | | | | Globally deperturbed | | | |
|---|---|---|---|---|---|---|---|---|
| Compound | 1:1$^0$ / cm$^{-1}$ | $I_{1:1}$ | b2 / cm$^{-1}$ | $I_{b2}$ | 1:1$^{00}$ / cm$^{-1}$ | b2$^0$ / cm$^{-1}$ | $W_{2f}$ / cm$^{-1}$ | $W_{2u}$ / cm$^{-1}$ |
| 1,2-DMIM | 3376(1) | 0.90(2) | 3212(5) | 0.10(2) | 3360(5) | 3228(5) | 49(5) | 49(5) |
| 1,2,4-TMIM | 3361(2) | 0.92(2) | 3206(5) | 0.08(2) | 3349(5) | 3218(5) | 42(6) | 42(5) |
| 1,2,4,5-TeMIM | 3349(3) | 0.88(5) | 3209(5) | 0.12(5) | 3332(7) | 3226(7) | 46(9) | 44(9) |

**Table S28:** Experimental centroids of the perturbed (and deperturbed) 1:1 (1:1$^0$) stretching vibrations and bending overtones b2 (b2$^0$) of different imidazoles, as well as their intensity fractions $I$ under the assumption that the Fermi resonance coupling constant is fixed at $W_{fix}$=40 cm$^{-1}$ and 60 cm$^{-1}$. Inspection of the associated jet spectra shows that a coupling constant larger than 60 cm$^{-1}$ can be safely excluded based on the experimental b2 intensity, whereas a coupling constant $W_{fix}$=40 cm$^{-1}$ is consistent with the very weak absorption in the b2 region due to hydrate complexes.

| | Raw (locally deperturbed for OHb/b2ON) | | | | Globally deperturbed | | |
|---|---|---|---|---|---|---|---|
| Compound | 1:1$^0$ / cm$^{-1}$ | $I_{1:1}$ | b2 / cm$^{-1}$ | $I_{b2}$ | 1:1$^{00}$ / cm$^{-1}$ | b2$^0$ / cm$^{-1}$ | $W_{fix}$ / cm$^{-1}$ |
| 1-MIM | 3439 | 0.969 | 3208 | 0.031 | 3432 | 3215 | 40 |
| 5-Cl-1,2-DMIM | 3401 | 0.958 | 3204 | 0.042 | 3393 | 3212 | 40 |
| 1-MIM | 3439 | 0.927 | 3208 | 0.073 | 3423 | 3225 | 60 |
| 5-Cl-1,2-DMIM | 3401 | 0.895 | 3204 | 0.105 | 3380 | 3225 | 60 |

Table S29 summarizes the OHb stretching values which can be used for the benchmarking of anharmonic and harmonic computations of imidazole mono- and dihydrates.

# 6 Integration methods

To calculate the deperturbed positions and coupling constants for the water complexes of N-methylated pyrazoles and imidazoles, band centers and integrated intensities were extracted from the experimental spectra (Figs. 2 and 3 in the main text). Note that absolute intensities are not relevant for this work. The relevant intensity fractions $I$ of components in a resonance polyad provided in the tables are dimensionless. The band intensities and centers (defined as the center of gravity of an individual band) were obtained by integrating the areas under the peaks using the NoisySignalIntegration method[30]. This method also provides uncertainty estimates for the intensities and band centers, based on the variation of the integration bounds and simulated noise. Detailed information on the integration method and the estimation of uncertainties is provided in references [30] and [31]. The raw (perturbed) intensities and band centres, evaluated using the NoisySignalIntegration program, are tabulated in Tables S19, S21, S22, S25, and S27.

**Table S29:** OHb stretching wavenumbers in $cm^{-1}$ for mono- (1:1) and dihydrates (1:2) of N-methylated imidazoles which can be used for experimental benchmarking purposes. The raw values are obtained directly from the spectra and are useful when comparing to computational methods which fully account for anharmonicity. The 1:1$^0$-dep(erturbed) values aim to remove the effect of b2ON resonance, either explicitly (last three entries) or implicitly ($^i$) by adopting an approximate coupling constant of 15 $cm^{-1}$ obtained from the explicit cases. The 1:1$^{00}$-dep values further remove the effect of b2 resonance, either explicitly (last three entries) or implicitly ($^i$) by adopting an approximate coupling constant of 40 $cm^{-1}$ obtained from the explicit cases.

| system | 1:1-raw | $^{P_{max}}_{P_{min}}$ | 1:1$^0$-dep | 1:1$^{00}$-dep | 1:2i-raw | 1:2d-raw |
|---|---|---|---|---|---|---|
| 1-MIM | 3442(2) | $^{1.00}_{0.85}$ | 3439(3)$^i$ | 3432(7)$^i$ | 3448(3) | 3179(5) |
| 5-Cl-1,2-DMIM | 3405(1) | $^{0.95}_{0.80}$ | 3401(3)$^i$ | 3393(7)$^i$ | 3459(3) | 3186(5) |
| 1,2-DMIM | 3384(1) | $^{0.75}_{0.60}$ | 3376(1) | 3360(5) | 3450(2) | 3143(5) |
| 1,2,4-TMIM | 3373(1) | $^{0.65}_{0.50}$ | 3361(2) | 3349(5) | 3448(2) | 3115(5) |
| 1,2,4,5-TeMIM | 3337(3) | $^{0.70}_{0.40}$ | 3349(3) | 3332(7) | 3444(2) | 3091(5) |

# 7 Summary of deperturbation strategies

If the results of this work are used as benchmark experiments for numerical quantum chemistry, the most straightforward comparison is that of the raw experimental mono- and dihydrate water OHb transitions with computational predictions. Only the experimental uncertainty due to limited spectral resolution, band broadening (and potentially assignment) is limiting the comparison.

However, in the specific case of pyrazoles and imidazoles, this implies that the computational predictions include anharmonic effects, not only diagonal anharmonicity (which can sometimes be approximated by scaling factors) but also the stretch/bend Fermi resonance with b2 as well as local perturbation with a three-quantum state (b2ON). For computational predictions which miss three-quantum local resonances, we offer a deperturbed wavenumber (dep) which plausibly assumes that the b2ON perturber carries no intrinsic intensity, such that its experimental intensity is exclusively derived from wavefunction mixing. In this limit (which assumes negligible vibrational Franck-Condon effects), the hypothetical position of OHb without interaction with b2ON (OHb$^0$) can be reconstructed from the position and relative intensity of the raw transitions. For systems which do not show b2ON signals, one can still estimate OHb$^0$ by transferring the (average) coupling constant from those related systems which show b2ON. A similar procedure can be applied to the stronger coupling with b2, but further uncertainties apply here. The intrinsic b2 intensity may be non-negligible, because it is a first overtone. This renders the coupling constants derived from a dark state 2-level analysis less reliable and one may be forced to assume an upper limit, estimated from water homoclusters. The b2 wavenumber is not very sensitive to cluster size, such that 1:1, 1:2 and 2:1 contributions may spectrally overlap. For these reasons, global deperturbations (gdp) including b2 have considerable uncertainty, in particular if b2 is not unambiguously observed. While raw and dep values differ in both wavenumber and uncertainty, gdp values based on uncertain b2 estimates keep the dep value, but increase the uncertainty asymmetrically.

Table S30 summarizes for the 11 pyrazoles and imidazoles how the deperturbation is applied to mono- and dihydrates for the predictions made in the present work and for the HyDRA database. The confidence level of the dominant raw OHb transitions ranges from ±1 to ±5 $cm^{-1}$, depending on the width and strength of the transitions, as outlined above and indicated after the wavenumber in parentheses in column 5. Deperturbation for visible resonances (column 7) in 9 out of the 33 OHb wavenumbers typically increases the wavenumber uncertainty to a high single-digit value due to intensity uncertainties. In another 8 cases, resonance is implied by analogy to related systems, leading to similar wavenumber uncertainties. In 5 of these 8 cases, not implying the resonance falls within the combined uncertainty, in the other 3 it exceeds it by less than a factor of 2. Conservative global deperturbation scenarios (column 8) always overlap within their uncertainties.

To give an example, computational approaches for the monohydrate of 1,2-DMIM which include full anharmonicity should target 3384(1) $cm^{-1}$ for the dominant OHb signal. If the b2ON resonance is not included in the calculation but lower order b2 Fermi resonance is, a value of 3376(1) $cm^{-1}$ should be targeted, and if no anharmonic resonance is included such as in scaled harmonic approaches, the target wavenumber is 3360(5) $cm^{-1}$. However, as the b2 intensity is relatively uncertain in this case, scaled harmonic predictions between 3355 and 3379 $cm^{-1}$ (column 8) might still be consistent with experiment within their intrinsic limitations. If a scaled harmonic prediction falls outside this range, deficiencies in the underlying electronic structure calculation are more likely.

Deperturbation for b2 is particularly challenging for pyrazole monohydrates due to the large spectral separation. In the three cases where it is possible, $W_2$ is consistently close to 40 $cm^{-1}$ but acquires a large uncertainty. As the associated uncertainty of the OHb position is still below 10 $cm^{-1}$, we opt for explicit deperturbation in these three cases. Computational methods which do not include the OHb/b2 Fermi resonance have to consider the associated

switch in the preferred dep and gdp values when introducing more than one methyl group to the pyrazole ring.

**Table S30:** Two-state coupling constants $W_2$ (OHb/b2, denoted $W_b$ in the main text) and $W_3$ (OHb/b2ON, denoted $W_N$ in the main text) in $cm^{-1}$ employed in deperturbation (dep) and global deperturbation (gdp) attempts at the service of simplified computational methods to predict 33 OHb transitions in mono-(1) and dihydrate transitions (2d, 2i) of substituted pyrazoles (PY) and imidazoles (IM) and the resulting raw, $P$, dep and gdp entries for the HyDRA database. To account for the OHb/b2 resonance where it remains unidentified in a certain complex, a worst case coupling constant of $60\,cm^{-1}$ is assumed when estimating the gdp uncertainty. Alternatively (second line for a given transition in the table in 8 cases), values extrapolated from homologs with visible resonances (with b2 and in some cases also with b2ON) are implied to derive alternative dep / gdp values (transferred $W$ values given without uncertainty). For water monomer, a coupling constant of $30\,cm^{-1}$ is implied in the gdp uncertainties. The last column marks the 33 entries which are planned to be included in the next HyDRA database update with an asterisk.

| heterocycle | OHb | $W_3$ | $W_2$ | raw | $P_{max}$ / $P_{min}$ | dep | gdp | HyDRA |
|---|---|---|---|---|---|---|---|---|
| 4-Cl-1-MPY | 1 | 0 | <60 | 3483(1) | 1.00 / 0.90 | 3483(1) | $3483(^{+03}_{-12})$ | * |
| | | 0 | 40 | 3483(1) | 1.00 / 0.90 | 3477(8) | $3477(^{+10}_{-07})$ | |
| | 2i | 0 | <60 | 3492(2) | 1.00 / 0.90 | 3492(2) | $3492(^{+04}_{-12})$ | * |
| | 2d | 0 | <60 | 3371(2) | 1.00 / 0.90 | 3371(2) | $3371(^{+04}_{-23})$ | * |
| | | 0 | 40 | 3371(2) | 1.00 / 0.90 | 3361(9) | $3361(^{+11}_{-08})$ | |
| 4-Br-1-MPY | 1 | 0 | <60 | 3483(1) | 1.00 / 0.90 | 3483(1) | $3483(^{+03}_{-12})$ | * |
| | | 0 | 40 | 3483(1) | 1.00 / 0.90 | 3477(8) | $3477(^{+10}_{-07})$ | |
| | 2i | 0 | <60 | 3492(2) | 1.00 / 0.90 | 3492(2) | $3492(^{+04}_{-12})$ | * |
| | 2d | 0 | <60 | 3370(2) | 1.00 / 0.90 | 3370(2) | $3370(^{+04}_{-23})$ | * |
| | | 0 | 40 | 3370(2) | 1.00 / 0.90 | 3360(9) | $3360(^{+11}_{-08})$ | |
| 1-MPY | 1 | 0 | <60 | 3457(1) | 1.00 / 0.90 | 3457(1) | $3457(^{+03}_{-13})$ | * |
| | | 0 | 40 | 3457(1) | 1.00 / 0.90 | 3451(8) | $3451(^{+10}_{-07})$ | |
| | 2i | 0 | <60 | 3482(2) | 1.00 / 0.90 | 3482(2) | $3482(^{+04}_{-13})$ | * |
| | 2d | 0 | <60 | 3317(2) | 1.00 / 0.90 | 3317(2) | $3317(^{+04}_{-38})$ | * |
| | | 0 | 40 | 3317(2) | 0.95 / 0.80 | 3301(9) | $3301(^{+11}_{-08})$ | |
| 1,4-DMPY | 1 | 0 | 38(16) | 3447(1) | 1.00 / 0.90 | 3440(7) | $3440(^{+09}_{-06})$ | * |
| | 2i | 0 | <60 | 3479(2) | 1.00 / 0.90 | 3479(2) | $3479(^{+04}_{-13})$ | * |
| | 2d | 0 | 38(9) | 3305(3) | 0.90 / 0.70 | 3286(9) | $3286(^{+11}_{-08})$ | * |
| 1,3-DMPY | 1 | 0 | 42(19) | 3439(1) | 1.00 / 0.90 | 3430(8) | $3430(^{+10}_{-07})$ | * |
| | 2i | 0 | <60 | 3475(2) | 1.00 / 0.90 | 3475(2) | $3475(^{+04}_{-13})$ | * |
| | 2d | 0 | 37(6) | 3286(3) | 0.85 / 0.60 | 3263(9) | $3263(^{+11}_{-08})$ | * |
| 1,3,5-TMPY | 1 | 0 | 39(11) | 3415(1) | 1.00 / 0.90 | 3408(6) | $3408(^{+08}_{-05})$ | * |
| | 2i | 0 | <60 | 3467(2) | 1.00 / 0.90 | 3467(2) | $3467(^{+04}_{-14})$ | * |
| | 2d | 0 | 39(3) | 3191(3) | 0.65 / 0.40 | 3228(9) | $3228(^{+11}_{-08})$ | * |
| 1-MIM | 1 | 0 | <60 | 3442(2) | 1.00 / 0.90 | 3442(2) | $3442(^{+04}_{-15})$ | * |
| | | 15 | 40 | 3442(2) | 1.00 / 0.85 | 3432(7) | $3432(^{+09}_{-06})$ | |
| | 2i | 0 | <60 | 3448(3) | 1.00 / 0.90 | 3448(3) | $3448(^{+05}_{-16})$ | * |
| | 2d | 0 | <60 | 3179(5) | 1.00 / 0.90 | 3179(5) | $3179(^{+56}_{-04})$ | * |
| 5-Cl-1,2-DMIM | 1 | 0 | <60 | 3405(1) | 1.00 / 0.90 | 3405(1) | $3405(^{+03}_{-17})$ | * |
| | | 15 | 40 | 3405(1) | 0.95 / 0.80 | 3393(7) | $3393(^{+09}_{-06})$ | |
| | 2i | 0 | <60 | 3459(3) | 1.00 / 0.90 | 3459(3) | $3459(^{+05}_{-15})$ | * |
| | 2d | 0 | <60 | 3186(5) | 1.00 / 0.90 | 3186(5) | $3186(^{+64}_{-04})$ | * |
| 1,2-DMIM | 1 | 13(2) | <60 | 3384(1) | 0.80 / 0.65 | 3376(1) | $3376(^{+03}_{-21})$ | |
| | | 13(2) | 49(5) | 3384(1) | 0.75 / 0.60 | 3360(5) | $3360(^{+07}_{-04})$ | * |
| | 2i | 0 | <60 | 3450(2) | 1.00 / 0.90 | 3450(2) | $3450(^{+04}_{-15})$ | * |
| | 2d | 0 | <60 | 3143(5) | 1.00 / 0.90 | 3143(5) | $3143(^{+36}_{-04})$ | * |
| 1,2,4-TMIM | 1 | 15(2) | <60 | 3373(1) | 0.65 / 0.50 | 3361(2) | $3361(^{+04}_{-25})$ | |
| | | 15(2) | 42(5) | 3373(1) | 0.65 / 0.50 | 3349(5) | $3349(^{+07}_{-04})$ | * |
| | 2i | 0 | <60 | 3448(2) | 1.00 / 0.90 | 3448(2) | $3448(^{+04}_{-15})$ | * |
| | 2d | 0 | <60 | 3115(5) | 1.00 / 0.90 | 3115(5) | $3115(^{+28}_{-04})$ | * |
| 1,2,4,5-TeMIM | 1 | 15(2) | <60 | 3337(3) | 0.75 / 0.50 | 3349(3) | $3349(^{+05}_{-28})$ | |
| | | 15(2) | 44(9) | 3337(3) | 0.70 / 0.40 | 3332(7) | $3332(^{+09}_{-06})$ | * |
| | 2i | 0 | <60 | 3444(2) | 1.00 / 0.90 | 3444(2) | $3444(^{+04}_{-15})$ | * |
| | 2d | 0 | <60 | 3091(5) | 1.00 / 0.90 | 3091(5) | $3091(^{+24}_{-04})$ | * |

# 8 A simple unified pyrazole/imidazole anharmonic model based on harmonic predictions

## 8.1 TZ model variant

One can now build a simple resonance model on top of the harmonic predictions at B3LYP-D3BJ/def2-TZVP level. It only uses uniform shifts and 2×2 Fermi resonance expressions $F[(\omega_1 + \omega_2)/2; \omega_1 - \omega_2; W)]$, where $\omega_1 - \omega_2$ is the wavenumber mismatch between two harmonic estimates for resonance partners and $(\omega_1 + \omega_2)/2$ their average position $\bar{\omega}_{W/\mathrm{cm}^{-1}}$, $W$ is the coupling matrix element and the dominant solution (OHb stretching character >50%) of the 2×2 problem is used. The parameters are the same for pyrazole and imidazole.

1:1 OHb fundamental:

$$\tilde{\nu}(\mathrm{OHb}) = F[\bar{\omega}_{15}; (F[\bar{\omega}_{40}; \omega(\mathrm{OHb}) - \omega(\mathrm{b2}); W_\mathrm{b} = 40\,\mathrm{cm}^{-1}] - 85\,\mathrm{cm}^{-1}) - (\omega(\mathrm{b2ON}) - 125\,\mathrm{cm}^{-1}); W_\mathrm{N} = 15\,\mathrm{cm}^{-1}]$$

The mismatch between the harmonic water OHb fundamental and the harmonic overtone of the water bending fundamental is used without any offset to determine a Fermi resonance splitting based on an effective coupling constant $W_\mathrm{b}$ of 40 cm$^{-1}$. The resulting coupled states are uniformly shifted by 85 cm$^{-1}$ to predict the actual anharmonic OHb fundamental (and b2 state). The OHb fundamental is further coupled by anharmonic resonance with the b2ON combination state, after uniformly downshifting its harmonic value by 125 cm$^{-1}$, to approximate the experimental crossing point. The effective coupling matrix element $W_\mathrm{N}$ is 15 cm$^{-1}$. Thus, three states (OHb, b2, b2ON) from the bright state of the 1:1 complex can be predicted.

1:2d OHb fundamental:

$$\tilde{\nu}(\mathrm{OHb}) = F[\bar{\omega}_{40}; \omega(\mathrm{OHb}) - \omega(\mathrm{b2}); W_\mathrm{b} = 40\,\mathrm{cm}^{-1}] - 50\,\mathrm{cm}^{-1}$$

The average between the in-phase and out-of-phase harmonic water bending fundamentals of dihydrates is coupled with the harmonic OHb fundamental of the directly bound water with a coupling constant $W_\mathrm{b} = 40\,\mathrm{cm}^{-1}$ and 50 cm$^{-1}$ are uniformly subtracted from the coupled states. Thus, two states (OHb, b2) are predicted, but b2 cannot be rigorously compared to experiment in the absence of spectral evidence for the two individual b2 states.

1:2i OHb fundamental:

$$\tilde{\nu}(\mathrm{OHb}) = \omega(\mathrm{OHb}) - 35\,\mathrm{cm}^{-1}$$

From the harmonic OHb fundamental of the indirectly solvating water, an estimate of the anharmonic counterpart is obtained by simply subtracting 35 cm$^{-1}$.

These simple models can also be used to predict relative intensities of the resonance polyad members in the dark state approximation [32].

Table S31 compares some predictions with experiment. One can see that the model performance is excellent for the 1:1 complex, in particular for pyrazole monohydrates, for the indirectly bound water in dihydrates and for the b2ON transition in imidazole monohydrates. It deteriorates for the imidazole monohydrates and the directly bound water in dihydrates, i.e. for the most strongly downshifted vibrations. This could be a limitation of the simple anharmonic model or of the electronic structure level leading to the harmonic reference values. However, there is a chance to apply individual corrections to the model in that spectral region, which is anyway plagued by unexpectedly missing b2 intensity enhancement due to Fermi resonance.

**Table S31:** Comparison of simple Fermi model predictions building on the TZ DFT approach with raw experimental wavenumbers (in cm$^{-1}$). Relative intensities of resonance polyad members rounded to one significant digit for a given species are given in brackets and compared to experiment, where available. Particularly uncertain b2 positions are marked with ?.

| system | 1:1 | 1:2d | 1:2i | b2 (1:1) | b2 (1:2d) | b2ON (1:1) |
|---|---|---|---|---|---|---|
| 4-Cl-1-MPY exp. | 3483(1) | 3371(2) | 3492(2) | 3204(5)? | 3204(5)? | - |
| TZ Fermi model | 3483 [1.0] | 3358 [0.9] | 3491 [1.0] | 3202 [0.02] | 3231 [0.1] | 3320 [0.01] |
| 4-Br-1-MPY | 3483(1) | 3370(2) | 3492(2) | 3204(5)? | 3204(5)? | |
| TZ Fermi model | 3485 [1.0] | 3360 [0.9] | 3491 [1.0] | 3201 [0.02] | 3231 [0.1] | 3316 [0.01] |
| 1-MPY | 3457(1) | 3317(2) | 3482(2) | 3202(5)? | 3202(5)? | |
| TZ Fermi model | 3456 [1.0] | 3322 [0.8] | 3480 [1.0] | 3210 [0.03] | 3229 [0.2] | 3339 [0.02] |
| 1,4-DMPY | 3447(1) | 3305(3) | 3479(2) | 3204(5) | 3204(4) | |
| TZ Fermi model | 3446 [1.0] | 3312 [0.7] | 3477 [1.0] | 3211 [0.03] | 3226 [0.3] | 3337 [0.02] |
| 1,3-DMPY | 3439(1) | 3286(3) | 3475(2) | 3203(5) | 3198(4) | |
| TZ Fermi model | 3437 [0.9] | 3291 [0.6] | 3477 [1.0] | 3208 [0.03] | 3211 [0.4] | 3333 [0.02] |
| 1,3,5-TMPY | 3415(1) | 3191(3) | 3467(2) | 3205(5) | 3271(4) | |
| TZ Fermi model | 3412 [0.9] | 3200 [0.6] | 3473 [1.0] | 3206 [0.04] | 3282 [0.4] | 3333 [0.04] |
| 1-MIM exp. | 3442(2) | 3179(5) | 3448(3) | 3208(5) | n.o. | - |
| TZ Fermi model | 3449 [0.9] | 3204 [0.7] | 3450 [1.0] | 3218 [0.03] | 3296 [0.3] | 3349 [0.02] |
| 5-Cl-1,2-DMIM | 3405(1) | 3186(5) | 3459(3) | 3204(5) | | |
| TZ Fermi model | 3410 [0.9] | 3197 [0.8] | 3458 [1.0] | 3214 [0.05] | 3288 [0.2] | 3339 [0.04] |
| 1,2-DMIM | 3384(1) | 3143(5) | 3450(2) | 3212(5) | | 3354(3) |
| TZ Fermi model | 3389 [0.7] | 3158 [0.9] | 3447 [1.0] | 3220 [0.07] | 3285 [0.1] | 3354 [0.2] |
| 1,2,4-TMIM | 3373(1) | 3115(5) | 3448(2) | 3206(5) | | 3343(3) |
| TZ Fermi model | 3375 [0.6] | 3132 [0.9] | 3448 [1.0] | 3218 [0.08] | 3279 [0.1] | 3343 [0.3] |
| 1,2,4,5-TeMIM exp. | 3337(3) [0.6] | 3091(5) [1.0] | 3444(2) [1.0] | 3209(5) [0.1] | n.o. | 3369(3) [0.3] |
| TZ Fermi model | 3341 [0.5] | 3115 [0.9] | 3445 [1.0] | 3220 [0.1] | 3281 [0.1] | 3371 [0.4] |
| Model offset (11) | 1.7 | 8.6 | 0.1 | 6.2 | | |
| RMSD (11) | 3.5 | 14.4 | 2.4 | 8 | | |
| Model offset (6 PY) | 0.7 | 0.6 | 0.3 | 2.6 | 21.2 | |
| RMSD (6 PY) | 1.7 | 8.7 | 2.9 | 5.1 | 22.3 | |
| Model offset (5 IM) | 4.6 | 18.2 | 0.2 | 10.4 | | 0.6 |
| RMSD (5 IM) | 4.8 | 19.1 | 1.7 | 10.5 | | 1 |

## 8.2 B2QZ model variant

A similar model applicable to the B2QZ harmonic calculations is:

1:1 OHb fundamental:

$$\tilde{\nu}(\mathsf{OHb}) = F[\bar{\omega}_{15}; (F[\bar{\omega}_{40}; \omega(\mathsf{OHb}) - \omega(\mathsf{b2}); W_\mathsf{b} = 40\,\mathsf{cm}^{-1}] - 145\,\mathsf{cm}^{-1}) - (\omega(\mathsf{b2ON}) - 165\,\mathsf{cm}^{-1}); W_\mathsf{N} = 15\,\mathsf{cm}^{-1}]$$

The empirical parameters are more physical than those of the TZ model, because $145\,\mathrm{cm}^{-1}$ is closer to twice the diagonal anharmonicity of an OH oscillator than $85\,\mathrm{cm}^{-1}$ and the $(165-145)\,\mathrm{cm}^{-1}$ difference is a more realistic value for twice the diagonal anharmonicity of the ON stretching vibration than $(125-85)\,\mathrm{cm}^{-1}$.

1:2d OHb fundamental:

$$\tilde{\nu}(\mathsf{OHb}) = F[\bar{\omega}_{40}; \omega(\mathsf{OHb}) - (\omega(\mathsf{b2}) + 25\,\mathsf{cm}^{-1}); W_\mathsf{b} = 40\,\mathsf{cm}^{-1}] - 130\,\mathsf{cm}^{-1}$$

The new need to augment $\omega(\mathsf{b2})$ by $25\,\mathrm{cm}^{-1}$ is not so easy to interpret, but is within the arbitrariness of averaging the bending fundamentals of the two water molecules in the dihydrate.

1:2i OHb fundamental:

$$\tilde{\nu}(\mathsf{OHb}) = \omega(\mathsf{OHb}) - 110\,\mathsf{cm}^{-1}$$

The larger offset of $110\,\mathrm{cm}^{-1}$ is more in line with the expected diagonal anharmonicity of this stretching mode.

Table S32 compares some predictions with experiment. One can see that the model performance is similar to that of the TZ model, in part even somewhat better. This points at larger remaining deficiencies in the anharmonic model than in the electronic structure, but more examples will be needed to corroborate this.

## 8.3 Improving the 1:2d performance (B2QZ')

Evidently, the performance of the model is least satisfactory for the 1:2d transition in imidazole dihydrates, coupled with the experimental finding that the b2 resonance partner is not observable in these mixed trimer spectra. This invites more speculative unified models and we suggest one such model based on the B2QZ calculations:

1:2d' OHb fundamental, with $\omega'(\mathsf{OHb}) = \omega(\mathsf{OHb}) - (\omega_\mathsf{M}(\mathsf{OH}) - \omega(\mathsf{OHb}))^2/\omega_\mathsf{M}(\mathsf{OH})$:

$$\tilde{\nu}(\mathsf{OHb})' = F[\bar{\omega}'_{40}; \omega'(\mathsf{OHb}) - (\omega(\mathsf{b2}) - 30\,\mathsf{cm}^{-1}); W_\mathsf{b} = 40\,\mathsf{cm}^{-1}] - 85\,\mathsf{cm}^{-1}$$

Essentially, this introduces hydrogen bond-induced diagonal anharmonicity into OHb which sets in when $\omega(\mathsf{OHb})$ falls below the monomer (symmetric stretch) value $\omega_\mathsf{M}(\mathsf{OH})$ (=$3839\,\mathrm{cm}^{-1}$ for B2QZ) and increases with increasing downshift rather than being independent of it, before Fermi coupling to b2. The coupled states are then uniformly downshifted by a smaller amount than for the model introduced in subsection 8.2. Note that this modification is completely ad hoc and it is up to future ab initio studies to show whether it can be justified by anharmonicity trends or by DFT deficiencies developing with increasing hydrogen bond strength, or not at all.

The performance for 1:2d is indeed improved, see Table S33. This model implies that the elusive b2 transition falls in the range of 3250-3275 $\mathrm{cm}^{-1}$ for most imidazoles, where weak and partially broad features are actually observed in some spectra recorded at higher concentrations.

**Table S32:** Comparison of simple Fermi model predictions building on the B2QZ DFT approach with raw experimental wavenumbers (in $cm^{-1}$). Relative intensities of resonance polyad members rounded to one significant digit for a given species are given in brackets and compared to experiment, where available. Particularly uncertain b2 positions are marked with ?.

| system | 1:1 | 1:2d | 1:2i | b2 (1:1) | b2 (1:2d) | b2ON (1:1) |
|---|---|---|---|---|---|---|
| 4-Cl-1-MPY exp. | 3483(1) | 3371(2) | 3492(2) | 3204(5)? | 3204(5)? | - |
| B2QZ Fermi model | 3483 [1.0] | 3359 [0.9] | 3489 [1.0] | 3189 [0.02] | 3219 [0.1] | 3324 [0.01] |
| 4-Br-1-MPY | 3483(1) | 3370(2) | 3492(2) | 3204(5)? | 3204(5)? | |
| B2QZ Fermi model | 3484 [1.0] | 3360 [0.9] | 3490 [1.0] | 3189 [0.02] | 3219 [0.1] | 3320 [0.01] |
| 1-MPY | 3457(1) | 3317(2) | 3482(2) | 3202(5)? | 3202(5)? | |
| B2QZ Fermi model | 3456 [1.0] | 3323 [0.8] | 3479 [1.0] | 3199 [0.03] | 3218 [0.2] | 3345 [0.02] |
| 1,4-DMPY | 3447(1) | 3305(3) | 3479(2) | 3204(5) | 3204(4) | |
| B2QZ Fermi model | 3445 [1.0] | 3312 [0.8] | 3475 [1.0] | 3199 [0.03] | 3216 [0.2] | 3342 [0.02] |
| 1,3-DMPY | 3439(1) | 3286(3) | 3475(2) | 3203(5) | 3198(4) | |
| B2QZ Fermi model | 3437 [1.0] | 3294 [0.7] | 3477 [1.0] | 3197 [0.03] | 3208 [0.3] | 3338 [0.02] |
| 1,3,5-TMPY | 3415(1) | 3191(3) | 3467(2) | 3205(5) | 3271(4) | |
| B2QZ Fermi model | 3414 [0.9] | 3201 [0.5] | 3470 [1.0] | 3191 [0.03] | 3281 [0.5] | 3335 [0.04] |
| 1-MIM exp. | 3442(2) | 3179(5) | 3448(3) | 3208(5) | n.o. | - |
| B2QZ Fermi model | 3434 [0.9] | 3201 [0.6] | 3448 [1.0] | 3197 [0.03] | 3284 [0.4] | 3345 [0.03] |
| 5-Cl-1,2-DMIM | 3405(1) | 3186(5) | 3459(3) | 3204(5) | | |
| B2QZ Fermi model | 3409 [0.9] | 3197 [0.6] | 3460 [1.0] | 3198 [0.04] | 3279 [0.4] | 3341 [0.05] |
| 1,2-DMIM | 3384(1) | 3143(5) | 3450(2) | 3212(5) | | 3354(3) |
| B2QZ Fermi model | 3387 [0.7] | 3167 [0.8] | 3449 [1.0] | 3203 [0.05] | 3271 [0.2] | 3355 [0.3] |
| 1,2,4-TMIM | 3373(1) | 3115(5) | 3448(2) | 3206(5) | | 3343(3) |
| B2QZ Fermi model | 3375 [0.6] | 3144 [0.9] | 3450 [1.0] | 3201 [0.05] | 3266 [0.1] | 3344 [0.4] |
| 1,2,4,5-TeMIM exp. | 3337(3) [0.5] | 3091(5) [1.0] | 3444(2) [1.0] | 3209(5) [0.1] | n.o. | 3369(3) [0.4] |
| B2QZ Fermi model | 3338 [0.5] | 3129 [0.9] | 3446 [1.0] | 3200 [0.1] | 3267 [0.1] | 3368 [0.4] |
| Model offset (11) | 0.4 | 12.1 | 0.3 | 8.9 | | |
| RMSD (11) | 3.2 | 19 | 2.4 | 9.7 | | |
| Model offset (6 PY) | 1 | 1.4 | 1.2 | 9.6 | 13 | |
| RMSD (6 PY) | 1.4 | 9 | 2.9 | 10.8 | 13.2 | |
| Model offset (5 IM) | 0.2 | 24.9 | 0.8 | 8.0 | | 0.1 |
| RMSD (5 IM) | 4.4 | 26.5 | 1.4 | 8.3 | | 0.8 |

**Table S33:** The refined 1:2d’ model with explicit dependence of diagonal anharmonicity on the downshift building on the same B2QZ DFT approach as in Table S32 shows a better fit to the experimental data. Particularly uncertain b2 positions are marked with ?.

| system | 1:2d’ | b2 (1:2d’) |
|---|---|---|
| 4-Cl-1-MPY exp. | 3371(2) | 3204(5)? |
| B2QZ Fermi model | 3368 [0.9] | 3211 [0.1] |
| 4-Br-1-MPY | 3370(2) | 3204(5)? |
| B2QZ Fermi model | 3370 [0.9] | 3210 [0.1] |
| 1-MPY | 3317(2) | 3202(5)? |
| B2QZ Fermi model | 3323 [0.9] | 3210 [0.1] |
| 1,4-DMPY | 3305(3) | 3204(4) |
| B2QZ Fermi model | 3310 [0.8] | 3208 [0.2] |
| 1,3-DMPY | 3286(3) | 3198(4) |
| B2QZ Fermi model | 3286 [0.7] | 3200 [0.3] |
| 1,3,5-TMPY | 3191(3) | 3271(4) |
| B2QZ Fermi model | 3190 [0.5] | 3270 [0.5] |
| 1-MIM exp. | 3179(5) | n.o. |
| B2QZ Fermi model | 3189 [0.6] | 3272 [0.4] |
| 5-Cl-1,2-DMIM | 3186(5) | |
| B2QZ Fermi model | 3184 [0.6] | 3267 [0.4] |
| 1,2-DMIM | 3143(5) | |
| B2QZ Fermi model | 3143 [0.9] | 3258 [0.1] |
| 1,2,4-TMIM | 3115(5) | |
| B2QZ Fermi model | 3113 [0.9] | 3253 [0.1] |
| 1,2,4,5-TeMIM exp. | 3091(5) [1.0] | n.o. |
| B2QZ Fermi model | 3093 [0.9] | 3255 [0.1] |
| Model offset (11) | 1.3 | |
| RMSD (11) | 4.1 | |
| Model offset (6 PY) | 1.1 | 4.3 |
| RMSD (6 PY) | 3.4 | 5.3 |
| Model offset (5 IM) | 1.5 | |
| RMSD (5 IM) | 4.7 | |

## 8.4 Testing the models with 6 new N-heterocycles

The computed structures for mono- and dihydrates of 6 (mostly commercially unavailable) N-alkylated pyrazoles and imidazoles (Table S34) are shown in Figs. S48 and S49.

**Table S34:** Table of N-heterocycles for which the OH stretching spectra are predicted in this work, prior to any spectral measurement, together with the used acronyms and their CAS number.

| Name | Code | CAS Number |
|---|---|---|
| 1,3,4,5-tetramethylpyrazole | 1,3,4,5-TeMPY | 1073-20-7 |
| 1-*tert*-butylpyrazole | 1-tBuPY | 15754-60-6 |
| 1-*tert*-butylimidazole | 1-tBuIM | 45676-04-8 |
| 1-*tert*-butyl-4-methylimidazole | 1-tBu-4-MIM | 67768-62-1 |
| 2-methyl(-1-trifluoromethyl)-1H-imidazole | 1-TFM-2-MIM | 2222049-66-1 |
| 5-fluoro-1,2,4-trimethylimidazole | 5-F-1,2,4-TMIM | |

Based on the harmonic monohydrate OHb (Table S35), b2 (Table S36) and b2ON (Table S37) as well as dihydrate OHb (Tables S38, S39), b2 (Tables S40, S41, S42) predictions of the 6 N-alkylated pyrazoles and imidazoles at various computational levels, anharmonic model predictions can be made.

**Table S35:** Harmonically predicted wavenumbers for the monohydrate (1:1) OHb stretching vibration at different levels of computation. Particularly interesting is the predicted closing of the pyrazole/imidazole spectral gap by switching from methyl- to *t-butyl-* substitution. Fluorination at the methyl group or directly ring raises the harmonic water wavenumber, whereas adding extra methyl groups is predicted to lead to a downshift. The average predicted wavenumber is similar for the four imidazoles and the two pyrazoles, helping to close the spectral gap between these two compound classes.

| system | TZ / $cm^{-1}$ | maTZ / $cm^{-1}$ | QZ / $cm^{-1}$ | B2QZ / $cm^{-1}$ |
|---|---|---|---|---|
| 1,3,4,5-TeMPY | 3481 | 3455 | 3487 | 3540 |
| 1-tBuPY | 3497 | 3473 | 3507 | 3556 |
| 1-tBuIM | 3506 | 3464 | 3498 | 3553 |
| 1-tBu-4-MIM | 3481 | 3443 | 3477 | 3532 |
| 1-TFM-2-MIM | 3527 | 3504 | 3534 | 3585 |
| 5-F-1,2,4-TMIM | 3465 | 3436 | 3470 | 3523 |

**Table S36:** Harmonically predicted wavenumbers for the monohydrate bend overtone vibration b2 at different levels of computation. The variation among the imidazoles is much smaller than for OHb.

| system | TZ / $cm^{-1}$ | maTZ / $cm^{-1}$ | QZ / $cm^{-1}$ | B2QZ / $cm^{-1}$ |
|---|---|---|---|---|
| 1,3,4,5-TeMPY | 3301 | 3277 | 3324 | 3343 |
| 1-tBuPY | 3296 | 3306 | 3339 | 3360 |
| 1-tBuIM | 3314 | 3284 | 3334 | 3352 |
| 1-tBu-4-MIM | 3319 | 3282 | 3335 | 3353 |
| 1-TFM-2-MIM | 3297 | 3275 | 3323 | 3345 |
| 5-F-1,2,4-TMIM | 3309 | 3285 | 3329 | 3349 |

For the highest harmonic level (B2QZ), the anharmonic model predictions are summarized in Table S43). For the simpler TZ level, the anharmonic model predictions are given in Table S44 and graphically presented in Fig. S50.

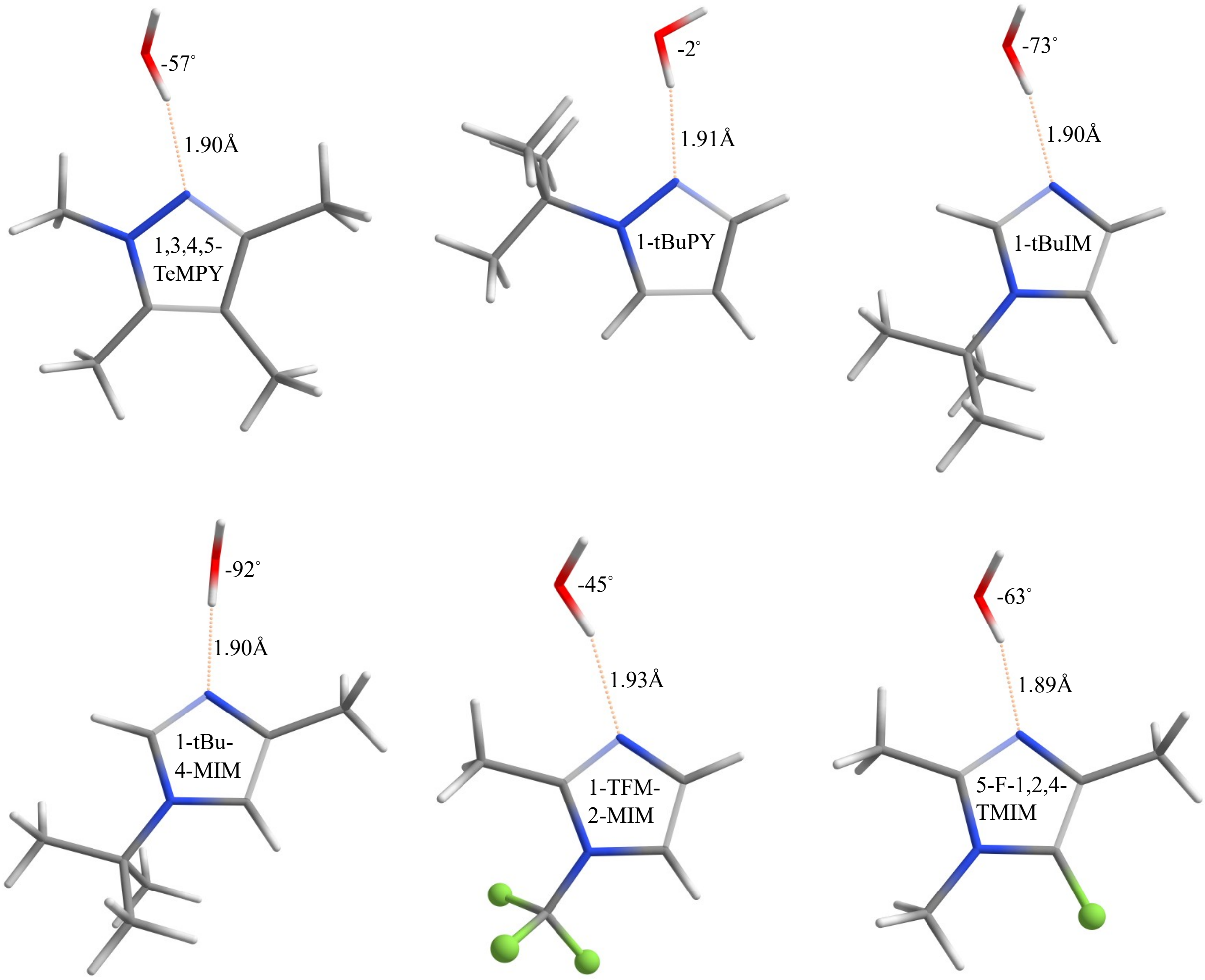


**Figure S48:** Optimized structures of 6 new monohydrates: 1,3,4,5-TeMPY, 1-tBuPY, 1-tBuIM, 1-tBu-4-MIM, 1-TFM-2-MIM and 5-F-1,2,4-TMIM at B2PLYP-D3BJ/def2-QZVP (B2QZ) level. Also shown are hydrogen bond lengths (in Å) as well as torsional angles (in degrees) of the free OH group relative to plane spanned by O, N and the adjacent ring C-atom.

**Table S37:** Harmonically predicted wavenumbers for the monohydrate b2ON combination band at different levels of computation. Data pairs in parentheses provide original harmonic wavenumbers where a demixing of the ON stretching fundamental or water bend fundamental b was necessary.

| system | TZ / $cm^{-1}$ | maTZ / $cm^{-1}$ | QZ / $cm^{-1}$ | B2QZ / $cm^{-1}$ |
|---|---|---|---|---|
| 1,3,4,5-TeMPY | 3461 | 3438 | 3485 | 3502 |
| 1-tBuPY | 3460 | 3472 | 3504 | 3523 |
| | (3458 / 3464) | | | |
| 1-tBuIM | 3478 | 3448 | 3498 | 3516 |
| 1-tBu-4-MIM | 3475 | 3439 | 3491 | 3507 |
| 1-TFM-2-MIM | 3454 | 3436 | 3484 | 3503 |
| | (3451 / 3457) | | | (3502 / 3505) |
| 5-F-1,2,4-TMIM | 3470 | 3446 | 3491 | 3509 |

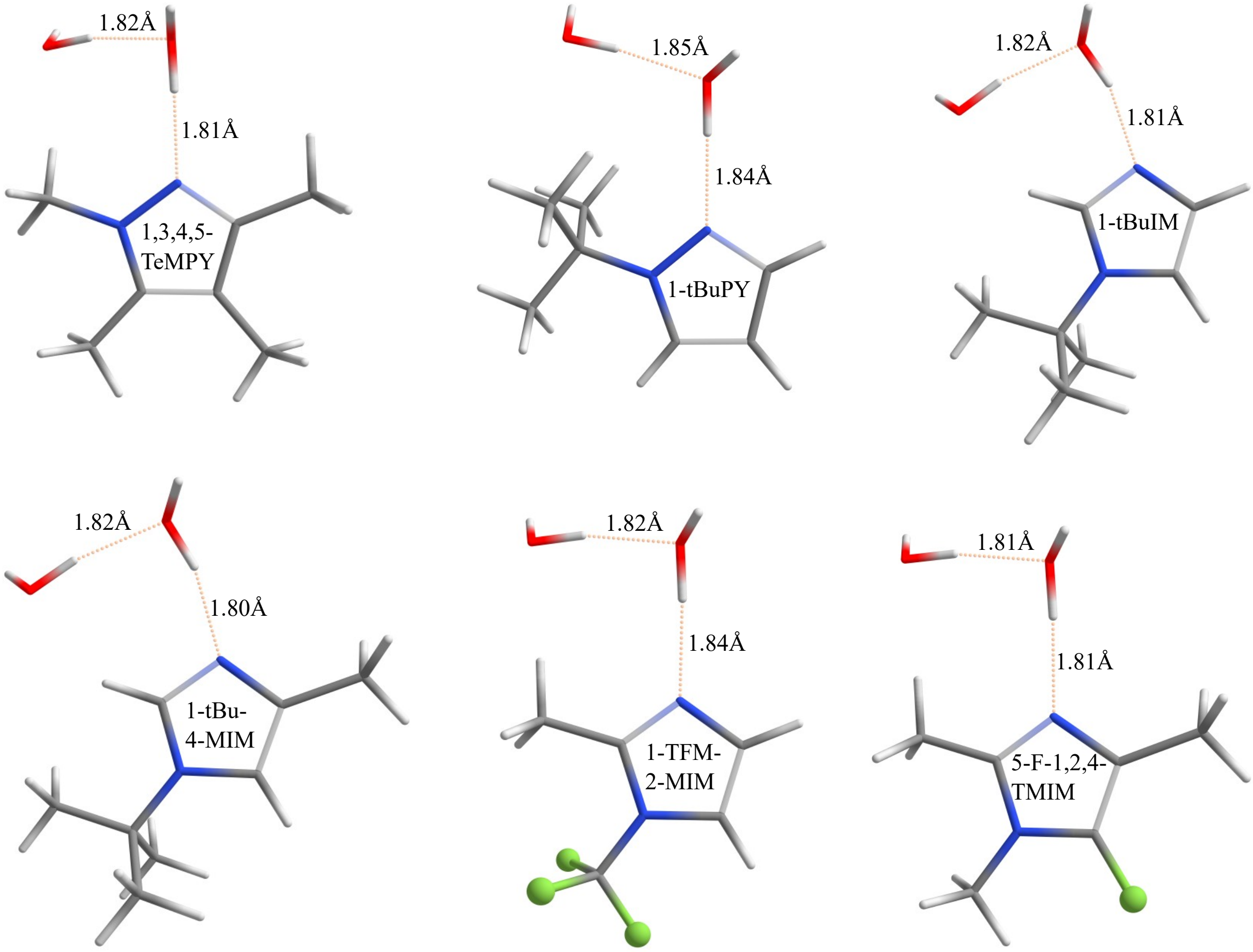


**Figure S49:** Optimized structures of 6 new dihydrates: 1,3,4,5-TeMPY, 1-tBuPY, 1-tBuIM, 1-tBu-4-MIM, 1-TFM-2-MIM and 5-F-1,2,4-TMIM at B2PLYP-D3BJ/def2-QZVP (B2QZ) level. Also shown are hydrogen bond lengths (in Å) for both OH bonds (1:2i and 1:2d). For 1:2i, the hydrogen bond length is taken as OH. . . O, and it is taken as OH. . . N for 1:2d. Note that the 1:2d hydrogen bond lengthens with ring halogenation and shortens with ring methylation.

**Table S38:** Harmonically predicted wavenumbers for the dihydrate (1:2d) OHb stretching vibration at different levels of computation. Because the most strongly downshifted OHb modes start to undergo harmonic mode mixing with CH modes, we decoupled them by mass variation of the H atoms.

| system | TZ / $cm^{-1}$ | maTZ / $cm^{-1}$ | QZ / $cm^{-1}$ | B2QZ / $cm^{-1}$ |
|---|---|---|---|---|
| 1,3,4,5-TeMPY | 3272 | 3256 | 3299 | 3364 |
| 1-tBuPY | 3343 | 3327 | 3364 | 3423 |
| 1-tBuIM | 3251 | 3225 | 3270 | 3338 |
| | (3238 / 3245 / 3254) | (3223 / 3247 / 3248) | | |
| 1-tBu-4-MIM | 3229 | 3201 | 3246 | 3317 |
| | (3228 / 3241) | | (3242 / 3248) | |
| 1-TFM-2-MIM | 3330 | 3314 | 3353 | 3418 |
| 5-F-1,2,4-TMIM | 3237 | 3216 | 3261 | 3331 |

**Table S39:** Harmonically predicted wavenumbers for the dihydrate (1:2i) OHb stretching vibration at different levels of computation.

| system | TZ / $cm^{-1}$ | maTZ / $cm^{-1}$ | QZ / $cm^{-1}$ | B2QZ / $cm^{-1}$ |
|---|---|---|---|---|
| 1,3,4,5-TeMPY | 3507 | 3497 | 3523 | 3579 |
| 1-tBuPY | 3555 | 3549 | 3572 | 3625 |
| 1-tBuIM | 3474 | 3469 | 3494 | 3548 |
| 1-tBu-4-MIM | 3474 | 3469 | 3494 | 3549 |
| 1-TFM-2-MIM | 3512 | 3507 | 3532 | 3589 |
| 5-F-1,2,4-TMIM | 3492 | 3483 | 3511 | 3568 |

**Table S40:** Harmonically predicted wavenumbers for the in-phase water bending overtone b2 of the dihydrate (1:2) at different levels of computation. Data pairs in parentheses provide original harmonic wavenumbers where a demixing of the water bend fundamental b was necessary.

| system | TZ / $cm^{-1}$ | maTZ / $cm^{-1}$ | QZ / $cm^{-1}$ | B2QZ / $cm^{-1}$ |
|---|---|---|---|---|
| 1,3,4,5-TeMPY | 3317 | 3302 | 3337 | 3357 |
| 1-tBuPY | 3329 | 3320 | 3349 | 3368 |
| 1-tBuIM | 3341 | 3333 | 3357 | 3378 |
| 1-tBu-4-MIM | 3340 | 3333 | 3356 | 3378 |
| 1-TFM-2-MIM | 3317 | 3307 | 3340 | 3359 |
| 5-F-1,2,4-TMIM | 3326 | 3318 | 3350 | 3370 |
| | | | (3347 / 3363) | (3366 / 3380) |

**Table S41:** Harmonically predicted wavenumbers for the out-of-phase water bending overtone b2 of the dihydrate (1:2) at different levels of computation.

| system | TZ / $cm^{-1}$ | maTZ / $cm^{-1}$ | QZ / $cm^{-1}$ | B2QZ / $cm^{-1}$ |
|---|---|---|---|---|
| 1,3,4,5-TeMPY | 3284 | 3264 | 3311 | 3329 |
| 1-tBuPY | 3288 | 3276 | 3313 | 3334 |
| 1-tBuIM | 3308 | 3288 | 3322 | 3341 |
| 1-tBu-4-MIM | 3309 | 3288 | 3323 | 3342 |
| 1-TFM-2-MIM | 3294 | 3270 | 3312 | 3330 |
| 5-F-1,2,4-TMIM | 3300 | 3277 | 3318 | 3336 |

**Table S42:** Average over harmonic ip and oop wavenumbers for the water bending overtones b2 of the dihydrate (1:2) at different levels of computation.

| system | TZ / $cm^{-1}$ | maTZ / $cm^{-1}$ | QZ / $cm^{-1}$ | B2QZ / $cm^{-1}$ |
|---|---|---|---|---|
| 1,3,4,5-TeMPY | 3300 | 3283 | 3324 | 3343 |
| 1-tBuPY | 3309 | 3298 | 3331 | 3351 |
| 1-tBuIM | 3325 | 3310 | 3340 | 3359 |
| 1-tBu-4-MIM | 3324 | 3310 | 3340 | 3360 |
| 1-TFM-2-MIM | 3306 | 3288 | 3326 | 3344 |
| 5-F-1,2,4-TMIM | 3313 | 3298 | 3334 | 3353 |

**Table S43:** Predictions of raw experimental wavenumbers for the mono- and dihydrates of 6 experimentally uncharacterized systems using the simple Fermi model predictions building on the B2QZ DFT approach, including the refined B2QZ' variant for 1:2d' of dihydrates. Relative intensities of resonance polyad members rounded to one significant digit for a given species are given in brackets. In the first row, the less rounded relative intensity is 0.55 for 1:2d', justifying the assignment of the nearly degenerate pair of coupled states. As a refinement attempt for strong transitions, the spectrally closest 1:1 training complexes above and below the 1:1 complex for a given new species (in terms of harmonic wavenumber predictions) are identified and the deviation of their experimental value from the model prediction is added to the model prediction for the new species. Also shown are harmonic BEsT guesses, which may deviate strongly from the anharmonic models (marked by bold face) close to the Fermi resonance.

| system | 1:1 | 1:2d | 1:2d' | 1:2i | b2 (1:1) | b2 (1:2d) | b2(1:2d') | b2ON (1:1) |
|---|---|---|---|---|---|---|---|---|
| 1,3,4,5-TeMPY | 3406 [0.9] | 3196 [0.5] | 3184 [0.5] | 3469 [1.0] | 3190 [0.04] | 3276 [0.5] | 3264 [0.5] | 3334 [0.04] |
| w 1,2-DMIM | 3403 | 3172 | 3184 | 3470 | 3199 | | | 3333 |
| w 5-Cl-1,2-DMIM | 3402 | 3185 | 3186 | 3468 | 3196 | | | |
| BEsT guess (2) | 3407 | 3190 | 3190 | 3469 | 3197 | | | |
| 1-tBuPY | 3422 [0.9] | 3316 [0.8] | 3313 [0.8] | 3515 [1.0] | 3207 [0.04] | 3223 [0.2] | 3215 [0.2] | 3355 [0.05] |
| w 1,3,5-TMPY | 3423 | 3306 | 3314 | 3512 | 3221 | 3213 | 3216 | |
| w 1-MIM | 3430 | 3294 | 3303 | 3515 | 3218 | | | |
| BEsT guess (2) | 3426 | **3237** | **3237** | 3512 | 3219 | | | |
| 1-tBuIM | 3419 [0.9] | 3185 [0.8] | 3167 [0.8] | 3438 [1.0] | 3200 [0.04] | 3277 [0.2] | 3265 [0.2] | 3347 [0.04] |
| w 1,3,5-TMPY | 3420 | 3175 | 3168 | 3435 | 3214 | 3267 | 3266 | |
| w 1-MIM | 3427 | 3163 | 3157 | 3438 | 3211 | | | |
| BEsT guess (2) | 3423 | 3156 | 3156 | 3437 | 3212 | | | |
| 1-tBu-4-MIM | 3399 [0.9] | 3168 [0.8] | 3145 [0.9] | 3439 [1.0] | 3199 [0.04] | 3273 [0.2] | 3261 [0.1] | 3338 [0.06] |
| w 1,2-DMIM | 3396 | 3144 | 3145 | 3440 | 3208 | | | 3337 |
| w 5-Cl-1,2-DMIM | 3395 | 3157 | 3147 | 3438 | 3205 | | | |
| BEsT guess (2) | 3399 | 3145 | 3145 | 3439 | 3206 | | | |
| 1-TFM-2-MIM | 3449 [0.9] | 3310 [0.8] | 3307 [0.8] | 3479 [1.0] | 3193 [0.03] | 3217 [0.2] | 3209 [0.2] | 3336 [0.02] |
| w 1,4-DMPY | 3451 | 3303 | 3302 | 3483 | 3198 | 3205 | 3205 | |
| w 1-MPY | 3450 | 3304 | 3301 | 3482 | 3196 | 3201 | 3201 | |
| BEsT guess (2) | 3451 | 3302 | 3302 | 3482 | 3198 | | | |
| 5-F-1,2,4-TMIM | 3391 [0.9] | 3178 [0.8] | 3159 [0.8] | 3458 [1.0] | 3195 [0.05] | 3271 [0.2] | 3258 [0.2] | 3339 [0.09] |
| w 1,2-DMIM | 3388 | 3154 | 3159 | 3459 | 3204 | | | 3338 |
| w 5-Cl-1,2-DMIM | 3387 | 3167 | 3161 | 3457 | 3201 | | | |
| BEsT guess (2) | 3390 | 3158 | 3158 | 3458 | 3202 | | | |

**Table S44:** Predictions of raw experimental OHb wavenumbers (in $cm^{-1}$) for the monohydrates of 6 experimentally uncharacterized systems using the simple Fermi model predictions building on the TZ DFT approach. Relative intensities of resonance polyad members rounded to one significant digit for a given species are given in brackets. As a refinement attempt for strong transitions, the spectrally closest 1:1 training complexes above and below the 1:1 complex for a given new species (in terms of harmonic wavenumber predictions) are identified and the deviation of their experimental value from the model prediction is added to the model prediction for the new species. Also shown are harmonic BEsT guesses.

| system | 1:1 | b2 (1:1) | b2ON (1:1) |
|---|---|---|---|
| 1,3,4,5-TeMPY | 3408 [0.9] | 3208 [0.04] | 3333 [0.04] |
| w 1,2-DMIM | 3403 | 3200 | 3333 |
| w 5-Cl-1,2-DMIM | 3403 | 3198 | |
| BEsT guess (2) | 3406 | 3197 | |
| 1-tBuPY | 3422 [0.9] | 3204 [0.04] | 3332 [0.03] |
| w 1,3,5-TMPY | 3425 | 3203 | |
| w 1,3-DMPY | 3424 | 3199 | |
| BEsT guess (2) | 3425 | 3201 | |
| 1-tBuIM | 3432 [0.9] | 3221 [0.04] | 3350 [0.03] |
| w 1,3,5-TMPY | 3435 | 3220 | |
| w 1,3-DMPY | 3434 | 3216 | |
| BEsT guess (2) | 3433 | 3217 | |
| 1-tBu-4-MIM | 3409 [0.9] | 3225 [0.05] | 3346 [0.06] |
| w 1,2-DMIM | 3404 | 3217 | 3346 |
| w 5-Cl-1,2-DMIM | 3404 | 3215 | |
| BEsT guess (2) | 3406 | 3215 | |
| 1-TFM-2-MIM | 3451 [0.9] | 3206 [0.03] | 3327 [0.02] |
| w 1-MIM | 3444 | 3196 | |
| w 1-MPY | 3452 | 3198 | |
| BEsT guess (2) | 3449 | 3197 | |
| 5-F-1,2,4-TMIM | 3394 [0.9] | 3215 [0.06] | 3340 [0.08] |
| w 1,2-DMIM | 3389 | 3207 | 3340 |
| w 5-Cl-1,2-DMIM | 3389 | 3205 | |
| BEsT guess (2) | 3391 | 3205 | |

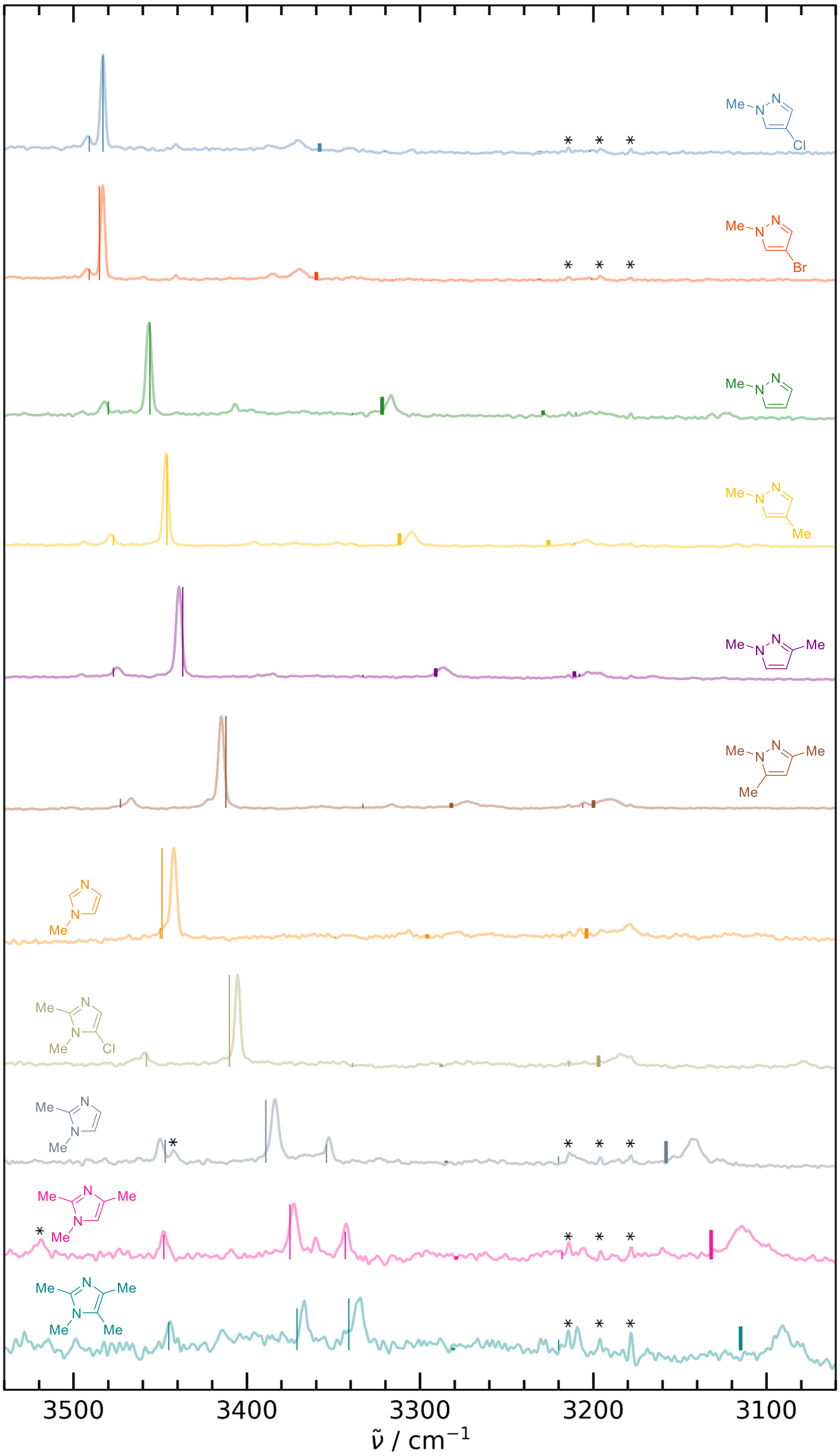


**Figure S50:** As Fig. 5 in the main text (B2QZ'), but using the TZ model. One can see that the performance is considerably inferior for the dihydrates than the the B2QZ' model. The shown spectra are indicated in Tables S3 and S4.

The plain anharmonic model predictions are refined by choosing for each new compound the two closest training members (for the 1:1 OH b transition, preferentially from both sides) w and by correcting the anharmonic model prediction by the model errors experienced in these training members. Furthermore, a purely harmonic BEsT guess is made, which derives an average harmonic scaling factor for the two closest training members and applies this average scaling factor to the harmonic prediction for the complex of the new compound.

Note that these are truly blind predictions, because the compounds are currently synthesized or stocked in another research group and will only be transferred to our spectroscopy laboratories after this manuscript has been prepublished.

A non-blind test of the 1:1 models is offered by the monohydrate OHb of plain imidazole, which has been observed [33] at 3458 $cm^{-1}$. The anharmonic B2QZ model predicts it at 3452 $cm^{-1}$, including [$P = 0.9$], based on the harmonic values for OHb, b2 and b2ON of 3589 $cm^{-1}$, 3344 $cm^{-1}$ and 3509 $cm^{-1}$. The two harmonically closest training systems are 1-MPY and 1,4-DMPY, and therefore the best anharmonic predictions based on their deviation from the model are 3453 $cm^{-1}$ and 3454 $cm^{-1}$, respectively. This may be compared to the BEsT harmonic B2QZ guess of 3454 $cm^{-1}$. The anharmonic TZ model predicts OHb at 3462 $cm^{-1}$, including [$P = 0.9$], based on the harmonic values for OHb, b2 and b2ON of 3538 $cm^{-1}$, 3306 $cm^{-1}$ and 3471 $cm^{-1}$. With the two harmonically closest training systems for the 1:1 complex, the best anharmonic predictions are 3462 $cm^{-1}$ and 3463 $cm^{-1}$. This may be compared to the BEsT harmonic TZ guess of 3462 $cm^{-1}$. If one considers that all additive model parameters are multiples of 5 $cm^{-1}$, it is reasonable to give the best prediction in steps of 5 $cm^{-1}$ as well. Independent of whether one weighs the B2QZ guess higher (3455 $cm^{-1}$) or gives the TZ and B2QZ predictions equal weight (3460 $cm^{-1}$), the agreement with experiment (3458 $cm^{-1}$) is excellent. Because resonances are relatively far away, a harmonic BEsT guess without any resonance modelling performs equally well.

Another non-blind test of all models is offered by 4-iodo-1-methylpyrazole, which was part of the recent HyDRA II blind challenge. [28] It is not very challenging because there are two very closely related halogen variants in the training set, such that both the anharmonic and the harmonic interpolations should work well. Depending on the computational level, their 1:1 complexes with water are both predicted at lower wavenumber (B2QZ) or to each side of the 4-iodo-1-methylpyrazole monohydrate prediction (TZ). We use these homologs for the refined predictions. The monohydrate OHb of 4-iodo-1-methylpyrazole has been observed [28] at 3482 $cm^{-1}$. The anharmonic B2QZ model predicts it at 3483 $cm^{-1}$, including [$P = 0.9$], based on the harmonic values for OHb, b2 and b2ON of 3622 $cm^{-1}$, 3340 $cm^{-1}$ and 3485 $cm^{-1}$. The two harmonically closest training systems are 4-Cl-1-MPY and 4-Br-1-MPY, and therefore the best anharmonic predictions based on their deviation from the model are 3483 $cm^{-1}$ and 3482 $cm^{-1}$, respectively. This may be compared to the BEsT harmonic B2QZ guess of 3483 $cm^{-1}$. The anharmonic TZ model predicts OHb at 3484 $cm^{-1}$, including [$P = 0.9$], based on the harmonic values for OHb, b2 and b2ON of 3562 $cm^{-1}$, 3292 $cm^{-1}$ and 3442 $cm^{-1}$. The two harmonically closest training systems are again 4-Cl-1-MPY and 4-Br-1-MPY, and therefore the best anharmonic predictions are 3484 $cm^{-1}$ and 3483 $cm^{-1}$. This may be compared to the BEsT harmonic TZ guess of 3483 $cm^{-1}$. Thus, all different model predictions converge to the experiment within 2 $cm^{-1}$.

The dihydrate 1:2d of 4-iodo-1-methylpyrazole has been observed [28] at 3367 $cm^{-1}$. The anharmonic B2QZ' model predicts it at 3367 $cm^{-1}$, including [$P = 0.9$], based on the harmonic values for 1:2d and b2 of 3475 $cm^{-1}$ and 3337 $cm^{-1}$. With the two harmonically closest 1:1 training systems, the best anharmonic predictions based on their deviation from the model are 3370 $cm^{-1}$ and 3367 $cm^{-1}$, respectively. This may be compared to the BEsT harmonic B2QZ guess of 3369 $cm^{-1}$. The dihydrate 1:2i of 4-iodo-1-methylpyrazole has been observed [28] at 3492 $cm^{-1}$. The anharmonic B2QZ model predicts it at 3489 $cm^{-1}$, including [$P = 1$], based on the harmonic value for 1:2i of 3599 $cm^{-1}$. With the two harmonically closest training systems for the 1:1 complex, the best anharmonic predictions based on their deviation from the model are 3492 $cm^{-1}$ and 3491 $cm^{-1}$, respectively. This may be compared to the BEsT harmonic B2QZ guess of 3492 $cm^{-1}$. Again, all different model predictions converge to experiment.

# References


[1] V. L. S. Freitas, M. D. M. C. Ribeiro da Silva in *The chemistry of nitrogen-rich functional groups*, (Eds.: A. Greer, J. F. Liebman), John Wiley & Sons, **2020**, pp. 287–328.
[2] A. R. Almeida, M. J. Monte, *J. Chem. Thermodyn.* **2014**, *77*, 46–53.
[3] A. R. Almeida, M. J. Monte, *J. Chem. Thermodyn.* **2012**, *44*, 163–168.
[4] W. L. Armarego, *Purification of laboratory chemicals, 7th ed.* Butterworth-Heinemann, **2013**.
[5] E. D. Tidwell, E. K. Plyler, W. S. Benedict, *J. Opt. Soc. Am.* **1960**, *50*, 1243–1263.
[6] P. Jimenez, M. Roux, C. Turrión, F. Gomis, *J. Chem. Thermodyn.* **1987**, *19*, 985–992.
[7] H. C. Gottschalk, T. L. Fischer, V. Meyer, R. Hildebrandt, U. Schmitt, M. A. Suhm, *Instruments* **2021**, *5*, 12.
[8] E. Lwin, M. J. Gölz, N. O. B. Lüttschwager, M. A. Suhm, S. Käser, V. Andreichev, M. A. Brandes, M. Meuwly, *Phys. Chem. Chem. Phys.* **2025**, *27*, 17692–17703.
[9] K. E. Otto, Z. Xue, P. Zielke, M. A. Suhm, *Phys. Chem. Chem. Phys.* **2014**, *16*, 9849–9858.
[10] S. Grimme, J. Antony, S. Ehrlich, H. Krieg, *J. Chem. Phys.* **2010**, *132*, 154104.
[11] S. Grimme, S. Ehrlich, L. Goerigk, *J. Comput. Chem.* **2011**, *32*, 1456–1465.
[12] A. D. Becke, E. R. Johnson, *J. Chem. Phys.* **2005**, *123*, 154101.
[13] E. R. Johnson, A. D. Becke, *J. Chem. Phys.* **2005**, *123*, 24101.
[14] E. R. Johnson, A. D. Becke, *J. Chem. Phys.* **2006**, *124*, 174104.
[15] A. D. Becke, *Phys. Rev. A* **1988**, *38*, 3098–3100.

[16] C. Lee, W. Yang, R. G. Parr, *Phys. Rev. B* **1988**, *37*, 785–789.
[17] B. Miehlich, A. Savin, H. Stoll, H. Preuss, *Chem. Phys. Lett.* **1989**, *157*, 200–206.
[18] S. Grimme, *J. Chem. Phys.* **2006**, *124*, 034108.
[19] F. Neese, *Wiley Interdiscip. Rev. Comput. Mol. Sci.* **2012**, *2*, 73–78.
[20] F. Neese, *Wiley Interdiscip. Rev. Comput. Mol. Sci.* **2022**, *12*, e1606.
[21] F. Neese, F. Wennmohs, U. Becker, C. Riplinger, *J. Chem. Phys.* **2020**, *152*, 224108.
[22] F. Weigend, R. Ahlrichs, *Phys. Chem. Chem. Phys.* **2005**, *7*, 3297–3305.
[23] J. Zheng, X. Xu, D. G. Truhlar, *Theor. Chem. Acc.* **2011**, *128*, 295–305.
[24] C. Bannwarth, S. Ehlert, S. Grimme, *J. Chem. Theory Comput.* **2019**, *15*, 1652–1671.
[25] S. Grimme, C. Bannwarth, P. Shushkov, *J. Chem. Theory Comput.* **2017**, *13*, 1989–2009.
[26] P. Pracht, F. Bohle, S. Grimme, *Phys. Chem. Chem. Phys.* **2020**, *22*, 7169–7192.
[27] S. Grimme, *J. Chem. Theory Comput.* **2019**, *15*, 2847–2862.
[28] M. Bödecker, E. Lwin, F. Knüppe, N. O. Evers, E. Sennert, M. Niessner, N. O. B. Lüttschwager, M. A. Suhm, *Phys. Chem. Chem. Phys.* **2026**, 14373–14381.
[29] N. O. B. Lüttschwager, *Phys. Chem. Chem. Phys.* **2024**, *26*, 10120–10135.
[30] N. O. B. Lüttschwager, *Journal of Open Source Software* **2021**, *6*, 3526.
[31] E. Lwin, N. O. B. Lüttschwager, M. A. Suhm, *Phys. Chem. Chem. Phys.* **2025**, *27*, 5808–5820.
[32] G. Herzberg, *Molecular spectra and molecular structure. II. Infrared and Raman spectra of polyatomic molecules*, van Nostrand, **1945**.
[33] J. Zischang, J. J. Lee, M. A. Suhm, *J. Chem. Phys.* **2011**, *135*, 061102.
